# Palaeoproteomics and microanalysis reveal techniques of production of animal-based metal threads in medieval textiles

**DOI:** 10.1038/s41598-024-54480-4

**Published:** 2024-03-04

**Authors:** Cristina Scibè, Kira Eng-Wilmot, Thomas Lam, Isetta Tosini, Maria José González López, Caroline Solazzo

**Affiliations:** 1 Programa de Doctorado Arte y Patrimonio, Escuela Internacional de Doctorado (EIDUS), Universidad de Sevilla, P de Las Delicias s/n, 41003 Sevilla, Spain; 2 Scientific Laboratory Opificio delle Pietre Dure (OPD), Viale F. Strozzi 1, 50129 Firenze, Italy; 3 Cooper Hewitt, Smithsonian Design Museum, 2 E 91st St, New York, NY 10128 USA; 4 Museum Conservation Institute (MCI), Smithsonian Institution, 4210 Silver Hill Road, Suitland, MD 20746 USA; 5 Departamento de Pintura, Facultad de Bellas Artes, Universidad de Sevilla, C/ Laraña 3, 41003 Sevilla, Spain

**Keywords:** Metal threads, Skin, Membrane, Gold, Collagen, Egg white, Sturgeon glue, Human blood, Characterization and analytical techniques, Characterization and analytical techniques

## Abstract

Animal-based metal threads were largely used between the 10th and the fifteenth century, in European, Middle Eastern and Far Eastern textile productions for the decoration of textiles and cloths. They belong to a larger group of metal threads, used either as flat threads or wrapped around a fiber core, that were backed by an organic support (animal or paper). This study focuses on the medieval production of metal threads backed by an animal membrane (e.g. gut membrane), or skin. A total of 91 samples were collected from a corpus of 66 textile fragments belonging to 54 catalogued objects. The relevance and novelty of the present study is represented by the combination of proteomics, cross-section analysis, and scanning electron microscopy (SEM–EDS and SEM-µXRF). The diversity of materials and manufacturing techniques found within each typology of thread, respectively, *membrane-based metal threads* and *skin-based metal threads,* hinted at different production technologies. Membrane-based threads were found to be invariably made from cattle gut membrane, coated with gilt-silver leaves. A possible sheep glue adhesive was found in a few samples. Skin-based threads were made from either goat or sheep leather, coated with metal leaves or powder. Within the three different types of coatings identified (silver, gold and gilt-silver), gold coatings were the most represented. Goat leather threads were associated with an egg-white binder, while sturgeon glue was identified as adhesive in all sheep leather threads. Collagen glue from other species (cattle, sheep, horse) was occasionally found in mixed adhesives. In two textiles, the finding of human proteins indicates past contamination due to handling or use. The analytical results show coherence between the fabrication patterns of animal-based metal threads and their probable geographical areas of manufacture, indicating that the study of materials and techniques provide further criteria to classify and group textiles, and trace correlations between manufacturing centers within Eurasian territories.

## Introduction

The embellishment of textiles with metals, most particular gold elements, in the form of appliqués or threads (either flat lamella or filé) can be traced to early cultures. Throughout the middle ages, a variety of metal elements has been used for the decoration of textiles and cloths to create luxurious fabrics and garments emblematic of wealth, power and status. As Járó (2003) pointed out^1^, the term “metal thread” was and is still used as a collective term to indicate every type of yarn-like textile decoration made by a metal or a metal-coated organic material. The CIETA (Centre International d’Etude des Textiles Anciens), indeed, defines “metal threads” as “A general term for threads composed partly or entirely of metal or metallic materials”^2^. Indictor et al.^3^ identified five categories to describe and distinguish the use of metals in textiles: (I) Metal applied (with adhesive) to already woven fabrics; (II) Metal wire or flattened strips; (III) Metal wire or flattened strips wound around fiber core; (IV) Metallic surface applied (with adhesive) to organic wrapping, (IVa) cellulosic or (IVb) proteinaceous, wound around fiber core; and (V) Metallic surface applied (with adhesive) to organic strips, (Va) cellulosic or (Vb) proteinaceous. The present research treats exclusively with categories IVb and Vb.

The nomenclature of the different types of metal threads was beyond the purpose of the present work and will be dealt in a later publication. In order to simplify the terminology, the general term “metal threads” is here adopted for every type of yarn-like metal element, either flat or wrapped around a fibrous core. When the metal thread is made by a metal-coated strip of organic nature (Indictor et al. categories IV and V), it is named “organic-based metal thread”, and “animal-based metal thread” to refer to the sub-categories IVb and Vb, specifically “wrapped lamella or strip” and “flat lamella or strip”. According to the CIETA, indeed, the definition of the term “lamella” is “A narrow strip of precious or base metal, or gilt or silvered leather, membrane, metal or paper used for thread. It may be used flat or wound around a core”^2^. When presenting the analytical results, a further distinction is made according to the nature of the animal-based strip as “membrane-based metal threads” and “skin-based metal threads”.

Between the 10th and the fifteenth century, organic-based metal threads were extensively used in medieval textiles decoration^4^. They were made by gilding/silver-plating an organic substrate, usually by an adhesive medium, likely then cut into narrow strips, thus forming the lamellas which were used either as flat or wound around a fibrous core, probably by a handmade process. As mentioned above, two sub-categories of organic-based strips can be distinguished^3,5^: protein-based (animal gut or membrane, vellum, parchment, or leather) and cellulose-based (paper). Their introduction represents a very important development and achievement in metal threads and textile technology, due to their flexibility, lightweight in weaving, and reduced price compared to the earlier metallic threads made of almost pure gold^4,6–8^. In European and Middle Eastern textiles, animal-based strips were exclusively wound around a fibrous core, in Z or S direction, in the production of filé metal threads, while in East and Central Asian fabrics, paper and skin-based strips were used as both wrapped and flat^9^.

To date, defining exactly the origin, geographical distribution and timeline of this type of threads is still challenging, as often earlier examples did not survive, and the nomenclature used in written sources is misleading. According to the scientific literature, the earliest evidence of animal-based metal threads of European production appear to be gilt-skin threads examples in 10th to eleventh century textiles attributed to al-Andalus workshops of Cordoba (the “Pyrenees” peacock tapestry and the veil of almaizar Hisham II)^10^ and Almeria (the shroud of St Lazarus from Autun) in modern Spain^11,12^. Animal-based metal threads may have been introduced in the Mediterranean countries of Europe with the Levantine trade, possibly transiting through the ports of Cyprus, hence the name “Cyprus gold” by which they became known^6,7,12^. Organic-based metal threads were historically and improperly known under the name “membrane threads” or “Cypriot gold” (referring to threads made with strips of either skin or animal gut, and sometimes including paper too), leading to a lack of clarity as what type of thread is referred to. However, as Jacoby (2014) pointed out, no organic-based “Cypriot” gold thread has been securely identified so far^11^; moreover, Monnas (2019) clearly showed that “Cyprus gold threads”, largely used between the thirteenth and fourteenth century in English embroidery, were made of silver-gilt *filé* thread with a silk core and not of membrane substrates like the “Cologne” and “Lucca” gold. These threads when new may have looked similar to Cyprus gold, which was considered of better quality, thereby also called “fine gold”^13^. In China, there is evidence of textiles with gilded paper wrapped threads from the eighth century^14^, and both paper and animal substrates were used as flat metal threads from the 10-eleventh century^15^. Paper and animal threads were found as far as the Western parts of Central Asia prior to the Mongol conquest^14^. Starting in the thirteenth century with the Mongol period, animal strips (mostly skin-based) wrapped around a fiber core, rather than flat, were introduced in the weaving centers of Yuan China^16^. From the 1260s, the textile production was dominated by the luxurious *Panni Tartarici* (Tartar fabrics) or *Cloths of gold* produced in Mongol-ruled territories^14^, that soon flooded the European Markets^17^. The growing Western demand of the oriental luxury silks might have required an additional supply of gold threads, well-documented in commercial Italian records (especially Genoese and Venetian)^11^. This demand may also have led to the production of a less expensive type of gold threads in European workshops, developed especially in Italy and Germany, using animal membranes (from internal organs, usually guts) coated with gilt-silver^18^. From the fifteenth century onwards, apart from a few examples on early renaissance velvets^19,20^, organic-based metal threads were no longer produced in Europe and were gradually replaced by base metal threads (no substrate) for wrapped threads. In the Far East, skin-based metal threads were still in use into the twentieth century^21^, although not as common as paper ones^4^.

Bock’s treatise of 1884 on gold threads^6^ was the first attempt to scientifically describe and classify the different types of metal threads, followed in the 1990s by extensive scientific works, mainly on base metal threads^3–5,7,9,18,19^, still today the focus of advanced analytical applications^22–28^. Characterizing organic-based metal threads turned out to be challenging due to the extremely thin metal coating, often just partly preserved on the organic substrate. Besides the most common investigation of the metal component^3,7,9,19,23,29–34^, an early attempt to identify the strips’ animal substrates with DNA was met with mixed success due to the extensive fragmentation of the DNA molecules and the complex composition of the threads^35^. A more comprehensive study^10^ was recently conducted on a large set of Hispano-Islamic animal-based gilt threads by high performance liquid chromatography (HPLC-DAD-QTOF-MS), identifying vegetable tannins and dyes (*Rhus coriaria* and *Reseda luteola*) in skin-based threads and fatty acids of uncertain nature in membrane-based threads, but not the nature of the substrates. The advantages of proteomics to study skin tissues such as leather and parchment have been demonstrated over the past years, either for species identification of the skin^36–42^ or the identification of other proteins associated or deposited on skin artifacts^43–45^. Studies on gilded animal membrane replicas^46^ and historical leather strips^21^ showed that proteomics was the ideal method for both species identification of the base layer and for the characterization of the protein adhesive, when present.

### Scope of the study

The present work focuses on metal threads made with an animal-based lamella from European (mainly Italian and Spanish) and Oriental textile objects (Middle Eastern pieces regarded as been made in Iran or Iraq by Persian artisans, and East and Central Asian objects). The present research is based on the assumption that the study of metal threads, here specifically animal-based metal threads, may bring further criteria to classify and group textiles, tracing connections between a certain type of metal thread and its use in a certain period and geographical area, without overlooking the intense trade of them as raw materials.

**Figure 1:**
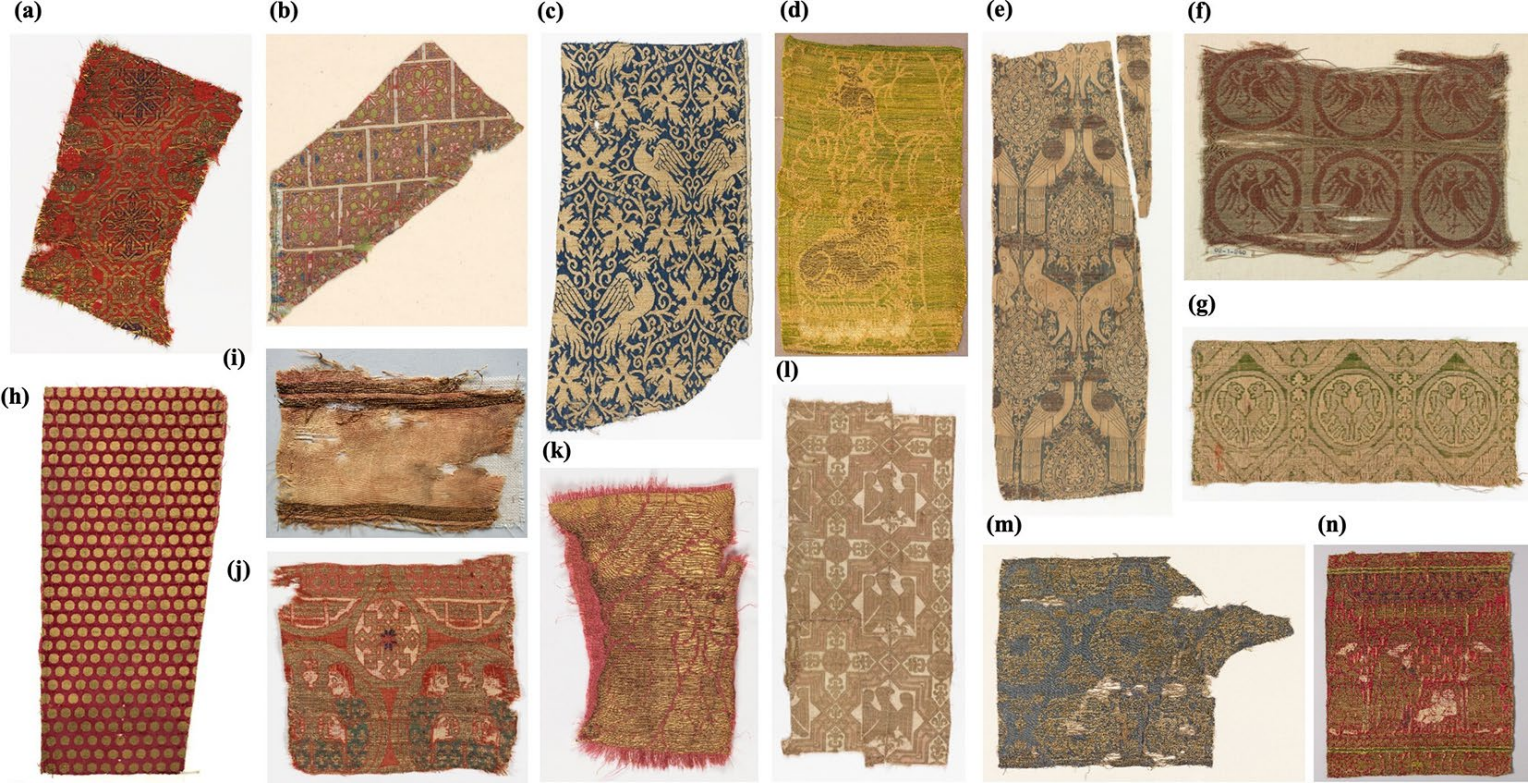
Textile fragments. Some of the textile fragments sampled: (**a**) 1902-1-310 (Lampas); (**b**) 1938-78-1 (Pseudo-lampas); (**c**) 1902-1-271a (Lampas); (**d**) 81.01.01bis (Lampas); (**e**) 1938-84-1 (Lampas, “Diasprum”); (**f**) 1902-1-240 (Samite); (**g**) 1902-1-241a (Samite, half-silk); (**h**) 1901-1-385 (Cut velvet); (**i**) 5968 (Tabby, “Sendal”); (**j**) 1965-33-5 (Pseudo-lampas); (**k**) 1902-1-233 (Lampas); (**l**) 1902-1-253a (Samite); (**m**) 1902-1-279 (Lampas); (**n**) I I161 (Lampas). Photos from a, b, c, e, f, g, h, j, k, l, m © Cooper Hewitt Smithsonian Design Museum; d © Museo del Tessuto; i © Museu Tèxtil, formerly Centre de Documentació i Museu Tèxtil; n © Collezione Tessile Gandini, Museo Civico.

A total of 91 samples were collected from 54 catalogued objects, often mentioning the presence of “metal threads” or the generic term “membrane threads” without further precision. Only a few cases reported “skin/leather threads”. The threads were then separated into two categories according to the strip type observed macroscopically: 40 samples were of the membrane type (made with the membrane of animal intestines or other internal organs) and 51 samples of skin type (9 of them used as flat strips and the rest wrapped around a fiber core). The textiles range from the eleventh-twelfth century to the sixteenth century, with a majority from the thirteenth-fourteenth century. The objects under investigation represent a range of weave structures: while the majority of them are lampas weave (Fig. 1a-e,j,k,m,n), some pieces also feature samite (including half-silks) (Fig. 1f,g,l), taqueté, tabby (Fig. 1i), twill, as well as two similar examples of velvet (Fig. 1h), and a tapestry. They include mostly textile fragments, but also fabric parts of five dalmatics and six copes, and a relic purse (*aumônière*). For a detailed description of textile objects and corresponding sample corpus, see Table 1 and Supplementary SI-1.A.

**Table 1:** Summary of the analyzed set of animal-based metal threads.

Origin	Date	Skin-based thread	Membrane-based thread	Museum
Spain	11th–12th c	1965-33-2		CH
	12th–13th c	313	6372	CDMT
		1902–1-216, 1965–33-5 (Fig. 1j)		CH
	13th c	5776		CDMT
		I A5bis		CG
		1902–1-229b, 1902–1-977c, 1938–78-1 (Fig. 1b), 1943–20-1b	1902–1-240 (Fig. 1f), 1902–1-241a (Fig. 1g), 1902–1-253a (Fig. 1l)	CH
	14th c	1902–1-310 (Fig. 1a), 1902–1-311		CH
possibly Spain	12th-13th c	5968 (Fig. 1i), 6369b		CDMT
	15th c	1902–1-251		CH
Italy	13th c		1902–1-227	CH
	14th c		I I161 (Fig. 1n)	CG
		1902–1-262, 1902–1-271a (Fig. 1c), 1902–1-272, 1902–1-273a, 1902–1-273b, 1902–1-292a, 1902–1-292b	1902–1-250, 1902–1-257b, 1902–1-257d, 1902–1-279 (Fig. 1m), 1902–1-329a, 1938–84-1 (Fig. 1e)	CH
			D10, D11, D13e, P7i, P7k, P10, P11	DB
			81.01.01bis (Fig. 1d)	MTP
	14th–15th c		5798	CDMT
		I A7		CG
		1902–1-285	1902–1-274a, 1902–1-274b	CH
	15th c		P7l, P9	DB
	around 16th c		P12	DB
Italy or Flanders	13th-14th c		3RU8457	CC
Germany	14th c		P1	DB
Iran	possibly 13th c	1902–1-385 (Fig. 1h)		CH
Spain or Iran	14th c	1902–1-233 (Fig. 1k)		CH
Middle East, Persia (Iran or Iraq)	13th-14th c		5963	CDMT
	around 14th c	D12b		DB
	possibly 14th c	164		CDMT
	14th c	03.02.02		MTP
Far East	13th c	D13a		DB
China	13th c	P4c, P4d		DB
Central Asia/Northern China	14th c	1862:16 I, 1862:16 II, 1862:16 III, 1862:16 IV, 1862:16 V		SM

List of the objects under investigation according to the type of metal thread identified (skin-based or membrane-based) based on the macroscopic and microscopic examination of the samples. For each object the attribution of origin and date, as reported in the corresponding museum’s catalogue entries, is indicated together with the acronym of the corresponding museum collection. CC = Ente Chiesa Cattedrale di Como (Como, Italy), samples provided by the Abegg-Stiftung Foundation of Riggisberg; CDMT = Museu Tèxtil, formerly Centre de Documentació i Museu Tèxtil (Terrassa, Spain); CG = Collezione Tessile Gandini, Museo Civico (Modena, Italy); CH = Cooper Hewitt Smithsonian Design Museum (New York, US); DB = Domstift Brandenburg (Brandenburg/Havel, Germany); MTP = Museo del Tessuto (Prato, Italy); SM = Stralsund Museum (Stralsund, Germany).

SEM–EDS and SEM-µXRF were used to characterize the metal surface. In selected cases, stratigraphic observation of the cross-sections by fluorescence microscopy shed light into the multi-layered structure of the threads. And finally, proteomics enabled the identification of both the strip substrate and protein components of the adhesives (detail of the analytical plan in Supplementary SI-1.B). The fiber identification of the cores was conducted in the present study mostly by a close-up examination, thus only a preliminary identification of raw materials is reported here.

## Results

### Membrane-based metal threads

**Figure 2:**
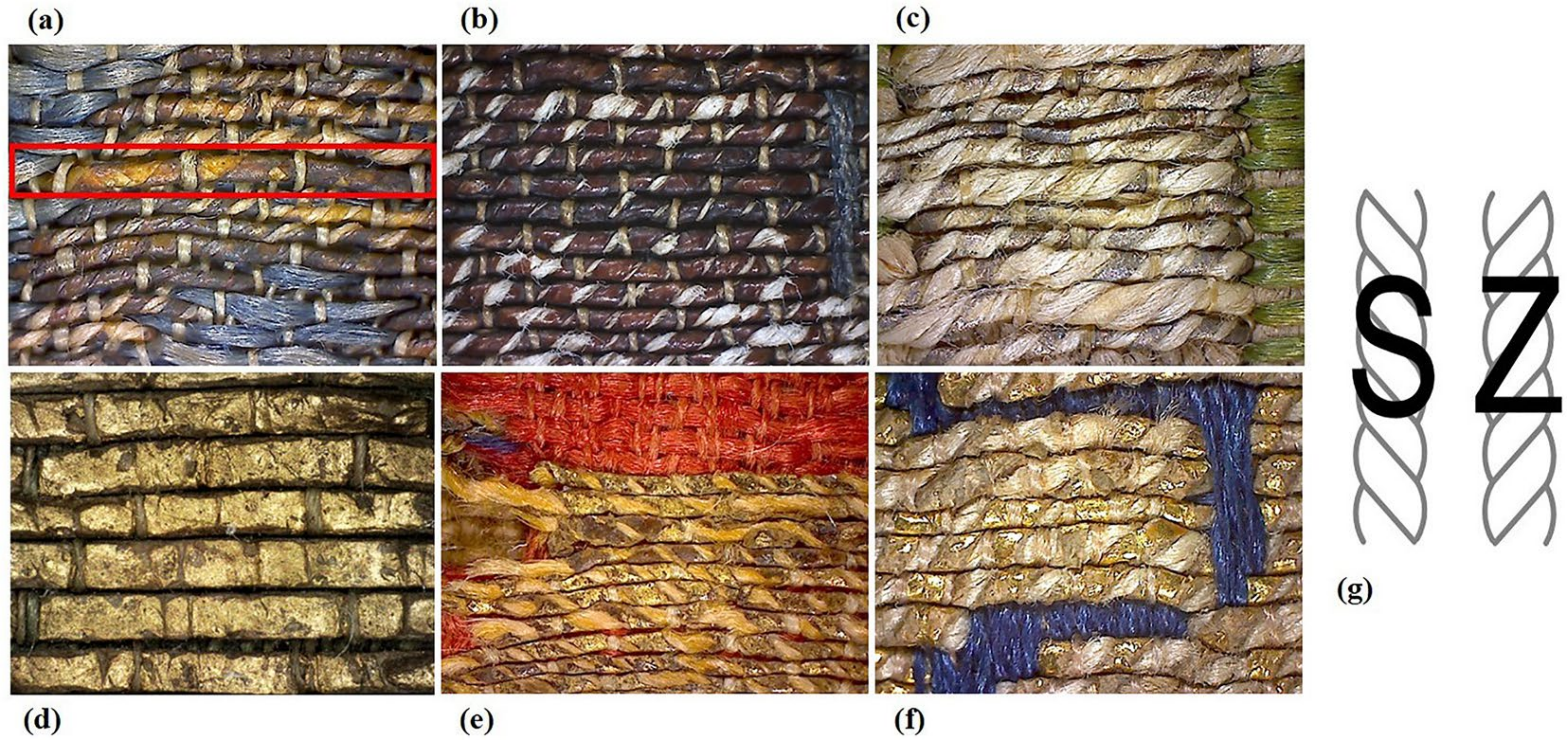
Animal-based metal threads visual and technical features. Detail images of the obverse of the weaves (Photos a, b, c, e and f by Cristina Scibè © Museum Conservation Institute, Smithsonian Institution). Membrane-based metal threads: (**a**) Gilt-silver membrane-based threads, coating largely tarnished and worn, yellow-ochre color of the membranous strips, strips single wrapped in S-direction and double wrapped in S- and Z-direction around a 2-ply core of ecru plant fibers (possibly linen), 1902-1-227, Italy, thirteenth century; (**b**) Gilt-silver membrane-based threads, coating largely tarnished and worn, reddish-brown color of the membranous strips, strips S wound around a 2-ply core of ecru plant fibers (possibly linen), 1938-84-1, Italy, fourteenth century; (**c**) Gilt-silver membrane-based threads, coating partly preserved, tan color of the membranous strips, strips S wound around a 2-ply core of ecru plant fibers (possibly linen), 1902-1-241a, Spain, thirteenth century; Skin-based metal threads: (**d**) Gilt skin-based flat strips, P4d, China, thirteenth century (Photo courtesy of Geertje Gerhold © Domstift Brandenburg, Brandenburg/Havel, Germany); (**e**) Gilt skin-based Z wound strips around a single-ply core of yellow and tan silk fibers, 1965-33-5, Spain, twelfth-thirteenth century; (**f**) Gilt skin-based S wound strips around a 3-ply core of ecru plant fibers (possibly linen), 1902-1-272, Italy, fourteenth century; (**g**) S and Z twist of fibrous yarns (Photo "Yarn twist S-Left Z-Right" @ Dfred (public domain)).

All membrane-based metal threads were made by single-wrapped strips in S-direction around a fibrous core. Only on object 1902-1-227, they were found to be combined with a few double-wrapped strips, one on top of the other in S- and Z-direction (red square in Fig. 2a, twist directions in Fig. 2g). The membranous nature of the strips was easily recognizable at close range by its translucent and tan color (Fig. 2c), exceptionally orange-ochre (1902-1-227, in Fig. 2a) and reddish-brown (1938-84-1, in Fig. 2b), possibly as a result of the degradation of the metallic coating or of the substrate itself. The strip’s width was notably variable, even within the same thread, ranging from 200 to 1130 µm. The fibrous cores were found to be made by two yarns of undyed plant fibers (possibly linen according to a close-up examination of the textile objects) plied together in S-direction (Table 2).

**Table 2:** Membrane-based metal threads analytical results.

Museum’s assignment		Sample	Wrapped strip			Strip thickness (µm)	Gilt-silver leaf coating	Strip Substrate	
Manufacture	Date		Strip Twist	Plant fiber core color	Core Twist/yarns		Ag:Au ratio (wt%)	Membrane species	Assumed Binder/Adhesive
Spain	12th-13th c	6372	S	Ecru	S/2-ply	30–35	*n.a*	Cattle	
	13th c	1902-1-240_1	S	White	S/2-ply	*n.a*	*n.a*	Cattle	
		1902-1-240_2				15–25	14:1	*n.a*	
		1902-1-241a_2	S	Ecru	S/2-ply	10–20	14:1	*n.a*	
		1902-1-241a_3				*n.a*	*n.a*	Cattle	
		1902-1-253a_1	S	Ecru	S/2-ply	*n.a*	3:1	Cattle	
		1902-1-253a_2				15–25	*n.a*	Cattle	Not-protein based
Italy	13th c	1902-1-227_1	S/ S and Z	Ecru	S/2-ply	10–15	*n.a*	Cattle	
		1902-1-227_2				*n.a*	36:1	*n.a*	
	14th c	1902-1-250_1	S	Ecru	S/2-ply	*n.a*	*n.a*	Cattle	
		1902-1-250_2				10–15	14.5:1	Cattle	
		1902-1-257b_1	S	Ecru	S/2-ply	*n.a*	15.5:1	Cattle	
		1902-1-257d_2	S	Ecru	S/2-ply	*n.a*	10.5:1	Cattle	
		1902-1-257d_3				15–30	*n.a*	Cattle	
		1902-1-279_1	S	Ecru	S/2-ply	10–20	4:1	Cattle	
		1902-1-279_2				20–30	n.a	Cattle	
		1902-1-329a_1	S	Ecru	S/2-ply	5–15	10:1	Cattle	Sheep hide glue*
		1902-1-329a_2				5–10	10:1	Cattle	Sheep hide glue*
		1938-84-1_1	S	Ecru	S/2-ply	*n.a*	no Au	Cattle	
		1938-84-1_2				5–15	*n.a*	Cattle	
		81.01.01bis	S	Ecru	S/2-ply	20–45	*n.a.*	Cattle	
		D13e	S	Ecru	S/2-ply	*n.a*	4:1	Cattle	
		D10	S	White	S/2-ply	*n.a*	4:1	Cattle	
		D11	S	Ecru	S/2-ply	*n.a*	8.5:1	Cattle	
		P7i	S	Ecru	S/2-ply	*n.a*	4:1	Cattle	
		P7k	S	Ecru	S/2-ply	*n.a*	6:1	Cattle	
		P10	S	Ecru	S/2-ply	*n.a*	no Au	Cattle	
		P11	S	Ecru	S/ 2-ply	*n.a*	5:1	Cattle	
		II161	S	White	S/2-ply	30–35	*n.a*	Cattle	
	14th-15th c	1902-1-274a	S	Ecru	S/2-ply	20–40	4:1	Cattle	Sheep hide glue*
		1902-1-274b	S	Ecru	S/2-ply	5–10	3:1	Cattle	Sheep collagen glue
		5798	S	Ecru	S/2-ply	10–20 (inner) 20–65 (outer)	*n.a*	Cattle	
	15th c	P7l	S	Ecru	S/2-ply	*n.a*	1.5:1	Cattle	
		P9	S	Ecru	S/2-ply	*n.a*	8:1	Cattle	
	16th c	P12	S	Ecru	S/2-ply	*n.a*	6.5:1	Cattle	Sheep collagen glue
Italy or Flanders	13th-14th c	3RU8457_1	S	White	S/2-ply	10–15	2:1	Cattle	
		3RU8457_2	S	White	S/2-ply	10–15	4.5:1	Cattle	
Germany	14th c	P1	S	Ecru	S/2-ply	*n.a*	3:1	Cattle	
Middle East (Iran or Iraq)	13th-14th c	5963_1	S	Ecru	S/2-ply	*n.a*	*n.a*	Cattle	
		5963_2				5–10	*n.a*	*n.a*	

n.a = not analyzed. * Identified as hide glue based on the presence of collagen type III markers (see **SI_Collagen Identification in Membranes).** The analytical notation of the fibrous core indicates the twist of the core, and the number of yarns that make it up. The spin direction of individual yarns was not always clearly identified, and thus not reported in the results.

**Figure 3:**
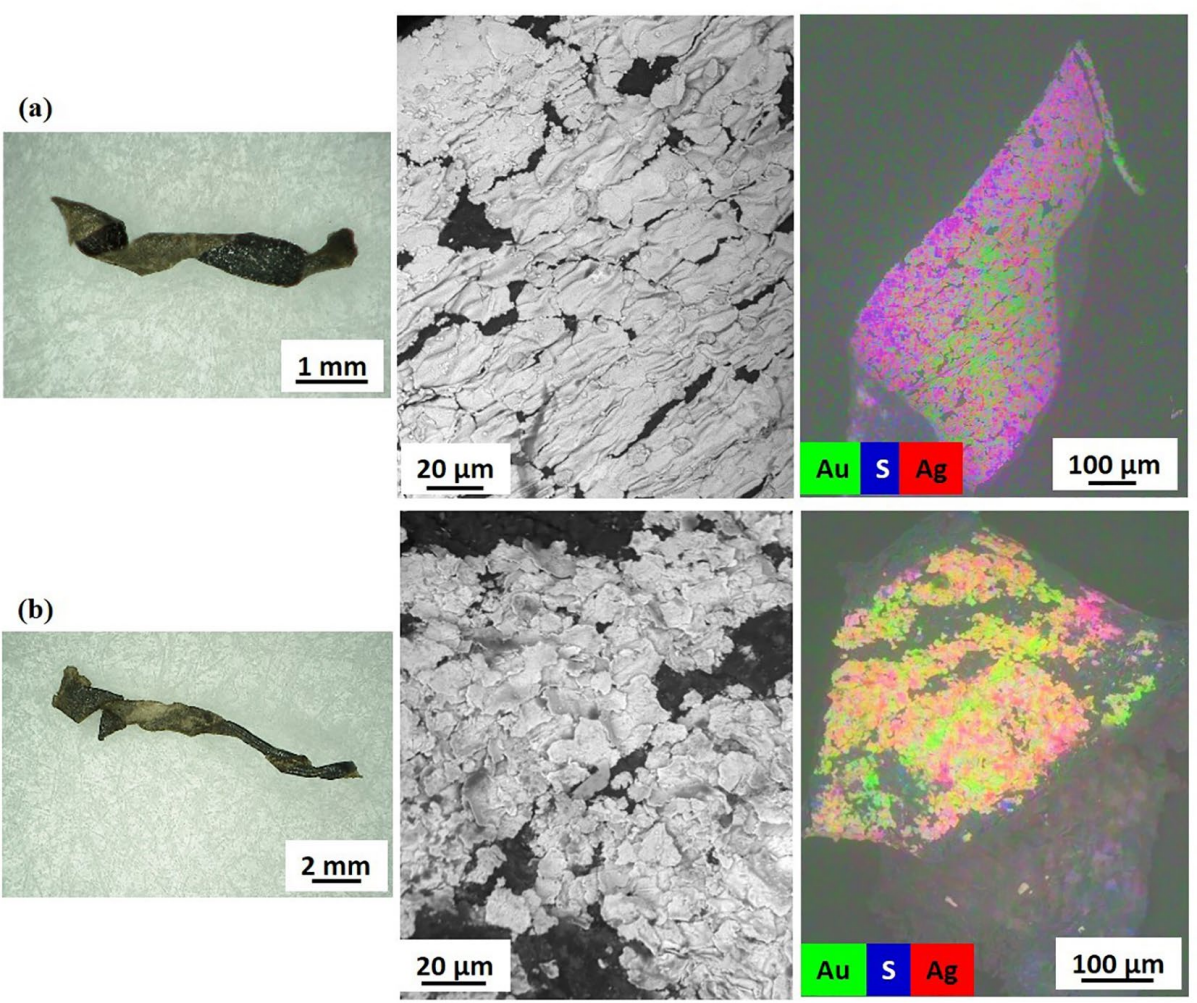
Metal coatings on membrane-based metal threads. (**a**) Sample 1902-1-240_2, from left to right: HIROX image of the sample investigated (scale bar 1 mm); SEM-BSE image (scale bar 20 µm), metal leaf coating; EDS surface mapping image (15 kV, scale bar 100 µm), composite map showing the distribution of gold (Au) in green, and silver (Ag) in red, in two different layers, with a gold layer partly preserved on top of the silver one, sulphur (S) in blue is associated to silver corrosion; (**b**) Sample 1902-1-257d_2, from left to right: HIROX image of the sample investigated (scale bar 2 mm); SEM-BSE image (scale bar 20 µm), leaves resembling powder flakes (possibly a smaller size of leaves); EDS surface mapping image (15 kV, scale bar 100 µm), composite map showing the distribution of gold (Au) in green, silver (Ag) in red, with a gold layer partly preserved on top of the silver one, sulphur (S) in blue is associated to silver corrosion. HIROX images by Cristina Scibè © Museum Conservation Institute, Smithsonian Institution; SEM micrographs by Thomas Lam © Museum Conservation Institute, Smithsonian Institution.

In spite of the metal coatings appearing overall largely tarnished and delaminated in almost all the threads, a bi-layered metal leaf coating was identified by the non-homogeneous distribution of gold and silver elements on the coating surface (examples shown in Fig. 3), hinting at the use of the so-called *Zwischgold* or *part-gold*^47,48^. Thus, assuming that the coatings consist in a gold layer on top of a silver one, a semi-quantitative analysis was attempted and silver/gold weight ratios calculated (Table 2, details are reported in Supplementary SI-4.B). The Ag/Au ratios were found to be highly dependent on the conservation state of the gold layer, indeed they ranged from 2:1, in sample 3RU8457_1, showing a well-preserved gilding, to 36:1 in sample 1902-1-227_2, where the metal coating was largely worn. In samples 1938-84-1_1 and P10, having an almost completely worn coating, gold was not even detected. Copper was found as minor or trace element in eight samples, seven of which were investigated by SEM-µXRF. The presence of mercury was noticeably detected in four out of eight samples analyzed by SEM-µXRF, possibly suggesting a fire-gilding technique, which involves dissolving gold in hot mercury to form a mixture, gold amalgam, which is then spread over a base metal surface, and subsequently heated to let the mercury evaporates, leaving behind a firmly bonded layer of gold^22,49–51^. Other trace elements detected were zinc, lead, titanium, and nickel.

The ubiquitous presence of sulphur (in concentrations from 0.73 wt% to 21.63 wt%), and sometimes chlorine (in concentrations of less than 1 wt% and up to 17.24 wt%), in correspondence of the metal layers, were attributed to degradation products (Ag_2_S and AgCl) caused by the reaction with silver. Silver sulphides are indeed the main cause for the characteristic blackening of the coatings, while chlorides are typical of archaeological soils or as residual of inappropriate cleaning methods of textiles^30^.

**Figure 4:**
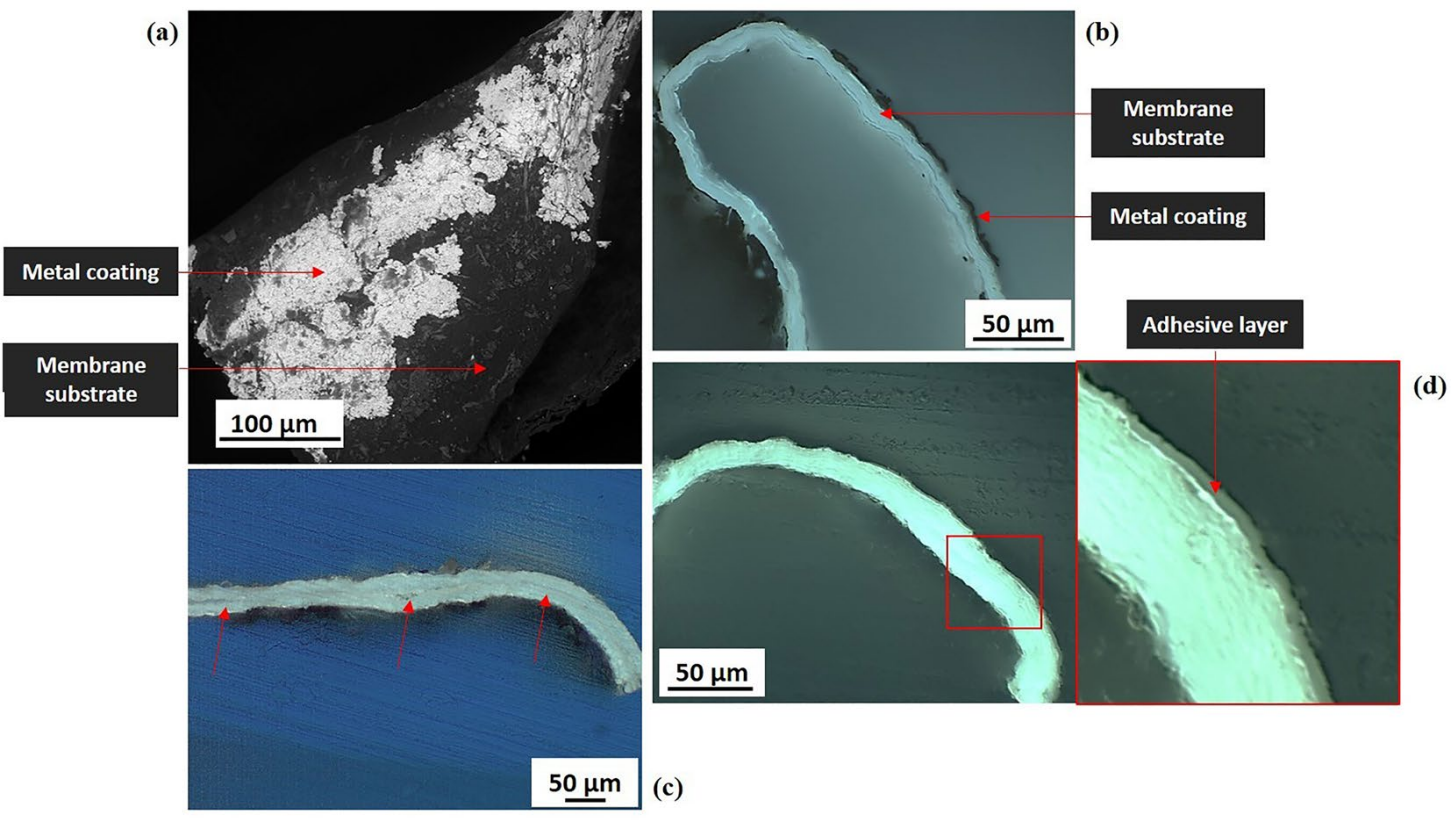
Stratigraphy of membrane-based metal strips. (**a**) Sample 1902-1-257b_1: SEM-BSE image (scale bar 100 µm), metal coating on membranous strip, no evidence of an intermediate adhesive ground; (**b**) Sample 1902-1-250_2: fluorescence cross-sectional image (OM2, UV reflected light, scale bar 50 µm), overall strip thickness of 10–15 µm, two clearly distinguishable layers, namely metal coating (black layer) and membranous substrate (white-bluish fluorescence); (**c**) Sample 81.01.01bis: fluorescence cross-sectional image (OM1, UV reflected light, scale bar 50 µm), overall strip thickness of 20–45 µm, the red arrows show two clearly distinguishable membranous layers coupled together; (**d**) Sample 1902-1-253a_2: (Left) fluorescence cross-sectional image (OM2, UV reflected light, scale bar 50 µm), overall strip thickness of 15–25 µm; (Right) detail of an area of the strip in cross-section, three clearly distinguishable layers, namely metal coating (black layer), possibly an adhesive layer (milky-grey fluorescence), and the membranous substrate (white-bluish fluorescence). SEM micrograph by Thomas Lam © Museum Conservation Institute, Smithsonian Institution; OM micrographs (**b**) and (**d**) by Cristina Scibè © Museum Conservation Institute, Smithsonian Institution; OM micrograph (**c**) by Cristina Scibè © Opificio delle Pietre Dure.

The strip’s cross-section, examined by fluorescence microscopy for 20 samples, appeared to have a simple stratigraphy with only two clearly distinguishable layers corresponding to the metal coating and the membrane substrate, also appreciable by SEM-BSE surface analysis **(**Fig. 4a**)**. The strip substrate, characterized by a bright white-bluish fluorescence, with a smooth texture, and an ordered structure of aligned longitudinal strata resembling tissue veining was identified as an animal membranous tissue, untanned and undyed (Fig. 4b–d). Exploring any similarity with animal histological samples, analogies could be found with tissues from gastrointestinal organs (details in Supplementary SI-2.B). In sample 81.01.01bis (Fig. 4c), the presence of two membranous layers, resembling two tissues joined together, led to a strip 20–45 µm thick, one of the thickest observed in membrane thread. Only in sample 1902–1-253a_2 (Fig. 4d**)**, a layer with a milky-grey fluorescence was clearly identified between the metal coating and the substrate. The material’s behavior to UV (ultraviolet light) excitation might indicate a gum or natural resin^52,53^. The overall thickness of the membranous strips was measured with average values ranging from 5 to 65 µm (Table 2). Details of the range of measured width and thickness values of the strips are shown respectively in Supplementary SI-2.A and SI-2.C.

The proteins identified by proteomics were consistent with animal membrane proteins observed in a pilot study^46,54^ (i.e. a range of collagen proteins, smooth muscle proteins and other intestinal membrane proteins from cattle gut), albeit in lower number than in mock-up samples of cow membranes (Supplementary SI-3.A). No adhesive proteins from milk, egg or plant were identified. The predominant proteins (highest number of peptides-to-spectrum matches PSM) were the collagen type I chains alpha-1 and alpha-2 (COL1A1 and COL1A2) followed by collagen type III alpha-1 chain (COL3A1); other collagen chains from type II, IV, V, VI were also identified in the ancient membranes, usually with lower coverage than in the references when present. All 36 membrane samples were matched to a *Bos* species based on species identification of available COL1A1, COL1A2 and COL3A1 sequences, more precisely the domestic cattle *B. taurus*, or, unlikely in this context, the zebu *B. indicus*. The samples notably had the COL1A2 peptide GIPGPVGAAGATGAR (not in *B. mutus*), and the COL3A1 peptides GAAGPPGPPGSAGTPGLQGMPGER and GAPGPQGPPGAPGPLGIAGLTGAR (not in *B. javanicus*). The COL1A1, COL1A2 and COL3A1 peptide markers that allow differentiation from the domestic sheep (*Ovis aries*) and goat (*Capra hircus*), are given in Supplementary SI_Collagen Identification in Membranes. Of these 26 peptides, an average of 24 were found; only sample 1938–84-1_2 had a low count of markers with 14 identified, possibly indicating a particularly degraded sample (see Supplementary SI-2.B) as sample 1938–84-1_1 from the same textile was similar to all other samples. In addition to *Bos taurus*, five samples (belonging to four textiles, see Table 2) had ovicaprid collagen markers including the peptide AGEVGPPGPPGPAGEK, present in sheep but not in goat (Supplementary SI_Collagen Identification in Membranes). To verify that the membrane species was indeed from cattle, further species markers were sought in other proteins. Because smooth muscle proteins are not specific enough (identical sequences between sheep and cattle), markers were found in other minor collagen chain proteins, i.e. COL5A2, COL6A1 and COL6A3, that are component of the membranous tissue. A number of *Bos taurus* markers were matched in all five samples but no *Ovis aries* markers (Supplementary SI-3.A), supporting the identification of the membrane as cattle. The sheep collagen, represented mainly by COL1A1, COL1A2 and COL3A1, is indicative of the possible use of animal glue (identified as hide glue based on the presence of collagen type III markers), although more evidence is needed. In contrast, in sample 1902-1-253a_2 where a possible adhesive layer was detected, no collagen from another species was identified, indicating the adhesive was either cattle (in which case it cannot be differentiated from the membrane) or not protein-based. The percentages of asparagine N and glutamine Q with deamidation were calculated for the three main chains of *Bos taurus* collagen and are shown in Supplementary SI-3.C. Deamidation of the textile membranes was higher than for the references but no clear increase of deamidation with age of samples was observed.

The top proteins identified after collagen type I and III, in both reference and ancient membranes, were proteins belonging or highly expressed in smooth muscles. The other membrane proteins were grouped as extracellular matrix proteins, actin-binding proteins, blood/plasma, enzymes and other cytoskeletal and intracellular proteins; the frequency of identification of proteins in each family is shown in Supplementary SI-3.A.

The smooth muscle proteins are contractile proteins found in the muscles that line hollow organs of the body, including the stomach, intestines, urinary bladder, and uterus, as well as the walls of passageways such as arteries and veins^55^. In the references, filamin-A, actins, desmin, and myosin heavy chain 11, all major proteins of the smooth muscles, were among the top matches. Desmin is unspecific as it is found in the intermediate filaments of cardiac muscle, skeletal muscle, and smooth muscle. The actin protein ACTG2 (Actin, gamma-enteric smooth muscle, found in smooth muscle cells of the urinary and intestinal tracts), was identified in the reference samples with two unique peptides (E_3_EETTALVCDNGSGLCK_19_ and W_357_ISKPEYDEAGPSIVHR_373_) allowing differentiation from the skeletal muscles (ACTA1), aortic smooth muscle (ACTA2), and cardiac muscle (ACTC1). The two peptides (in N- and C-terminus respectively) were however not found in the ancient membranes with the exception of P9 (identification of WISKPEYDEAGPSIVHR, Supplementary SI-3.A). However, the identification of myosin heavy chain 11, a protein found in smooth muscle only, confirms that an organ from the gastro-intestinal tract such as stomach, esophagus, intestine or urinary bladder is the source of the membrane.

### Skin-based metal threads

Skin-based metal threads used as flat strips (Fig. 2d) were from four objects of East/Central Asian origin, while strips wrapped around a fiber core (Fig. 2e–f) were from 26 objects, of Hispano-Islamic, Middle Eastern and Italian provenance.

In composite threads, strips wrapped in Z-direction were found to be combined exclusively with a Z-plied single yarn of silk fibers (Fig. 2e). To this group were found to belong all the Hispano-Islamic objects, the two velvet fragments with same pattern, one of Iranian (possibly Tabriz) the other of Italian (possibly Florence) manufacture (1902-1-385 and I A7 respectively) and a Middle Eastern (Iran or Iraq) fragment (D12b). Conversely, S-twisted strips (Fig. 2f) were found to be combined with a S-plied core of either silk or plant fibers (most likely linen), and exclusively on Middle Eastern and Italian objects (details in Table 3). Similar features were observed by Wardwell and grouped under her category VIII of textiles claiming a Middle Eastern origin (possibly Western Iran) but influenced by Italian silk designs^14^. Wrapped strips, with a width ranging from 210 to 700 µm, were generally narrower than flat strips, which width ranged from 340 to 950 µm, most likely to simplify the winding process around the fibrous core.

**Table 3:** Skin-based metal threads analytical results.

Museum’s assignment		Sample	Wrapped strip			Strip thickness (µm)	Metal coating		Strip Substrate		General attribution based on technical and analytical results
Manufacture	Date		Strip Twist	Fiber core	Core twist/yarns		Medium	Group	Skin species	Assumed Binder/ Adhesive	
Spain	11th-12th c	1965-33-2_1	Z	Yellow silk	Z/single-ply	*n.a*	*n.a*	*n.a*	Goat		Hispano-Islamic manufacture: Goat leather strip, Z wound around a Z/single-ply silk core Main adhesive: egg white
		1965-33-2_2				*n.a*	Leaf	II.2.b	Goat		
	12th-13th c	313	Z	Tan silk	Z/single-ply	10–20	*n.a*	*n.a*	Goat		
		1902-1-216	Z	Tan silk	Z/single-ply	*n.a*	*n.a*	*n.a*	Goat		
		1965-33-5_1	Z	Yellow-tan silk	Z/single-ply	35–45	Leaf	II.1.c	Goat	Egg white	
		1965-33-5_2				*n.a*	*n.a*	*n.a*	Goat	Egg white	
possibly Spain		5968	Z	Yellow silk	Z/single-ply	*n.a*	Powder	*n.a*	Goat	Egg white + Cattle hide glue*	
		6369b	Z	Yellow-pink silk	Z/single-ply	*n.a*	Leaf	*n.a*	Goat	Egg white	
Spain	13th c	IA5bis_1	Z	Tan silk	Z/single-ply	*n.a*	*n.a*	*n.a*	Goat	Egg white	
		1902-1-229b_1	Z	Tan silk	Z/single-ply	*n.a*	*n.a*	*n.a*	Goat	Egg white	
		1902-1-229b_2				40–55	Leaf	I	Goat	Egg white	
		1902-1-977c_3	Z	Tan silk	Z/single-ply	35–40	Leaf	II.2.b	Goat	Egg white	
		1938-78-1_2	Z	Yellow silk	Z/single-ply	*n.a*	Leaf	II.2.c	Goat	Egg white	
		1943-20-1b_2	Z	Yellow silk	Z/single-ply	30–35	Leaf	II.2.c	Goat	Egg white	
		5776_1	Z	Yellow silk	Z/single-ply	*n.a*	Leaf	*n.a*	Goat	Egg white + Sheep hide glue*	
	14th c	1902-1-310_1	Z	Yellow silk	Z/single-ply	95.-150	Powder	*n.a*	Goat	Hide glue sheep + cattle*	
		1902-1-310_2				overall: 100–125 leather only: 55–95	Powder	III	Goat	Cattle collagen glue	
		1902-1-311_1	Z	Yellow silk	Z/single-ply	40–60	Leaf	III	Goat	Cattle collagen glue	
		1902-1-311_2				*n.a*	Leaf	*n.a*	Goat	Egg white + Sheep/Cattle collagen glue	
Italy (possibly Florence)	14th–15th c	IA7_2	Z	Yellow silk	Z/single-ply	25–40	*n.a*	*n.a*	*n.a*	*n.a*	Italian or Iranian (possibly Tabriz) manufacture: Sheep leather strip, Z wound around a Z/single-ply silk core Main adhesive: sturgeon glue
		IA7_3				*n.a*	*n.a*	*n.a*	Sheep	Sturgeon glue	
Iran (possibly Tabriz)	Possibly 13th c	1902-1-385_2	Z	Yellow silk	Z/single-ply	70–105	Leaf	II.1.a	Sheep	Sturgeon glue	
Persia	around 14th c	D12b	Z	Yellow silk	Z/single-ply	35–45	Leaf	II.1.b	Sheep	Sturgeon glue	
		D12b (back)							Sheep	Sturgeon glue + Horse hide glue*	
Iran or Iraq	14th c	03.02.02_2	S	Ecru linen	S/3-ply	65–70	*n.a*	*n.a*	*n.a*	*n.a*	Italian or Middle Eastern (Persia: Iran or Iraq) manufacture: Sheep leather strip, S wound around a S/2-ply or 3-ply linen core (2-ply silk core in one case) Main adhesive: sturgeon glue
		03.02.02_3				*n.a*	*n.a*	*n.a*	Sheep	Sturgeon glue	
	Possibly 14th c	164	S	Ecru linen	S/3-ply	70–90	*n.a*	*n.a*	Sheep	Sturgeon glue	
Italy	14th c	1902-1-262_1	S	Ecru linen	S/2-ply	40–60	*n.a*	*n.a*	Sheep	Sturgeon glue + Cattle hide glue*	
		1902-1-262_2				*n.a*	Leaf	II.1.b	Sheep	Sturgeon glue	
		1902-1-262_3				*n.a*	*n.a*	*n.a*	Sheep	Sturgeon glue	
		1902-1-271a_1	S	Ecru linen	S/3-ply	*n.a*	Leaf	II.1.b	*n.a*	*n.a*	
		1902-1-271a_2				*n.a*	*n.a*	*n.a*	Sheep	Sturgeon glue	
		1902-1-271a_3				95–120	*n.a*	*n.a*	Sheep	Sturgeon glue	
		1902-1-272_1	S	Ecru linen	S/3-ply	25–45	*n.a*	*n.a*	Sheep	Sturgeon glue	
		1902-1-272_2				*n.a*	Leaf	II.1.b	*n.a*	*n.a*	
		1902-1-273a	S	Yellow silk	S/2-ply	*n.a*	Leaf	II.1.a	Sheep	Sturgeon glue	
		1902-1-273b	S	Yellow silk	S/2-ply	80–130	*n.a*	*n.a*	Sheep	Sturgeon glue	
		1902-1-292a	S	Ecru linen	S/3-ply	65–75	Leaf	II.1.b	Sheep	Sturgeon glue	
		1902-1-292b	S	Ecru linen	S/3-ply	95–110	Leaf	II.1.b	Sheep	Sturgeon glue	
	14th–15th c	1902-1-285_1 (gold)	S	Ecru linen	S/3-ply	90–130	Leaf	II.1.b	Sheep	Sturgeon glue	
		1902-1-285_2 (gold)				*n.a*	*n.a*	*n.a*	Sheep	Sturgeon glue	
possibly Spain	15th c	1902-1-251_1	S	Ecru linen	S/2-ply	40–50	Leaf	II.1.b	*n.a*	*n.a*	
		1902-1-251_2				*n.a*	*n.a*	*n.a*	Sheep	Sturgeon glue	
Far East	13th c	D13a	Flat Strip			70–100	Leaf	II.1.a	Sheep	Sturgeon glue	Mongol-ruled territories (Central Asia, Mongolia, China) manufacture: Sheep leather strip, flat strip Main adhesive: sturgeon glue
China		P4c	Flat Strip			70–85	Leaf	II.1.a	Sheep	Sturgeon glue	
		P4d	Flat Strip			110–130	Leaf	II.1.b	Sheep	Sturgeon glue	
Spain or Iran	14th c	1902-1-233	Flat Strip			85–100	Leaf	II.3	Sheep	Sturgeon glue	
Central Asia/Northern China		1862:16 I	Flat Strip			165–210	Leaf	II.1.a	Sheep	Sturgeon glue + Cattle hide glue*	
		1862:16 II	Flat Strip			*n.a*	Leaf	II.1.b	Sheep	Sturgeon glue	
		1862:16 III (front)	Flat Strip			105–150	Leaf	II.1.c	Sheep	Sturgeon glue + Horse hide glue*	
		1862:16 III (back)					Leaf	III			
		1862:16 IV (front)	Flat Strip			*n.a*	Leaf	II.2.a	Sheep	Sturgeon glue + Horse hide glue*	
		1862:16 IV (back)					Leaf	III			
		1862:16 V	Flat Strip			265–290	Leaf	I	Sheep	Sturgeon glue + Wheat	

n.a = not analyzed. * Identified as hide glue based on the presence of collagen type III markers (see **SI_Proteomics Identification in Skins**). Metal coating groups correspond to: I (silver), II (gold) and III (gilt-silver). Group II’s sub-groups are defined according to the gold content in weight percentage (wt%) and purity in carats (k): sub-group II.1a-c (almost pure gold) from 23.94 k to 23.16 k, sub-group II.2a-c (gold-silver alloy) from 22.66 k to 19.74 k), and sub-group II.3 (gold-silver-copper alloy) at 17.43 k. The analytical notation of the fibrous core indicates the twist of the core, and the number of yarns that make it up. The spin direction of individual yarns was not always clearly identified, and thus not reported in the results.

In most of the strips, either wrapped or flat, the metal coating was found to be leaf-made, thus, made by thin leaves obtained by hammering gold bars or blocks, with the exception of two Hispano-Islamic textiles showing a powder coating of the strips (Table 3 and Supplementary SI-4.C). Tool marks left by the burnishing process were observed either in leaf and powder coatings by SEM-BSE analysis. Flat strips from fabrics III and IV of the dalmatic 1862:16 showed a double-sided coating (gold on the front side and gilt-silver on the back side of the strip), whereas all the other strips were metal-coated just on one side. A yellow-orange coat layer of organic nature resembling a lacquer was identified on top of the silver coating by SEM–EDS on sample 1862:16 V, possibly as a gilding imitation; thus far, one similar example has been described in the literature, identified in a fourteenth century Central-Asian weaving^1^. Details of the visual and technical characterization of wrapped and flat skin-based strips are shown in Supplementary SI-2.A.

**Figure 5:**
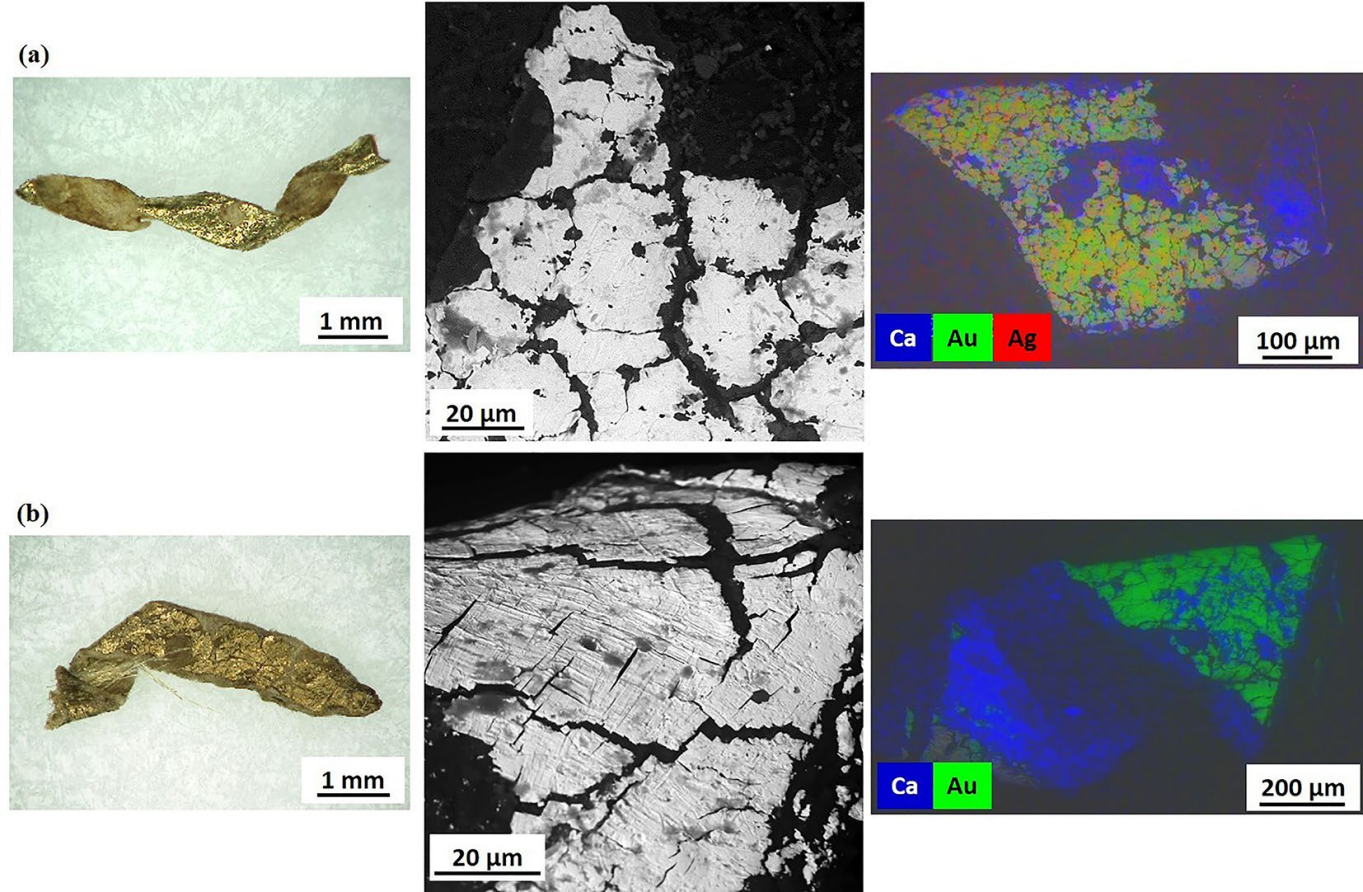
Metal coatings on skin-based metal threads. (**a**) Sample 1938-78-1_2, from left to right: HIROX image of the sample investigated (scale bar 1 mm); SEM-BSE image (scale bar 20 µm), metal leaf coating showing a net of cracks (“craquelure”); EDS surface mapping image (15 kV, scale bar 100 µm), composite map showing the homogeneous distribution of gold (Au) in green, and silver (Ag) in red on the metal surface, while calcium (Ca) in blue appeared distributed mostly on the underneath ground and the inner surface of the skin-based strip. Gold-silver alloy coating (group II.2.c); (**b**) Sample 1902–1-385_2, from left to right: HIROX image of the sample investigated (scale bar 1 mm); SEM-BSE image (scale bar 20 µm), metal leaf coating showing a net of cracks (“craquelure”) and burnishing marks; EDS surface mapping image (15 kV, scale bar 200 µm), composite map showing the distribution of solely gold (Au) in green on the metal surface, and calcium (Ca) in blue on the underneath ground and the inner surface of the skin-based strip. Almost pure gold coating (group II.1.a). HIROX images by Cristina Scibè © Museum Conservation Institute, Smithsonian Institution; SEM micrographs by Thomas Lam © Museum Conservation Institute, Smithsonian Institution.

On skin strips, EDS surface elemental mapping identified mostly single-layer coatings (examples reported in Fig. 5), with the exception of sample 1902-1-311_1, where a bi-layered coating made of gilt-silver leaves was identified. In the two flat strips coated on the back side with gilt-silver, gold was only present sporadically; it was indeed detected by SEM–EDS surface analysis only in sample 1862:16 III in concentration lower than 1 wt%. According to the EDS semi-quantitative analysis, three main compositional groups were identified: silver coatings (group I), gold coatings (group II) and gilt-silver coatings (group III). Most of the samples were found to belong to group II, within which 7 sub-groups were further defined according to the gold content in weight percentage (wt%) and purity in carats (k), namely: almost pure gold coatings (sub-group II.1a-c, from 23.94 k to 23.16 k), gold-silver alloy coatings (sub-group II.2a-c, from 22.66 k to 19.74 k), and gold-silver-copper alloy coatings (sub-group II.3, 17.43 k) (Table 3 and Supplementary SI-4.C).

Flat strips as well as Hispano-Islamic wrapped strips showed a broader variety of coating compositions. In contrast, all the wrapped strips belonging to Middle Eastern and Italian objects showed a quite homogeneous composition, with coatings of almost pure gold. Within the gold group, Hispano-Islamic samples had coatings of lower quality. Interestingly, gilt-silver coatings were identified only in threads belonging to fourteenth century objects (Central Asian or Chinese and Hispano-Islamic), testifying of the tendency to use less precious metals in later periods (see Supplementary SI-4.C).

In nine wrapped-strip samples investigated by SEM-µXRF, copper was as expected the most common trace element detected, indicating a cupellation refining process of the metal sources, or the use of already refined metal artefacts. Whereas the higher amount detected in sample 1902-1-233 (3.58 wt%) hints to an intentional addition to enhance the hardness of the gold-silver alloy, or give a reddish shade to the coating. Other minor and trace elements detected were iron, zinc, nickel, titanium, and lead. Mercury traces were detected on one sample with an almost pure gold coating (1902-1-292b), suggesting an amalgamation process used for gold extraction, or a gold refining method using mercury, both were attested to be largely used before the fourteenth century in the Middle East only^22^. However, mercury was not found on the sample belonging to another fragment of the same object (1902-1-292a), nor on gilt-silver coatings. Trace elements detected by SEM-µXRF in selected samples are informative of the original gold composition. Nevertheless, assigning them to the original gold/silver source in order to establish the metal provenance is challenging, since the process of extraction, refining, re-melting (and such also mixing gold from different sources) and re-use of metals complicate the identification of the ores supplies^56^.

**Figure 6:**
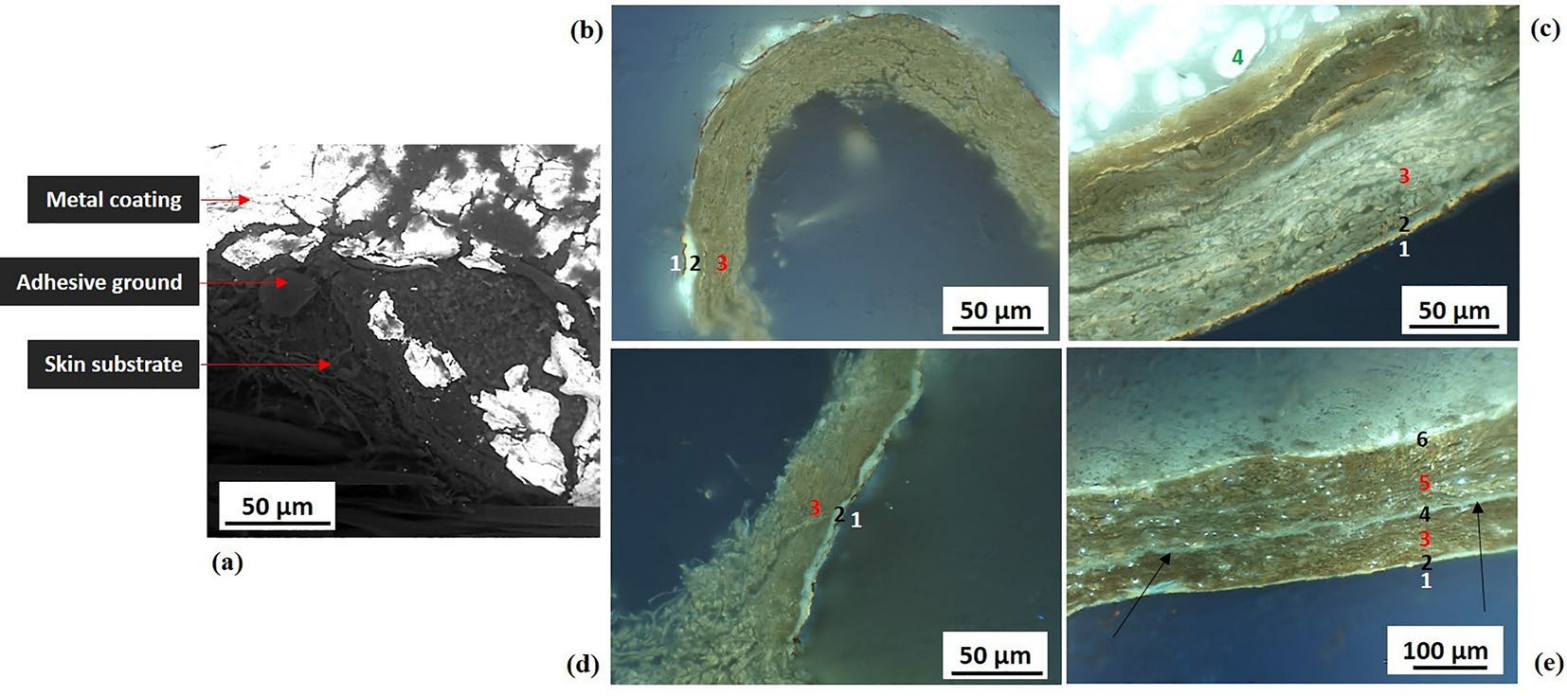
Stratigraphy of skin-based metal strips. (**a**) Sample 1902–1-385_2: SEM-BSE image (scale bar 50 µm), metal coating on a skin-based strip with an intermediate adhesive ground; (**b**) Sample 1965–33-5_1: fluorescence cross-sectional image (OM2, UV reflected light, scale bar 50 µm), overall strip thickness of 35–45 µm; three layers identified: (1) metal coating (black layer), (2) adhesive (bright milky-white fluorescence), (3) tanned leather substrate (brown color); (**c**) Sample 1902–1-271a_3: fluorescence cross-sectional image (OM2, UV reflected light, scale bar 50 µm), overall strip thickness of 95–120 µm; four layers identified: (1) metal coating (black layer), (2) adhesive (milky-grey fluorescence), (3) tanned leather substrate (brown-greyish color), (4) fibrous core, linen fibers (white fluorescence); d) Sample 1902-1-272_1: fluorescence cross-sectional image (OM2, UV reflected light, scale bar 50 µm), overall strip thickness of 25–45 µm, three layers identified: (1) metal coating (black layer), (2) adhesive (bright milky-white fluorescence), (3) tanned leather substrate (brown color); e) Sample 1862:16 I: fluorescence cross-sectional image (OM2, UV reflected light, scale bar 100 µm), overall strip thickness of 165–210 µm; six layers identified: (1) metal coating (black layer), (2, 4, 6) adhesives (from bright milky-white to milky-grey fluorescence), (3, 5) tanned leather substrates (brown color); the black arrows show the presence of an adhesive layer (4) between two leather strips (3 and 5). SEM micrograph by Thomas Lam © Museum Conservation Institute, Smithsonian Institution; OM micrographs by Cristina Scibè © Museum Conservation Institute, Smithsonian Institution.

On 28 samples observed in cross-section by fluorescence microscopy, the skin substrates were identified as tanned leathers, due to the collagen fiber structure, the lack of fluorescence and the brown-greyish/greenish color^57^ (Fig. 6b–e).

Proteomics analysis mainly identified collagens since most other proteins from skin have been eliminated during the cleaning and tanning process (Supplementary SI-3.B shows the protein families identified in the skin threads compared to reference samples of raw hide, parchment, vellum, and leathers). The source species of the skin were determined to be either domestic goat (*Capra hircus*) or sheep (*Ovis aries*), based on the primary collagen species identified COL1A1, COL1A2 and COL3A1, and ovicapra markers (Supplementary SI_Proteomics Identification in Skins). The percentages of asparagine N and glutamine Q with deamidation were calculated for the three main chains of collagen for the skin species and are shown in Supplementary SI-3.C. As with membrane threads, no clear increase of deamidation with age of samples was observed. The average of deamidation for the sheep-skin threads was similar to deamidation of vegetable-tanned leather, and goat-skin threads were on average more deamidated than the sheep’s.

Wrapped strips were characterized by a basic structure of three layers that includes an adhesive layer between the metal coating and the skin substrate (Fig. 6a), with an overall thickness ranging from about 10 to 60 µm in Spanish samples (with the exception of sample 1902–1-310, powder coated, with a skin strip of 55–95 µm thickness in sample 2 and of 95-150 µm thickness in sample 1, Table 3), to 25 to 130 µm in the Middle Eastern and Italian samples. The extreme thinness of the wrapped-strip substrates compared to the known thickness of mammalian skins could be the result of *splitting* the original skins, as largely practiced in parchment making^58^ and *skivers* making, that are thin sheep leathers used for traditional bookbinding (^57^, p.54). Flat strips were found to be thicker and surprisingly very complex with up to 6/7 visible layers (Fig. 6e). Indeed, a second adhesive layer was clearly visible on most cross-sections, applied on the reverse of the strips, even if ungilt, and in samples 1862:16 I, in Fig. 6e, and 1862:16 V an intermediate layer resembling an adhesive ground divided the skin substrate. By consequent, these strips were the thickest ones (and even the widest ones), at respectively 165–210 µm and 265–290 µm (Table 3). In all the strips examined in cross-section, the adhesive layers, when detected, were found to show a similar UV fluorescence (with slight variances in brightness and hue) that is characteristic of protein-based adhesives, such as the ones containing milk, egg or animal glue (for the layering of either wrapped and flat skin strips, see Supplementary SI-2.B; for the range of width and thickness values of the strips see respectively Supplementary SI-1.A and SI-2.C).

Wrapped threads made of goat skin were all of Hispano-Islamic origin, and for a majority of them, an egg white adhesive was identified (Tables 3 and Supplementary SI-3.B). In the earlier samples 1902-1-216, 1965-33-2, and 313, which showed an overall bad state of conservation, egg proteins were not detected. In the later samples (fourteenth century), egg white was also not detected with the exception of two ovalbumin peptides in 1902-1-311_2. Instead, mammalian collagen was identified in samples from 1902-1-310 to 1902-1-311, indicative of collagen glue. It is noteworthy, that in both objects, samples taken from different locations had different adhesive composition. This could be from the original composition but also possibly a sampling effect due to an uneven application or conservation of the adhesive. Samples 5776_1 and 5968 also had sheep and cattle collagen respectively, in addition to egg white, suggesting a mixture of binders.

Sheep skin strips, either used wrapped or flat, were invariably found to contain a fish glue adhesive **(**Table 3**)**, identified as coming from a sturgeon species from the *Acipenser* genus. There are currently only two Eurasian sturgeon species with collagen sequences represented in databases: *A. ruthenus* (starlet) and *A. schrenckii* (Amur sturgeon). To complement the few collagen sequences, peptides identified through de novo sequencing were added to an in-house database. Due to the complex nature of the sturgeon genome (e.g. *A. ruthenus* is considered a tetraploid species and contains duplicate genes^59^), it was common to find multiple sequences for the same peptide in a sample. The identification of de novo markers in all samples also seem to indicate that a species other than *A. ruthenus* was present. Three *A. schrenckii* sequences were identified only in samples 1902-1-233, 1902-1-292a and 1862:16 (II, III and IV), while one COL1A2 sequence, DGQPGHPGPIGPAGSR, was found only in all 1862:16 samples, and with low score in D13a**,** possibly indicating a sturgeon glue from a species not used in the other samples.

For most metal-wrapped threads the data did not indicate the presence of collagen from another species, however the presence of glue from sheep would be indiscernible from the proteins coming from the skins. In one case, a strong cattle collagen signal was found in sample 1 but not in samples 2 and 3 tested in two other locations from the textile 1902-1-262, most likely as a contamination from conservation practices. Generally, no adhesive is visible on the back side of metal-wrapped strips, in contact with the fibrous core. But in sample D12b, a remarkable adhesive migration into the skin substrate was observed on fluorescence cross-sectional images, as in a few flat strip samples. In a fragment of sample D12b, carefully scrapped on the back side of the strip, specific *Equus* peptides were detected in addition to sturgeon collagen, including one peptide specific to horse (*E. caballus*). In flat strips, cattle (*B. taurus*) collagen was found in sample 1862:16 I and horse (*E. caballus*) collagen was found in the two double-sided coated samples, 1862:16 III and 1862:16 IV, when analyzing the whole strip. Sample 1862:16 V, with a yellow top coating, contained the usual fish glue adhesive (without the addition of hide glue), as well as a plant binding material identified by the presence of barley and wheat peptides from proteins such as B3-hordein and alpha and beta-amylase. Such proteins were not found in any other samples tested but the cross-section does not allow for the conclusion of whether the plant binding is part of the adhesive layers or the top-coat layer, since the latter was not well preserved in the fragment embedded in resin (see Supplementary SI-2.B).

Milk proteins were identified in four samples, but three out of the four samples shared proteins and peptides with the blank, indicating a likely lab contamination with cow milk**.** Only in D13a were two ovicapra milk peptides from alpha-S1 casein identified, distinct from the blank; as a minor component, the milk might have been deposited on the sample, rather than being part of the adhesive.

Finally, two samples yielded human proteins: sample 1902-1-229b_1 (but not 1902-1-229b_2 sampled at a different location of the textile), and sample P4d. Both samples have blood proteins (serum albumin, hemoglobin, immunoglobulins and apolipoprotein) but sample 1902-1-229b_1 was characterized by the presence of proteins expressed in the epidermis, possibly from a skin lesion or injury, while sample P4d was characterized by a range of proteins found in relation to immunological response and blood coagulation, in particular positive acute-phase proteins. In addition, a few fungi proteins were identified in P4d (with matches to *Aspergillus* sp.), not present in any other samples; hints of a biological contamination were found by the BSE imaging of the metal surface of the sample. The association of fungi proteins with proteins expressed by the immune system might indicate a response to a fungal infection, such as aspergillosis, an infection caused by spores from *Aspergillus*, a common mold^60,61^. Such infection can lead to coughing blood^61^.

Details of the identification of proteins and species markers are shown in Supplementary SI_Proteomics Identification in Skins and SI-3.B for all skin, adhesive and contaminating proteins.

## Discussion

Based on the multi-analytical investigation on a set of 91 samples, it was possible to define some technical features, identify materials used (metal, strip and protein-based adhesives), and better understand the assembling methods of animal-based metal threads. Consistent analogies were found in metal threads from textiles of the same provenance, and as a consequence, the identified patterns of technology could be attributed to specific geographical areas. Conversely, in a few cases, the skin-based thread features led to assign them to a different manufacture than the one given by museums to the corresponding textile, as defined by stylistic and weave type analysis (see Table 3).

All membrane threads were found to be made of strips from cattle tissue coated with gilt-silver leaves, S-twisted around a 2-ply core of plant fibers (possibly linen). Besides one possible Middle Eastern object, they were found mostly in textiles of Italian production, as well as in a few Spanish and one German textiles, attesting of their preferred use in European workshops. The different thickness, and sometimes morphology, of the membranes measured in cross-section, might suggest that different parts of the intestinal tract were used to make the strips. The anatomy of the wall, which surrounds the lumen of the gastrointestinal organs, consists of several layers (mucosa, submucosa, muscularis externa or propria, and serosa), which differ in composition, morphology and thickness from one organ to another according to its specific function^62^. Most likely the two middle layers were used to make the membrane substrate of the threads. The protein identification in the strips is indeed in agreement with the composition of these two layers; the submucosa is composed of connective tissue (mainly collagen type I and III proteins) and smooth muscle proteins, and the muscularis externa is mainly composed of smooth and striated muscles. The use of the submucosa was already suggested at the end of the nineteenth century by two professors of Munich, Dr. von Miller and Dr. Harz, who received medieval samples from Franz Bock for microscopic examination and disagreed with the findings of earlier research that identified the membrane substrates as the peritoneum (the membrane lining the abdominal cavity) of slaughtered cattle^6^.

The use of animal gut membranes is attested in cultural heritage in Europe^63^. Although of later periods (between the sixteenth and eighteenth century), the Italian production of violin’s “cat-strings” made from the muscularis externa layer of the small intestines of sheep was known to be of the highest quality^64^; the function and properties required for the strings are quite similar to those of membrane threads, such as strength and flexibility. Perhaps the closest equivalent to membrane threads is goldbeater’s skin, a gut membrane prepared from the outer or peritoneal coat of cow’s caecum^65^. It holds its name from the production of gold leaves, in which the membranes were placed between sheets of gold to keep them separated during the goldbeating process^63,66,67^. However, in membrane threads, gilt-silver leaves must adhere to the substrate, either through its natural adhesive properties^3^, or through the use of a binder, which might be collagenous in nature (as can be suggested by proteomics), or non-proteinaceous. To make goldbeater’s skin, two sheets of gut “skin” were coupled by binding their muscle fascia layers together^65^, a morphology that seems to match only with sample 81.01.01bis. However, similar membrane layers and preparation techniques might have been used, and membrane threads could have been prepared in goldbeating workshops. For instance, an Italian document of 1273 describes a work agreement between the owner of a goldbeating workshop and two workers, husband and wife, who were dedicated to the beating of gold leaf for the former, and the washing of membranes and the spinning of gold (around a fibrous core) for the latter^68,69^. While these membranes were undoubtedly used for goldbeater’s skins, it cannot be excluded that they were also used for making membrane-based metal threads and assembled into metal-wrapped threads.

Generally, the production and trade of goldbeater’s skin is better documented than membrane threads, however, the raw material may have been supplied from the same places. A document from 1288 attests of a trade of intestine membranes (“*budellas*”), specifically from oxen and cows, to make goldbeater’s skin sheets (“*buccio*”). This document gave evidence that in Lucca during the end of the thirteenth century, a lot of goldbeaters were active and the availability of membranes was in short supply, thus an agreement was signed to get guts from Lyon (in France) or to work them directly there and bring them to their workshop, without any profit^69,70^. It is possibly an interesting coincidence that many of the medieval textiles with membrane-based metal threads are attributed to Lucca.

Goat leather strips were found exclusively in Hispano-Islamic threads, and were all Z-wrapped around a single-ply core of silk fibers. Not surprisingly, goat skins were used for the contemporary production of the Hispano-Islamic “*cordobanes*”, artistic leathers made from goat skins (mostly tanned with sumac). They were first manufactured in Cordoba during the *Califal period*, and then also produced in other Spanish centers, becoming a highly demanded product in the rest of Europe^71^. They were considered durable and flexible leathers, thus, perhaps for this reason suitable for weaving purposes as well. In most goat strips, an assumed adhesive made of egg white from chicken was identified with varying levels of confidence: ovalbumin was identified with 9% coverage in samples 1902-1-311_2 and up to 73% in 1943-20-1b_2. In medieval recipe books and art technology treatises, the use of egg white (or glair as called by gilders) in the gilding of different substrates is extensively reported, either used alone (in *Mappae Clavicula*^72^ and *De diversis artibus* of Theophilus^73^, both twelfth century texts), or mixed with other binding substances (mostly animal glues and gums), additives (such as sugar, honey and even ear wax) and colouring agents (saffron and ochre), especially on leather and parchment substrates^74–76^. Moreover, Theophilus (Book I, Ch. XXXI) also recommended old glair to varnish gilding areas ^77^, and Cennini (chapter CLX) prescribed its use (or gum arabic in alternative) in grinding gold leaves to make gold powder^78^. Thus, we cannot exclude that in the samples investigated here, egg white may have been used for multiple purposes and not just as an adhesive.

A mixture of size (gelatine) and glair was also very common and particularly suitable for gilding purposes^76^. In a few cases, indeed, collagen from sheep or cattle was also identified, indicating a possible mixed adhesive of egg white with hide/bone glue (5968, 5776_1 and 1902-1-311_2). In a few samples, only the collagen was identified (1902-1-310_1 and _2, 1902-1-311_1). Type III collagen markers indicative of hide glue^79^ were identified for sheep/cattle in samples 5968, 5776_1 and 1902–1-310_1. In Heraclius’ *De coloribus et artibus Romanorum* (tenth -thirteenth century)*,* the use of egg white mixed with cattle glue is recommended to gild parchment, and especially recommended with gold powder^74,75,80^. In the three powder-coated samples investigated here (5968 and 1902-1-310_1 and _2), such an adhesive could only be confidently identified in the earlier object (5968), dated to the twelfth-thirteenth century, as noted by the strong identification of both egg white (20 ovalbumin peptides) and cattle hide glue (9 cattle-specific collagen markers). The absence of egg white peptides but the strong identification of collagen from multiple species (in total 14 specific sheep and cattle collagen markers) in sample 1902-1-310_1, dated to the fourteenth century, gives confidence to an adhesive made of hide glue alone; mixing species may have been aimed at providing stronger binding qualities. The absence of egg white might also indicate a change in the adhesives’ choice or availability over time.

The flat strips from the Eastern/Central Asian objects were made with sheep leather, as well as the wrapped strips from Middle Eastern and Italian objects. For this last group, the strip’s twist direction and core construction seem to match with those described under Category VIII by Wardwell in “Panni Tartarici”, 1989^14^. As with the goat-leather threads in which egg white was the predominant adhesive material, the association of sturgeon fish collagen glue with a sheep leather substrate was found to be consistent across all Eastern (Middle Eastern and Central Asian/Chinese) and Italian workshops. Fish glue from sturgeon, also called isinglass, is made from the dried swim-bladders of species such as *Huso Huso* (beluga sturgeon)*, Acipenser sturio* (European sea sturgeon), and *Acipenser gueldenstaedtii* (Russian sturgeon)^81^. Fish glue (gelatin) can also be made from the skin, bones and cartilages of fish^82^. In Eurasia, there are 12 species of *Acipenser*, and two species of *Huso,* but full collagen sequences are only available in NCBI for the sterlet *A. ruthenus*. Without more sequence information for the various species of sturgeon, it is unclear if the exact species present in the samples could be determined, but there are some interesting markers that might indicate different origins of the sturgeon glue. The use of bladders of “huso” (likely referring to a sturgeon species *Huso sp.*) was mentioned in Theophilus’ treatise (Book I Ch. XXX and Book III, Ch. XCII and CX) to make fish glue for gilding purposes^73^. The use of swim bladders to prepare glue similar to the western isinglass also appeared in a seventeenth century Chinese treatise (*T’ien-kung K’ai-wu*)^83^. In objects of more recent period (early twentieth century) from eastern China, fish glue from a *Larimichthys* species (yellow croacker) was found as adhesive of gilded leather strips; the species corresponded to the geographical area where the objects (children’s hat) were likely made^21^.

Already in the tenth century, fish glue was considered a stable adhesive for gold printing on textiles, as recommended by Muḥammad b. Aḥmad b. Saʿīd al-Tamīmī (Egypt)^84^. Fish glue was a common binder for gilding in Europe, especially for manuscript illumination on parchment^77,85^. It was considered a highly penetrative adhesive, and as such not suitable for thin leathers^86^, perhaps one of the reason it was used on sheep skins but not goat skins that are generally thinner (for skin-based strips’ thickness comparison see Supplementary SI-2.C). Fish glue, either gelatin or isinglass, is more elastic than hide glue, a property that confers an advantage for wrapping threads, while mammalian hide glue produces a stronger glue than fish^82^. While identification of hide glue from sheep is hindered by the sheep identification of the skin substrate, in a few threads, fish glue appeared to be mixed with mammalian hide glue obtained from cattle (1902-1-262_1 possibly as contamination, and 1862:16 I) and, more surprisingly, from horse (D12b, 1862:16 III and IV). Fish glue and horse glue are both reported as ideal glues for the manufacture of Mongol bows in the Book of Artificers (an encyclopedia of Chinese science and technology, dated to the 5th BC^87–89^, and horses were extensively bred and exploited by nomadic populations of Mongolia and China. The identification of horse implies a relation between the workshops under Mongolian rule and the choice of raw materials or their availability in each geographical area.

A particularity in flat strips was the use of a second adhesive layer on the backside of the leather, even when no metal was applied. Such a coating has been noted before^15,32^, and a few cases have been reported to be gilded on both sides^15^. Márta Járó described a flat strip coated with gold on one side and gilt-silver on the other side^1^, as found in samples 1862:16 III and IV. The adhesive layer might have been applied with the intention of being metal-coated, or most likely as a protective coating to enhance the strip strength and prepare it for the weaving process, similarly to the modern sizing of warp fibers. The addition of mammalian glue in the adhesives of the flat strips might have been done purposefully to favor strength rather than flexibility. Even more intriguing was the presence of an intermediate adhesive layer in the threads from 1862:16 I and V, revealing the binding of two skin layers to form a much thicker strip, and in sample 1862:16 V of wheat and barley peptides, possibly originating from a wheat flour paste which is a known adhesive to stick papers^83^, parchments and leathers^90^. Sample 1862:16 V was also the only sample having a yellow coat layer on top of a silver coating. Proteins were often added to ground layers of Asian lacquer as a binder, among them animal glues^91^ and wheat glue was used in East Asian lacquer^92^, thus we cannot exclude that a protein glue was also added to the ground layer of the top coating, even though the cross-section of the fragment analyzed was inconclusive.

A small group of textiles, dated from the 13th to the fourteenth-fifteenth century, of Middle Eastern (1902-1-385 and D12b) and Italian (I A7) provenance, deserves special attention. While the proteomics results are consistent with the given origin of the threads, the construction of the threads, Z-twisted strips wound around a single-ply yellow silk core, is the same as in all the Hispano-Islamic samples. This special combination of features may lead to ascribe the manufacture of this group to a specific workshop within the Middle Eastern production area, possibly in Iran. Similar fragments in museums worldwide claim different origins, usually Italian and/or Iranian; for many Tabriz is said to be the manufacturing center. Tabrīz was the capital of the Mongol Empire under *Il-Khan Maḥmūd Ghāzān* (1295–1304) and his successor, and interestingly, by the middle of the thirteenth century, a residential colony of Italian merchants was established there^93^. This specific pattern of fabrication corresponds to Wardwell’s Category VI of “Panni Tartarici”, also claiming a Tabriz manufacture^14^. Particularly noteworthy was the detection of horse collagen (otherwise only found in two flat strips samples) on the back side of the wrapped-skin sample D12b, a feature that could be associated with Mongolian production.

Only gilt-silver coatings were identified on membrane strips, in agreement with the literature, even though silver coatings have also been reported^1,4,5,9,10,32^. However, in the light of the present investigation, silver coatings alone might not have been used but rather the worn gilding led to misinterpretations. Indeed, the gold presence is often clearly detectable, even if spotty, when the whole textile is examined.

It is known that the most common gilding technique of silver in Europe in medieval times was the fire-gilding technique. Even if only a selected set of samples was analyzed by SEM-µXRF, the absence of mercury in many of them is challenging to interpret. However, due to the high volatility of mercury, the element is not easy to detect^49^, and its presence may not be totally excluded. A gilding technique that we feel confident to exclude, at least on membrane strips based on the large number of samples analyzed, is the one consisting of applying the gold leaf on the silver (or copper) surface by the use of an adhesive such as egg white, mentioned by *Heraclius*, Le Begué^80^, as well as *Theophilus* in *De diversis artibus*^94^, which presence was not detected by proteomics in any of the membrane samples analyzed. However**,** on a leather-based strip with a gilt-silver coating (1902-1-311_2), traces of egg white were detected with two peptides, perhaps the remains of gilding the silver with a glair-based adhesive, thus challenging the initial hypothesis that egg white was used in a mixed adhesive with hide glue for binding the gilt-silver leaf to the skin support.

On leather strips, a broader variety of coatings composition and morphology (leaf and powder) was found. Within the three different compositional groups defined (silver, gold and gilt-silver coatings), gold coatings were the most represented. It is largely known, that the main supply of gold for medieval painters and goldbeaters was the coinage current in their time^76^. It represented an easily accessible source of gold, with a guaranteed quality, since gold coins were produced according to fixed criteria established by authorities, ensuring the purity of the gold. The Italian Florin, with a gold content of ca. 99 wt%^95^, was the most important gold coin circulating in Europe and also in the Levant, where the Venetian Ducat replaced it in the first half of the fourteenth century^96,97^, and which standard was adopted for Islamic coins^98^. Cennini, in his treatise, point to the use of Venetian gold Ducats to make gold leaves^78,96^. Interestingly, the purest gold coatings identified on the samples under investigation, showed a composition that match with the one from Ducats and Florins, and belong to Italian, Middle Eastern and Asian/Chinese objects dated to thirteenth and fourteenth centuries. The use of gold leaves of similar composition may prove the extensive flow of gold threads, or at least of gold leaves, during that period through the trade routes joining the Mediterranean to the Chinese Sea after the Mongol conquest of Asia.

The variety of coatings composition found in Hispano-Islamic samples, as well, seems to correspond to the variable composition of the coinage used during the Arab domination in the Iberian Peninsula, reflecting the successive economical contexts of each Kingdom that minted its own coinage. The gold content of the coins minted after 1100 did not exceed 90%^99^, a composition similar to the one found in the gold coatings on Hispano-Islamic threads, and that also led to the hypothesis that sample 1965-33-5_1, having a gold coating of higher quality (23.16 k), could be manufactured in earlier periods. The coins minted in the later *Nazari Kingdom,* which suffered financial difficulties and a lack of gold supply, were mostly silver-made (“dirham”), then probably gilded^99^. This data again match with our two findings on fourteenth century examples, supported also by the study conducted by Borrego et al.^10^.

Even if it is not possible to prove that circulating coins were used to produce the metal coatings of animal-based metal threads that have been analyzed in our study, their diverse composition according to the geographical area of provenance of the objects, may be representative of the economic and political context that marked these territories within the time period investigated (eleventh–fifteenth century), and as a consequence the gold and precious metals sources and technologies in ordinary use.

Finally, the discovery in two textiles of human proteins as a record of past handling and use was an unexpected consequence of this project. In 1902-1-229b, the skin proteins might have been shed from someone with a mild skin wound or skin lesion and a relatively recent contamination, perhaps from someone handling the textile when it first arrived at the museum at the beginning of the twentieth century^100^. In P4d, the human contamination seems to come from a response to a fungal infection. This could come from spore inhalation from contaminated food (e.g. moldy barley), provoking an allergic reaction or a more serious case of aspergillosis. In both cases, we cannot know for certain when the human proteins were deposited on the threads, whether during the construction of the threads or later, considering the long use and re-use of these fabrics or their many handlings. However, these threads offer yet another opportunity to obtain glimpses on object’s biographies as well as on people’s story, as has recently been shown from the recovery of human proteins from other substrates (e.g. birth girdle^43^, Chekhov’s death shirt^101^).

## Conclusions

By combining paleoproteomics with microanalysis, the present research is the first study entirely focused on metal threads backed with an animal support, providing the most complete analysis of the morphology and elementary composition of the metal coating, as well as the first characterization of the proteinaceous tissues and adhesives in a large range of threads. The identified materials and assembling techniques allowed to define consistent patterns of fabrication, possibly suggesting that the choice of raw materials and construction techniques depended on their local availability and current use in other crafts at that time. A firm geographical assignment of the threads is still challenging, and can only be improved with the investigation of more samples from well-known objects of certain provenance and date. Textiles attributions have usually been made on the basis of stylistic and aesthetic parallelism, leading sometimes to confusion and contradiction, especially due to the migration of motifs and patterns from one area to another, resulting in an international style of silk design^93^. Moreover, across Eurasian workshops, the intense trade of fabrics, as well as of raw materials, and the continuous movements or forced relocation of textile workers, have obscured the origins of many textiles of that time^17^. Thereby, the present study would emphasize that animal-based metal thread materials and manufacturing techniques provide further criteria to classify and group textiles, and trace correlations between manufacturing centers within Eurasian territories, according to the use of a specific type of metal thread, sometimes in disagreement with museum’s assignments.

In addition to expanding the corpus of samples (especially to samples of Central/East Asian origins that showed the most variation in their proteinic compositions), further research should focus on improving the analytical strategy, expand the search for materials and biomolecular information with other analytical techniques, and combine the analytical results with the modern reproduction of membrane and skin threads. Moreover, further research needs to be carried out to improve the cross-sectioning method and the micro-sampling. For flat strips where up to three layers of adhesive were found, methods should be developed to attempt the identification of each layer separately. In wrapped threads, the back of the strips needs to be studied to explore if any traces of adhesives were used to guarantee the wrapping twist around the core. Immunofluorescence microscopy^102,103^ or chemical mapping by MALDI-MSI^104^ in cross-sections could be used to localize each binding media within the stratigraphy of the samples, for example to verify that they were used exclusively in adhesive layers or for other purposes (e.g. in varnish layers, in the strips supports, etc.).

Considering the incredible variety of ingredients mentioned in medieval gilding recipes, other classes of organic components (from oils and waxes, to sugars, gums or natural resins), that could have been used in association with the protein-based binders, need to be investigated with other analytical techniques. Besides characterizing the nature of materials and their species, biomolecular studies such as isotopic studies and DNA analysis could bring additional resolution on the origin of the substrates and exact species found in the sturgeon glue. Finally, the coating method on membrane threads by gilt-silver leaves remain to be determined, as neither cross-sections nor proteomics were conclusive regarding the presence of an adhesive layer.

While our scientific investigation of ancient samples has provided some answers and set the baseline for the establishment of an efficient analytical protocol, reproducing membrane and skin threads is key to understand how the threads were made. Trying the different adhesives and coating techniques on experimental threads while testing their spinning and weaving capabilities can underline their resistance to mechanical wear and other environmental damages as well as their visual appeal in the textiles. In that regards, our study has been the reference point for a recent experimental replication of membrane-based type of threads (private communication by Drs. Kania and Niepold). Despite the great popularity of metal threads on an organic substrate, they have been poorly documented in historical records, maybe intentionally kept to secret. The outcomes presented here have highlighted the intricate correlations existing between the making of animal-based metal threads and other contemporary crafts (e.g. goldbeating, leather and manuscripts gilding techniques, etc.), proving the high skill and expertise of the artisans (men and women) dedicated to such a fascinating and complex manufacture.

## Methods summary

### Micro-analysis

The close-up examination of textile objects, conducted on 45 fragments, was performed with portable digital microscopes: Dino-Lite (DUNWELL TECH, INC. DINO-LITE US) and MiScope—MP2 portable digital microscope (ZARBECO LLC, NJ, USA). Most of the samples were documented using an HIROX KH-8700 3D digital microscope (HIROX-USA, INC., NJ) at different magnifications (from 25 × to 250 ×) (MCI). Samples from 13 objects were observed by using a LEICA M205C stereomicroscope (magnifications up to 160x) equipped with a LED light source (OPD). Cross-sections were prepared by embedding micro-fragments of the samples perpendicularly in polyester resin (BIO-PLASTIC AND *PRESI* 2S), and grinding and polishing the resin blocks with silicon carbide discs (grit up to grade 1200). They were observed at different magnifications (from 5 × to 50 ×) by a LEICA DM LM/DM 2500 optical microscope (OM2) in reflected light, bright field and UV fluorescence, filter cube A (BP 340-380, FT 400, and LP 425). 9 cross-sections were observed using a ZEISS Axio Imager.A1 microscope (magnifications from 10 × to 50 ×) (OM1), equipped with a mercury vapor lamp HBO100 and filter set 49 (G 365, FT 395, BP 445/450) for fluorescence microscopy. Characteristic measurements (strips width and thickness) were carried out on calibrated micrographs by ImageJ software, Fiji version 1.53f51 (National Institutes of Health, Bethesda, Maryland, USA).

### SEM-EDS

Compositional imaging and analysis were performed using an HITACHI S3700N scanning electron microscope (SEM) equipped with a BRUKER 6|60 silicon drift X-ray detector and ESPRIT 2 analysis software. Samples were mounted on carbon double stick adhesive tape (from an electron microscopy vendor) onto an aluminum stub and analyzed at a working distance of 10 mm with a 15 kV electron beam and on the order of ≈ 1 nA of current in variable pressure. A large solid angle 5 segment backscattered electron detector was also used for average atomic number imaging. Hyperspectral X-ray imaging of the specimens allowed for extraction of spectra from compositional images. P/B ZAF matrix corrections were applied to raw X-ray data for quantification; these results were then normalized and reported in elemental mass percent. The morphological characterization of the metal coating and the strip support was conducted in BSE mode.

### SEM-µXRF

Nine skin-based samples and eight membrane-based samples were mounted on an aluminum stub using a carbon double stick adhesive tape (from an electron microscopy vendor) and analyzed by SEM-µXRF to complement SEM–EDS analysis and determine trace elements from a qualitative point of view. Using the HITACHI 3700N, a BRUKER XTrace is used as the μX-ray Fluorescence source yielding a spot size ≈ 33 μm (measured using Cu Kα1) with the BRUKER 6|60 serving as the silicon drift X-ray detector. The conditions of 50 kV for the high voltage and 600 μA and a 12.5 μm aluminum filter was used. To achieve μXRF mapping, a piezo stick–slip substage (termed “rapid stage” by BRUKER) capable of 5 cm travel in both X and Y axes was used. Maps were collected with a 24 ms per pixel dwell. μXRF data were also analyzed using the ESPRIT 2 software.

### PROTEOMICS

Extraction, digestion and purification according to previously published protocols^105^, and are detailed in Supplementary SI-3_Proteomics. Protein analysis was conducted by nanoLC-Orbitrap MS/MS using a THERMO SCIENTIFIC DIONEX Ultimate 3000 UHPLC (equipped with THERMO BioBasic C18 precolumn (30 mm × 75 µm i.d.) and in-house packed analytical column (210 mm × 75 µm i.d.) made of the same stationary phase). The UHPLC was directly coupled to a THERMO SCIENTIFIC LTQ Orbitrap Velos mass spectrometer which analyzed the peptides in positive mode. For bioinformatics analysis, two fractions of each sample were combined into one search to create one output file. PEAKS 8.5 (BIOINFORMATICS SOLUTIONS INC.) was used to search the RAW data for matches against publicly available sequences of mammals, birds and fish species in imported UniProt (www.uniprot.org) and NCBI (https://www.ncbi.nlm.nih.gov/protein) databases.

Full details of the Methods are reported in Supplementary SI-1.B.

### Supplementary Information

Supplementary Information 1.

Supplementary Information 2.

Supplementary Information 3.

Supplementary Information 4.

Supplementary Information 5.

Supplementary Information 6.

## Data Availability

The mass spectrometry proteomics data have been deposited to the ProteomeXchange, Consortium (http://proteomecentral.proteomexchange.org) via the MassIVE partner repository with the dataset identifier MSV000092355 for the membrane-based threads, and MSV000092358 for the skin-based threads.
