# Supplementary material for: Palaeoproteomics and microanalysis reveal techniques of production of animal-based metal threads in medieval textiles
Source: Sci Rep. 2024 Mar 4;14:5320. doi: 10.1038/s41598-024-54480-4 (PMC10912450; doi:10.1038/s41598-024-54480-4)
Supplement: Supplementary file 1 — Supplementary Information 1. [file 41598_2024_54480_MOESM1_ESM.pdf]

SI-1_Samples and Methods

Palaeoproteomics and microanalysis reveal techniques of production of animal-based metal threads in medieval textiles

This PDF file includes:

Supplementary Text and Tables

Contents:

1.A Sampling
1.B Methods

List of tables:

Table S1.A1 Textile objects and sample corpus
Table S1.B1 Analytical plan

1.A Sampling

For the present investigation, a corpus of 66 textile fragments belonging to 54 catalogued objects from one American and six European museum collections was formed. The main criteria for selection was to cover as much as possible the historical period when these types of threads were used and the main geographical areas of production (as determined by museums' assessment), in order to characterize and compare European and Oriental patterns of fabrication. Apart from one object attributed to Germany, and one of Italian or Flemish provenance, the European textiles were either Italian or Spanish. Oriental samples come from Middle Eastern pieces regarded as been made in Iran or Iraq by Persian artisans, and from Central Asian, Chinese and more generically Far Eastern objects.

The textiles range from the 11th-12th century to the 16th century, with a majority from the 13th-14th century; in some cases, they are precisely related to a geographical center, whereas in other cases the provenance is uncertain, especially for many 14th-15th century fabrics.

The objects under investigation include mostly textile fragments, but also fabric parts of five dalmatics (D10, D11, D12, D13, 1862:16) and six copes (P1, P7, P9, P10, P11, P12), and a relic purse (3RU8457). They represent a range of weaving techniques: while the majority of them are lampas weave, some pieces also feature samite (including half-silks), taqueté, tabby, twill, as well as two examples of velvet (1902-1-385 and I A7), possibly coming from the same manufacturing center, and a tapestry (3RU8457). Similar fragments within different collections were also selected (1902-1-216 and 313*, 1902-1-977 and I A5bis, 1902-1-385 and I A7), to compare metal threads technological and compositional features.

The nature of the metal threads was often not precisely reported in museum archive's entries or the online catalogues, where many terms can be found: "membrane threads", "metal threads", "metal wrapped", "Cyprus gold". Only in some cases a more detailed description was given, i.e. "beaten gold on vellum or membrane", "gilt leather threads". Thereby, the selection of the objects to sample was based on the thread's appearance on textile images (when the collection was explored through the online catalogue), or on the specific choice and selection carried on by conservators.

For the set of objects under investigation, several samples were taken whenever possible; they consist of strip fragments or fragments of complete threads including the fibrous core, sometimes from the same textile area and sometimes from different areas. The sampling was conducted by the textile conservators of each collection in order to be as respectful as possible of each museum sampling protocol. Due to the limited access to some textiles (especially when mounted on a support), or the fragile conservation state of the pieces, the sampling from different and representative areas was not always feasible. The most suitable areas, indeed, were along the edges, on the reverse of the textile (when it was accessible), or in areas where metal threads were loose from the weaving structure.

In **Table S1.A1** a detailed description of each object and the samples taken from each textile fragment is reported. The objects are identified by the accession numbers given by the museums; when they are formed by multiple separate fragments, the accession number is followed by a lowercase letter indicating the fragment sampled, e.g. 1902-1-271 is made of four fragments "a" to "d", but only fragment "a" was sampled, i.e. 1902-1-271a. Numbers in brackets or after the underscore (e.g. 1965-33-5 (1) or 1965-33-5_1) refer to the sample analyzed (see **Table 2** and **Table 3**).

Table S1.A1. Textile objects and sample corpus. The set of objects investigated is presented by museum. For each textile is indicated the corresponding accession number (Acc. No.), museum's date and original attribution (century – geographic area), as well as a brief description. For each object, the list of samples investigated is provided. For some objects, the chronological and geographical assignment is still "research in progress". CC= Ente Chiesa Cattedrale di Como (Como, Italy); CDMT=Museu Tèxtil, formerly Centre de Documentació i Museu Tèxtil (Terrassa, Spain); CG=Collezione Tessile Gandini, Museo Civico (Modena, Italy); CH=Cooper Hewitt Smithsonian Design Museum (New York, US); DB= Domstift Brandenburg (Brandenburg/Havel, Germany); MTP=Museo del Tessuto (Prato, Italy); SM= Stralsund Museum (Stralsund, Germany); ar=Arabic term; sp=Spanish term

Museum	Acc. No.	Attribution	Description	Samples
**CC**	3RU8457	$13^{th}/14^{th}$ –Italy or Flanders	Textile – accessories<br>Aumônière (relic purse) – different fabrics<br>(Front) Tapestry: silk, metal threads<br>H x W: 32.5 x 35 cm	(1) (2)<br>[samples provided by the Abegg-Stiftung Foundation of Riggisberg]
**CDMT**	164	possibly 14th - Persia<br>(Iran or Iraq)	Woven textile - fragment<br>Lampas: silk, metal threads<br>H x W: 26 x 18 cm	(1)
	313*	$12^{th}/13^{th}$ - Spain<br>(Al-Andalus)	Woven textile – different fragments<br>"The sphinx fabric"<br>Lampas: silk, metal threads<br>H x W: not measured<br>*Object of research in progress<br>*Provenance*: tomb of St. Bernard Calvó, Bishop of Vic (d. 1243), Cathedral of Vic, Spain<br>Similar fragments: **CH *1902-1-216***	(1)
	5776	13th - Spain<br>(Al-Andalus)	Woven textile – different fragments<br>"The eagle fabric"<br>Lampas: silk, metal threads<br>H x W: 7 x 45; 8 x 50; 2.5 x 11.5<br>*Provenance*: Sant Pere Cercada, Spain	(1)
	5798	$14^{th}/15^{th}$ - Italy<br>(possibly Lucca)	Woven textile - different fragments<br>Lampas: silk, metal threads<br>H x W: 6.5 x 7 cm; 15.5 x 8 cm	(1)
	5963	$13^{th}/14^{th}$ - Iran	Woven textile - fragment<br>Lampas/ double-cloth: silk, metal threads<br>H x W: 8.5 x 13 (Mount: 25 x 34.5 cm)	(1) (2)
	5968	$12^{th}/13^{th}$ – possibly Spain	Woven textile - fragment<br>Tabby ("Sendal"): silk, metal threads<br>H x W: 5.5 x 8 cm	(1)
	6369	$12^{th}/13^{th}$ – possibly Spain	Woven textile – fragments a, b<br>Tabby ("Sendal"): silk, metal threads<br>**b:** H x W: 9 x 6 cm	**b:** (1)
	6372	$12^{th}/13^{th}$ - Spain	Woven textile - fragment<br>Twill: silk, metal threads<br>H x W: 10 x 8 cm	(1)

Museum	Acc. No.	Attribution	Description	Samples
**CG**	I A 5bis	13th (before 1274) – Spain	Woven textile - fragment<br>Taqueté: silk, metal threads<br>H x W: 19 x 8 cm<br>*Provenance*: tomb of Don Felipe, Infante of Castile (d. 1274), Villalcázar de Sirga, Palencia, Spain<br>Similar fragments: **CH *1902-1-977***	(1)
	I A 7	14th/15th – Italy (possibly Florence)	Woven textile - fragment<br>Cut velvet: silk, metal threads<br>H x W: 8.3 x 23 cm<br>Similar fragments: CH *1902-1-385*	(2) (3)
	I I161	half 14th – Italy (Florence or Lucca)	Woven textile - fragment<br>Lampas: silk, metal threads<br>H x W: 30 x 25 cm	(1)
**CH**	1902-1-216	12th/13th - Spain	Woven textile - fragment<br>“The sphinx fabric”<br>Taqueté: silk, metal threads<br>H x W: 31.8 x 33 cm (Mount: 45.7 x 61 cm)<br>*Provenance*: tomb of St. Bernard Calvó, Bishop of Vic (d. 1243), Cathedral of Vic, Spain<br>Similar fragments: **CDMT *313****	(1)
	1902-1-227	13th - Italy	Woven textile - fragment<br>Samite: silk, metal threads<br>Warp x Weft: 14 x 17.8 cm	(1) (2)
	1902-1-229	13th - Spain	Woven textile – fragments a-c<br>**b:** Tabby: silk, metal threads<br>H x W: 16.4 x 18.1 cm<br>*Provenance*: tomb of Don Felipe, Infante of Castile (d. 1274), Villalcázar de Sirga, Palencia, Spain	**b:** (1) (2)
	1902-1-233	14th – Spain or Iran	Woven textile - fragment<br>Lampas: silk, metal threads<br>H x W: 17 x 11.2 cm	(1)
	1902-1-240	13th – Spain	Woven textile - fragment<br>Samite: silk, metal threads<br>H x W: 12.8 x 19.7 cm	(1) (2)
	1902-1-241	13th – Spain	Woven textile - fragments a, b<br>Samite, half-silk: silk, linen, metal threads<br>**a:** Warp x Weft: 16.8 x 37.5 cm<br>*Provenance*: ascribable to the Cope of Sant Cugat, worn by Abbot Arnaldo Ramón Biure (d. 1351), formerly in the monastery of San Cugat del Vallès, Catalonia, Spain	**a:** (2) (3)
	1902-1-250	14th - Italy	Woven textile – fragment (two rectangular fragments sewed together to form a T-shaped piece)<br>Samite, half-silk: silk, linen, metal threads<br>H x W: 26.7 x 23.5 cm	(1) (2)
	1902-1-251	15th – possibly Spain	Woven textile – fragment (two fragments sewed together)<br>Lampas: silk, metal threads<br>Warp x Weft: 42.5 x 33 cm	(1) (2)
	1902-1-253	13th - Spain	Woven textile - fragments a, b<br>Samite: silk, metal threads<br>**a:** Warp x Weft: 41 x 19 cm<br>*Provenance*: altar frontal in the monastery of San Juan de las Abadesas, Ripolles, Catalonia, Spain	**a:** (1) (2)
	1902-1-257	14th - Italy	Woven textile - fragments a-d<br>Lampas: silk, metal threads<br>**b:** H x W: 17.1 x 22.2 cm<br>**d:** H x W: 14.9 x 10.8 cm	**b:** (1)<br>**d:** (2) (3)

Museum	Acc. No.	Attribution	Description	Samples
	1902-1-262	14th - Italy	Woven textile - fragment<br>Lampas: silk, metal threads<br>Warp x Weft: 61.6 x 43.8 cm	(1) (2) (3)
	1902-1-271	14th - Italy	Woven textile - fragments a-d<br>Lampas: silk, metal threads<br>**a:** H x W: 40 x 20.5 cm	**a:** (1) (2) (3)
	1902-1-272	14th - Italy	Woven textile - fragment<br>Lampas: silk, metal threads<br>H x W: 25.6 x 59.8 cm	(1) (2)
	1902-1-273	14th – Italy	Woven textile - fragments a, b<br>Lampas: silk, metal threads<br>**a:** H x W: 47.6 x 21.9 cm<br>**b:** H x W: 15.2 x 26.7 cm	**a:** (1)<br>**b:** (1)
	1902-1-274	$14^{th}/15^{th}$– Italy (possibly Lucca)	Woven textile - fragments a, b<br>Lampas: silk, metal threads<br>Warp x Weft: 51 x 24 cm	**a:** (1)<br>**b:** (1)
	1902-1-279	14th - Italy	Woven textile - fragment<br>Lampas: silk, metal threads<br>Warp x Weft: 26.7 x 38.1 cm	(1) (2)
	1902-1-285	$14^{th}/15^{th}$ - Italy	Woven textile - fragment<br>H x W: 43.8 x 29.5 cm<br>Lampas: silk, metal threads	Gold-looking threads<br>(1) (2)
	1902-1-292	14th - Italy	Woven textile - fragments a, b<br>Lampas: silk, metal threads<br>**a:** H x W: 15.6 x 28.9 cm<br>**b:** H x W: 15.6 x 23.8 cm	**a:** (1)<br>**b:** (1)
**CH**	1902-1-310	14th - Spain	Woven textile - fragment<br>Lampas: silk, metal threads<br>H x W: 29.1 x 20.1 cm	(1) (2)
	1902-1-311	14th – Spain	Woven textile - fragment<br>Lampas: silk, metal threads<br>H x W: 35.6 x 25.4 cm	(1) (2)
	1902-1-329	14th - Italy	Woven textile - fragments a, b<br>Lampas: silk, metal threads<br>**a:** Warp x Weft: 42.8 x 23 cm	**a:** (1) (2)
	1902-1-385	possibly 13th – Iran	Woven textile - fragment<br>Cut velvet: silk, metal threads<br>H x W: 53 x 26 cm<br>Similar fragments: **CG *I A 7***	(2)
	1902-1-977	13th - Spain	Woven textile - fragments a-d<br>**c:** fragment of the "Jubba" (ar.) or "Aljuba" (sp.)<br>Taqueté: silk, metal threads<br>H x W: 18.4 x 33.7 cm<br>*Provenance*: tomb of Don Felipe, Infante of Castile (d. 1274), Villalcázar de Sirga, Palencia, Spain<br>Similar fragments: **CG *I A 5bis***	**c:** (3)
	1938-78-1	13th - Spain	Woven textile - fragment<br>"Textile of stars", it belongs to a collection of liturgical garments, in detail the "qaba" (ar.) or "capa" (sp.)<br>Pseudo-lampas [1], double cloth ground –tabby pattern: silk, metal threads<br>Warp x Weft: 18.1 x 16.8 cm<br>*Provenance*: attributed to the cult of Saint Valerius, bishop of Saragossa (290-315). Formerly in the Cathedral of San Vicente de Roda d'Isábena (1279), Huesca, then moved to the Cathedral of Lérida (15th c.), finally sold to the Barcelona museums (1932)	(2)

Museum	Acc. No.	Attribution	Description	Samples
**CH**	1938-84-1	14th - Italy	Woven textile – fragments (two)<br>Lampas, “Diasprum” [2]: silk, metal threads<br>H x W: 79 x 27 cm	(1) (2)
	1943-20-1	13th - Spain	Woven textile - fragments a, b<br>“Textile of lions”, it belongs to a collection of liturgical garments, in detail the “casulla” (sp.) or chasuble<br>**b:** Taqueté: silk, metal threads<br>H x W: 6.7 x 1.6 cm<br>*Provenance*: attributed to the cult of Saint Valerius, bishop of Saragossa (290-315). Formerly in the Cathedral of San Vicente de Roda d’Isábena (1279), Huesca, then moved to the Cathedral of Lérida (15th c.), finally sold to the Barcelona museums (1932)	**b:** (2)
	1965-33-2	11th/12th - Spain	Woven textile - fragment<br>Lampas: silk, metal threads<br>H x W: 17 x 90 cm<br>*Provenance*: part of the so-called “Baghdad silk” group of textiles [3-5]	(1) (2)
	1965-33-5	12th/13th - Spain	Woven textile – fragment<br>“Textile with Musicians”<br>Pseudo-lampas [1,6], double cloth ground- tabby pattern: silk, metal threads<br>Warp x Weft: 40 x 11.5 cm<br>*Provenance*: considered by some authors as part of the bookbinding of a 13th century manuscript in the Cathedral of Vic, Spain [7,8]	(1) (2)
**DB**	D10	end 14th - Italy	Textile - ecclesiastical garment<br>Tunic (H x W: 118 x 137 cm) - outer fabric<br>Lampas: silk, metal threads	(1)
	D11	end 14th - Italy	Textile - ecclesiastical garment<br>Dalmatic (H x W: 118 x 137 cm) – outer fabric<br>Lampas: silk, metal threads	(1)
	D12	around 1300 - possibly Persia	Textile - ecclesiastical garment<br>Dalmatic (H x W: 129 x 159 cm) – different fabrics<br>**b:** “Zapfenstoff”<br>Lampas: silk, metal threads	**b:** (1)
	D13	**a:** end 13th - Far East<br>**e:** 14th – possibly Italy	Textile - ecclesiastical garment<br>Tunic (H x W: 130 x 153 cm) – different fabrics<br>**a:** *“Drachenstoff”*<br>Lampas: silk, metal threads<br>**e:** Dolphins fabric<br>Taqueté faconné, half-silk: silk, linen, metal threads	**a:** (1)<br>**e:** (1)
	P1	14th - Germany	Textile - ecclesiastical garment<br>Cope (H x W: 143 x 302 cm) – braid (clasp)<br>Samite, half-silk: silk, linen, metal threads<br>H x W: 9 x 21 cm	(1)
	P4	2nd half 13th - China	Woven textile – fragments c, d<br>Patches from cope P4<br>Lampas: silk, metal threads<br>**c:** H x W: 46 x 30 cm<br>**d:** H x W: 27 x 25 cm	**c:** (1)<br>**d:** (1)
	P7	**i:** 2nd half 14th - Italy<br>**k:** end 14th - Italy<br>**l:** middle 15th - Italy	Textile - ecclesiastical garment<br>Cope (H x W: 148 x 303 cm) – different fabrics<br>Lampas: silk, metal threads<br>**i:** outer fabric<br>**k:** fragment lower edge<br>**l:** hood	**i:** (1)<br>**k:** (1)<br>**l:** (1)

Museum	Acc. No.	Attribution	Description	Samples
**DB**	P9	1st third 15th - Italy	Textile - ecclesiastical garment<br>Cope (H x W: 145 x 305 cm) - outer fabric<br>Lampas: silk, metal threads	(1)
	P10	2nd half 14th - Italy	Textile - ecclesiastical garment<br>Cope (H x W: 146 x 309 cm) - outer fabric<br>Lampas: silk, metal threads	(1)
	P11	14th - Italy	Textile - ecclesiastical garment<br>Cope (H x W: 121 x 279 cm) - outer fabric<br>Lampas: silk, metal threads	(1)
	P12	around 1500 - Italy	Textile - ecclesiastical garment<br>Cope (H x W: 138 x 318 cm) - outer fabric<br>Lampas: silk, metal threads	(1)
**MTP**	03.02.02	14th - Persia<br>(Iran or Iraq)	Woven textile - fragment<br>Lampas: silk, metal threads<br>H x W: 53 x 21 cm	(2) (3)
	81.01.01<br>bis	half 14th –<br>Italy (possibly<br>Lucca)	Woven textile - fragment<br>Lampas: silk, metal threads<br>H x W: 36.5 x 22 cm	(1)
**SM**	1862:16	1st half 14th -<br>Central Asia or<br>Northern China	Textile - ecclesiastical garment<br>Dalmatic – five different fabrics<br>Lampas: silk, metal threads<br>**I:** (Front) central rectangular band and upper rectangular inserts on the sleeves<br>**II:** (Front) side trapezoid inserts<br>**III:** (Front) lower rectangular bands on the sleeves<br>**IV:** (Front) lower trimming and side smaller trapezoid inserts<br>**V:** (Front) triangular inserts on sleeves<br>*Provenance*: Stralsunder Kaland brotherhood at St Nicholas' Church, Stralsund	**I:** (1)<br>**II:** (1)<br>**III:** (1)<br>**IV:** (1)<br>**V:** (1)

1.B Methods

Optical Microscopy (OM)

48 samples were selected for the preparation of cross-sections, based on the suitable size and geometry to be embedded. Thus, while for some objects cross-sections of more than one sample were prepared, for others, no sample was embedded due to its low representativeness (see **Table 2** and **Table 3**).

A total of 28 cross-sections from skin-based samples and 20 from membrane-based samples were prepared and examined in two different laboratories (Opificio delle Pietre Dure, OPD, and Museum Conservation Institute, Smithsonian Institution, MCI).

Polyester resins, *BIO-PLASTIC* (MCI) and *PRESI* 2S (OPD), ratio of resin/hardener of 1: 1, were used as embedding medium for the yellowing resistance, despite the infiltrations drawbacks perceived in some skin-based strips having a less compact and dense fibrous weave. The first stages of the grinding process were conducted in wet mode and by a grinding machine with successively finer grades of silicon carbide discs (grit from p120 to p1200). Wet grinding provides a flushing action for loose particles, but could also solubilize components of the sample as glues. For this reason, the last grinding stages were conducted in dry conditions, to avoid the solubilization of layers components. When the sample was close to the resin block surface, the final polishing was manually carried out by micro-mesh abrasive cloths (up to 1200 mesh grades), in order to avoid the loss of the sample.

Starting from the consideration of animal-based metal threads as stratified structures, the examination of cross-sections by fluorescence microscopy was carried out to determine the multi-layered sequence of the strips and preliminary identify the nature of the materials that make up each layer, according to their intrinsic fluorescence or auto-fluorescence. Proteinaceous materials are generally excited using UV incident radiation with a wavelength ranging from 280 to 365 nm [9], thus the set of filters used for fluorescence microscopy had an excitation emission of 340/380 (OM2) and 365 (OM1). A series of optical micrographs of cross-sections at different depth was collected, exploring different slices of the stratigraphy of the samples by successive grinding/polishing cycles. Maintaining the sample stationary and getting a flat surface for observations was often challenging due to the tiny dimensions of the samples and, for wrapped-threads, their geometry already altered by the spinning process. Moreover, the thinness and non-homogeneity of the layers, and the natural aging of materials, especially organic ones, had slightly altered the natural fluorescence and their solubility properties, representing a limitation in the interpretation of the visual information brought by cross-sectional images.

For comparison purposes, the thickness of the strip substrates and adhesive layers of samples was measured on calibrated UV fluorescence cross-sectional images by *ImageJ software*. The great variability of values recorded, even within the same sample, was strongly influenced by the not perfectly perpendicular exposed surface of all the layers of the strips. Thus, to get representative values, more than a measurement was taken in different points of the cross-section (excluding those where the strip’s geometry appeared most altered), and the average value calculated. The values correspond to the overall strip thickness observed in cross-section.

The examination of cross-sections also in bright field (BF) was performed to investigate the morphology of the metal coating according to the profile of the metal layer observed within the strip stratigraphy.

Proteomics

For most threads, the sample was too small to be divided and the whole sample was processed in one single extraction. This represented about 0.1 mg or less, and samples of about 1 mm in length. Reference skin samples (raw hide, parchment, vellum and leather) were obtained from archive materials at the Museum Conservation Institute. Replica membranes were obtained from Elisabeth Delvai during completion of her master degree at the Institute of Conservation, University of Applied Arts Vienna [10,11]. For the reference materials, samples of 1 x 1 mm were processed the same way as metal threads.

Extraction: Proteins were extracted by overnight shaking in 100 µL of a solution of 8M urea, 50 mM Tris and 50 mM tris(2-carboxyethyl)phosphine (TCEP) at pH 8.0. The whole supernatant was alkylated for 45 min in the dark with 10 µL of 400 mM of iodoacetamide for a final concentration of 40 mM. Samples were dialyzed for 6h in dialysis units at 3500 Da (THERMO SCIENTIFIC-Slide-A-Lyzer 3.5K MWCO MINI Dialysis) with 100 mM Ambic (ammonium bicarbonate) at pH 8.0 (two changes).

Enzymatic digestion and purification: The whole dialyzed sample was digested overnight with 1µg of trypsin at 37°C. After about 16h, the samples were acidified with 1% formic acid (FA) and the proteins are extracted and purified by solid phase extraction with EMPORE SPE Extraction Disk (3M). The disks (Ø 0.1 cm) were washed with acetonitrile (1 min), conditioned with methanol (1 min) and washed with 0.1% FA solution (1 min) before being added to the samples and mixed for three hours to allow protein loading on the disk. Following a brief wash of the disk in 0.1% FA solution (1 min), the peptide mixtures were then eluted in 100 µL of 75:25 (v/v) acetonitrile:0.1% FA. All samples were then dried down on speedvac and resuspended in 10 µL of 0.1% FA.

Protein analysis by nanoLC-Orbitrap MS/MS: The textile samples were injected without further dilution; the injection volume was 1 µL. Samples were run in duplicates. The peptides were first loaded onto an in-house packed THERMO BioBasic C18 precolumn (30 mm x 75 µm i.d.) after which they were separated on an in-house packed analytical column (210 mm x 75 µm i.d.) made of the same stationary phase, using a THERMO SCIENTIFIC DIONEX Ultimate 3000 UHPLC system with the following gradient: 2% B 0-8 min, 55% B 98 min, 90% B 100-103 min, 2% B 104-120 min, where buffer A is 0.1% FA in H_2O and buffer B is 0.1% FA in acetonitrile (ACN). The UHPLC was directly coupled to a THERMO SCIENTIFIC LTQ Orbitrap Velos mass spectrometer which analyzed the peptides in positive mode using the following parameters: MS1 60,000 resolution, 100 ms acquisition time, 1×10^6 automatic gain control (AGC), MS2 15,000 resolution, 250 ms acquisition time, 5×10^5 AGC, top 8, 30 normalized collision energy (NCE) higher-energy collisional dissociation (HCD).

Bioinformatics analysis: For each sample, the two fractions were combined into one search to create one output file. PEAKS 8.5 (BIOINFORMATICS SOLUTIONS INC.) was used to search the RAW data for matches against publicly available sequences of mammals, birds and fish species in imported UniProt (www.uniprot.org) and NCBI (https://www.ncbi.nlm.nih.gov/protein) databases. Searches were carried out using trypsin as enzyme, one allowed non-trypsin cleavage at any end, one missed cleavage, peptide mass tolerance (PMS) of 10-15 ppm, fragment mass error tolerance (MS/MS) of 0.02 Da, carbamidomethylation as a fixed modification, and deamidated (NQ), hydroxylation (P), and oxidation (M) as variable modifications. PEAKS PTM was enabled to identify unspecific PTMs (see details for each category of samples).

Scanning electron microscopy - Energy dispersive X-ray spectrometry (SEM-EDS)

27 skin-based samples and 26 membrane-based samples were analyzed by SEM-EDS to characterize the morphology of the metal coating and the substrate support, and to determine the elemental composition of the metal.

The EDS mapping proves to be the most representative method to conduct elemental analysis on this target of samples. Once detected the main and minor elements present in the sample, a semi-quantitative analysis was attempted considering just the elements related to the metal composition, according to the distribution maps.

The accuracy of the semi-quantitative analysis is highly dependent on the thickness and homogeneity of the coatings [12]. Gilt/silvered animal-based threads are a complex target of samples and the major EDS analytical limit stems from the spatial non-homogeneity and the surface non-planarity of the samples under investigation; therefore, EDS measurements were conducted for purposes of comparison of data in the framework of the present study, as well as with other published data. The elements concentrations have to be considered as approximate values indicative of the original metal composition, instead of absolute values. Trying to achieve results as much representative as possible of the original composition of the coatings, different acquisition modes (maps and areas) were chosen and different approaches applied for the semi-quantitative analysis according to the type of sample. All acquisitions were conducted at 15kV in HITACHI S3700N in variable pressure equipped with a BRUKER 6 | 60 XFlash EDS detector. The elements associated to environmental contamination and/or the substrate composition were not included in the qualitative and semi-quantitative analysis of the metal coating composition by peak-background (PB) ZAF.

For 11 membrane-based samples, different area acquisitions were performed in potential gold areas and silver areas as inform by the map distribution of gold and silver on the surface of the sample, and the corresponding average concentrations were reported. The close inspection of the threads within the textiles or by HIROX microscope hinted to the presence of gilt-silver coatings (in different conservation state) on the membrane-based samples under investigation, thereby two layers, a gold on top of a silver one, were assumed. Area acquisitions at different voltage, 5kV and 15kV, were also performed on one sample, having a well-preserved metal coating, to explore compositional differences between the outer surface and deeper layers.

Despite the organic nature of the strip materials (adhesive ground and skin-based or membrane-based support), the EDS mapping provided useful information on the strips raw materials and treatments, thus, EDS mapping was performed on a selection of three cross-sections of skin-based samples to explore the elemental distribution within the sample stratigraphy.

Table S1.B1. Analytical plan. Detail by analytical technique of the number of samples analyzed. OM=Optical microscopy; P= Proteomics; SEM-EDS= Scanning electron microscopy - Energy dispersive X-ray spectrometry; SEM-µXRF= Scanning electron microscopy - micro-XRF spectrometry.

Skin-based metal threads: 51 samples								Membrane-based metal threads: 40 samples			
Flat strips: 9 samples				Wrapped strips: 42 samples							
OM	P	SEM-EDS	SEM-µXRF	OM	P	SEM-EDS	SEM-µXRF	OM	P	SEM-EDS	SEM-µXRF
7	9	9	-	21	37	18	9	20	36	26	8

BIBLIOGRAPHY

1 Vial, G. Les vêtements liturgiques dits de Saint Valère. Étude technique de pseudo-lampas à fond (ou effet) double-étoffe. *Techniques & culture* **34**, 67-81 (2000).

2 Flanagan, J. F. Early Silk Weaves. *The Burlington Magazine for Connoisseurs* **65**, 133-135 (1934).

3 Elsberg, H. A. & Guest, R. Another Silk Fabric Woven at Baghdad. *The Burlington Magazine for Connoisseurs* **64**, 271-272 (1934).

4 Shepherd, D. G. A Dated Hispano-Islamic Silk. *Ars Orientalis* **2**, 373-382 (1957).

5 Shepherd, D. G. The Hispano-Islamic textiles. *Chronicle of the Museum for the Arts of Decoration of the Cooper Union* **1**, 357-401 (1943).

6 Desrosiers, S., Cornu, G., Huchard, V. & Valantin, F. *Soieries et autres textiles de l'Antiquité au XVI siècle. Catalogue du Musée National du Moyen Âge, Thermes de Cluny*. (Éditions de la Réunion des Musées Nationaux, 2004).

7 Breck, J. A Hispano- Moresque Textile fragment. *Metropolitan Museum of Art Bullettin* **24**, 253-254 (1929).

8 Otavsky, K. & Salīm, M. A. M. *Mittelalterliche Textilien I. Ägypten, Persien und Mesopotamien, Spanien und Nordafrika*. (Abegg-Stiftung, 1995).

9 Sandu, I. C. A. *et al.* Fluorescence recognition of proteinaceous binders in works of art by a novel integrated system of investigation. *Microsc. Res. Techniq.* **75**, 316-324 (2012).

10 Popowich, A. K., Cleland, T. P. & Solazzo, C. Characterization of membrane metal threads by proteomics and analysis of a 14th c. thread from an Italian textile. *J. Cult. Herit.* **33**, 10-17 (2018).

11 Krist, G., Kimmel, T., Pichler, B. & Delvai, E. *Medieval fabric fragments from the collection of the Universalmuseum Joanneum in Graz, Austria. Technological analysis and conservation*, University of Applied Arts, Vienna, (2017).

12 Hoke, E. & Petrascheck-Heim, I. Microprobe analysis of gilded silver threads from Mediaeval textiles. *Stud. Conserv.* **22**, 49-62 (1977).
