# Supplementary material for: Palaeoproteomics and microanalysis reveal techniques of production of animal-based metal threads in medieval textiles
Source: Sci Rep. 2024 Mar 4;14:5320. doi: 10.1038/s41598-024-54480-4 (PMC10912450; doi:10.1038/s41598-024-54480-4)
Supplement: Supplementary file 2 — Supplementary Information 2. [file 41598_2024_54480_MOESM2_ESM.pdf]

# SI-2_Technical examination

# Palaeoproteomics and microanalysis reveal techniques of production of animal-based metal threads in medieval textiles

**This PDF file includes:**

**Supplementary Text, Figures and Tables**

**Contents:**


**2.A Visual and technical characterization**

**2.B Strips stratigraphy in cross-section**

**2.C Strips thickness**


**List of figures:**


## 2.A

**Figure S2.A1.** Box plot of the measured width of animal-based strips.

## 2.B

**Figure S2.B1.** Membrane-based strips stratigraphy and morphology.

## 2.C

**Figure S2.C1.** Different thickness of the membranous strips.

**Figure S2.C2.** Animal-based strips thickness range by category of samples (membrane, wrapped and flat skin strips).


**List of tables:**


## 2.A

**Table S2.A1.** Skin-based wrapped metal threads' groups based on distinctive technical features.

**Table S2.A2.** Skin-based flat metal threads' groups based on the coating technique

## 2.B

**Table S2.B1.** Layering of skin-based wrapped strip samples in cross-section.

**Table S2.B2.** Layering of skin-based flat strip samples in cross-section.

## 2.A Visual and technical characterization

Upon the microscopic inspection of 45 textiles (out of 66 fragments) by portable digital microscopes (see methods) and 91 samples by a HIROX KH-8700 3D digital microscope and a LEICA M205C stereomicroscope, some technical and visual features were determined to be distinctive enough to define patterns of technology and preliminarily group the threads (**Table S2.A1** and **S2.A2**). In details:

(a) the metal coating and strip visual appearance,
(b) the winding of the strips around the core or the use of them as flat strips.

Then, in filé metal threads (strips wound around the fibrous core [1]):

(c) the strip twist direction,
(d) the fibrous core type and construction.

Among characteristic measurements (thread diameter, number of coils per unit length and strip width) only the width values (and although they were highly variable along the same thread and same strip sample) were considered for comparison purposes between wrapped and flat strips, and membrane-based and skin-based strips (**Figure S2.A1**). Wrapped strips appeared often stretched or overlapped, and showed a non-planar geometry induced by the spinning and winding process itself, thereby the width values measured were found to be more variable comparing to flat strips. Taking into consideration that the original width of the strips might have been altered by the natural aging and damage of the skin or membrane substrates, the highly diverse measurements might also suggest that the strips were cut manually.

**Table S2.A1 Skin-based wrapped metal threads' groups based on distinctive technical features.** The twist direction of the strips, the core type and construction allowed to bracket the skin-based wrapped threads in groups, which showed interesting analogies in objects of the same provenance. An example for each group identified is reported, the attribution (origin and date) of the textiles correspond to museum's assignments. Details of the technical characterization of the strips for all the objects here investigated are shown in **Table 3**. Photos by Cristina Scibè © Museum Conservation Institute, Smithsonian Institution. Photo of the object 1902-1-273a courtesy of Kira Eng-Wilmot © Cooper Hewitt, Smithsonian Design Museum.

| **Strip Twist** | **Z** | | **S** | | |
|---|---|---|---|---|---|
| **Fibrous core** | Silk | Silk | Plant fibers. Linen? | Plant fibers. Linen? | Silk |
| **Twist / #yarns** | Z/single-ply | Z/single-ply | S/3-ply | S/2-ply | S/2-ply |
| **Fibers color** | Tan | Yellow | Ecru | Ecru | Pale yellow |
| **Detail of the weave of representative textiles** | 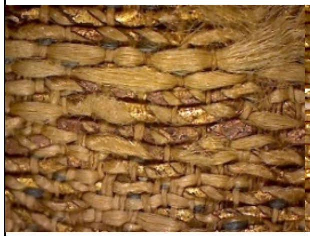 *1902-1-977c (obverse)* | 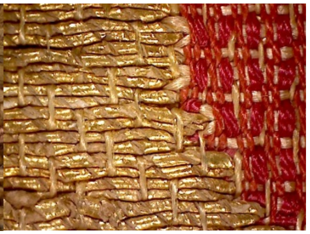 *1902-1-385 (reverse)* | 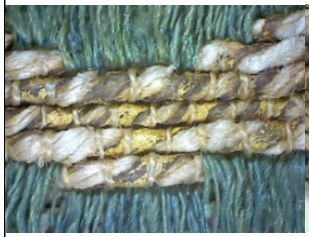 *1902-1-292a (obverse)* | 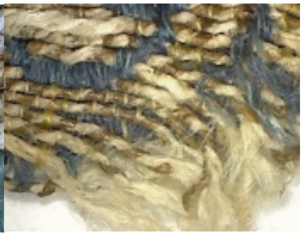 *1902-1-262* (obverse)* | 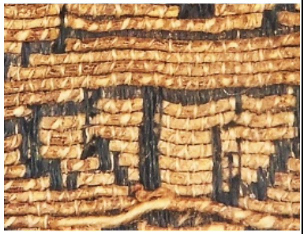 *1902-1-273a** (obverse)* |
| **Attribution** | **Spain 13th c.** | **Iran (possibly Tabriz) possibly 13th c.** | **Italy 14th c.** | **Italy 14th c.** | **Italy14th c.** |

*Wardwell [2]: S and Z gilded leather strips. A similar fragment in the Cleveland Museum of Art (https://www.clevelandart.org/art/1939.44) indeed has wrapped strips in both twist directions. Indictor [3], who also examined the fragment of the Cleveland Museum of Art, reports S and Z twisted leather strips and a 2-ply undyed linen core. However, the textile here investigated has just S-twisted wrapped strips.
** Indictor [3]: S twisted leather or membrane strip and a Z-spun tan silk core (no indication of the number of yarns); Wardwell [2]: silk core.

**Table S2.A2. Skin-based flat metal threads' groups based on the coating technique.** An example for each coating technique identified is reported, the attribution (origin and date) of the textiles correspond to museum's assignments. HIROX images by Caroline Solazzo © Museum Conservation Institute, Smithsonian Institution.

| **Coating technique** | **One-side coated** | | | **Double-side coated** |
|---|---|---|---|---|
| | **Gold coating** | **Gold-alloy coating** | **Yellow top coating + Silver coating** | **Gold coating (front) Gilt-silver coating (back)** |
| **Strip front side** HIROX image | 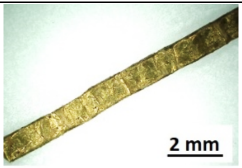 | 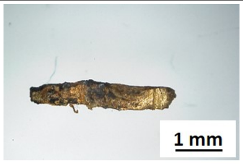 | 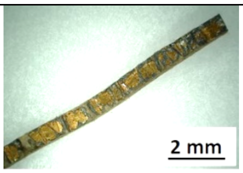 | 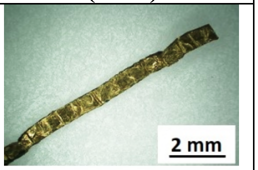 |
| **Strip back side** HIROX image | 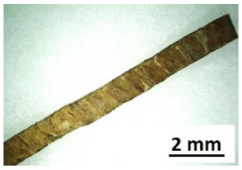 | 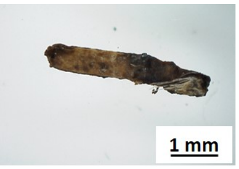 | 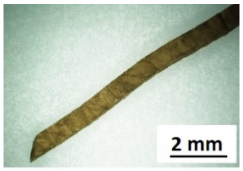 | 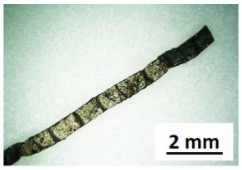 |
| | *1862:16 I* | *1902-1-233* | *1862:16 V* | *1862:16 IV* |
| **Attribution** | **Central Asia/Northern China 1st half 14th** | **Spain or Iran 14th** | **Central Asia/Northern China 1st half 14th** | **Central Asia/Northern China 1st half 14th** |

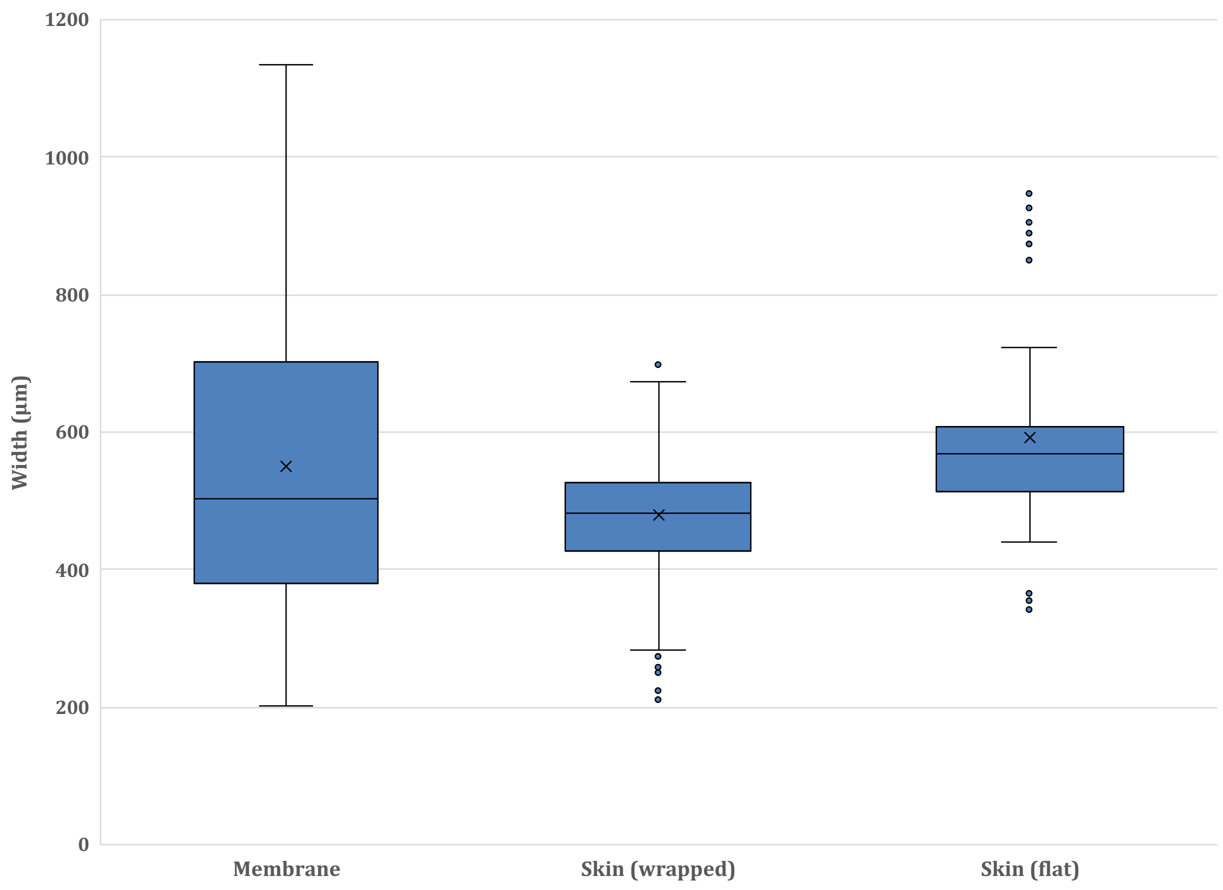


**Figure S2.A1. Box plot of the measured width of animal-based strips.** Box plot showing the range of measured strip width values for each category of samples: membrane threads, skin wrapped threads and skin flat strips (one to eleven measurements per fragment, and one to eight measurements per sample).

## 2.B Strips stratigraphy in cross-section

**Membrane-based metal threads**

Twenty cross-sections were prepared from membrane-based metal thread samples; five of them (**5963, 5798, I I161, 3RU8457_1** and **3RU8457_2**) were complete thread samples (including the wrapping strip and the fibrous core), the other 15 were strip samples (wrapped strip alone), thus did not include the fibrous core. In details, according to the textile geographical attribution assigned by each museum, the samples embedded and studied were:

- 1 from a Middle Eastern object;
- 4 from Spanish objects;
- 13 from Italian objects;
- 2 from an Italian or Flemish object.

In **Figure S2.B1** are showed selected examples of membranous strips' stratigraphy. Sample **1902-1-257d_3** (**Figure S2.B1a**) showed the morphology and bright white fluorescence most commonly found in the membrane strips analyzed by fluorescence microscopy. A different stratigraphy and morphology were found on sample **1938-84-1_2** (**Figure S2.B1b**) and sample **5798** (**Figure S2.B1c**). In sample **1938-84-1_2**, only the substrate layer was visible, as the threads presented an advanced state of degradation with an almost complete loss of the metal coating within the whole textile fragment, already observable upon close inspection of the threads. Moreover, the uneven brown color of the substrate might be indicative of an advanced state of degradation of the membrane, presumably due to an improper treatment of the raw material (i.e. an improper cleaning of the membrane that could made it vulnerable to biological damage, or the presence of iron oxides in the salt used to remove blood traces in the membrane, which might have contributed to its brownish color).

The overlapping of the strips around the fiber core is perfectly represented in sample **5798,** where two strips joined together were clearly observable, one wrapped on top of the other. Moreover, a different morphology and fluorescence of the two strips was appreciated: while the inner one (10-20 μm) appeared similar to all the other strips examined in cross-section, the outer strip, notably thicker (20-65), showed a lower fluorescence, a grey-bluish color, and a less ordered structure. The different morphology might be associated to the type of membrane tissue used for making the strips. In the absence of metal traces between the two strips (in correspondence of the joining area), it is assumed that the strips might have been joined first to reach the desired length, and then coated with metal. The white fluorescence appreciable between the two strips (see white areas marked in **Figure S2.B1c**), might hint at the presence of an adhesive to join and keep the two strips wrapped.

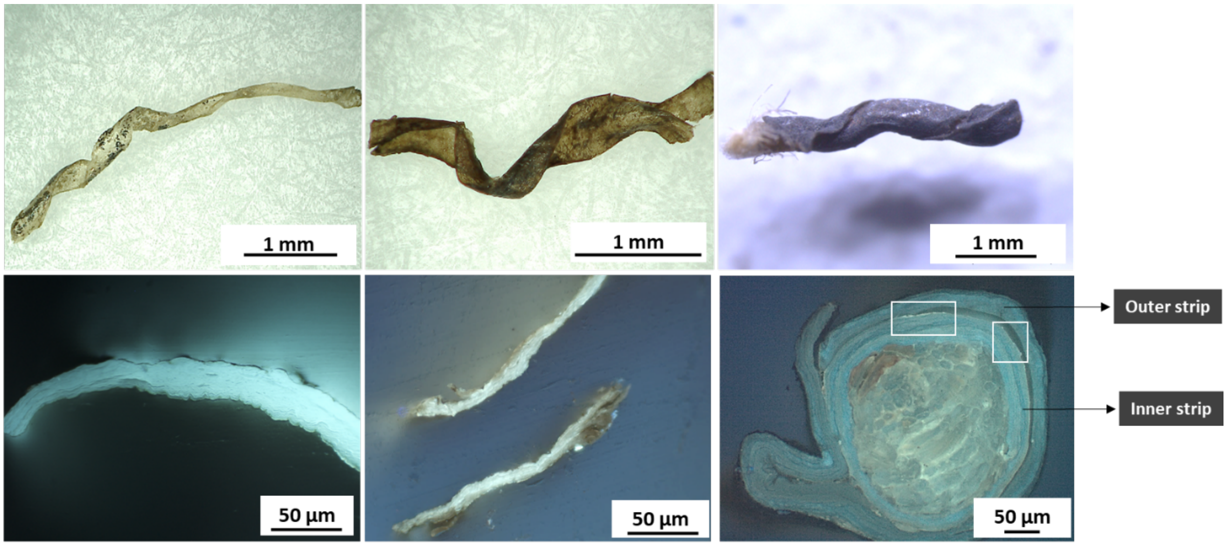


(a) (b) (c)

**Figure S2.B1. Membrane-based strips stratigraphy and morphology:** a) Sample **1902-1-257d_3:** (top) HIROX image (scale bar 1mm), (bottom) fluorescence cross-sectional image (OM2, UV reflected light, 50x, scale bar 50 µm); b) Sample **1938-84-1_2:** (top) HIROX image (scale bar 1mm), (bottom) fluorescence cross-sectional image (OM2, UV reflected light, 50x, scale bar 50 µm). Micrographs by Cristina Scibè © Museum Conservation Institute, Smithsonian Institution; c) Sample **5798: (**top) stereomicroscope image (scale bar 1 mm), (bottom) fluorescence cross-sectional image (OM1, UV reflected light, 10x, scale bar 50 µm). Micrographs by Cristina Scibè © Opificio delle Pietre Dure.

The morphology of the strips observed in cross-section was compared with histological images from animal membrane tissues samples, available in literature (Veterinary Histology – Open Textbook (pressbooks.pub)) [4,5], as well as in open source Atlas of Histology (the SIU, Southern Illinois University, Welcome to Histology at SIU; the Vigo University Atlas of plant and animal histology, mmegias.webs.uvigo.es/02-english/index.html; the University of Michigan Medical School, Virtual Slide List | histology (umich.edu); the University of Delaware, Color Images Home Page (udel.edu)). The main focus was on gastrointestinal organs, according to the mention in literature of gut membranes when membrane threads are described.

The gastrointestinal tract is essentially a tube extending from the oral cavity to the anus, structurally and functionally differentiated according to the function of each region: esophagus, stomach, small intestine (duodenum, jejunum, ileum) and large intestine (appendix, colon, rectum). It is organized into a series of four distinct layers which are fairly consistent throughout its length. The anatomy of the wall, which surrounds the lumen of these organs, consists of several layers also called tunicae (from inner to outer ones): mucosa, submucosa, muscularis externa or propria and serosa or adventitia. The four distinct layers differ in composition, morphology and thickness from one organ to another, according to the specific function of each organ [5].

The submucosa and muscularis externa layers of most of these organs showed the most similar morphology to our samples.

The submucosa is a tough layer composed of connective tissue (mainly collagen type I and III proteins) and smooth muscle proteins. Collagen fibrils in the submucosa are organized in thick fibres arranged in a criss-cross pattern. The muscularis externa or muscular layer is mainly composed of smooth and striated muscle and can be subdivided into two discrete layers according to the main direction of the muscular fibres. In the inner layer, the orientation is circumferential (circular arrangement); in the external layer it is axial (longitudinal direction parallel to the surface of the wall) [4].

Interesting analogies, which deserves further investigation, were found with stomach, esophagus and small intestine tract organs (duodenum and jejunum). The remarkably different morphology and thickness showed by the outer strip of sample **5798**, was found to be similar to esophageal wall tissues (histologyslides.med.umich.edu/Histology/Digestive System/Pharynx Esophagus and Stomach/153_HISTO_20X.htm; Animal organs. Digestive system. Esophagus. Atlas of plant and animal histology. (uvigo.es)) [4].

**Skin-based metal threads**

Twenty-eight cross-sections were prepared from skin-based metal thread samples, 21 from skin-based wrapped threads and seven from skin-based flat threads.

Among the wrapped strips examples, six of them (**1902-1-977c_3, 313, 164, I A7_2, 1902-1-271a_3**, and **D12b**) consisted of complete threads (strips + fibrous core), while the others were strip samples without the core.

In details, according to the textile geographical attribution assigned by each museum, the samples embedded and studied were:

- 10 from Spanish objects (one of them considered as possibly Spanish, **1902-1-251**, and one as Spanish or Iranian, **1902-1-233**);
- 8 from Italian objects;
- 4 from Middle Eastern objects (one of them possibly from Tabriz, **1902-1-385**);
- 6 from Asian objects (one of them from Far East, **D13a**, two from China, **P4c** and **d**, and the other three from Central Asia/Northern China)

Through the observation of leather in cross-sections by optical microscopy [6-10], it is possible to distinguish morphologically the different layers of the animal skin from the outer surface to the underlying muscles: papillary dermis or grain layer, reticular dermis or corium layer, and flesh layer. The grain layer is made by fine fiber bundles compactly weaved and it contains hairs, sebaceous and sweat glands and blood vessels; while the fiber bundles of the reticular layer are thick and dispersed uniformly forming a three-dimensional net structure; finally, in the flesh layer, fibers run in a horizontal plane and contains fat substances. It is known that in mature cattle the skins are generally thicker, with a thicker grain layer, while goat skins are characterized by a compactly weaved corium layer. In woolly sheep skins, there is a tendency to looseness at the junction of the corium and grain layer, and natural fat cells, stored in a layer in between the grain and corium layer, interrupt the fiber weave [6,7].

The skin substrates of the metal threads under investigation were possibly made from grain and/or corium skin layers. Nevertheless, no remarkable differences of the skin morphology were observed to be able to distinguish exactly which layers of the skin were used or which animal species. The dense plexus of collagen fibrous bundles, interwoven in different directions, running perpendicularly as well as parallel to the cross-section surface, allowed to unquestionably distinguish skin threads from the smooth and aligned structure of the membranous strips.

The fibers structure in wrapped strips was found to be more compactly waved in Spanish samples than in Italian and Middle Eastern samples, where it looked generally slightly looser. In flat strips, the fibers structure appeared more stratified than in wrapped strips. However, it was not possible to relate the different structure observed with the type of animal skin rather than to deterioration processes of the skin substrate itself. Indeed, in a few cases, a delamination of the fibrous weave was observable, either in wrapped and in flat strips (see samples **P4c, D13a** and **1862:16 V** in **Table S2.B2**). A remarkably different skin morphology was observed in samples **1902-1-233**, **1862:16 III** and **P4d**. The latter showed indeed a bright white fluorescence in correspondence of the central section of the substrate. The morphology shown by this central area, might indicate the use of skin layers including fat cells [6]. Similar morphology and fluorescence was shown by the central section of the skin substrate on sample **1902-1-233**, which, interestingly, was no more evident in a deeper slice of the cross-section, indicating that this area was not homogeneously present within the skin, as shown in **Table S2.B2**.

In **Table S2.B1** are shown the most representative examples of the stratigraphic sequence observable in skin-based wrapped strips, made by three layers: the metal coating, the adhesive layer and the skin substrate. An example, **313**, where the adhesive layer was not detected is also reported. **Table S2.B2** shows flat strips layering, mostly consisting in four layers: the metal coating, the adhesive layer, the skin substrate, and a second adhesive layer applied on the reverse of the strips, with the exception of sample **P4c**, where the adhesive layer (layer 4) was not clearly detected on the reverse of the strip. Nevertheless, its presence was assumed even in this sample, according to the visual appearance of the strip, and the not perfectly planar surface of the back of the strip in cross-section. All samples observed under HIROX 3D microscope showed a smooth and shiny appearance, resembling a finishing treatment, on the reverse surface of the strips, even when no metal coating was applied on that side. The adhesive layer on the reverse of the strips was often less preserved than the one under the metal coating, probably due to its exposure. Moreover, samples **1862:16 I** and **1862:16 V,** showed an intermediate layer (layer 3), in the middle of the skin substrate, resembling an adhesive layer according to its fluorescence under UV light. Additionally, in one of the two double-side coated samples embedded, **1862:16 III**, a back-side metal coating layer was observed, even if partly preserved (layer 5). On sample **1862:16 V**, where up to 7 layers were observed, the silver coating (layer 6) and a yellow top coating (not numbered) were barely visible in cross-section; indeed, the metal layer was just partially preserved in the sample embedded, and a top coat layer presence was assumed by the yellow-orange fluorescence slightly perceived under the resin surface (as the white arrow indicates in the corresponding sample image in **Table S2.B2**).

When clearly visible, the adhesive layers, showed little variance of brightness or hue (milky-white or milky-grey) in fluorescence, as shown in **Tables S2.B1** and **S2.B2**. A remarkable adhesive migration into the skin-substrate layer was observed in sample **D12b**, as well as in some flat strips (**D13a**, **P4c** and **P4d**), that might be due to the type of adhesive used.

Subsequent grinding/polishing cycles sometimes brought layers (as the adhesive one) or skin structures (as in sample **1902-1-233**) to the surface, barely visible or not detectable at all in upper slices. Furthermore, cross-sections of different samples from the same textile or object showed different stratigraphy and morphology, as for samples **1902-1-310_1** and **1902-1-310_2** (see **Table S2.B1**).

**Table S2.B1. Layering of skin-based wrapped strip samples in cross-section**. The layers are numbered (1-3) from outer to inner layer and localized in the micrographs. In white are metal layers, in black the adhesive layers and in red the skin substrates. The overall strips thickness is reported as range values; when the adhesive layer was measurable, the thickness range is reported as well. Fluorescence cross-sectional images were acquired (in UV reflected light) with two different microscopes in different laboratories (OM1 and OM2, see **SI-1.B Methods**), thus, for comparison purposes, images at same or comparable magnification are shown. OM=Optical Microscope. Micrographs by Cristina Scibè © Opificio delle Pietre Dure (OM1)/ Museum Conservation Institute, Smithsonian Institution (OM2)

| 313 | 1943-20-1b_2 | 1902-1-310_1 | 1902-1-310_2 |
|---|---|---|---|
| *Overall strip: 10-20µm*<br>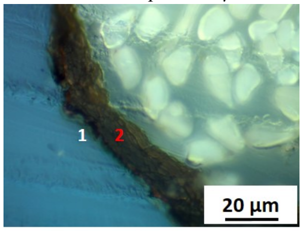<br><br>OM1, UV, 50x, scalebar 20µm<br>1. Metal coating: powder?<br>[Adhesive: not detectable]<br>2. Skin substrate (blackish color): tanned leather<br>[Inner fibrous core present: tan silk fibers] | *Overall strip: 30-35µm*<br>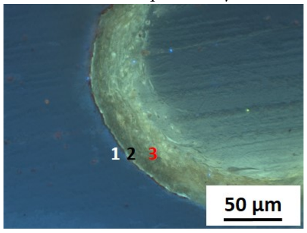<br><br>OM2, UV, 50x, scalebar 50µm<br>1. Metal coating: leaf<br>2. Adhesive (bright milky-white fluorescence): protein-based binder<br>3. Skin substrate: tanned leather | *Overall strip: 95-150µm*<br>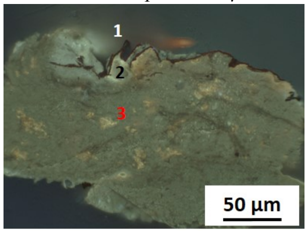<br><br>OM2, UV, 50x, scalebar 50µm<br>1. Metal coating: powder<br>2. Adhesive (partly preserved, milky-grey fluorescence): protein-based binder<br>3. Skin substrate: tanned leather | *Overall strip: 100-125µm*<br>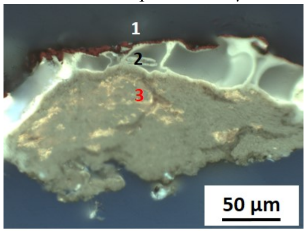<br><br>OM2, UV, 50x, scalebar 50µm<br>1. Metal coating: powder<br>2. Adhesive (bright milky-white fluorescence, *15-40µm*): protein-based binder<br>3. Skin substrate *(55-95µm)*: tanned leather |
| **D12b** | **1902-1-251_1** | **1902-1-262_1** | **1902-1-292b** |
| *Overall strip: 35-45µm*<br>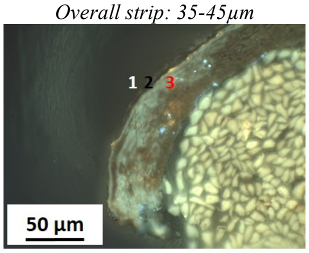<br><br>OM2, UV, 50x, scalebar 50µm<br>1.Metal coating: leaf<br>2. Adhesive (milky-grey fluorescence, *3-3.5µm*): protein-based binder<br>3. Skin substrate: tanned leather (with adhesive migration)<br>[Inner fibrous core present: yellow silk fibers] | *Overall strip: 40-50µm*<br>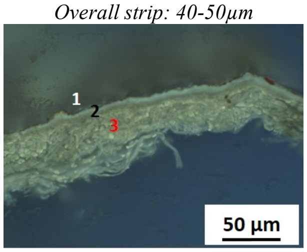<br><br>OM2, UV, 50x, scalebar 50µm<br>1. Metal coating: leaf<br>2. Adhesive (milky-grey fluorescence, *3-5µm*): protein-based binder<br>3. Skin substrate: tanned leather | *Overall strip: 40-60µm*<br>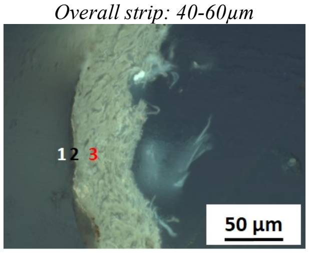<br><br>OM2, UV, 50x, scalebar 50µm<br>1. Metal coating: leaf<br>2. Adhesive?: barely detectable<br>3. Skin substrate: tanned leather | *Overall strip: 95-110µm*<br>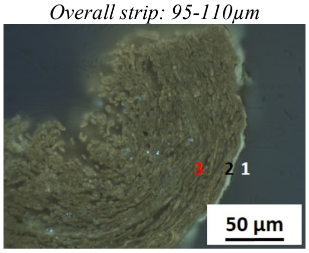<br><br>OM2, UV, 50x, scalebar 50µm<br>1. Metal coating: leaf<br>2. Adhesive (bright milky-white fluorescence, *2.5-5.5µm*): protein-based binder<br>3. Skin substrate: tanned leather |

**Table S2.B2. Layering of skin-based flat strip samples in cross-section.** The layers are numbered (1-6) from top to bottom of the image and localized in the micrographs. In white are metal layers, in black the adhesive layers and in red the skin substrates. The overall strips thickness is reported as range values; when the adhesive layers and the skin substrates were separately measured, the thickness range are reported as well. Fluorescence cross-sectional images were acquired with OM2 microscope in UV reflected light (see **SI-1.B Methods**), thus, for comparison purpose, images at same magnification are shown. OM=Optical Microscope. Micrographs by Cristina Scibè © Museum Conservation Institute, Smithsonian Institution (OM2)

| 1862:16 I | 1862:16 V | 1862:16 III | P4d |
|---|---|---|---|
| *Overall strip: 165-210µm* | *Overall strip: 265-290µm* | *Overall strip: 105-150µm* | *Overall strip: 110-130µm* |
| 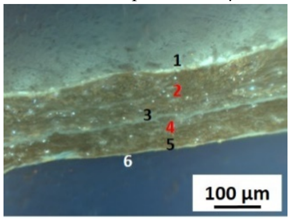<br>OM2, UV, 20x, scalebar 100µm | 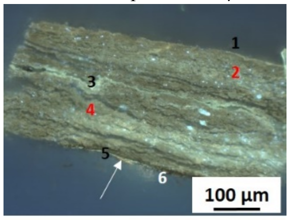<br>OM2, UV, 20x, scalebar 100µm | 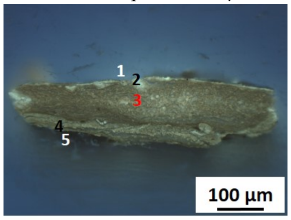<br>OM2, UV, 20x, scalebar 100µm | 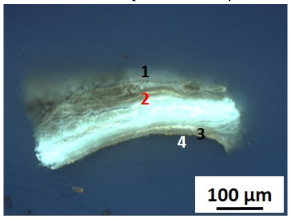<br>OM2, UV, 20x, scalebar 100µm |
| 1. Adhesive (bright milky-white fluorescence): protein-based binder<br>2. Skin substrate ($1^{st}$ strip?, *95-100µm*): tanned leather<br>3. Adhesive? (middle layer, milky-grey fluorescence, *4.5-5µm*): protein-based binder?<br>4. Skin substrate ($2^{nd}$ strip?, *55-65µm*): tanned leather<br>5. Adhesive (bright milky-white fluorescence, *5.5-8.5µm*): protein-based binder<br>6. Metal coating: leaf | 1. Adhesive (partly preserved, milky-white fluorescence, *2-4.5µm*): protein-based binder<br>2. Skin substrate ($1^{st}$ strip?, *100-110µm*): tanned leather<br>3. Adhesive? (middle layer, bright milky-white fluorescence, *4-8µm*): protein-based binder?<br>4. Skin substrate ($2^{nd}$ strip?, *140-165µm*): tanned leather<br>5. Adhesive (bright milky-white fluorescence, *4-4.5µm*): protein-based binder<br>6. Metal coating (partly preserved): barely visible<br>[The white arrow shows the top coating (yellow-orange fluorescence) barely visible] | 1. Metal coating – front side (partly preserved): leaf<br>2. Adhesive (bright milky-white fluorescence, *3-5µm*): protein-based binder<br>3. Skin substrate (*105-150µm*): tanned leather<br>4. Adhesive (partly preserved, milky-white fluorescence): protein-based binder<br>5. Metal coating – back side (partly preserved): leaf | 1. Adhesive (partly preserved, milky-grey fluorescence): protein-based binder<br>2. Skin substrate (*105-125µm*): tanned leather (with adhesive migration)<br>3. Adhesive (milky-grey fluorescence, *4.5-6.5µm*): protein-based binder<br>4. Metal coating: leaf |
| **P4c** | **D13a** | **1902-1-233** | |
| *Overall strip: 70-85µm* | *Overall strip: 70-100µm* | *Overall strip: 85-100µm* | |
| 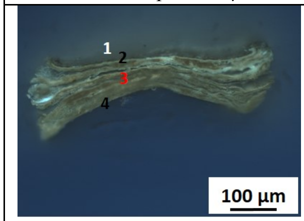<br>OM2, UV, 20x, scalebar 100µm | 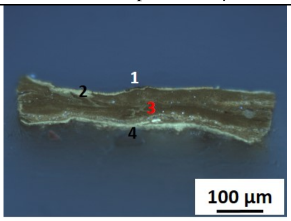<br>OM2, UV, 20x, scalebar 100µm | 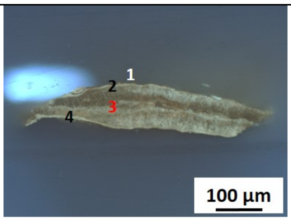<br>OM2, UV, 20x, scalebar 100µm, $1^{st}$ grinding | 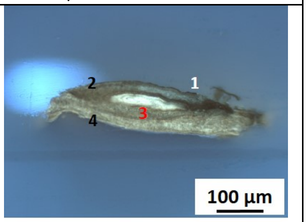<br>OM2, UV, 20x, scalebar 100µm, $2^{nd}$ grinding |
| 1. Metal coating: leaf<br>2. Adhesive (milky-grey fluorescence, *4-6µm*): protein-based binder<br>3. Skin substrate (*65-85µm*): tanned leather (with adhesive migration)<br>4. Adhesive: not clearly detectable | 1. Metal coating (partly preserved): leaf<br>2. Adhesive (bright milky-white fluorescence, *6-6.5µm*): protein-based binder<br>3. Skin substrate (*70-100µm*): tanned leather (with adhesive migration)<br>4. Adhesive (bright milky-white fluorescence): protein-based binder | 1. Metal coating: leaf<br>2. Adhesive (milky-grey fluorescence): protein-based binder<br>3. Skin substrate: tanned leather<br>4. Adhesive (milky-grey fluorescence): protein-based binder | 1. Metal coating (partly preserved): leaf<br>2. Adhesive (milky-grey fluorescence, *2.5-7µm*): protein-based binder<br>3. Skin substrate (*75-95µm*): tanned leather<br>4. Adhesive (milky-grey fluorescence, *2-5µm*): protein-based binder |

## 2.C Strips thickness

The membrane strips' thickness measured in cross-section ranges from ultra-thin membranes in the order of 10 µm (**Figure S2.C1a**) or less to membranes up to three times that value (**Figure S2.C1b**). For sample **5798**, two outliers measurements were obtained above 60 µm for the outer strip. In a few samples coming from the same object (**1902-1-274a** and **1902-1-274b**) or fragment (**1902-1-279_1** and **1902-1-279_2),** as **Figures S2.C1c** and **d** show, different thickness values were obtained. This difference might indicate the use of different metal threads batches to decorate the textile. No correlation could be observed between the age of the samples and the thickness of the membranes.

(a) (b)

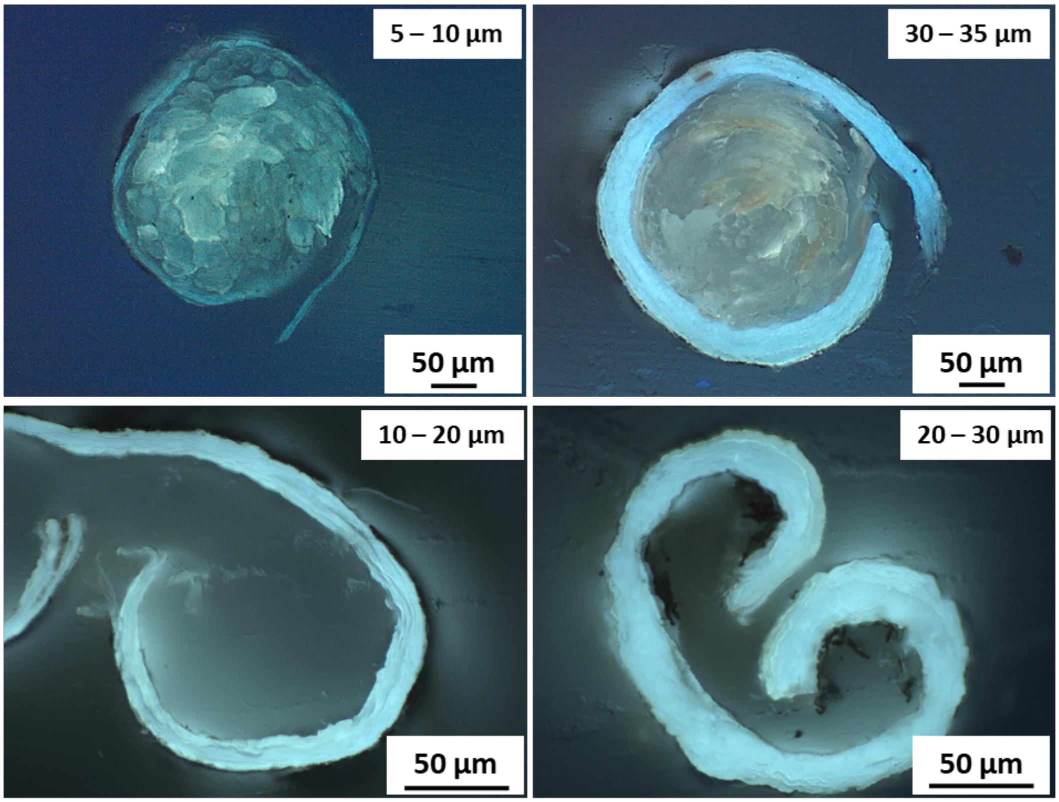


(c) (d)

**Figure S2.C1. Different thickness of the membranous strips.** Fluorescence cross-sectional images (scale bar 50 µm): **a)** Example of ultra-thin membrane strip, sample **5963** (OM1, UV reflected light, 10x); **b)** Example of thick membrane strip sample **I I161** (OM1, UV reflected light, 10x). Micrographs by Cristina Scibè © Opificio delle Pietre Dure. Examples of membrane strips, sampled from the same textile fragment, showing different thickness: **c)** sample **1902-1-279_1** (OM2, UV reflected light, 50x)**; d)** sample **1902-1-279_2** (OM2, UV reflected light, 50x). Micrographs by Cristina Scibè © Museum Conservation Institute, Smithsonian Institution

The range of animal-based strips' thickness measured in cross-section is shown on **Figure S2.C2**. As expected, skin strips were much thicker than membrane strips. The thickness of skin strips was found to be quite variable within each cross-section and between samples, but was overall higher in flat strips than in wrapped strips. Spanish samples (goat leather) showed values ranging on average from 10 to 60 µm (with the exception of the two samples from object 1902-1-310, which showed a skin strip of 55-95 µm thickness in sample 2 and of 95-150 µm thickness in sample 1), while Middle Eastern/Italian samples (sheep leather) ranged from 25 to 130 µm. Interestingly, samples belonging to the same object (**1902-1-292a** and **1902-1-292b**), or similar fragments (**I A7** and **1902-1-385**) showed quite different thickness of the skin strip. The extreme thinness of the strip substrates compared to the known thickness of mammalian skins, led to suppose that they might be the product of splitting of the original skins (a manual delamination of skins to produce two supports), a very ancient practice largely used in Medieval Europe and Near-East in writing supports making, in order to optimize skins [11]. The skins substrates of flat strips were found to span from 70 to 150 µm, reaching up to 210 µm in sample **1862:16 I** and 290 µm in sample **1862:16 V**, where possibly two skins were coupled (**Table S2.B2**).

It is known that the thickness of the skins may be related to the animal species and the type of animal (i.e. goat skins and calf skins are thinner than sheep one; among sheep, higher quality skins are those from young animals and hair-sheep), and the skin area on the animal body (thicker and with a more compact and denser weave of fibers in the backbone, than in the belly region) [6]. Adhesive layers, when measured, were found to be from 2 to 6 µm thick, in wrapped strip samples. Among Spanish samples, an exception was represented by the two powder-coated samples from object **1902-1-310**, which showed the characteristic tridimensional profile of a powder coating, thus, the overlay of the metal flakes left open spaces between the metal and the skin layers, where the resin penetrated creating bubbles of air into the adhesive layer, that reached up to 40 µm thickness.

In flat strips, the adhesive layers were found to range from 2.5 to 8.5 µm thickness, in correspondence to the metal coating underneath layer (front of the strip); while on the reverse of the strips, where they were measured in just two samples (**1902-1-233** and **1862:16 V**) having more continuous layers, they were found to range from 2 to 5 µm thickness. The middle layer (within the skin substrate) resembling an adhesive layer, in samples **1862:16 I** and **1862:16 V**, was found to range from 4 to 8 µm thickness.

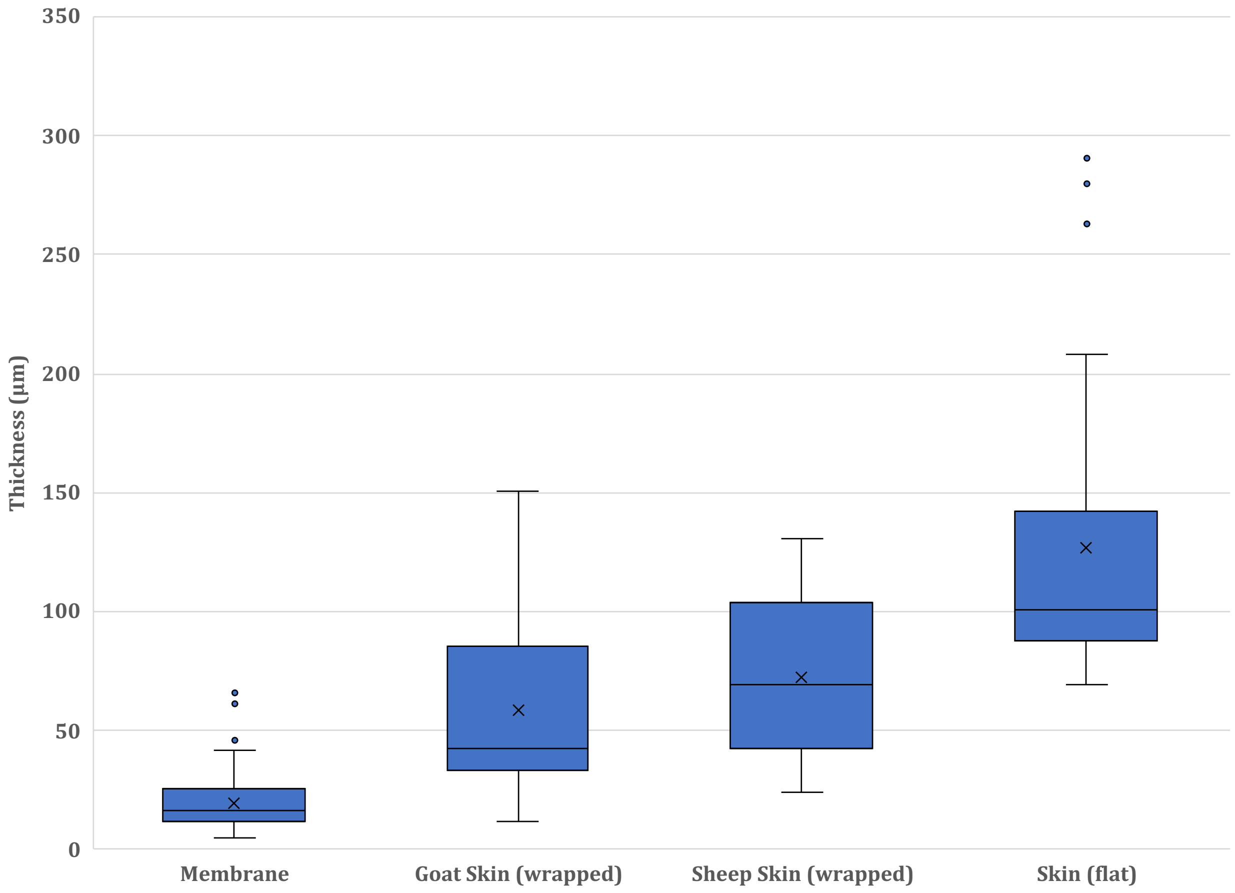


**Fig. S2.C2. Animal-based strips thickness range by category of samples (membrane, wrapped and flat skin strips).** For each sample the strips thickness was measured in cross-sectional images by *ImageJ* software. The outlier points in the flat skins belong to sample 1862:16 V, that is made of two skin strips.

## BIBLIOGRAPHY


1 Monnas, L. & Guelton, M.-H. VOCABULARY OF TECHNICAL TERMS ENGLISH with translations of the terms into French, German, Italian, Portuguese, Spanish and Swedish, Pages (CIETA, 2021).

2 Wardwell, A. E. Panni Tartarici: Eastern Islamic Silks Woven with gold and silver ($13^{th}$ and $14^{th}$ Centuries). *Islamic Art* **3**, 95-173 (1989).

3 Indictor, N., Koestler, R. J., Blair, C. & Wardwell, A. E. The evaluation of metal wrappings from Medieval textiles using scanning electron microscopy - energy dispersive X-ray spectrometry. *Text. Hist.* **19**, 3-22 (1988).

4 Natali, A. N., Carniel, E. L. & Gregersen, H. Biomechanical behaviour of oesophageal tissues: material and structural configuration, experimental data and constitutive analysis. *Med Eng Phys* **31**, 1056-1062 (2009).

5 Mohamed, A. A., Kadhim, K. H. & Hussein, D. M. Morphological and Histological study of the cecum and colon in adult local Camelus dromedarius. *Adv. Anim. Vet. Sci.* **6**, 286-291 (2018).

6 Haines, B. M. The fibre structure of leather in *Conservation of leather and related materials* (eds M. Kite & R. Thompson) 11-21 (Elsevier, 2006).

7 Haines, B. M. & Barlow, J. R. The anatomy of leather. *Journal of Materials Science* **10**, 525-538 (1975).

8 Liu, C.-K. Microscopic observations of leather looseness and its effects on mechanical properties. *Journal of the American Leather Chemists Association* **v. 104**, pp. 230-236-2009 v.2104 no.2007 (2009).

9 Zhang, H., Xia, Y., Shi, L. & Li, T. A novel technique for getting leather section image based on metallographic sample preparation. *JALCA* **108**, 166-170 (2013).

10 Zhang, H., Xia, Y., Shi, L. & Li, T. Characterization of leather structure via metallographic sample preparation. *JALCA* **109**, 1-7 (2013).

11 Couvrat Desvergnes, A. Skin Against Paper: Identification of Historical Interleaving Materials in Indo-Iranian Manuscripts. *The Book and Paper Group Annual* **34**, 130-139 (2015).
