# Supplementary material for: Palaeoproteomics and microanalysis reveal techniques of production of animal-based metal threads in medieval textiles
Source: Sci Rep. 2024 Mar 4;14:5320. doi: 10.1038/s41598-024-54480-4 (PMC10912450; doi:10.1038/s41598-024-54480-4)
Supplement: Supplementary file 3 — Supplementary Information 3. [file 41598_2024_54480_MOESM3_ESM.pdf]

SI-3_Proteomics

Palaeoproteomics and microanalysis reveal techniques of production of animal-based metal threads in medieval textiles

This PDF file includes:

Supplementary Text, Figures and Tables

Contents:

3.A Proteomics identification: Membrane-based metal threads
3.A.1. Bioinformatics strategy
3.A.2. Protein identification in the membrane metal threads
3.A.3. Peptidic markers in the membrane metal threads

3.B Proteomics identification: Skin-based metal threads
3.B.1. Bioinformatics strategy
3.B.2. Protein identification in the skin metal threads

3.C Calculation of deamidation

List of figures:

3.A

Figure S3.A1. Number of proteins found in textile membranes, references and a blank.

Figure S3.A2. Percentage of proteins identified in membrane samples, classified by protein families.

Figure S3.A3. Heatmap of the percentage coverage for collagen proteins in membrane samples.

Figure S3.A4. Frequency of identification of the different proteins for all categories.
S3.A4a. Smooth muscle proteins
S3.A4b. Actin-binding proteins
S3.A4c. Other cytoskeletal proteins
S3.A4d. Blood/plasma proteins
S3.A4e. Extracellular Matrix proteins
S3.A4f. Enzymes
S3.A4g. Other intracellular proteins

Figure S3.A5. Heatmap of the percentage coverage for key non-collagen proteins in membrane samples.

Figure S3.A6. Actin peptide marker.

3.B

Figure S3.B1. Percentage of proteins identified in each sample, classified by protein families.

Figure S3.B2. Egg white peptide markers in skin threads.

Figure S3.B3. Identification of the *Acipenser ruthenus* collagen chains.

Figure S3.B4. Sturgeon *de novo* peptide X.GPPGPQGTSGAPGAK.X (collagen type I alpha 1) in sample 1902-1-385_2.

Figure S3.B5. Sturgeon *de novo* peptide X.GVVGAPGAVGAPGK.X (collagen type I alpha 1) in sample 1902-1-272_1.

Figure S3.B6. Sturgeon *de novo* peptide X.GFTGLQGLPGSPGPHGEQGPAGASGPAGPR.X (collagen type I alpha 1) in sample 1902-1-233.

Figure S3.B7. Sturgeon *de novo* peptide X.GFPGTPGLPGLEGHR.X (collagen type I alpha 2) in sample 1862:16 I.

Figure S3.B8. Sturgeon *de novo* peptide X.GSPGEAGPSGPAGNR.X (collagen type I alpha 2) in sample 1862:16 I.

Figure S3.B9. Sturgeon *de novo* peptide X.GIAGDSGMPGPAGLR.X (collagen type I alpha 2) in sample D13a.

Figure S3.B10. Sturgeon *de novo* peptide X.GNPGAAGSQGPQGPAGPR.X (collagen type I alpha 2) in sample 1902-1-262_3.

Figure S3.B11. Sturgeon *de novo* peptide X.GEPGSGGAQGPLGPAGAR.X (collagen type I alpha 2) in sample 1902-1-285_1.

Figure S3.B12. Wheat peptide markers in 1862:16 V.

Figure S3.B13. Milk peptide markers in D13a.

Figure S3.B14. Human peptide markers in 1902-1-229b_1.

Figure S3.B15. Human peptide markers in P4d.

Figure S3.B16. Fungi peptide markers in P4d.

Figure S3.B17. Possible biological contamination on the surface of sample P4d.

Figure S3.B18. Horse collagen marker in sample 1862:16 III.

Figure S3.B19. a) White deposits on the object 1902-1-262. b) FTIR-ATR spectrum of the white deposits

3.C

Figure S3.C1. a) Deamidation of asparagine N in membrane threads. b) Deamidation of glutamine Q in membrane threads. Figure S3.B11b.

Figure S3.C2. a) Deamidation of asparagine N in skin threads. b) Deamidation of glutamine Q in skin threads.

Figure S3.C3. Percentage of N and Q with deamidation by sample categories

List of tables:

3.A

Table S3.A1. Actin peptides.

Table S3.A2. Other collagen peptide markers.

3.B

Table S3.B1. Identification of egg proteins in metal-wrapped skin threads.

Table S3.B2. Sturgeon species in Eurasia.

Table S3.B2. Milk peptides.

Table S3.B3. Homology of *Acipenser* collagens.

Table S3.B4. Alignment of the main Acipenser collagen I alpha 1 chains identified in skin samples.

Table S3.B5. Alignment of the main Acipenser collagen I alpha 2 chains identified in skin samples.

Table S3.B6. Human proteins identified in sample 1902-1-229b_1.

Table S3.B7. Human proteins identified in sample P4d.

Table S3.B8. Fungi proteins identified in sample P4d.

3.A Membrane-based metal threads

3.A.1. Bioinformatics strategy

For each sample, the two fractions were combined into one search to create one output file. PEAKS 8.5 (BIOINFORMATICS SOLUTIONS INC.) was used to search the RAW data for matches against publicly available sequences in imported UniProt (www.uniprot.org) and NCBI (https://www.ncbi.nlm.nih.gov/protein) databases.

Searches were carried out using trypsin as enzyme, one allowed non-trypsin cleavage at any end, one missed cleavage, peptide mass tolerance (PMS) of 15 ppm, fragment mass error tolerance (MS/MS) of 0.02 Da, carbamidomethylation as a fixed modification, and deamidated (NQ), hydroxylation (P), and oxidation (M) as variable modifications.

1- A general search was conducted against the reviewed Swiss-Prot **database UniProtKB/Swiss-Prot** (last imported on 23rd August 2021) in PEAKS PTM allowing a maximum of three post-translational modifications. This was done to search for non-membrane proteins, e.g. plant or animal adhesives of mammalian and non-mammalian nature. Data were filtered with a false positive rate (FDR) of 1%, a protein score -10lgP ≥ 50, and two unique peptides.

1- The search for the collagen species was conducted against **a database of all vertebrate collagen sequences available in NCBI** (last imported on 19th August 2021) in PEAKS PTM allowing a maximum of six post-translational modifications to account for the large quantity of proline modifications to hydroxyproline. This search allowed to identify the species of the membrane as cattle (*Bos taurus*), as well as the presence or not of collagen from another species that could be part of an adhesive or varnish. Data were filtered with a FDR of 1%, a protein score -10lgP ≥ 50, and zero unique peptide.

2- Finally, a restricted search was conducted against a **Uniprot Bovinae database** (imported on 20th November 2020) in PEAKS PTM allowing a maximum of seven post-translational modifications. Since *Bos taurus* was determined to be the source of the membrane, this search was conducted to obtain the list of membrane proteins (collagen and non-collagen) identified in the samples. Data were filtered with a FDR of 1%, a protein score -10lgP ≥ 20, a minimum of two peptides and one unique peptide.

3.A.2. Protein identification in the membrane metal threads

The proteins identified in the membrane samples were consistent with animal membrane proteins as observed in a pilot study [1], i.e. a range of collagen proteins, smooth muscle proteins (desmin, myosin, tropomyosin, etc.) and other intestinal membrane proteins. As in the pilot study [1] no adhesive proteins (e.g. milk, egg) or plant proteins were identified. Only in the blank sample was casein identified, likely due to laboratory contamination. **Figure S3.A1**. shows the number of proteins identified using the general *UniProtKB/Swiss-Prot database* in the textile membrane threads, as opposed to reference membrane samples of cow and cow coated with silver (for a description of the making of the replica samples see [1,2]). The break-down by sample shows that the textile membranes have at best (sample **FB 619_1** from 3RU8457) only half of the proteins identified in the reference membrane cow+silver. The number of proteins identified varies greatly among the textile membranes with the lowest identification for sample **1938-84-1_2**, below the blank. The sample is badly conserved and appears brown in cross-section with low fluorescence.

Samples taken from different areas of the textile can also exhibit very different results. This disparity could be due to a range of factors, some of them might be difficult to assess: state of preservation of the sample and area sampled (on more or less damaged edges of the textile), quality of the original membrane and processing, thickness, age of the animal and type of tissue from which the membrane thread is made of. In spite of the range of results, all samples' top match was one of the collagen chain from *Bos taurus* (collagen alpha 1 type I COL1A1, collagen alpha 2 type I COL1A2 or collagen alpha 1 type III COL3A1. The blank's top match was trypsin.

All 36 membrane samples were matched to *Bos taurus* when searched against the targeted *NCBI collagen database* from all vertebrates. The COL1A1, COL1A2 and COL3A1 peptide markers that allow differentiation from the domestic sheep (*Ovis aries*) and goat (*Capra hircus*), are shown in **SI_Collagen identification in Membranes.xlsx**). In addition, the samples had the COL1A2 peptide GIPGPVGA**A**GATGAR found in *B. taurus* (domestic cattle), *B. indicus* (zebu) and *B. javanicus* (banteng) but not the peptide GIPGPVGA**S**GATGAR found in *B. mutus* (wild yak), and the COL3A1 peptides GA**A**GPPGPPGSAGTPGLQGMPGER and GAPG**P**QGPPGAPGPLGIAGLTGAR found in *B. taurus*, *B. indicus* and *B. mutus* but not GA**V**GPPGPPGSAGTPGLQGMPGER and GAPG**S**QGPPGAPGPLGIAGLTGAR found in *B. javanicus*.

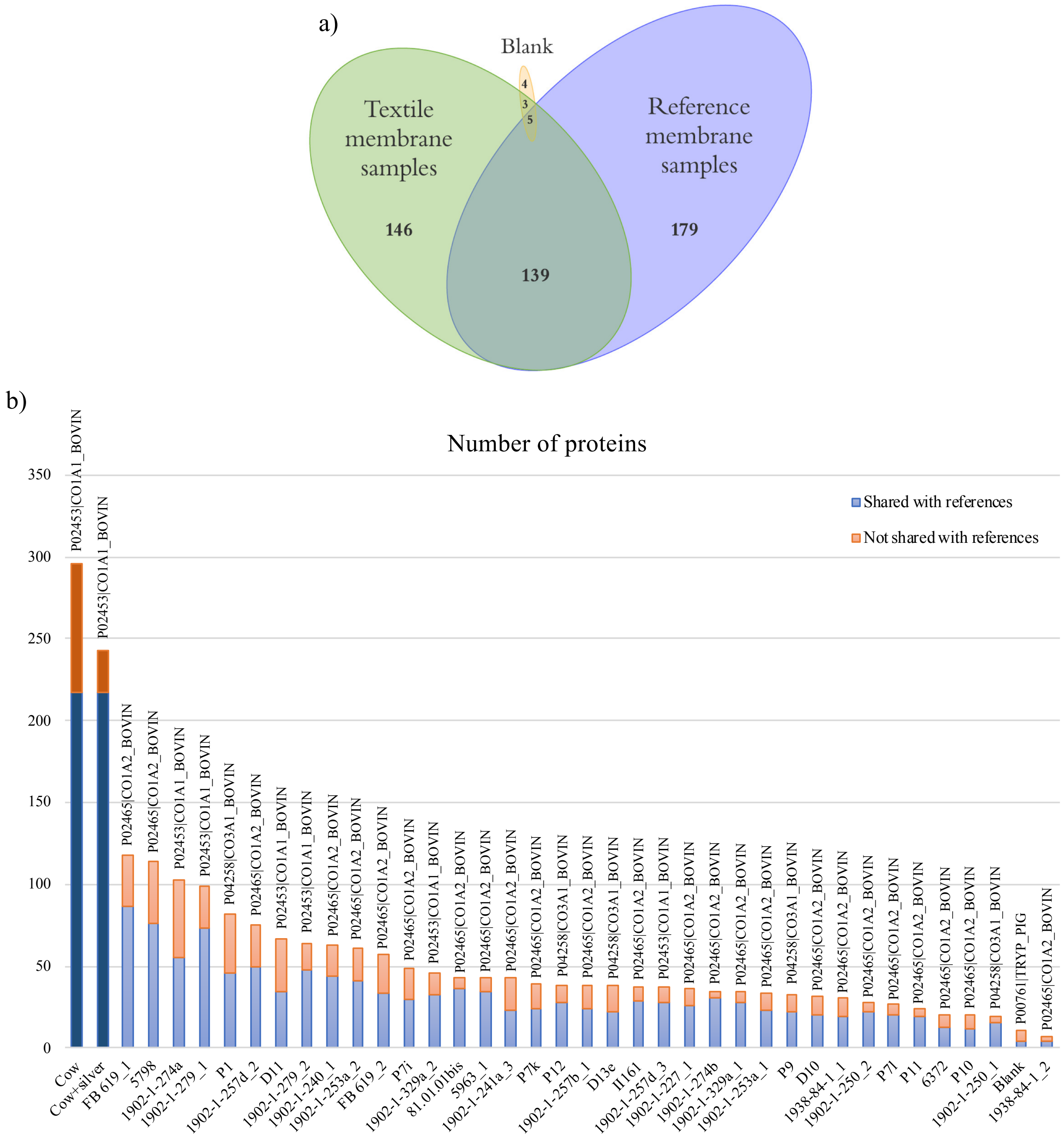

Figure S3.A1. Number of proteins found in textile membranes, references and a blank. a) Overall number of shared proteins (diagram realized with Lucidchart, lucidchart.com). b) Detail by sample of the number of proteins found in reference and textile membranes. Dark blue are proteins found in both reference samples, and in dark orange, proteins found in either of them. Above each sample is indicated the top match identified with the most Peptide-Spectrum Matches (PSMs) for each sample. With *UniProtKB/Swiss-Prot*, FDR 1%, protein score -10lgP ≥ 50, and two unique peptides.
FB 619-1 and 2 are from textile 3RU8457.

Using a *Bovinae-restricted Uniprot database*, with a FDR of 1%, a protein score -10lgP ≥ 20, a minimum of two peptides and one unique peptide, the number of proteins (excluding keratins) identified in the reference or membrane samples only (0%), and the number of proteins identified in both the references and the membrane samples (from 0 to 100% of the 36 membrane samples) indicate that a larger number of proteins (76) were found in the reference cow membrane only than in the ancient membrane samples only. The proteins only identified in the ancient samples (29) were found in less than 25% of the samples. Similarly, the majority of the proteins found in both reference and ancient membranes were found in less that 25% of the ancient samples, while only four proteins were found in over 75% of samples. These four proteins were actin (ACTG2 or ACTA2), desmin, myosin 11 and synemin, all proteins present in smooth muscles and thus representing reliable biomarkers of membrane metal threads.

R \ M	0	>0-25%	26-50%	51-75%	75-100%
0		29	0	0	0
>0-100%	76	45	12	3	4

The breakdown by protein families, excluding keratins, is given in **Figure S3.A2** for each sample. Compared to the even distribution of proteins in the modern reference samples, the ancient samples are dominated by the more robust structural proteins of collagen. Except for sample 1938-84-1_2, the ancient membrane samples had similar COL1A1 and COL1A2 protein coverages as reference samples, but slightly lower in the ancient samples for COL3A1. Smooth muscle proteins are present in all samples. Blood and plasma proteins are mostly present in samples from the 14th century onward, while other proteins (extra-cellular matrix, intracellular, cytoskeletal proteins, enzymes) are present in samples of all ages. Blood and plasma proteins might be more susceptible to degradation and could be an indicator of younger samples.

Figure S3.A3 details the identification of the collagen chains for each sample as a heatmap of the percentage coverage of the main collagen entries. The frequency of identification of the different proteins for all categories is shown in **Figures S3.A4a to g** as heatmaps of the percentage of reference or membrane samples in which the protein is identified. Same proteins identified under different accession numbers were grouped under one gene (GN) entry.

Figure S3.A5 details the identification of key smooth muscle proteins for each sample as a heatmap of the percentage coverage. Finally **Table S3.A1** gives the specific peptides allowing differentiation of the different actin proteins in *Bos taurus.* The spectra of ACTG2's specific peptide PEYDEAGPSIVHR (identified with a missed cleavage as WISKPEYDEAGPSIVHR) are shown in **Figure S3.A6** in the cow reference (**Figure S3.A6a**) and in sample P9 (**Figure S3.A6b**).

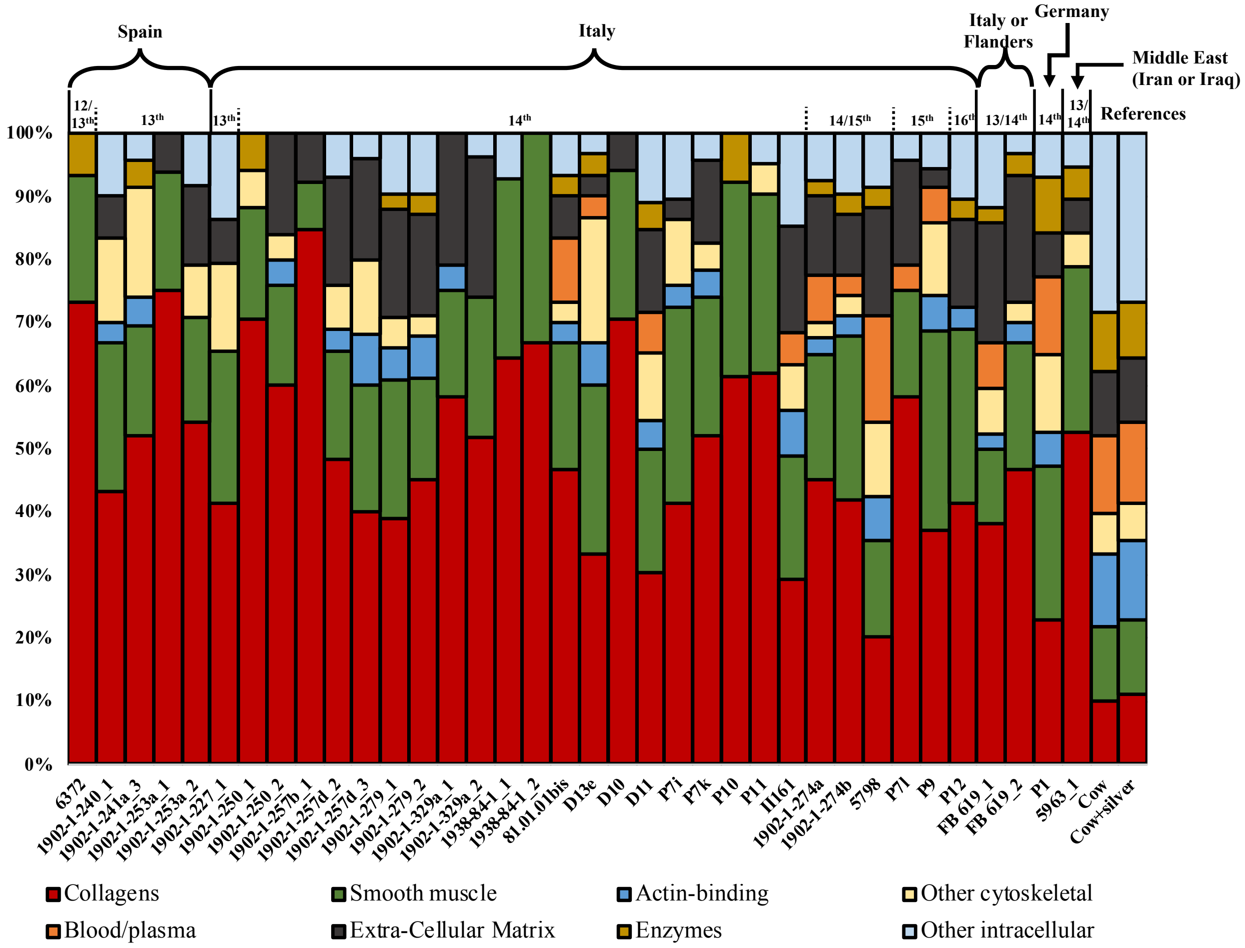

Figure S3.A2. Percentage of proteins identified in membrane samples, classified by protein families. With *Uniprot Bovinae* with a FDR of 1%, a protein score -10lgP ≥ 20, a minimum of two peptides and one unique peptide. Keratins are not included.
FB 619-1 and 2 are from textile 3RU8457.

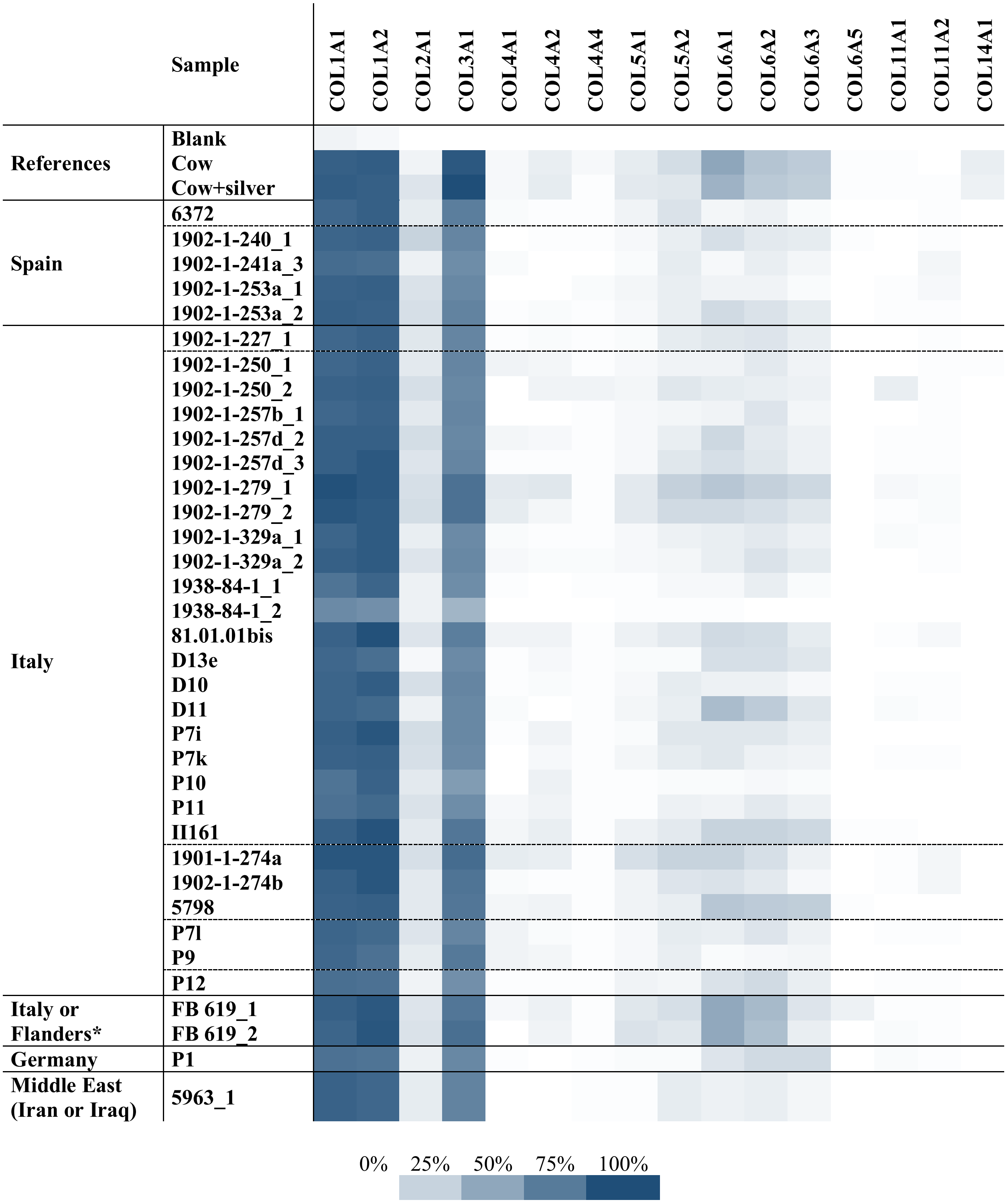

Figure S3.A3. Heatmap of the percentage coverage for collagen proteins in membrane samples (showing proteins with identification in a minimum of two samples). With *Uniprot Bovinae* with a FDR of 1%, a protein score -10lgP ≥ 20, a minimum of two peptides and zero unique peptide.
* from textile 3RU8457

(Next pages) **Figure S3.A4. Frequency of identification of the different proteins for all categories.** With *Uniprot Bovinae* with a FDR of 1%, a protein score -10lgP ≥ 20, a minimum of two peptides and one unique peptide.

0% 25% 50% 75% 100%

Figure S3.A4a. Smooth muscle proteins

GN	Description	Notes	%R	%M
ACTA2/ ACTG2	Actin aortic smooth muscle/Actin gamma-enteric smooth muscle	Cytoskeleton		
CNN1	Calponin-1	Cytoskeleton; Actin-binding		
DES	Desmin	Also in cardiac and skeletal muscles		
FLNA	Filamin A	Cytoskeleton; Actin-binding		
MYH11	Myosin 11	Cytoskeleton; Actin-binding		
MYL12B	Myosin regulatory light chain 12B	Cytoskeleton		
MYL6	Myosin light polypeptide 6	Cytoskeleton		
MYL9	Myosin regulatory light polypeptide 9	Cytoskeleton		
MYLK	Myosin light chain kinase smooth muscle	Cytoskeleton; Actin-binding		
PGM5	Phosphoglucomutase-like protein 5	Cytoskeleton		
SMTN	Smoothelin	Cytoskeleton; Actin-binding		
SYNM	Synemin	Cytoskeleton; Also in cardiac and skeletal muscles		
TAGLN	Transgelin	Cytoplasm; Actin-binding		
TPM1	Tropomyosin alpha-1 chain	Cytoskeleton; Actin-binding; Also in cardiac and skeletal muscles		
TPM2/ TPM4	Tropomyosin beta chain	Cytoskeleton; Actin-binding		

Figure S3.A4b. Actin-binding proteins

GN	Description	Notes	%R	%M
ACTN1	Alpha-actinin	Cytoskeleton		
ACTN3	Alpha-actinin-3	Cytoskeleton		
ACTN4	Alpha-actinin-4	Cytoskeleton		
CAP1	Adenylyl cyclase-associated protein	Cytoskeleton		
CFL1	Cofilin-1	Cytoskeleton		
CORO1C	Coronin	Cytoskeleton		
DBN1/PDLIM7	Drebrin 1/PDZ and LIM domain protein 7	Cytoskeleton		
DSTN	Destrin	Cytoskeleton		
FLNC	Filamin C	Cytoskeleton		
GSN	Gelsolin	Cytoskeleton		
MYH10	Myosin-10	Cytoskeleton; Non-muscle		
MYH14	Myosin heavy chain 14	Cytoskeleton; Non-muscle		
MYH9	Myosin heavy chain 9	Cytoskeleton; Non-muscle		
MYO1C	Unconventional myosin-Ic	Cytoskeleton		
PFN1	Profilin-1	Cytoskeleton		
PLEC	Plectin	Cytoskeleton		
SPTAN1	Spectrin alpha non-erythrocytic 1	Cytoskeleton		
SYNPO2	Synaptopodin 2	Cytoskeleton		
TLN1	Talin 1	Cytoskeleton		
VCL	Metavinculin/Vinculin	Cytoskeleton		

Figure S3.A4c. Other cytoskeletal proteins

GN	Description	Notes	%R	%M
ACTB/ACTG1	Actin cytoplasmic 1 and 2	Microfilaments; Non-muscle		
ACTBL2	Beta-actin-like protein 2	Microfilaments; Non-muscle		
GFAP	Glial fibrillary acidic protein	Intermediate filaments		
TUBA	Tubulin alpha chain	Microtubules		
TUBB	Tubulin beta chain	Microtubules		
VIM	Vimentin	Intermediate filaments		

Figure S3.A4d. Blood/plasma proteins

GN	Description	%R	%M
A1BG	Alpha-1B-glycoprotein		
A2M	Alpha-2-macroglobulin		
AHSG	Alpha-2-HS-glycoprotein		
ALB	Albumin		
APOA1	Apolipoprotein A-I		
C3	Complement C3		
FGA	Fibrinogen alpha chain		
FGB	Fibrinogen beta chain		
FGG	Fibrinogen gamma-B chain		
GC	Gc-globulin		
HBA	Hemoglobin subunit alpha		
HBB	Hemoglobin beta		
IGHM	Immunoglobulin heavy constant mu		
IGLL1	Immunoglobulin light chain		
SERPIND1	Serpin family D member 1		
TGFBI	Transforming growth factor-beta-induced protein ig-h3		

Figure S3.A4e. Extracellular Matrix proteins

GN	Description	Notes	%R	%M
ASPN	Asporin	Collagen-binding		
BGN	Biglycan			
DCN	Decorin	Collagen-binding		
DPT	Dermatopontin			
FBN1	Fibrillin-1			
FN1	Fibronectin			
HSPG2	Heparan sulfate proteoglycan 2	Basement membrane		
LAMA4	Laminin subunit alpha 4	Basement membrane		
LAMB1	Laminin subunit beta 1	Basement membrane		
LAMB2	Laminin subunit beta 2	Basement membrane		
LAMC1	Laminin subunit gamma 1	Basement membrane		
LGALS1	Galectin-1			
LUM	Lumican	Collagen-binding		
NID1	Nidogen 1 protein	Basement membrane; Collagen-binding		
NID2	Nidogen 2 protein	Basement membrane; Collagen-binding		
OGN	Mimecan			
POSTN	Periostin			
PRELP	Prolargin			
SFTPD	Pulmonary surfactant-associated protein D			

Figure S3.A4f. Enzymes

GN	Description	Notes	%R	%M
ALDH1A1	Retinal dehydrogenase 1	Oxidoreductase		
ALDH2	Aldehyde dehydrogenase mitochondrial	Oxidoreductase		
AOC3	Amine oxidase	Oxidoreductase		
ATP5B	ATP synthase subunit beta (Fragment)	Translocase		
ATP5F1B	ATP synthase subunit beta mitochondrial	Translocase		
CKB	Creatine kinase	Kinase, Transferase		
GAPDH	Glyceraldehyde-3-phosphate dehydrogenase	Oxidoreductase, Transferase		
GSTP1	Glutathione S-transferase P	Transferase		
IDH2	Isocitrate dehydrogenase [NADP] mitochondrial	Oxidoreductase		
ILK	Integrin-linked protein kinase	Kinase, Serine/threonine-protein kinase, Transferase		
LDHA	L-lactate dehydrogenase A chain	Oxidoreductase		
LDHB	L-lactate dehydrogenase B chain	Oxidoreductase		
MDH2	Malate dehydrogenase mitochondrial	Oxidoreductase		
PRSS1 (TRY1)	Cationic trypsin OS=Bos taurus	Serine protease		
PYGB	Alpha-1 4 glucan phosphorylase	Allosteric enzyme, Glycosyltransferase, Transferase		
TGM2	Protein-glutamine gamma-glutamyltransferase 2	Acyltransferase, Transferase		
TPI1	Triosephosphate isomerase	Isomerase, Lyase		

Figure S3.A4g. Other intracellular proteins

GN	Description	%R	%M
AHNAK	Neuroblast differentiation-associated protein		
ANXA2	Annexin A2		
ANXA5	Annexin A5		
ANXA6	Annexin A6		
ATP5F1A	ATP synthase subunit alpha mitochondrial		
CAVIN1	Caveolae associated protein 1		
CAVIN2	Caveolae associated protein 2		
CLTC	Clathrin heavy chain 1		
CSRP1	Cysteine and glycine-rich protein 1		
DPYSL2	Dihydropyrimidinase-related protein 2		
EEF1A1	Elongation factor 1-alpha		
EEF2	Elongation factor 2		
EHD2	EH-domain containing 2		
ENO1	2-phospho-D-glycerate hydro-lyase		
FERMT2	FERMT2 protein		
FHL1	Four and a half LIM domains 1		
GDI2	Rab GDP dissociation inhibitor beta		
H2A	Histone H2A		
H2B	Histone H2B		
H3	Histone H3		
H4	Histone		
HNRNPK	Heterogeneous nuclear ribonucleoprotein K		
HSPA1A/ HSPA1B	Heat shock 70 kDa protein 1A/1B		
HSPB1	Heat shock protein beta-1		
HSPB6	Heat shock protein beta-6		
HSPD1	60 kDa heat shock protein mitochondrial		
IQGAP1	IQ motif containing GTPase activating protein 1		
ITGA5	Integrin alpha-5		
ITGB1	Integrin beta-1		
LMNA	Lamin A/C		
NIBAN1	Niban apoptosis regulator 1		
PDLIM3	PDZ and LIM domain protein 3		
PKM	Pyruvate kinase		
PPIA	Peptidyl-prolyl cis-trans isomerase A		
PRDX2	Peroxiredoxin-2		
PRPH	Peripherin		
SORBS1	Sorbin and SH3 domain containing 1		
YWHAB	14-3-3 protein beta/alpha		
YWHAE	14-3-3 protein epsilon		
YWHAG	14-3-3 protein gamma		
YWHAZ	14-3-3 protein zeta/delta		

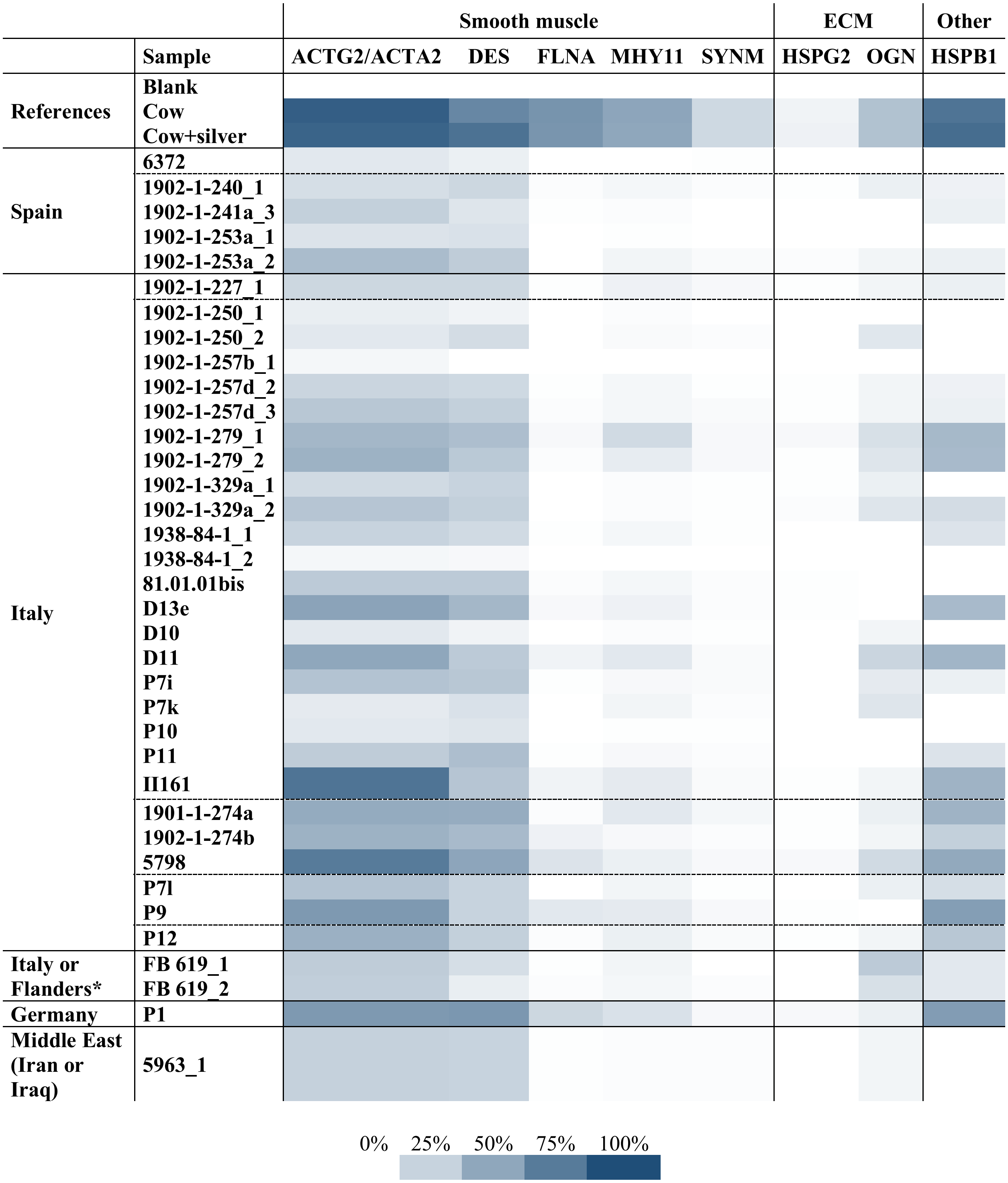

Figure S3.A5. Heatmap of the percentage coverage for key non-collagen proteins in membrane samples. With *Uniprot Bovinae* with a FDR of 1%, a protein score -10lgP ≥ 20, a minimum of two peptides and zero unique peptide. ACTG2/ACTA2= Actin aortic smooth muscle/Actin gamma-enteric smooth muscle; DES=Desmin; FLNA=Filamin A; MHY11=Myosin 11; SYNM=Synemin; HSPG2=Heparan sulfate proteoglycan 2; OGN=Mimecan; HSPB1= Heat shock protein beta-1.
* from textile 3RU8457

3.A.3. Peptidic markers in the membrane metal threads

The different actin proteins (alpha skeletal muscle, aortic smooth muscle, alpha cardiac muscle, and gamma-enteric smooth muscle) are mostly homologous but can be differentiated in *Bos taurus* by a few peptides. These peptides are shown in Table S3.A1 as well as their identification in the membrane metal threads.

Table S3.A1. Actin peptides. Peptides allowing differentiation of the different actin proteins (ACTA1, ACTA2, ACTC1 and ACTG2) in *Bos taurus*

ACTA1: P68138|ACTS_BOVIN Actin, alpha skeletal muscle;
ACTA2: P62739|ACTA_BOVIN Actin, aortic smooth muscle, Q58DT9|Q58DT9_BOVIN Alpha 2 actin, G8JKX4|G8JKX4_BOVIN Actin, aortic smooth muscle;
ACTC1: Q3ZC07|ACTC_BOVIN Actin, alpha cardiac muscle 1;
ACTG2: Q5E9B5|ACTH_BOVIN Actin, gamma-enteric smooth muscle

PROTEIN	SEQUENCE	REFERENCES	MEMBRANES
**ACTA1**	MCDEDETTALVCDNGSGLVK<br>DLYANNVMSGGTTMYPGIADR<br>YSVWIGGSILASLSTFQQMWITK		
**ACTA1**<br>**ACTC1**	IWHH**T**FYNELR*	Both	
**ACTA2**<br>**ACTG2**	IWHH**S**FYNELR<br>SFYNELR	Both<br>-	-<br>1902-1-274b; P9
**ACTA2**	M**C**EEE**DS**TALVCDNGSGLCK<br>**M**EE**DS**TALVCDNGSGLCK		
**ACTG2**	M**C**EEE**TT**ALVCDNGSGLCK<br>**P**EYDEAGPSIVHR	Both<br>Both	 P9
**ACTA1**<br>**ACTC1**<br>**ACTA2**	**Q**EYDEAGPSIVHR	Both	P1; P9; II161
**ACTC1**<br>**ACTA2**<br>**ACTG2**	DLYANNVLSGGTTMYPGIADR	Both	1902-1-274a; 1902-1-274b; 1902-1-279_1; 1902-1-279_2; D13e; D11; P1; P7i; P7l; P9; P11; P12; 5798; II161

*also in ACTB (P60712|ACTB_BOVIN Actin, cytoplasmic 1)/ACTG1 (P63258|ACTG_BOVIN Actin, cytoplasmic 2)

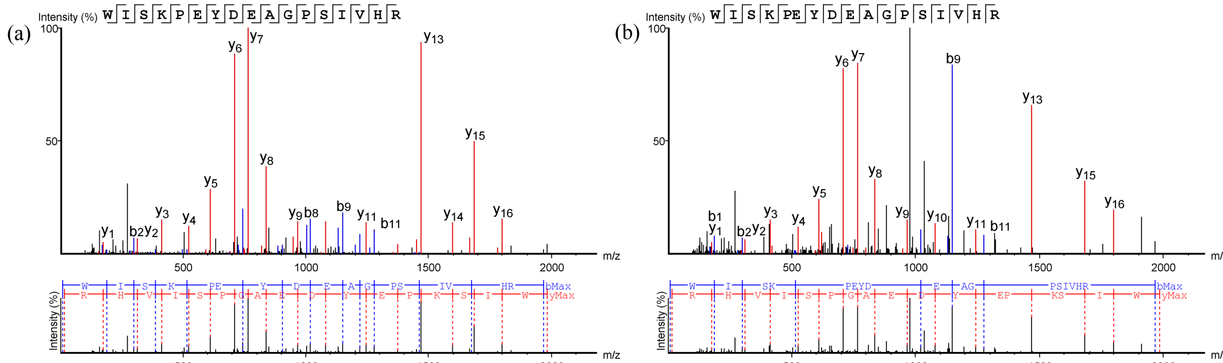

Figure S3.A6. Actin peptide marker. Bos peptide WISKPEYDEAGPSIVHR in **a) Cow+silver reference membrane,** score -l0lgP=59.01, *m/z*=662.0045, z=3, ppm=3.3; **b) Sample P9,** score -l0lgP=45.47, *m/z*=662.0058, z=3, ppm=5.3.

In five membrane metal thread samples, 1902-1-329a_1, 1902-1-329a_2, 1901-1-274a, 1901-1-274b, and P12, both cattle and sheep were identified based on collagen markers from COL1A1, COL1A2, and COL3A1. To determine exactly the species of the membranous tissue, additional peptidic markers were sought. The main smooth muscle proteins identified in the membranes, i.e. actic, desmin and myosin 11, have identical sequences between sheep and cattle and were not good candidates for species markers. However, markers were identified in other minor collagen chain proteins that are likely to come from the membranes as shown in all other samples. These peptides are shown in **Table S3.A2** as well as their identification in the membrane metal threads. All markers were identified as coming from *Bos taurus*, and none of the *Ovis aries* markers were identified, adding weight to the identification of the membranous tissue as cattle.

Table S3.A2. Other collagen peptide markers. Peptides allowing differentiation of sheep and cattle in minor collagen chain proteins. Samples in which the cattle markers were found are indicated, as well as the equivalent sheep sequence (not found in the membrane samples)

COL5A2: XP_024835542.1 collagen alpha-2(V) chain [Bos taurus]
COL6A1: DAA32939.1 TPA: collagen, type VI, alpha 1 [Bos taurus]; NP_001137337.1 collagen alpha-1(VI) chain precursor [Bos taurus]
COL6A3: XP_024846035.1 collagen alpha-3(VI) chain isoform X6 [Bos taurus]; DAA30927.1 TPA: collagen, type VI, alpha 3-like isoform 4 [Bos taurus]; XP_024846034.1 collagen alpha-3(VI) chain isoform X5 [Bos taurus]; DAA30925.1 TPA: collagen, type VI, alpha 3-like isoform 2 [Bos taurus]; DAA30926.1 TPA: collagen, type VI, alpha 3-like isoform 3 [Bos taurus]; XP_024846032.1 collagen alpha-3(VI) chain isoform X3 [Bos taurus]; XP_024846; 031.1 collagen alpha-3(VI) chain isoform X2 [Bos taurus]; DAA30924.1 TPA: collagen, type VI, alpha 3-like isoform 1 [Bos taurus]; XP_027393801.1 collagen alpha-3(VI) chain isoform X4 [Bos indicus x Bos taurus]

PROTEIN	BOS TAURUS SEQUENCE	MEMBRANES	OVIS ARIES SEQUENCE
**COL5A2**	GDPGTVGPPGPVGER	274A, 274B	GDPGTVGPPGPMGER
	GFTGLQGLPGPPGP**T**GEQGSAGIPGPFGPR	329A-1, 274A, 274B	GFTGLQGLPGPPGP**N**GEQGSAGIPGPFGPR
**COL6A1**	DAEEVISQTIDTITDMIK	329A-1, 329A-2, P12	DAEEVISQTIETITDMIK
	LLPPT**P**NNR	274A, P12	LLPPT**Q**NNR
**COL6A3**	I**A**EGVPQLLIVLTADR	329A-1, 329A-2	I**E**EGVPQLLIVLTADR
	VSVVALTPSGPVEAFDFAEYQ**P**ELFEK	329A-1, 329A-2, P12	VSVVALTPSGPVEAFDFAEYQ**S**ELFEK
	VVIHFTDGVDGDLAD**L**QR	329A-1, 329A-2, 274A, P12	VVIHFTDGVDGDLAD**V**QR
	GL**T**LLGGPAPNTGAALEFVLR	329A-2	GL**A**LLGGPAPNTGAALEFVLR
	IEDGVPQHLVLFLGG**K**	329A-2, P12	IEDGVPQHLVLFLGG**R**
	PVFPTELAFALDTSEGVTQD**R**	329A-2	PVFPTELAFALDTSEGVTQD**T**
	DLP**S**IEER	274A, P12	DLP**N**IEER
	GF**T**YNRPLR	P12	GF**M**YNRPLR

3.B Skin-based metal threads

3.B.1. Bioinformatics strategy

For each sample, the two fractions were combined into one search to create one output file. PEAKS 8.5 (BIOINFORMATICS SOLUTIONS INC.) was used to search the RAW data for matches against publicly available sequences in imported UniProt (www.uniprot.org) and NCBI (https://www.ncbi.nlm.nih.gov/protein) databases.

Searches were carried out using trypsin as enzyme, one allowed non-trypsin cleavage at any end, one missed cleavage, peptide mass tolerance (PMS) of 15 ppm, fragment mass error tolerance (MS/MS) of 0.02 Da, carbamidomethylation as a fixed modification, and deamidated (NQ), hydroxylation (P), and oxidation (M) as variable modifications.

1- A general search was conducted against the reviewed Swiss-Prot **database UniProtKB/Swiss-Prot** (last imported on 23rd August 2021) in PEAKS PTM allowing a maximum of three post-translational modifications. This was done to search for non-collagen proteins, e.g. plant or animal adhesives of mammalian and non-mammalian nature (milk, egg). Data were filtered with a false positive rate (FDR) of 1%, a protein score -10lgP ≥ 50, and one or two unique peptides.
2- The search for the collagen species was conducted against **a database of all vertebrate collagen sequences available in NCBI** (last imported on 19th August 2021) in PEAKS PTM allowing a maximum of six post-translational modifications to account for the large quantity of proline modifications to hydroxyproline. This search allowed to identify the most likely species of the leather as an ovicapra species (sheep or goat), as well as the presence or not of collagen from another species that could be part of an adhesive or varnish. Data were filtered with a FDR of 1%, a protein score -10lgP ≥ 50, and zero unique peptide.
3- Finally, a restricted search was conducted against a **database of collagen for Bovidae+Equus+Acipenser** (assembled on 20th January 2022) to which *de novo* peptides derived from *Acipenser* (sturgeon) were added. Files were searched in PEAKS PTM allowing a maximum of seven post-translational modifications. Data were filtered with a FDR of 1%, a protein score -10lgP ≥ 20, a minimum of two peptides and one unique peptide.

3.B.2. Protein identification in the skin metal threads

The proteins identified by the general database UniProtKB/Swiss-Prot are shown **Figure S3.B1** (excluding cytoskeletal keratins from handling contamination); they consist mainly of collagens. For comparison, skin samples prepared with different methods were analyzed by proteomics under the same conditions that the archaeological samples: raw hide (goat), parchment (cattle), vellum (cattle), vegetable-tanned leather (cattle), alum-tawed leather (cattle) and oil-tanned leather (cattle). Ovicapra markers were found in all samples with 21 markers on

average; 313 had only 13 markers. The skin substrate species was identified as *Ovis* sp. in 29 samples and *Capra* sp. in 17 samples, most specifically *Ovis aries* (domestic sheep) and *Capra hircus* (domestic goat), the only species for which collagen sequences are fully available in NCBI. The presence of sheep or goat-specific peptides, as well as the protein coverage of the main three collagen chains is shown in **SI_Peptide identification in Skins.xlsx**. Egg white was found in 11 samples (see below), milk in four samples but could come from laboratory contamination, and wheat in two samples. Besides for samples **1902-1-229b_1** and **P4d** (detailed below), the *Other* proteins are typically histones, actin or tubulins found as minor components. In the raw hide, parchment and vellum samples, some *Other* proteins are found, but not in the leather, indicative harsher processing conditions for these proteins to remain in tanned leather.

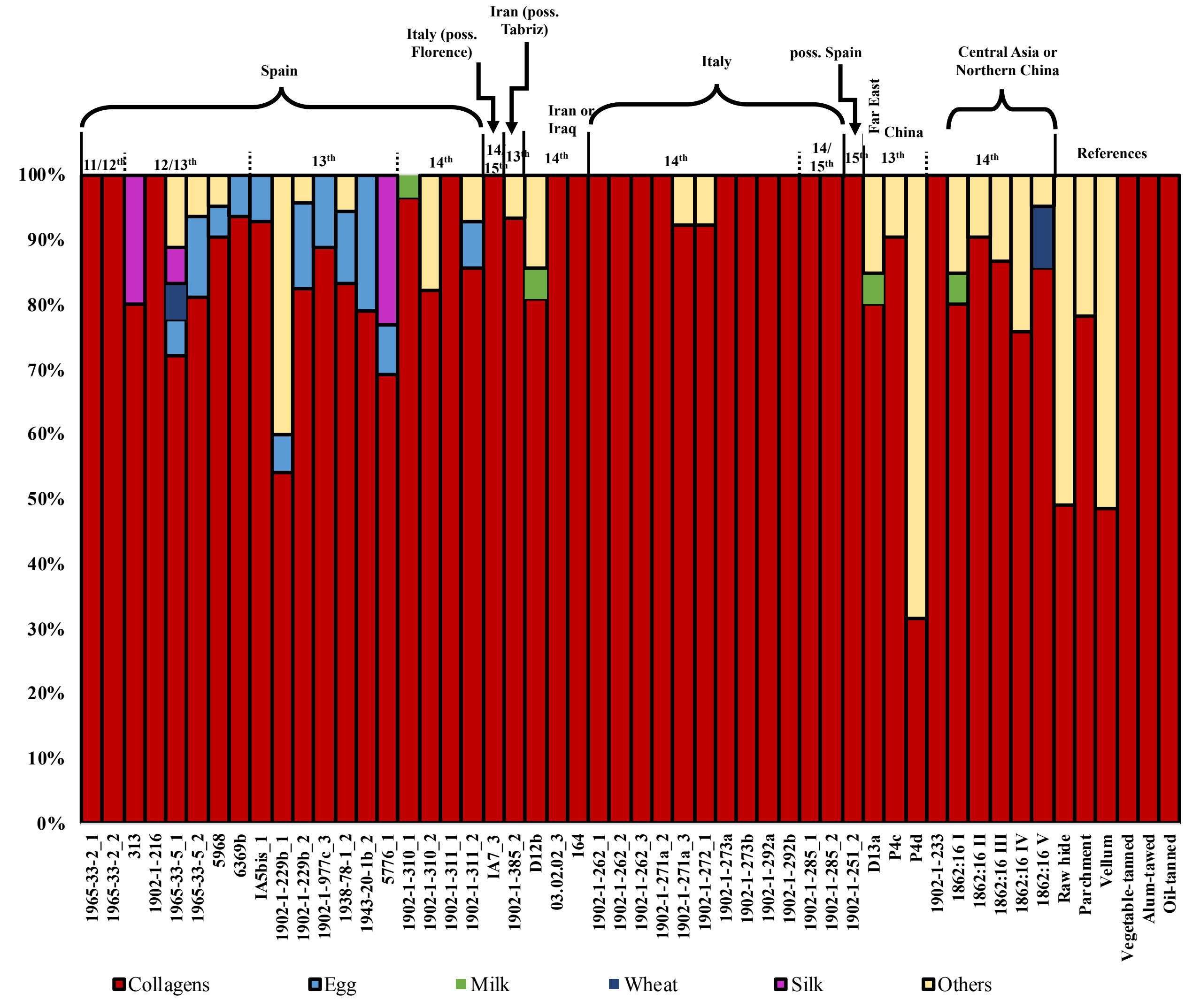

Figure S3.B1. Percentage of proteins identified in each sample, classified by protein families. With *UniProtKB/Swiss-Prot reviewed*, with a FDR of 1%, a protein score -10lgP ≥ 50, a minimum of two unique peptides.

Identification of Egg white proteins

The full identification of egg white proteins is detailed in **Table S3.B1** for the 11 samples where they were detected. **Figure S3.B2** show MS/MS spectra of peptides unique to *Gallus gallus*, identified in some of the metal threads.
For unique peptide Blast results: See excel file **SI_Proteomics Identification in Skins (Egg peptide identification).**

Table S3.B1. Identification of egg proteins in metal-wrapped skin threads. With *UniProtKB/Swiss-Prot reviewed*, with a FDR of 1%, a protein score -10lgP ≥ 50, a minimum of two peptides and **one** unique peptide. -10lgP is Peaks score; TOTAL PEPT. is total number of peptides; U. is number of unique peptides in PEAKS; % is protein percentage coverage and # PSM is number of peptide-to-spectrum matches

SAMPLE	*ACCESSION NUMBER*	*PROTEIN NAME*	*SPECIES*	*-10lgP*	*%*	*TOTAL PEPT.*	*U.*	*# PSM*
IA5bis	P01012|OVAL_CHICK	Ovalbumin	*Gallus gallus*	123.11	31	10	9	53
	P01014|OVALY_CHICK	Ovalbumin-related protein Y	*Gallus gallus*	51.76	5	2	1	5
5776_1	P01012|OVAL_CHICK	Ovalbumin	*Gallus gallus*	92.84	20	7	7	33
5968	P01012|OVAL_CHICK	Ovalbumin	*Gallus gallus*	167.98	53	20	19	162
	P01014|OVALY_CHICK	Ovalbumin-related protein Y	*Gallus gallus*	64.37	5	2	1	12
6369b	P01012|OVAL_CHICK	Ovalbumin	*Gallus gallus*	62.97	11	3	3	14
1902-1-229b_1	P01012|OVAL_CHICK	Ovalbumin	*Gallus gallus*	144.43	47	20	12	97
	P02789|TRFE_CHICK	Ovotransferrin	*Gallus gallus*	52.07	3	2	2	5
1902-1-229b_2	P01012|OVAL_CHICK	Ovalbumin	*Gallus gallus*	188.81	58	30	18	178
	P02789|TRFE_CHICK	Ovotransferrin	*Gallus gallus*	84.07	5	3	3	14
1902-1-311_2	P01012|OVAL_CHICK	Ovalbumin	*Gallus gallus*	63.61	9	2	2	2
1902-1-977_3	P01012|OVAL_CHICK	Ovalbumin	*Gallus gallus*	186.37	60	26	14	220
	O73860|OVAL_MELGA	Ovalbumin	*Meleagris gallopavo*	117.95	20	11	1	60
	P01014|OVALY_CHICK	Ovalbumin-related protein Y	*Gallus gallus*	64.11	5	2	1	16
	P00698|LYSC_CHICK	Lysozyme	*Gallus gallus*	77.47	16	2	1	11
	P00703|LYSC_MELGA + 9 other species	Lysozyme	*Meleagris gallopavo*	69.85	18	2	1	6
	P02789|TRFE_CHICK	Ovotransferrin	*Gallus gallus*	97.91	6	4	4	19
1938-78-1_2	P01012|OVAL_CHICK	Ovalbumin	*Gallus gallus*	178.26	52	24	21	142
	P01014|OVALY_CHICK	Ovalbumin-related protein Y	*Gallus gallus*	63.54	5	2	1	12
	P00698|LYSC_CHICK	Lysozyme	*Gallus gallus*	101.89	30	4	2	24
1943-20-1b_2	P01012|OVAL_CHICK	Ovalbumin	*Gallus gallus*	192.18	73	39	24	275
	O73860|OVAL_MELGA	Ovalbumin	*Meleagris gallopavo*	108.45	24	12	1	58
	P19104|OVAL_COTJA	Ovalbumin	*Coturnix japonica*	85.75	19	9	2	30
	P01014|OVALY_CHICK	Ovalbumin-related protein Y	*Gallus gallus*	55.57	5	3	2	19
	P00698|LYSC_CHICK	Lysozyme	*Gallus gallus*	90.36	24	3	2	27

	P00702|LYSC_PHACO	Lysozyme	*Phasianus colchicus colchicus*	68.84	16	2	1	13
	P49663|LYSC_PHAVE	Lysozyme	*Phasianus versicolor*	68.84	18	2	1	13
	P02789|TRFE_CHICK	Ovotransferrin	*Gallus gallus*	81.05	6	4	4	18
1965-33-5_1	P01012|OVAL_CHICK	Ovalbumin	*Gallus gallus*	89.58	12	4	4	23
1965-33-5_2	P01012|OVAL_CHICK	Ovalbumin	*Gallus gallus*	187.68	62	31	19	170
	P01014|OVALY_CHICK	Ovalbumin-related protein Y	*Gallus gallus*	65.95	5	2	1	10
	P00698|LYSC_CHICK	Lysozyme	Gallus gallus	84.24	27	4	4	10

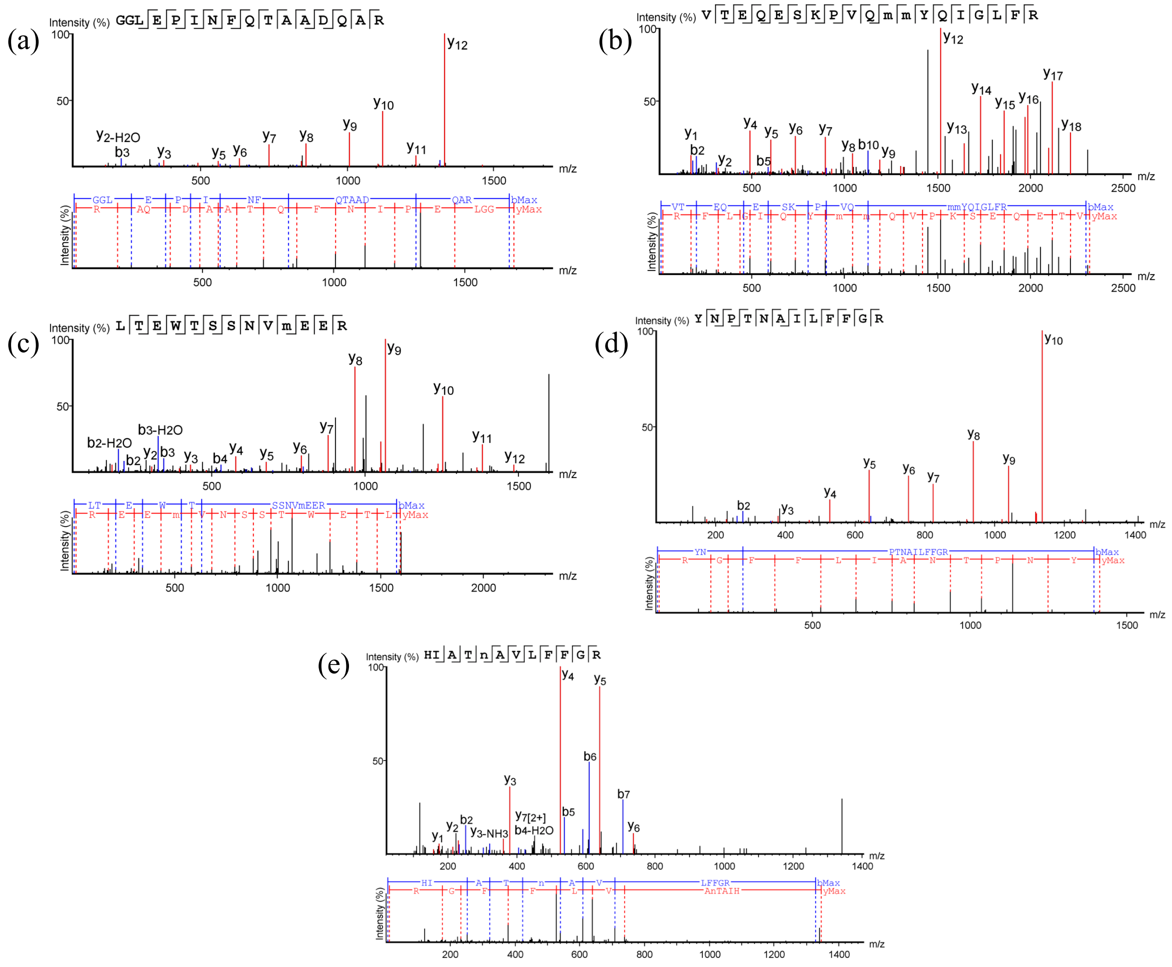

Figure S3.B2. Egg white peptide markers in skin threads. a) Chicken ovalbumin peptide R.GGLEPINFQTAADQAR.E, score -l0lgP=57.60, *m/z*=844.4238, z=2, ppm=0.3, in **sample 1965-33-5_2**; b) Chicken ovalbumin peptide R.VTEQESKPVQMMYQIGLFR.V with oxidation of methionine, score -l0lgP=53.94, *m/z*=772.7169, z=3, ppm=-0.1, in **sample 1902-1-977_3;** c) Chicken ovalbumin peptide K.LTEWTSSNVMEER.K with oxidation of methionine, score -l0lgP=45.27, *m/z*=799.3635, z=2, ppm=2.1, in **sample 1943-20-1b_2**; d) Chicken ovalbumin-related protein Y peptide R.YNPTNAILFFGR.Y, score -l0lgP=50.69, *m/z*=706.8727, z=2, ppm=4.3, in **sample 5968**; e) Chicken ovalbumin peptide K.HIATNAVLFFGR.C with deamidation of asparagine, score -l0lgP=46.73, *m/z*=449.5811, z=3, ppm=5.5, in **sample 1902-1-229b_1**

Identification of fish proteins

A database of all vertebrate collagen sequences available in NCBI identified sturgeon collagen in all samples made from sheep skins. Fish glue, or isinglass, is made from the dried air-bladders of some fish; in the past mostly sturgeon [3]. In Eurasia, there are 12 species of *Acipenser*, two species of *Huso* and three species of *Pseudoscaphirhynchus*, the latest are unlikely to have been used in fish glue due to their smaller size. Larger and thick air-bladders are most cost-effectively sourced from large species [3]. The Eurasian species of *Acipenser* and *Huso* are listed in **Table S3.B2**. Species from Southern China, Korea and Japan (*A. mikadoi, A. dabryanus, A. sinensis*) have geographic habitats quite remote from where the threads were likely produced.

Table S3.B2. Sturgeon species in Eurasia (source: http://www.fishbase.org)

Latin name	Distribution	Habitat
**Western Europe**		
Acipenser naccarii (Adriatic sturgeon) Bonaparte, 1836	Adriatic Sea and its tributaries between Po (Italy) and Buna (Albania) drainages.	Marine; freshwater
Acipenser sturio (European sea sturgeon) Linnaeus, 1758	North Sea, European coasts of Atlantic, northern Mediterranean eastward to Rhodos, western and southern Black Sea	Marine; freshwater
Huso huso (beluga) Linnaeus, 1758	Caspian, Black, Azov and Adriatic Sea basins.	Marine; freshwater
**Caspian Sea/Black Sea/ Sea of Azov**		
Acipenser gueldenstaedtii (Russian sturgeon) J. F. Brandt & Ratzeburg, 1833	Black Sea, Sea of Azov and Caspian Sea basins. In Azerbaijan, Bulgaria, Georgia, Iran, Kazakhstan, Romania, Russia, Turkey, Turkmenistan, and Ukraine.	Marine; freshwater
Acipenser nudiventris (Fringebarbel sturgeon) Lovetsky, 1828	Black, Azov, Caspian and Aral Sea, ascending some rivers (Danube up to Bratislava, Volga up to Kazan, Ural up to Chkalov)	Marine; freshwater
Acipenser persicus (Persian sturgeon) Borodin, 1897	Caspian basin, most abundant in the southern part	Marine; freshwater
Acipenser ruthenus (Sterlet) Linnaeus, 1758	Rivers draining to Black, Azov and Caspian Seas; Siberia from Ob eastward to Yenisei drainages.	Freshwater
Acipenser stellatus (Starry sturgeon) Pallas, 1771	Caspian, Black, Azov and Aegean Seas, ascending rivers to spawn.	Marine; freshwater
Huso huso (beluga) Linnaeus, 1758	Caspian, Black, Azov and Adriatic Sea basins.	Marine; freshwater
**Amur river basin/Sea of Japan/Sea of Okhotsk**		
Acipenser mikadoi (Sakhalin sturgeon) Hilgendorf, 1892	Bering Sea, Tumnin (Datta) river, to northern Japan and Korea.	Marine; freshwater
Acipenser schrenckii (Japanese sturgeon) J. F. Brandt, 1869	Endemic to the Amur River system.	Marine; freshwater
Huso dauricus (kaluga) Georgi, 1775	Amur basin, ascending far up to the Argun, Shilka, and Onon.	Marine; freshwater

Siberia		
Acipenser baerii<br>*Acipenser baerii baerii* (Siberian sturgeon)<br>J. F. Brandt, 1869	Siberia, rivers Ob, Irtysh, Yenisei, Lena, Kolyma, Khatanga, Pyasina, Anabar, Olenyok, Yana and Lake Baikal	Freshwater
Acipenser ruthenus (Sterlet)<br>Linnaeus, 1758	Rivers draining to Black, Azov and Caspian Seas; Siberia from Ob eastward to Yenisei drainages.	Freshwater
**Yangtze River**		
Acipenser dabryanus (Yangtze sturgeon)<br>A. H. A. Duméril, 1869	China in Yangtze River system and Korea	Freshwater
Acipenser sinensis (Chinese sturgeon)<br>J. E. Gray, 1835	China and Japan	Marine; freshwater

After establishing the species present in the skin threads, a restricted database **(Bovidae + Equus + Acipenser)** was created to focus on the identification of the sturgeon peptides, including a few sequences determined by *de novo* sequencing, either using PEAKS DENOVO or manual sequencing.

At the time of writing, the sturgeon family of proteins is best represented in NCBI, with complete collagen sequences for *Acipenser schrenckii* (the Amur sturgeon) and *Acipenser ruthenus* (the sterlet). The *Huso* (large Eurasian sturgeons) and the *Pseudoscaphirhynchus* (small sturgeons from the Aral sea and tributaries) genera are not represented for collagen. The protein accession numbers in NCBI for *Acipenser schrenckii* are BAR72356.1 and BAR72360.1 (100% homology), BAR72357.1, BAR72358.1, and BAR72359.1 (100% homology) for collagen I alpha 1 (the two entry groups are identical sequences of 1457 amino acids but for one residue in position 932: S_{932} in the first group and G_{932} in the second), and BAT51012.1 for collagen I alpha 2 (1357 amino acids). There are also three entries for collagen type II alpha 1, but this collagen type was identified with low coverage in the threads and therefore not reported here. *Acipenser ruthenus* (the only sturgeon species with an annotated genome [4]) has close to 300 collagen entries in NCBI, last uploaded in June 2020. The main collagen I alpha 1 sequences identified in the threads are XP_034775076.1 and XP_033913598.1 (1457 amino acids) and XP_033911740.1 (1452 amino acids), and the main collagen I alpha 2 sequences are XP_033848627.2 and XP_033855900.1 (1357 amino acids). The homology between sequence and number of residues where substitutions are found are shown **Table S3.B3**. At 99% homology, *A. ruthenus* XP_034775076.1 and XP_033913598.1 are almost identical. They are however quite distinct from XP_033911740.1 (91%/92% homology). For COL1A2, the two *A. ruthenus* sequences are also distinct with 95% homology only. The presence of at least two distinct sequences for both COL1A1 and COL1A2 is due to the polyploidy of the sturgeon species. *A. ruthenus* is considered a tetraploid species and contains duplicate genes [4]. The *A. schrenckii* sequences are more similar to XP_033911740.1 and XP_033848627.2 (see **Tables S3.B4 and S3.B5**), however the full *A. schrenckii* proteome is not available, so the collagen type I set is likely incomplete.

Table S3.B3. Homology of *Acipenser* collagens: Percentage (number of differing residues). *A. ruthenus:* XP_034775076.1, XP_033911740.1 and XP_033913598.1 (COL1A1), and XP_033848627.2 and XP_033855900.1 (COL1A2). *A. schrenckii:* BAR72356.1/60.1 (COL1A1), and BAT51012.1 (COL1A2).

COL1A1				COL1A2		
	XP_034775076.1	*XP_033913598.1*	*BAR72356.1/60.1*		*XP_033855900.1*	*BAT51012.1*
XP_033911740.1	91% (30)	92% (29)	92% (20)	XP_033848627.2	95% (70)	98% (22)
XP_034775076.1	-	99% (17)	98% (28)	BAT51012.1	95% (67)	-
BAR72356.1/60.1	98% (28)	99% (11)	-			

Figure S3.B3 shows the identification of the *A. ruthenus* sequences in the modern and archaeological samples (*A. schrenckii* is not shown since similar to *A. ruthenus*). *Acipenser* COL1A1 is found in all samples due to the fact that some parts of the sequences are conserved with mammals, and in particular sheep. The identification in some samples is essentially due to these common *Ovis aries-Acipenser* peptides (see **Tables S3.B4 and S3.B5**). Samples for which the fish was confirmed through identification of specific markers are indicated in green. The samples for which fish was not confirmed by markers are easily identified for not having COL1A2, as these sequences do not contain matched peptides common to mammals. The presence of *Acipenser* COL1A2 in a sample is therefore a strong indicator of the presence of sturgeon glue.

			XP_034775076.1 XP_033913598.1 *Acipenser ruthenus*	XP_033911740.1 *Acipenser ruthenus*	XP_033848627.2 *Acipenser ruthenus*	XP_033855900.1 *Acipenser ruthenus*
References	20th	Blank				
		Raw hide	7	5		
		Parchment	9	4		
		Vellum	10	6		
		Vegetable-tanned	5	3		
		Alum-tawed	6	9		
		Oil-tanned		6		
Spain	11th/12th	1965-33-2_1	9	6		
		1965-33-2_2	14	7		
	12th/13th	313		4		
		1902-1-216	5	3		
		1965-33-5_1	9			
		1965-33-5_2	7			
possibly Spain		5968	12	3		
		6369b	11			
Spain	13th	IA5bis_1	9	4		
		1902-1-229b_1	14	10		
		1902-1-229b_2	10			
		1902-1-977c_3	7			
		1938-78-1_2	10			
		1943-20-1b_2	9	6		
		5776_1	5	5		
	14th	1902-1-310_1	16	7		
		1902-1-310_2	12	4		
		1902-1-311_1	9			
		1902-1-311_2	7	4		
Italy (possibly Florence)	14th/15th	IA7_3	16	26	15	6
Iran (possibly Tabriz)	possibly 13th	1902-1-385_2	15	33	24	14
Persia	around 14th	D12b	15	38	25	16
		D12b (back)	16	42	36	18
Iran or Iraq	14th	03.02.02_3	11	20	15	6
	possibly 14th	164	28	53	40	32
Italy	14th	1902-1-262_1	13	27	18	10
		1902-1-262_2	12	22	11	5
		1902-1-262_3	14	19	20	13
		1902-1-271a_2	11	18	12	5
		1902-1-271a_3	13	24	16	7
		1902-1-272_1	26	59	46	35
		1902-1-273a	24	48	43	30
		1902-1-273b	17	25	23	12
		1902-1-292a	12	28	17	13
		1902-1-292b	12	25	17	13
	14th/15th	1902-1-285_1	31	54	51	37
		1902-1-285_2	21	42	39	27
possibly Spain	15th	1902-1-251_2	10	19	15	7
Far East	13th	D13a	20	40	28	19
China		P4c	13	26	15	10
		P4d	13	22	13	6
Spain or Iran	14th	1902-1-233	22	49	37	32
Central Asia or Northern China		1862:16 I	25	54	52	44
		1862:16 II	19	46	41	32
		1862:16 III	18	39	31	23
		1862:16 IV	19	43	35	26
		1862:16 V	26	47	38	28

Figure S3.B3. Identification of the *Acipenser ruthenus* collagen chains. The percentage coverage, visualized with data bars, is given for both modern and archaeological samples. In green are samples for which sturgeon presence was confirmed by specific markers.

***De novo* sequencing**: Due to the lack of genomes and collagen sequences for sturgeon, a few sequences were discovered by *de novo* sequencing. These sequences are shown in grey **Table S3.B4** and **Figures S3.B4 to S3.B6** for collagen type I alpha 1 and **Table S3.B5** and **Figures S3.B7 to S3.B11** for collagen type I alpha 2. Due to the complex nature of the sturgeon genome, it was common to find multiple sequences for the same peptide in a sample. Up to four versions were found for COL1A2 peptide GIAGDSGXPGXAGXR in position 569-583, including a new *de novo* version. All four versions were identified in two samples: **1902-1-233** and **1862:16 II**, indicating perhaps that multiple species of sturgeon might have contributed to the glue. Of the five markers found to be from *A. schrenckii* only in COL1A2, peptide G_{323}GPGPQGPAGAAGPR_{337} was found in all samples, but the four other markers (A_{220}GAAGPAGAR_{229}, G_{409}GPMGAPGSR_{418}, G_{569}IAGDSGLPGAAGSR_{583}, and G_{878}LPGGAGGTGESGK_{891}) were only identified in samples **1902-1-233**, **1902-1-292a** and **1862:16 (II, III and IV)**. While there is possibly a degradation effect that might explain why these markers are not more commonly found, it should be noted that sample **1902-1-292a** has three of these markers while not having a generally high percentage of protein coverage of the sturgeon collagen (**Figure S3.B3**). Sample **1902-1-292b** with similar coverages does not contain these markers.

The COL1A2 peptide in position 1060-1075 is singularly found only in samples **1862:16**, and **D13a**. Two versions of the peptide are found DGQPGHPGPIG**P**AGSR and DGQPGHPGPIG**Q**AGSR, with the first version found in all 1862:16 samples (it is also found with low score in sample **D13a**). This gives strong evidence that these samples were made with sturgeon glue from a species not used in the other samples, and considering the geographical origin of these samples, likely indicates a sturgeon species found in Mongolia/Russia/China (e.g. *A. baerii*, *A. schrenckii*, *A. ruthenus* or *Huso Dauricus*, see **Table S3.B2**). Another peptide found only in samples **1862:16** is COL1A1 G_{727}AAGLPGIK_{735}; it is a peptide common to both *A. schrenckii* and *A. ruthenus*.

Table S3.B4. Alignment of the main *Acipenser* collagen type I alpha 1 chains identified in skin samples: *Acipenser ruthenus* XP_034775076.1 and XP_033911740.1, and *Acipenser schrenckii* BAR72356.1 using NCBI Blast alignment tool https://blast.ncbi.nlm.nih.gov/. Amino acid substitutions, compared to the top sequence XP_034775076.1, are indicated in red font. Highlighted are the most commonly identified peptides used as markers. Green: found in all three sequences; Blue: found in both *A. schrenckii* and *A. ruthenus*; Yellow: found in *A. schrenckii* only, White: found in *A. ruthenus* only. The common peptides highlighted in Red are sequences found in some mammals including *Ovis aries*, and therefore not considered as *Acipenser* markers. New peptides identified by de novo are highlighted in Grey.

XP_034775076.1	1	MFSFVDTRIALLLAATVLLARGQGEDDLSAGNCMKDGQLYNDKDVWKPEPCQICVCDSGN
XP_033911740.1	1	MFSFVDTRIALLLAATVLLARGQGEDDLSGGSCMKDGQLYNDKDVWKPEPCQICVCDSGN
BAR72356.1	1	MFSFVDTRIALLLAATVLLARGQGEDDLSAGNCMKDGQLYNDKDVWKPEPCQICVCDSGN
XP_034775076.1	61	ILCDEVICEDTTDCPNPEIPFGECCPICPDSEVSQEPAYSEVEGPQGPKGERGMKGDRGL
XP_033911740.1	61	ILCDEVICEDTTDCPNPEIPFGECCPICPDSEVAQEP---EVERPQGPKGDRGMKGDRGL
BAR72356.1	61	ILCDEVICEDTTDCPNPEIPFGECCPICPDSEVSQEPAYSEVEGPQGPKGERGIKGDRGL
XP_034775076.1	121	PGPAGNDGIPGQPGLPGPPGPPGPPGLGGNFAPQMSYGYDEKSGGGGMSAPGPMGPMGPR
XP_033911740.1	118	PGSAGNDGIPGQPGLPGPPGPPGPPGLGGNFAPQMSHGYDEKSTG--ISVPGPMGPMGPR
BAR72356.1	121	PGPAGNDGIPGQPGLPGPPGPPGPPGLGGNFAPQMSYGYDEKSGGGGMSAPGPMGPMGPR
XP_034775076.1	181	GPPGPAGSNGPQGFPGPHGEPGEPGASGPMGPRGPAGPPGKNGDDGESGKPGRPGERGPS
XP_033911740.1	176	GPPGPPGLNGPQGFPGPHGEPGEPGASGAMGPRGPAGPPGKNGEDGESGKPGRPGE GPS
BAR72356.1	181	GPPGPAGSNGPQGFPGPHGEPGEPGASGAMGPRGPAGPPGKNGDDGESGKPGRPGERGPS
XP_034775076.1	241	GPQGARGFPGTPGLPGIKGHRGFSGLDGAKGDSGPAGPKGEPGSSGENGTPGAMGPRGLP
XP_033911740.1	236	GPQGARGFPGTPGLPGIKGHRGFSGLDGSKGETGPAGPKGEPGSSGENGTPGAMGPRGLP
BAR72356.1	241	GPQGARGFPGTPGLPGIKGHRGFSGLDGAKGDSGPAGPKGEPGSSGENGTPGAMGARGLP
XP_034775076.1	301	GERGRPGPSGPAGARGNDGASGAAGPPGSTGPAGAPGFPGGPGAKGEVGPAGGRGSDGPQ
XP_033911740.1	296	GERGRPGPPGTAGARGNDGAVGAAGPPGATGPAGAPGFPGSPGAKGEVGPAGGRGSDGAQ
BAR72356.1	301	GERGRPGPSGPAGARGNDGASGAAGPPGSTGPAGAPGFPGGPGAKGEVGPAGGRGSDGPQ
XP_034775076.1	361	GARGEPGSPGPAGAPGAAGNPGTDGQAGAKGGVGAPGIAGAPGFPGPRGPPGPQGPGGAP
XP_033911740.1	356	GARGEPGNPGPAGAAGAAGNPGTDGQAGAKGATGAPGIAGAPGFPGPRGPPGPQGTGGAP
BAR72356.1	361	GARGEPGNPGPAGAPGAAGNPGTDGQAGAKGGVGAPGIAGAPGFPGPRGPPGSQGPGGAP
de novo		GPPGPQGTSGAP
XP_034775076.1	421	GAKGNNGDAGTPGARGEAGAKGEPGPGGVQGPPGPSGEEGKRGARGEPGPGGAPGPNGER
XP_033911740.1	416	GAKGNNGESGAPGARGEAGAKGEPGTPGVQGPPGASGEEGKRGARGEPGPSGAPGPNGER
BAR72356.1	421	GAKGNNGDAGTPGARGEAGAKGEPGPGGVQGPPGPSGEEGKRGARGEPGPGGAPGPNGER
de novo		GAK
XP_034775076.1	481	GSPGSRGFPGADGAAGPKGGPGERGGPGPAGAKGSTGESGRPGEPGLPGAKGLTGSPGSP
XP_033911740.1	476	GSPGSRGFPGADGSAGPKGSPGERGSAGVAGPKGSPGEVGRAGEPGLPGAKGLTGSPGSP
BAR72356.1	481	GSPGNRGFPGADGAAGPKGGPGERGGPGPAGAKGSTGESGRPGEPGLPGAKGLTGSPGSP
XP_034775076.1	541	GPDGKTGPAGPAGQDGRAGPPGPSGVRGQPGVMGFPGPKGAAGDAGKPGERGVAGAPGAL
XP_033911740.1	536	GPDGKTGPAGPAGQDGRSGPPGPTGARGQPGVMGFPGPKGAAGEAGKPGERGVVGAPGAL
BAR72356.1	541	GPDGKTGPAGPAGQDGRAGPPGPSGARGQPGVMGFPGPKGAAGDAGKPGERGVAGAPGAL
de novo		GVVGAPGAV
XP_034775076.1	601	GATGKDGDAGAPGPPGPAGPAGERGEGGPAGAPGFQGLPGPQGAAGESGKPGEQGIPGDV
XP_033911740.1	596	GAPGKDGDAGAPGPSGPAGPAGERGEGGPAGAPGFQGLPGPQGSSGEPGKPGEQGVPGDA
BAR72356.1	601	GATGKDGDAGAPGPSGPAGPAGERGEGGPAGAPGFQGLPGPQGAAGESGKPGEQGIPGDV
de novo		GAPGK
XP_034775076.1	661	GSPGPAGSRGERGFPGERGGPGATGPAGARGSPGSPGKDGAKGETGPAGAPGGQGAPGLQ
XP_033911740.1	656	GAPGPAGSRGERGFPGERGGPGATGPAGARGSPGSPGNDGAKGETGAVGAPGGQGPPGLQ
BAR72356.1	661	GSPGPAGSRGERGFPGERGGPGATGPAGARGSPGSPGNDGAKGETGPAGAPGGQGAPGLQ
XP_034775076.1	721	GMPGERGAAGLPGIKGDRGDVGVKGGDGAPGKDGIRGMTGPIGPPGPAGAPGEKGESGPA
XP_033911740.1	716	GMPGERGAAGLPGIKGDRGDAGAKGADGASGKDGIRGMTGPIGPPGPSGAPGDKGETGPA
BAR72356.1	721	GMPGERGAAGLPGIKGDRGDGGVKGGDGAPGKDGIRGMTGPIGPPGPSGAPGEKGESGPA

XP_034775076.1	781	GPGGPTGSRGAPGERGEAGPPGPAGFAGPPGADGQAGAKGEPGDSGAKGDAGAPGPAGPT
XP_033911740.1	776	GPGGPTGARGAPGERGEPGPAGPAGFAGPPGADGQAGAKGEPGDSGAKGDAGAPGAAGPT
BAR72356.1	781	GPGGPTGSRGSPGERGESGPPGPAGFAGPPGADGQAGAKGEPGDSGAKGDAGSPGPAGPT
XP_034775076.1	841	GAPGPAGNVGATGPKGSRGAAGPPGATGFPGAAGRVGPPGPSGNAGAPGPPGPGGKEGQK
XP_033911740.1	836	GAPGPAGNVGATGPKGSRGAAGPPGATGFPGAAGRVGPPGPAGNSGPPGPPGPSGKEGQK
BAR72356.1	841	GAPGPAGNVGATGPKGSRGAAGPPGATGFPGAAGRVGPPGPSGNAGAPGPPGPGGKEGQK
XP_034775076.1	901	GNRGDTGAAGRPGEPGPAGPPGPTGEKGSPGSDGSPGPSGIPGPQGIAGQRGIVGLPGQR
XP_033911740.1	896	GNRGDTGPAGRSGEPGPAGPPGSTGEKGSPGSDGAPGSAGIPGPQGIAGQRGIVGLPGQR
BAR72356.1	901	GNRGDTGAAGRPGEPGPAGPPGPTGEKGSPGSDGSPGSSGIPGPQGIAGQRGIVGLPGQR
XP_034775076.1	961	GERGFSGLPGPTGEPGKQGPGGPSGERGPPGPMGPPGLSGAPGEAGREGAAGNEGAPGRD
XP_033911740.1	956	GERGFPGLPGPGGEPGKQGPSGPSGERGPPGPMGPPGMSGAPGEIGREGAPGNEGSPGRD
BAR72356.1	961	GERGFSGLPGPTGEPGKQGPGGPSGERGPPGPMGPPGLSGAPGEAGREGAAGNEGAPGRD
XP_034775076.1	1021	GGPGPKGDRGETGPAGAPGAPGAPGAPGPVGPAGKNGDRGESGPAGPAGPIGPAGARGPA
XP_033911740.1	1016	GAAGPKGDRGETGAAGTPGAPGPPGAPGAVGPAGKSGDRGETGPAGPAGPAGPAGARGVA
BAR72356.1	1021	GGPGPKGDRGESGPAGAPGAPGAPGAPGPVGPAGKNGDRGESGPAGPAGPAGPAGARGSA
XP_034775076.1	1081	GAQGARGDKGEAGEAGERGMKGHRGFTGLQGLPGPAGPSGEQGPAGASGPAGPRGPAGPA
XP_033911740.1	1076	GAQGLRGDKGEAGEAGERGLKGHRGFTGLQGLPGPPGSHGEQGPAGASGPAGPRGPAGSA
BAR72356.1	1081	GAQGARGDKGEAGEAGERGMKGHRGFTGLQGLPGPAGHSGEQGPAGASGPAGPRGPAGPA
de novo		GFTGLQGLPGSPGPHGEQGPAGASGPAGPR
XP_034775076.1	1141	GSSGKDGVSGLPGPIGPPGPRGRTGDIGPAGPPGTPGPPGPPGPPGGGFDFGFVAQPSQE
XP_033911740.1	1136	GSAGKDGASGMPGPIGPPGPRGRTGEIGPAGPPGIPGPPGPPGPPGGGFDFGFVAQPAQE
BAR72356.1	1141	GSSGKDGVSGLPGPIGPPGPRGRTGDIGPAGPPGTPGPPGPPGPPGGGFDFGFVAQPSQE
XP_034775076.1	1201	KAPDPFRHYRADDANVARDRDLEVDTTLKSLSQQIENIRSPEGSRKNPARTCRDLKMCHP
XP_033911740.1	1196	KAPDPFRHYRADDANVARDRDLEVDTTLKSLSQQIENIRSPEGTKKNPARSCRDLRMCHP
BAR72356.1	1201	KAPDPFRHYRADDANVARDRDLEVDTTLKSLSQQIENIRSPEGTKKNPARSCRDLKMCHP
XP_034775076.1	1261	EWKSGEYFVDPNQGCTLDAVKVYCNMETGETCVYPTQANIPQKNWYTSKNAKDKKHVWFG
XP_033911740.1	1256	EWKSGEYFVDPNQGCTLDAVKVYCNMETGETCVYPTQANIPQKNWYTSKNAKDKKHVWFG
BAR72356.1	1261	EWKSGEYFVDPNQGCTLDAVKVYCNMETGETCVYPTQANIPQKNWYTSKNAKDKKHVWFG
XP_034775076.1	1321	ETMSDGFQFEYGGEGSDAADVNIQLTFLRLMSTEASQNITYHCKNSVAYMDQQTGNLKKA
XP_033911740.1	1316	ETMSDGFQFEYGGEGSNADDVNIQLTFLRLMATEASQNITYHCKNSIAYMDQQAGNLKKA
BAR72356.1	1321	ETMSDGFQFEYGGEGSDAADVNIQLTFLRLMATEASQNITYHCKNSIAYMDQQAGNLKKA
XP_034775076.1	1381	LLLQGSNEIELRGEGNSRFTYSVTEDGCTRHTGAWGKTVIEYKTTKTSRLPIIDIAPMDV
XP_033911740.1	1376	LLLQGSNEIEIRAEGNSRFTYSVTEDGCTRHTGTWGKTVIDYKTTKTSRLPITDIAPMDV
BAR72356.1	1381	LLLQGSNEIEIRAEGNSRFTYSVTEDGCTRHTGAWGKTVIDYKTTKTSRLPIIDIAPMDV
XP_034775076.1	1441	GAPDQEFGIEVGPVCFL
XP_033911740.1	1436	GAPDQEFGIEVGPVCFL
BAR72356.1	1441	GAPDQEFGIEVGPVCFL

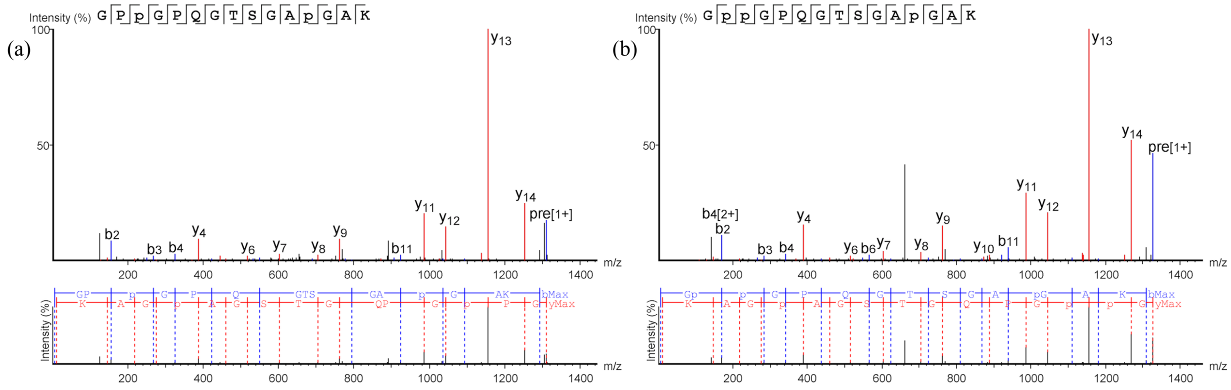

Figure S3.B4. Sturgeon *de novo* peptide X.GPPGPQGTSGAPGAK.X (collagen type I alpha 1) in sample 1902-1-385_2: with (a) two hydroxylation (P), score -l0lgP=39.11, *m/z*=655.8187, z=2, ppm=-2.6, # PSM=6, and (b) three hydroxylation (P), score -l0lgP=41.79, *m/z*=663.8159, z=2, ppm=-2.9, # PSM=6

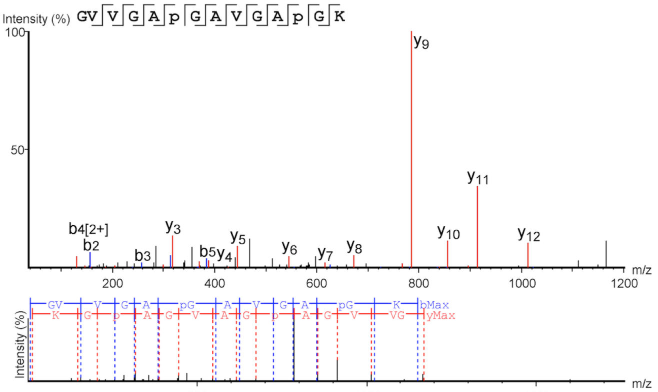

Figure S3.B5. Sturgeon *de novo* peptide X.GVVGAPGAVGAPGK.X (collagen type I alpha 1) in sample 1902-1-272_1: with two hydroxylation (P), score -l0lgP=47.91, *m/z*=584.8185, z=2, ppm=-2.0, # PSM=6

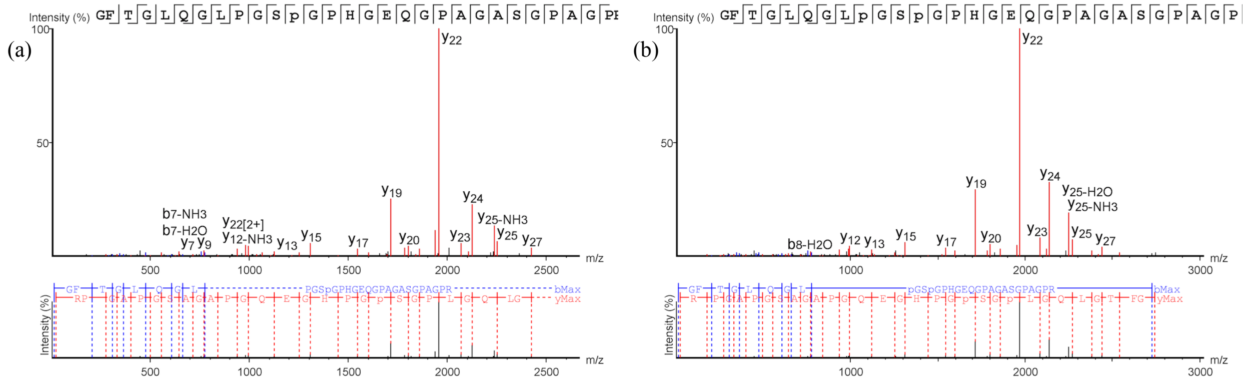

Figure S3.B6. Sturgeon *de novo* peptide X.GFTGLQGLPGSPGPHGEQGPAGASGPAGPR.X (collagen type I alpha 1) in sample 1902-1-233: with (a) one hydroxylation (P), score -l0lgP=72.15, *m/z*=909.7841, z=3, ppm=5.4, # PSM=4, and (b) two hydroxylation (P), score -l0lgP=75.54, *m/z*=915.1176, z=3, ppm=-7.3, # PSM=8

Table S3.B5. Alignment of the main *Acipenser* collagen type I alpha 2 chains identified in skin samples: *Acipenser ruthenus* XP_033848627.2 and XP_033855900.1, and *Acipenser schrenckii* BAT51012.1 using NCBI Blast alignment tool https://blast.ncbi.nlm.nih.gov/. Amino acid substitutions, compared to the top sequence XP_034775076.1, are indicated in red font. Highlighted are the most commonly identified peptides used as markers. Green: markers found in all three sequences; Blue: markers found in both *A. schrenckii* and *A. ruthenus*; Yellow: markers found in *A. schrenckii* only, White: markers found in *A. ruthenus* only. New peptides identified by de novo are highlighted in Grey.

XP_033848627.2	1	MLSFVDTRTLLLLAVTSCLATCQYTQNLEGPKGPRGDKGPMGDRGPQGPNGRDGEPGTPG
XP_033855900.1	1	MLSFVDTRIVLLLAVTSYLATCQYTQNLEGPQGPRGDKGPMGDTGSQGPNGRDGVPGTPG
BAT51012.1	1	MLSFVDTRIVLLLAVTSYLATCQYTQNLEGPKGPRGDKGPMGDRGPQGPNGRDGEPGTPG
XP_033848627.2	61	PAGPPGPPGLGGNFAAQYKAPDAGPGPMGLMGPRGPPGPPGPPGAQGFQGHPGEPGEPGQ
XP_033855900.1	61	PAGPPGPPGLGGNFAAQYKAPDAGPGPMGLMGARGPQGPTGPPGAQGFQGHPGEPGEPGQ
BAT51012.1	61	PAGPPGPPGLGGNFAAQYKAPDAGPGPMGLMGPRGPSGPPGPPGAQGFQGHPGEPGEPGQ
XP_033848627.2	121	TGPVGARGPTGPPGKAGDDGNPGRSGKPGDRGTGGAQGARGFPGTPGLPGMKGHRGYTGL
XP_033855900.1	121	TGPVGARGPTGPPGKAGDDGNPGRSGKPGDRGTGGAQGARGFPGTPGLPGMKGHRGYTGL
BAT51012.1	121	TGPVGARGPTGPPGKAGDDGNPGRSGKPGDRGTGGAQGARGFPGTPGLPGMKGHRGYTGL
de novo		GFPGTPGLPGLEGHR
XP_033848627.2	181	DGQKGEVGAAGVKGETGARGENGSPGLAGARGISGERGRAGPAGPAGARGSDGNVGPAGP
XP_033855900.1	181	DGSKGDAGATGVKGETGARGENGSPGQAGSRGMSGERGRAGPAGSAGARGSDGNAGPSGP
BAT51012.1	181	DGSKGEVGAAGVKGETGARGENGSPGLAGARGISGERGRAGAAGPAGARGSDGNVGPAGP
XP_033848627.2	241	AGPIGPAGPPGFPGSPGPKGEVGPGGPNGPTGAQGARGEPGAAGAVGPVGPIGNPGNNGL
XP_033855900.1	241	AGPLGPSGPPGFPGSPGPKGEVGLGGPNGPTGAQGARGEPGAAGAVGPVGPIGNPGNNGL
BAT51012.1	241	AGPIGPAGPPGFPGSPGPKGEVGPGGPNGPTGAQGARGEPGAAGAVGPVGPIGNPGNNGL
XP_033848627.2	301	NGPKGSAGLPGVAGAPGFPGPRGGPGPQGPVGAAGPRGNSGDPGPSGPKGDTGSKGPPGN
XP_033855900.1	301	NGPKGTAGLPGVSGAPGFPGPRGGPGPQGPAGAAGLRGNSGDPGPSGPKGDTGSKGPPGD
BAT51012.1	301	NGPKGSAGLPGVAGAPGFPGPRGGPGPQGPAGAAGPRGNSGDPGPSGPKGDTGSKGPPGS
XP_033848627.2	361	AGPQGQQGPAGEEGKRGSTGEAGPPGPAGNRGGRGVPGTRGLPGPDGRGGPVGAPGSRGA
XP_033855900.1	361	SGPQGQHGPAGEEGKRGSPGEAGPPGPAGNRGGRGVPGTRGLPGPDGRGGPMGGPGSRGA
BAT51012.1	361	AGPQGQQGPAGEEGKRGSTGEAGPPGPAGNRGGRGVPGTRGLPGPDGRGGPMGAPGSRGA
de novo		GSPGEAGPSGPAGNR
XP_033848627.2	421	TGPPGVTGPSGDAGRPGEPGLPGSRGYPGNSGKSGPPGKEGPAGANGQDGRSGAPGPAGP
XP_033855900.1	421	TGAPGVTGPSGDAGRPGEPGLPGSRGYPGNSGNSGPQGKEGPAGANGQDGRSGAPGPAGP
BAT51012.1	421	TGPPGVTGPSGDAGRPGEPGLPGSRGYPGNSGKSGPPGKEGPAGVNGQDGRSGAPGPAGP
XP_033848627.2	481	RGQPGNIGFPGPKGPAGLPGKPGEKGPAGPVGARGAPGPDGNTGPQGLPGVAGNAGEKGE
XP_033855900.1	481	RGQPGNIGFPGPKGPAGLPGKPGEKGPAGPVGARGAAGPDGNTGPQGLPGVAGNTGEKGE
BAT51012.1	481	RGQPGNIGFPGPKGPAGLPGKPGEKGPAGPVGARGAPGPDGNTGPQGLPGVAGNTGEKGE
XP_033848627.2	541	QGPAGAPGFQGLPGPAGPAGEAGKVGDRGIAGDSGLPGPAGLRGERGNPGAAGSQGAQGP
XP_033855900.1	541	QGPAGAPGFQGLPGPAGPAGEAGKVGDRGIAGDSGLPGAAGLRGERGNPGAAGSQGAQGP
BAT51012.1	541	QGPAGAPGFQGLPGPAGPAGEAGKVGDRGIAGDSGLPGAAGSRGERGNPGAAGSQGAQGP
de novo		GIAGDSGMPGPAGLR GNPGAAGSQGPQGP
XP_033848627.2	601	AGPRGPSGSPGTDGGKGEPGNVGAVGAAGYQGASGMPGERGAAGIPGGKGEKGETGSKGP
XP_033855900.1	601	AGPRGPSGTPGTDGGKGEPGNTGSAGAAGYQGASGMPGERGTAGIPGGKGEKGETGSRGP
BAT51012.1	601	AGPRGPSGTPGTEGGKGEPGNVGAVGAAGYQGASGMPGERGGAGIPGGKGEKGETGSKGP
de novo		AGPR
XP_033848627.2	661	DGNTGRDGGRGAPGPSGPPGPSGATGDKGESGPNGASGPAGPRGPSGERGEVGPAGAPGF
XP_033855900.1	661	EGNTGRDGGRGAPGPSGPPGPSGAAGDKGESGPNGASGPAGPRGPSGERGEVGPAGSSGF
BAT51012.1	661	DGNTGRDGGRGAPGPSGPPGPSGATGDKGESGPNGASGPAGPRGPSGERGEVGPAGAPGF
XP_033848627.2	721	AGPPGADGQPGARGERGPGGPKGDIGPQGPTGPAGSSGPAGPSGPGGPPGPRGDVGPTGM
XP_033855900.1	721	AGPPGADGQPGARGERGPGGSKGDIGPQGPTGPAGSSGPAGPSGPGGAPGPRGDVGPTGM
BAT51012.1	721	AGPPGADGQPGARGERGPGGPKGDIGPQGPTGPAGSSGPAGPSGPGGPPGPRGDVGPTGM
XP_033848627.2	781	TGFPGSAGRVGGPGPAGISGPPGPPGNAGKDGPRGARGDTGPAGRPGEQGMVGPSGMAGE
XP_033855900.1	781	TGFPGGAGRVGGPGPAGISGPPGPSGNAGKDGPRGARGDTGPAGRPGEQGMVGPSGMAGE
BAT51012.1	781	TGFPGSAGRVGGPGPAGISGPPGPPGNAGKDGPRGARGDSGPAGRPGEQGMVGPSGMAGE

XP_033848627.2	841	KGPSGESGPPGPPGISGPSGVLGSPGIVGLPGSRGDRGLPGGAGGSGESGKLGPAGAQGP
XP_033855900.1	841	KGPSGESGPPGPTGISGPSGVLGSPGIVGLPGSRGDRGLPGGAGGTGEPGGLGPAGSQGQ
BAT51012.1	841	KGPSGESGPPGPPGISGPSGVLGSPGIVGLPGSRGDRGLPGGAGGTGESGKIGPAGAQGP
XP_033848627.2	901	RGAAGNMGAPGMTGAPGETGRDGHPGNDGPPGRNGAPGFKGDRGEGGPAGIAGASGAPGA
XP_033855900.1	901	RGAAGNMGAPGMTGAPGEAGRDGHPGNDGPPGRNGAAGFKGDRGEGGPAGIAGTSGAPGA
BAT51012.1	901	RGAAGNMGAPGMTGAPGEAGRDGHPGNDGPPGRNGAPGFKGDRGEGGPAGIAGASGAPGA
XP_033848627.2	961	PGPAGAAGRPGNRGESGSGGPQGPLGPAGARGISGPQGPRGEKGVAGEKGERGMKGLRGH
XP_033855900.1	961	PGPAGAAGRPGNRGETGSGGAQGPLGPAGARGISGPQGPRGEKGVAGEKGERGMKGLTGH
BAT51012.1	961	PGPAGAAGRPGNRGESGSGGPQGPLGPAGARGISGPQGPRGEKGVAGEKGERGMKGLRGH
de novo		GEPGSGGAQGPLGPAGAR
XP_033848627.2	1021	SGMQGLPGPSGPSGEQGSAGQTGPSGPRGPAGPSGPPGKDGQPGHPGPIGPAGSRGPNGQ
XP_033855900.1	1021	SGMQGLPGPSGPSGEQGSSGQTGPSGPRGPAGPSGPPGKDGQPGHPGPIGQAGSRGPNGQ
BAT51012.1	1021	TGMQGLPGPSGASGEQGSAGQTGPSGPRGPAGPSGPPGKDGQPGHPGPIGPAGSRGPNGQ
XP_033848627.2	1081	SGPTGPPGSPGLPGLPGISGGGYEVSGYEGEYRADQATQKAKDYEIGATLKSLNNQINNL
XP_033855900.1	1081	SGPTGPPGSPGLPGLPGISGGGYEVSGYEGEYRADQAAQKAKDYEIGATLKSLNNQIDNL
BAT51012.1	1081	SGPTGPPGSPGLPGLPGISGGGYEVSGYEGEYRADQATQKAKDYEIGATLKSLNNQINNL
XP_033848627.2	1141	LTPEGSKKNPARTCRDIRLSHPEWSSGYYWIDPNQGCIMDAIKVHCDFSTGETCIYPNPA
XP_033855900.1	1141	LTPEGSKKNPARTCRDIRLSHPEWSSGYYWIDPNQGCIMDAIKVHCDFSTGETCIYPNPA
BAT51012.1	1141	LTPEGSKKNPARTCRDIRLSHPEWSSGYYWIDPNQGCIMDAIKVHCDFSTGETCIYPNPA
XP_033848627.2	1201	SIPRKNWYRNAKDKKHTWFGETINGGTQFEYNDESMTPQTMATQLAFMRLLANQASQNIT
XP_033855900.1	1201	SIPRKNWYRNAKDKKHTWFGETINGGTQFEYSDESMTPQTMATQLAFMRLLANQASQNIT
BAT51012.1	1201	SIPRKNWYRNAKDKKHTWFGETINGGTQFEYNDESMTPQTMATQLAFMRLLANQASQNIT
XP_033848627.2	1261	YHCKNSIAYMDAEAGNLKKAVLLQGSNDVELRAEGNSRFTFSVLEDDCTRHTGQWGKTVI
XP_033855900.1	1261	YHCKNSIAYMDAEAGNLKKAVLLQGSNDVELRAEGNSRFTFSVLEDDCTRHTGQWGKTVI
BAT51012.1	1261	YHCKNSIAYMDAEAGNLKKAVLLQGSNDVELRAEGNSRFTFSVLEDDCTRHTGQWGKTVI
XP_033848627.2	1321	QYKTTKPSRLPILDIAPLDIGGADQEFGLDIGPVCFK
XP_033855900.1	1321	QYKTTKPSRLPILDIAPLDIGGADQEFGLDIGPVCFK
BAT51012.1	1321	QYKTTKPSRLPILDIAPLDIGGADQEFGLDIGPVCFK

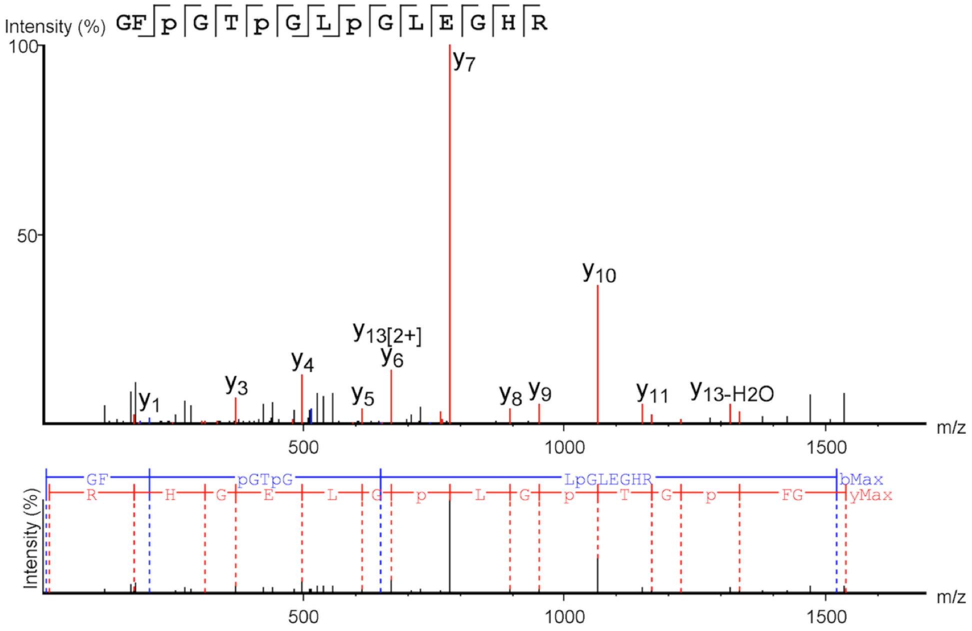

Figure S3.B7. Sturgeon *de novo* peptide X.GFPGTPGLPGLEGHR.X (collagen type I alpha 2) in sample 1862:16 I: with three hydroxylation (P), score -10lgP=45.41, *m/z*=513.9243, z=3, ppm=2.2, # PSM=7

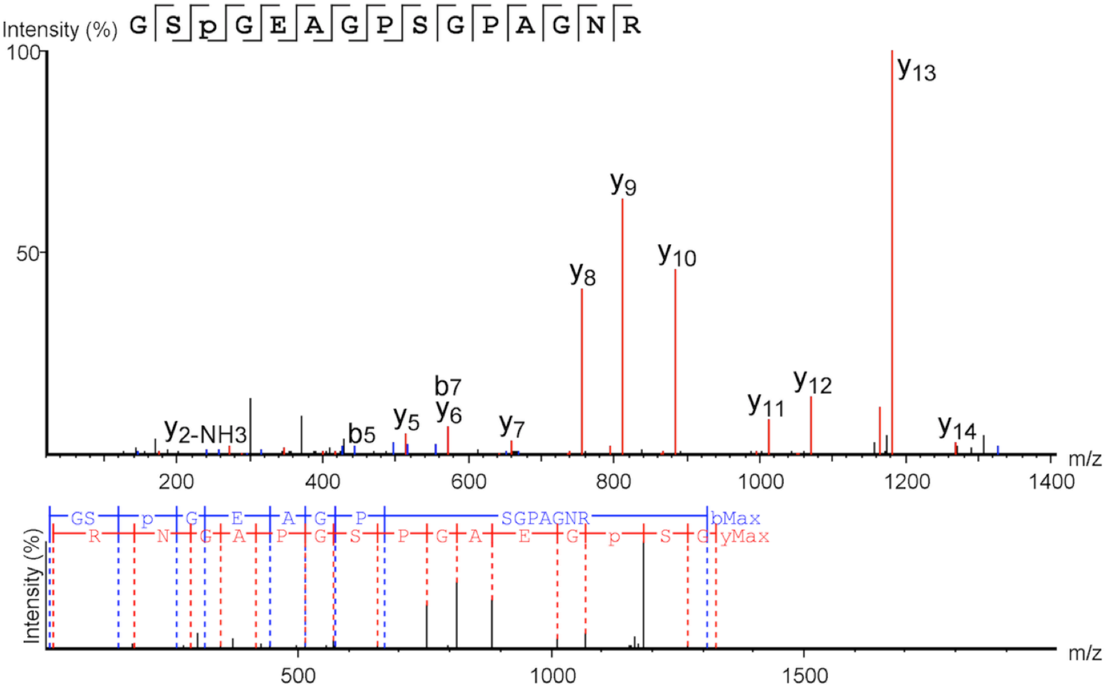

Figure S3.B8. Sturgeon *de novo* peptide X.GSPGEAGPSGPAGNR.X (collagen type I alpha 2) in sample 1862:16 I: with one hydroxylation (P), score -10lgP=53.39, *m/z*=663.8036, z=2, ppm=-2.5, # PSM=4

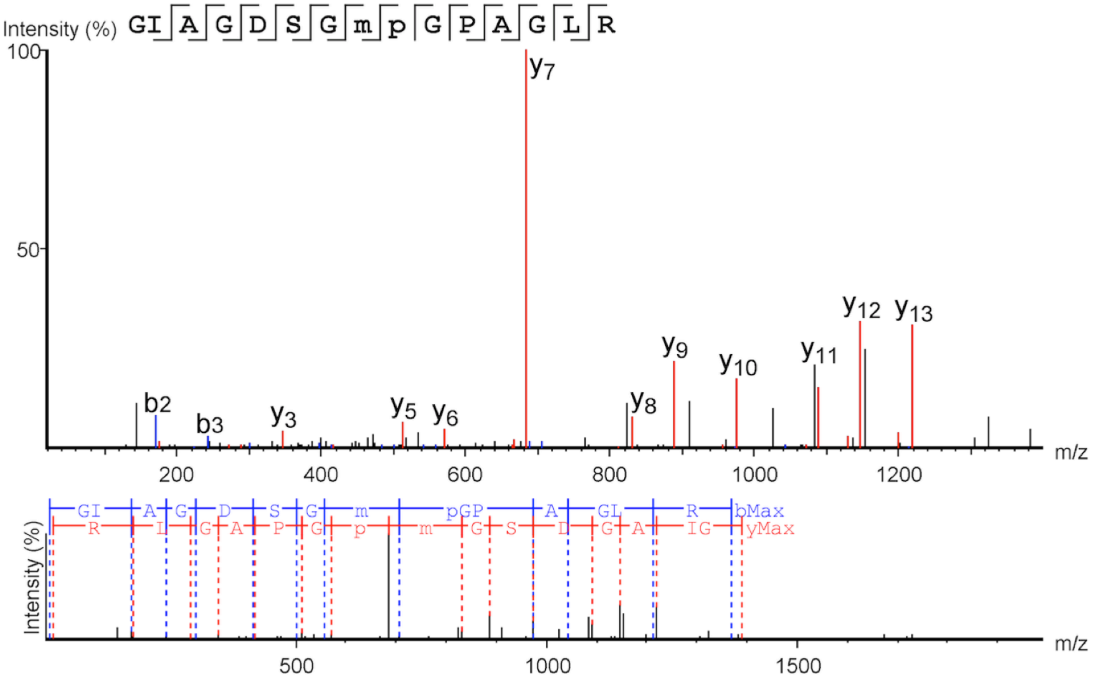

Figure S3.B9. Sturgeon *de novo* peptide X.GIAGDSGMPGPAGLR.X (collagen type I alpha 2) in sample D13a: with one hydroxylation (P) and oxidation (M), score -10lgP=58.78, *m/z*=694.3377, z=2, ppm=-3.4, # PSM=6

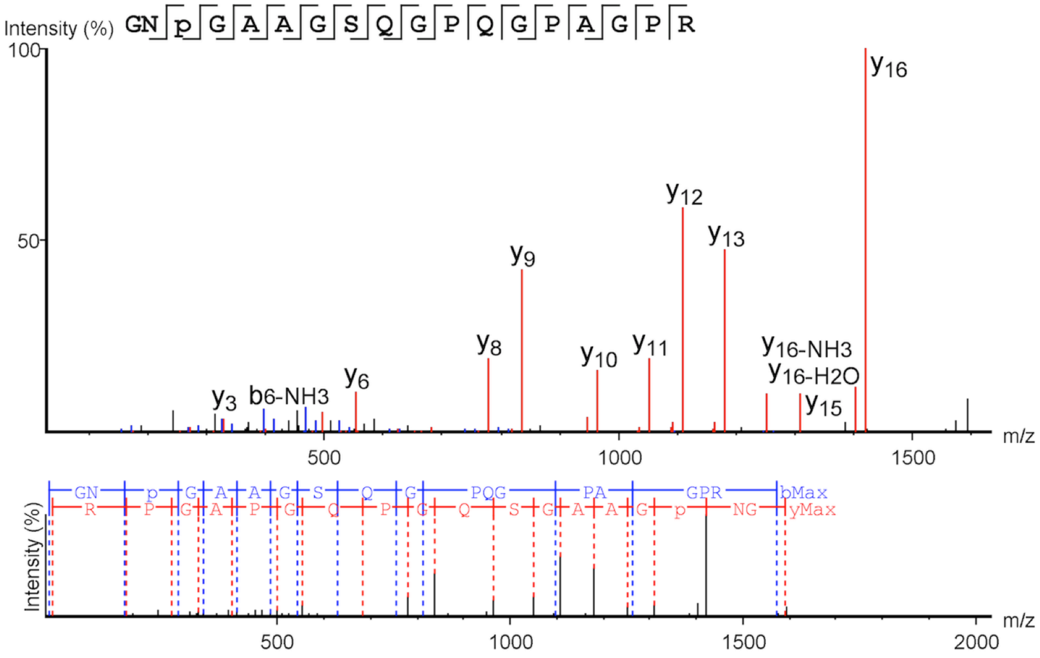

Figure S3.B10. Sturgeon *de novo* peptide X.GNPGAAGSQGPQGPAGPR.X (collagen type I alpha 2) in sample 1902-1-262_3: with one hydroxylation (P), score -l0lgP=62.83, *m/z*=796.3896, z=2, ppm=9.3, # PSM=6

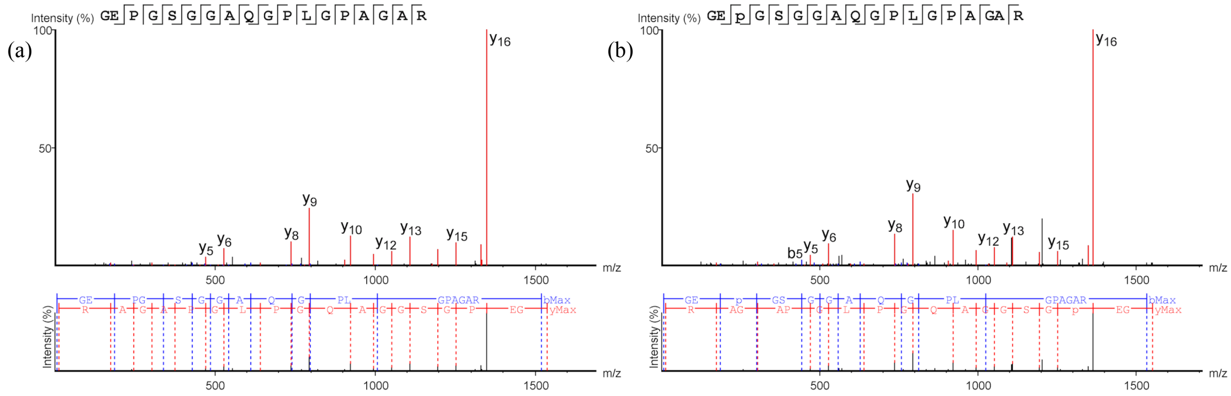

Figure S3.B11. Sturgeon *de novo* peptide X.GEPGSGGAQGPLGPAGAR.X (collagen type I alpha 2) in sample 1902-1-285_1: (a) score -l0lgP=53.52, *m/z*=768.3857, z=2, ppm=5.2, # PSM=6 and (b) with one hydroxylation (P), score -l0lgP=50.57, *m/z*=776.3838, z=2, ppm=6.0, # PSM=6

Identification of wheat and barley proteins

Cereal proteins were found in two samples, **1862:16 V** and **1965-33-5_1**. In the latter, only two peptides (one protein) was identified, corresponding to wheat (*Triticum* sp.). These peptides were likely deposited during the use of the textiles, rather than being part of the making of the metal threads, as they constitute a minor component. The identification of wheat/barley in **1862:16 V**, with 11 peptides, is more convincing as part of the thread. Both *Triticum* sp. (wheat) and *Hordeum* sp. (barley) peptides were found. For unique peptides Blast results: See excel file **SI_Proteomics Identification in Skins (Cereal peptides identification).** The spectra of B3-hordein (Fragment) peptide R.IVPLAIDTR.V, Alpha-amylase/trypsin inhibitor CM3 peptide R.LLVAPGQCNLATIHNVR.Y, Beta-amylase peptide R.YDPTAYNTILR.N and Gamma gliadin-D4 peptide R.APFASIVAGIGGQ.- are shown in **Figure S3.B12a-d.**

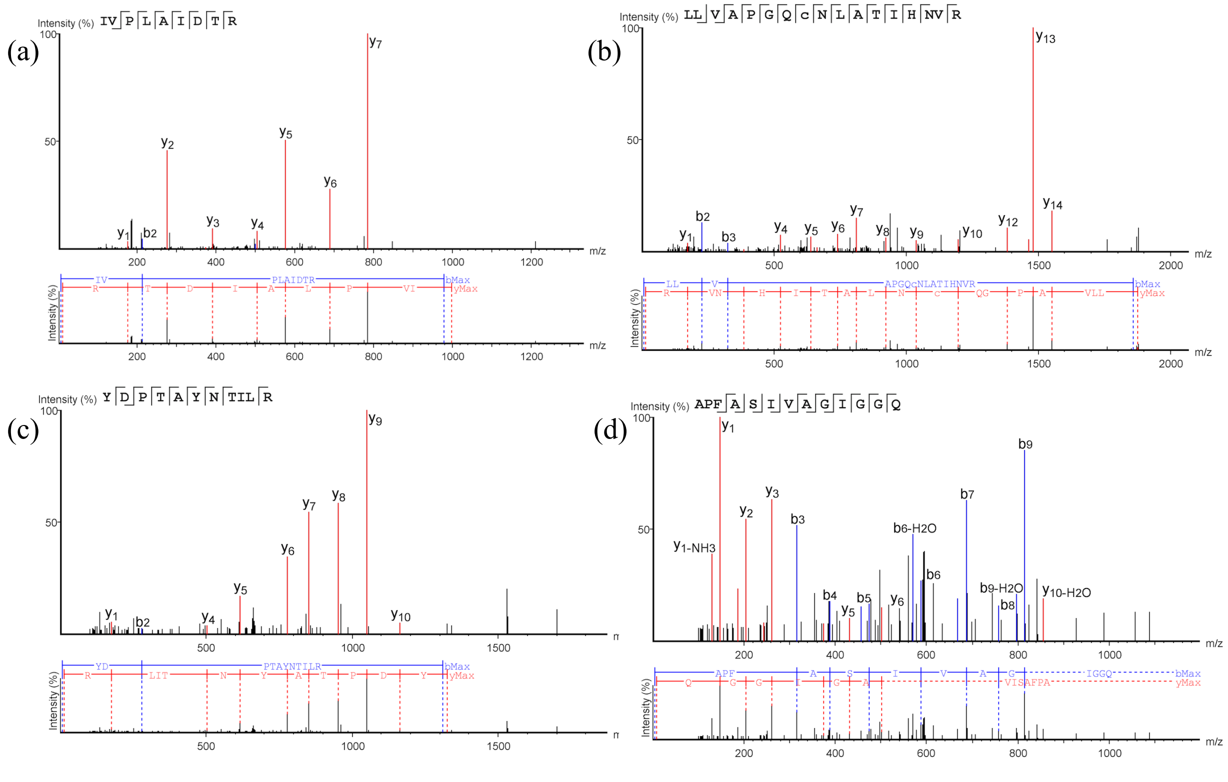

Figure S3.B12. Wheat peptide markers in 1862:16 V. a) B3-hordein (Fragment) peptide R.I_{253}VPLAIDTR_{261}.V, score -l0lgP=35.47, *m/z*=499.3070, z=2, ppm=2.5, # PSM=10**; b)** Alpha-amylase/trypsin inhibitor CM3 peptide R.L_{141}LVAPGQCNLATIHNVR_{157}.Y, score -l0lgP=49.06, *m/z*=626.0130, z=3, ppm=1.7, # PSM=6**; c)** Beta-amylase peptide R.Y_{56}DPTAYNTILR_{66}.N, score -l0lgP=46.71, *m/z*=663.8400, z=2, ppm=3, # PSM=6; **d)** Gamma gliadin-D4 peptide R.APFASIVAGIGGQ.- , score -l0lgP=43.28, *m/z*=594.3250, z=2, ppm=1, # PSM=6

Identification of Milk proteins

Milk was found in four skin samples: **D12b, D13a, 1862:16 I** and **1902-1-310_1**, but in each case only one protein with two peptides were identified (**Table S3.B5**). In contrast, in the blank, four proteins were identified for a total of 12 peptides, and at least one unique *Bos* sp. peptide in each protein. This likely comes from peptide carry-over in the instrument due to previous analysis of cow milk. All samples, except for **D13a**, have cow peptides, most of them also found in the blank. Only **D13a** has two sheep/goat milk peptides not identified in the blank (**Figures S3.B3a and b**), suggesting these might be genuine peptides from the sample. However, one protein with two peptides makes the identification of milk as part of the adhesive unconvincing and more likely a surface contamination during the use history of the textile.

Table S3.B5. Milk peptides. Peptides allowing identification of the different milk proteins in *Bos sp.*, *Ovis aries* and *Capra hircus*

PROTEIN	SEQUENCE	BLANK	SKIN THREADS	BLAST (BOS/OVIS/CAPRA)
**P02666|CASB_BOVIN BETA-CASEIN OS=BOS TAURUS**	IHPFAQTQ	YES	-	*Bos* sp.
	VLPVPQK	YES	-	*Bos* sp., *Ovis aries, Capra hircus*
	AVPYPQR	YES	-	*Bos* sp.
	DMPIQAFLLYQEPVLGPVR	YES	1902-1-310_1	*Bos* sp., *Ovis aries, Capra hircus*
	LLYQEPVLGPVR	YES	1902-1-310_1	*Bos* sp., *Ovis aries, Capra hircus*
**P02662|CASA1_BOVIN ALPHA-S1-CASEIN OS=BOS TAURUS**	HQGLPQEVLNENLLR	YES	-	*Bos* sp.
	FFVAPFPEVFGK	YES	D12b; 1862:16 I	*Bos* sp.
	YLGYLEQLLR	No	D12b; 1862:16 I	*Bos* sp., *Ovis aries, Capra hircus*
**P18626|CASA1_CAPHI ALPHA-S1-CASEIN OS=CAPRA HIRCUS**	FVVAPFPEVFR	No	D13a	*Ovis aries, Capra hircus*
	YNVPQLEIVPK	No	D13a	*Ovis aries, Capra hircus*
**P02663|CASA2_BOVIN ALPHA-S2-CASEIN OS=BOS TAURUS**	ALNEINQFYQK	YES	-	*Bos* sp., *Ovis aries, Capra hircus*
	NAVPITPTLNR	YES	-	*Bos* sp.
	FALPQYLK	YES	-	*Bos* sp.
**P02668|CASK_BOVIN KAPPA-CASEIN OS=BOS TAURUS**	YIPIQYVLSR	YES	-	*Bos* sp., *Ovis aries, Capra hircus*
	SPAQILQWQVLSNTVPAK	YES	-	*Bos* sp.

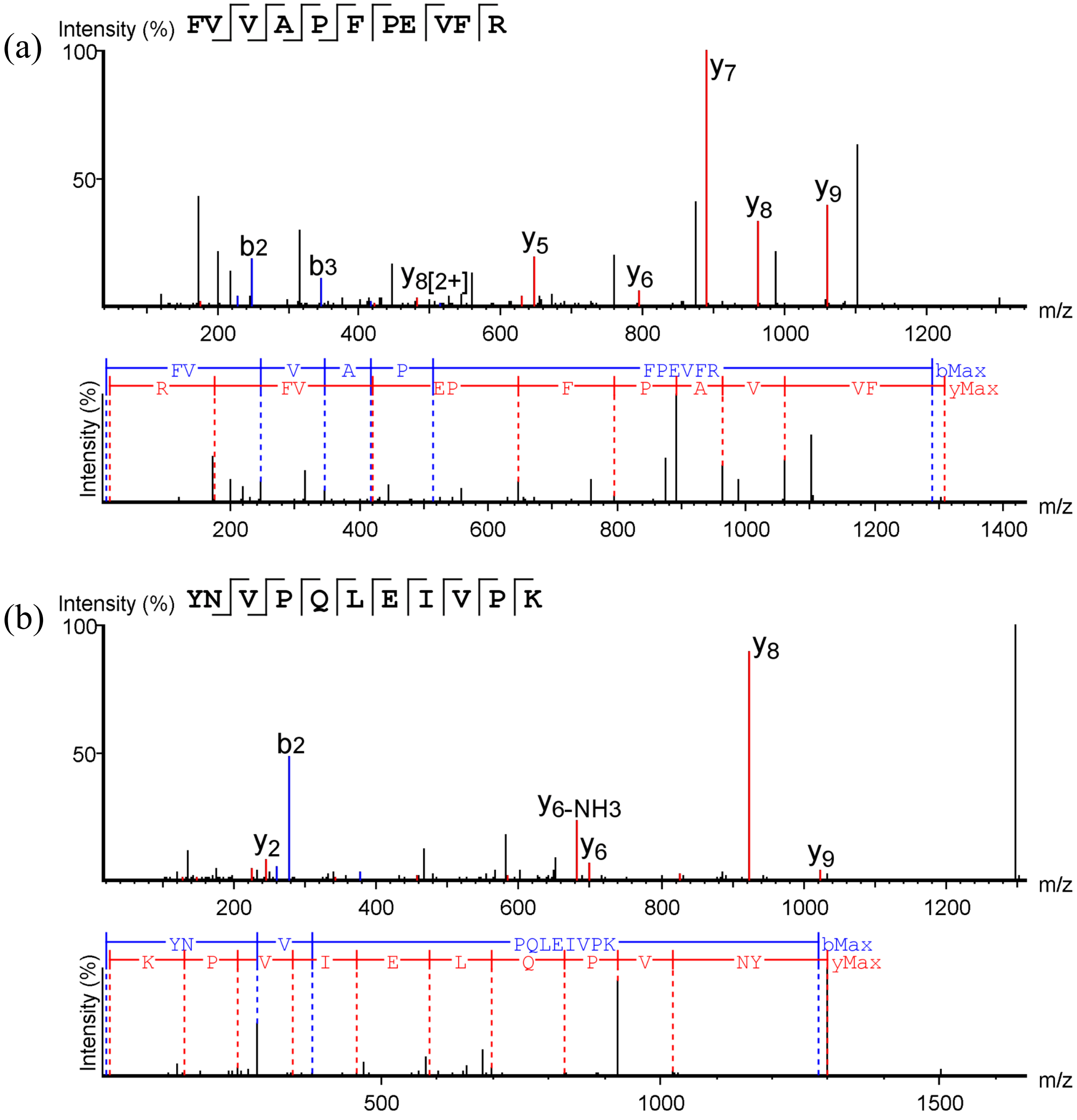

Figure S3.B13. Milk peptide markers in D13a. **a)** Ovicapra alpha-S1-casein peptide R.F_{38}VVAPFPEVFR_{48}.K, score -l0lgP=32.50, *m/z*=654.3644, z=2, ppm=5.3, # PSM=5; **b)** Ovicapra alpha-S1-casein peptide K.Y_{119}NVPQLEIVPK_{129}.S, score -l0lgP=45.03, *m/z*=650.3716, z=2, ppm=4.1, # PSM=4

Identification of human and fungi proteins

Two samples, **1902-1-229b_1** and **P4d**, had an above average percentage of *Others* proteins (**Figure S3.B1**). In both cases, the proteins are best matched to *Homo sapiens*. The human protein identification is shown in **Table S3.B6** for sample **1902-1-229b_1** and in **Table S3.B7** for sample **P4d**. In addition, fungi proteins were also identified in sample **P4d** and these are shown in **Table S3.B8**. For unique peptides Blast results: See excel file **SI_Proteomics Identification in Skins (Human and Fungi peptides identification).**

Sample **1902-1-229b_1** is characterized by proteins found in the dermis and epidermis. Besides the cytoskeletal keratins (included in **Table S3.B2**) which usually come from handling contamination, the proteins with the highest protein coverage are the S100 proteins, hemoglobins, caspase-14 and calmodulin. Caspase-14 is a cysteine-aspartic acid protease, expressed in the epidermis, that plays a role in terminal keratinocyte differentiation [5]. The protein is highly enriched in human skin [6], where it is only expressed in the differentiating and cornifying layers of the epidermis [5], i.e. in the upper layers of the epidermis. Calmodulin-like protein 5 is a calcium-binding protein, and like Caspase-14, is expressed in the epidermis [6] and plays a role in differentiating keratinocytes [7]. The S100 proteins are all found in the keratinocytes of the epidermis [8]: for S100A7 (psoriasin), tissues enriched with this protein are limited to skin and tonsils, while S100A8 and A9 are more largely distributed [6]. S100A8 and A9 are co-expressed to form heterodimers; in normal epidermis their level is very low but become high in response to epidermal injury as well as various skin stresses (inflammation, disease, etc) [8-10]. S100A7 is present in normal epidermis but is released at high levels in wound exudates where it has been shown to have antibacterial activity [11]. All three S100 proteins are also overexpressed in psoriasis disease [9,10,12].

It should be noted that S100A7 is not present in blood [11], whilst caspase-14 is downregulated in psoriatic lesions [5,13]. Other proteins (hemoglobin, haptoglobin, serum albumin, etc) indicate the presence of blood. The spectra of Caspase-14 peptide R.EGSEEDLDALEHMFR.Q, protein S100-A7 peptide K.GTNYLADVFEK.K, and hemoglobin subunit-beta peptide K.VLGAFSDGLAHLDNLK.G are shown in **Figure S3.B14a-c.**

This textile was part of a donation in 1902 of three textile collections from Europe from J.P. Morgan to the Hewitt sisters for the Cooper Union Museum that would later become the Cooper Hewitt, Smithsonian Design Museum [14]. Textiles were placed behind display cases to allow students to look at them. The good preservation of the hemoglobin proteins could indicate a recent contamination; these proteins have so far rarely been found in old samples. In the recent identification of human blood on a 1000-year old gold mask from Peru, for example, hemoglobin was not found [15]. It was not found either in early Iron Age mortuary vessels in which a large range of other human proteins were identified [16]. Hemoglobin from sacrificial blood from multiple animal species was found with high coverage on 19-20th c African objects [17], but not among the 108 human serum proteins from a blood spot on Anton Chekhov's death shirt (1904) [18]. We cannot exclude however that the proteins were deposited during the making of the thread and survived below the metal layer.

Table S3.B6. Human proteins identified in sample 1902-1-229b_1: searched against *UniProtKB/Swiss-Prot reviewed* database, with a FDR of 1%, a protein score -10lgP ≥ 50, a minimum of two peptides and **one** unique peptide. -10lgP is Peaks score; TOTAL PEPT. is total number of peptides; U. is number of unique peptides in PEAKS; % is protein percentage coverage and # PSM is number of peptide-to-spectrum matches

PROTEIN GROUP	*ACCESSION NUMBER*	*-10lgP*	*%*	*TOTAL PEPT.*	*U.*	*# PSM*	*AVG. MASS*	*DESCRIPTION*
61	P35527|K1C9_HUMAN	208.68	53	21	20	106	62064	Keratin type I cytoskeletal 9
62	P13645|K1C10_HUMAN	189.07	38	16	6	106	58827	Keratin type I cytoskeletal 10
68	P04264|K2C1_HUMAN	166.67	29	19	12	97	66039	Keratin type II cytoskeletal 1
77	P02768|ALBU_HUMAN	166.06	26	14	6	58	69367	Serum albumin
73	P02538|K2C6A_HUMAN	164.01	38	18	2	67	60045	Keratin type II cytoskeletal 6A
53	P02461|CO3A1_HUMAN	162.04	15	28	3	138	138564	Collagen alpha-1(III) chain
78	P04259|K2C6B_HUMAN	155.67	37	17	1	58	60067	Keratin type II cytoskeletal 6B
79	P35908|K22E_HUMAN	147.18	28	11	5	42	65433	Keratin type II cytoskeletal 2 epidermal
80	P06702|S10A9_HUMAN	123.92	56	7	7	48	13242	Protein S100-A9
84	P68871|HBB_HUMAN	118.01	54	6	3	38	15998	Hemoglobin subunit beta
88	P69905|HBA_HUMAN	116.41	62	6	2	30	15258	Hemoglobin subunit alpha
92	P08779|K1C16_HUMAN	105.98	18	7	3	20	51268	Keratin type I cytoskeletal 16
97	P02647|APOA1_HUMAN	101.41	25	5	2	18	30778	Apolipoprotein A-I
101	P31944|CASPE_HUMAN	97.03	32	5	5	15	27680	Caspase-14
91	P02533|K1C14_HUMAN	95.62	21	8	4	19	51562	Keratin type I cytoskeletal 14
113	P0DOX5|IGG1_HUMAN	92.48	10	3	3	10	49329	Immunoglobulin gamma-1 heavy chain
	P01857|IGHG1_HUMAN	92.48	14	3	3	10	36106	Immunoglobulin heavy constant gamma 1
125	P00738|HPT_HUMAN	76.13	10	4	4	8	45205	Haptoglobin
99	P31151|S10A7_HUMAN	75.94	22	2	2	16	11471	Protein S100-A7
137	Q9NZT1|CALL5_HUMAN	73.96	32	3	3	7	15893	Calmodulin-like protein 5
107	P05109|S10A8_HUMAN	72.17	31	3	3	14	10835	Protein S100-A8
121	P05997|CO5A2_HUMAN	62.86	3	4	4	9	144910	Collagen alpha-2(V) chain
120	P01834|IGKC_HUMAN	59.23	30	2	2	9	11765	Immunoglobulin kappa constant
	P0DOX7|IGK_HUMAN	59.23	15	2	2	9	23379	Immunoglobulin kappa light chain
209	P0CG04|IGLC1_HUMAN	55.59	14	1	1	4	11348	Immunoglobulin lambda constant 1

PROTEIN GROUP	*ACCESSION NUMBER*	*-10lgP*	*%*	*TOTAL PEPT.*	*U.*	*# PSM*	*AVG. MASS*	*DESCRIPTION*
	P0DOY2|IGLC2_HUMAN	55.59	14	1	1	4	11294	Immunoglobulin lambda constant 2
	P0DOY3|IGLC3_HUMAN	55.59	14	1	1	4	11266	Immunoglobulin lambda constant 3
	P0CF74|IGLC6_HUMAN	55.59	14	1	1	4	11277	Immunoglobulin lambda constant 6
	A0M8Q6|IGLC7_HUMAN	55.59	14	1	1	4	11254	Immunoglobulin lambda constant 7
	P0DOX8|IGL1_HUMAN	55.59	7	1	1	4	22830	Immunoglobulin lambda-1 light chain
	B9A064|IGLL5_HUMAN	55.59	7	1	1	4	23063	Immunoglobulin lambda-like polypeptide 5
208	P01024|CO3_HUMAN	50.46	2	2	2	3	187147	Complement C3

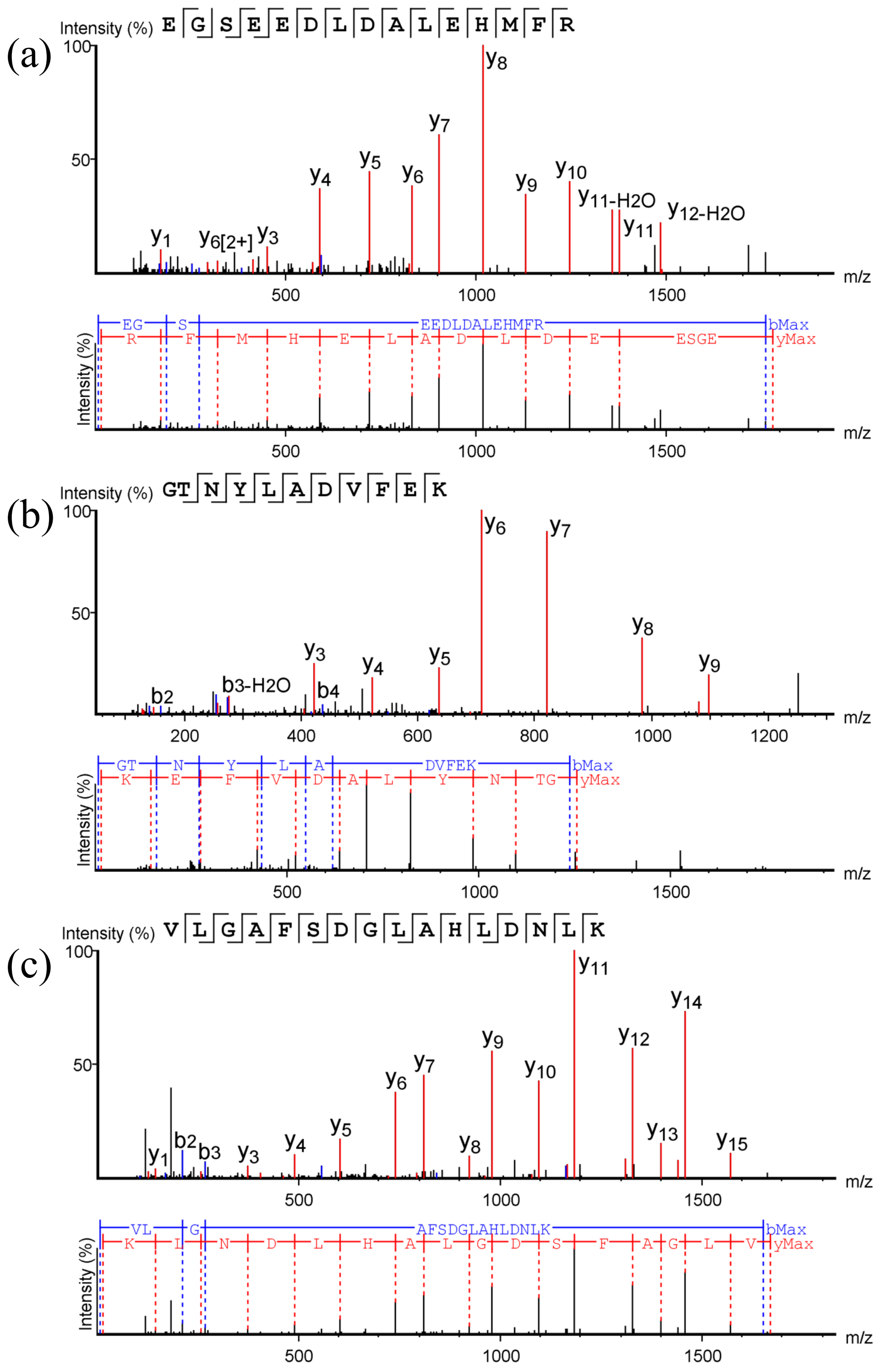

Figure S3.B14. Human peptide markers in 1902-1-229b_1. a) Caspase-14 peptide R.E_{30}GSEEDLDALEHMFR_{44}.Q, score -10lgP=42.87, *m/z*=593.2629, z=3, ppm=2.6, # PSM=6**; b)** Protein S100-A7 peptide K.G_{51}TNYLADVFEK_{61}.K, score -10lgP=52.08, *m/z*=628.8128, z=2, ppm=2.1, # PSM=6**; c)** Hemoglobin subunit-beta peptide K.V_{67}LGAFSDGLAHLDNLK_{82}.G, score -10lgP=52.41, *m/z*=557.3026, z=3, ppm=1.4, # PSM=6

The set of human proteins found in **P4d** is different from **1902-1-229b_1**, indicating that a different scenario happened here to account for the human contamination. Sample **P4d** is characterized by acute-phase proteins of the immune system, mainly alpha-1-antitrypsin, alpha-1-antichimotrypsin, alpha-2-macroglobulin, complement C3 and C4, fibrinogen, ceruloplasmin and haptoglobin (**Table S3.B7**) which are positive acute-phase proteins, i.e. their concentration increases in the plasma in response to inflammation and infection [19,20]. The spectra of alpha-1-antichymotrypsin peptide K.ITLLSALVETR.T, immunoglobulin alpha-2 heavy chain peptide R.WLQGSQELPR.E, and hemopexin peptide K.NFPSPVDAAFR.Q are shown in **Figure S3.B15a-c.**

The fungi proteins were matched to a few species rather than one, and the peptides identified are largely distributed among fungi species (**SI_Proteomics Identification in Skins (Fungi peptides identification)**). The best hit (with the highest number of peptide matches: **Table S3.B8**) was for *Neurospora crassa,* a fungi first reported after an orange bread mold infestation in French bakeries in the hot and humid summer of 1842 [21]; its growth if facilitated by humidity and heat and affects other food and plant products but it is not known to cause disease or infection in humans [22]. *Blumeria graminis* is a fungus that causes powdery mildew on cereals and grasses, with a worldwide distribution [23]. Of the two peptides matched to *Blumeria graminis*, one (GPTLLEDFIFR) is found in both barley and wheat forms but the second one (FGFDLLDPTK) is only found in the barley form, hence the match to *Blumeria graminis* f. sp. *hordei* specifically, which is restricted to barley for host. The fungus causes major damage on crops, with decrease in yield and quality of barley [24].

However, most matches belong to the *Aspergillus* genus, with *Emericella nidulans* corresponding to *Aspergillus nidulans* (sexual/asexual stage) [25]. In addition, all the fungi peptides identified are found in the *Aspergillus* genus (see **SI_Proteomics Identification in Skins**), a genus of common and widespread molds of which there are more than 180 different types [26], some of them affect foods such as grain [25]. Some *Aspergillus* species (about 40 [26]) are responsible for a range of animal diseases: through the production of mycotoxins (e.g. aflatoxins) (i), or aspergillosis, of which there are several forms: allergic (ii), chronic and invasive (iii) [26]. Infections mostly affect people who are immunocompromised people or have an underlying condition such as asthma [25].

(i) Aflatoxins have been found on all major crops and are mainly produced by *A. flavus* and *A. parasiticus* during crop production, harvest, storage and food processing. Exposure to aflatoxins can cause acute liver failure with symptoms such as vomiting and bleeding [27].

(ii) Pre-disposed individuals can develop allergic reactions to *Aspergillus* by inhalation of the spores or from fungi-contaminated food [25]. Hypersensitivity pneumonitis, aspergillus-mediated asthma and allergic bronchopulmonary aspergillosis (ABPA, which occurs in non-immunocompromised patients) are allergic diseases induced by Aspergillus [28]. ABPA can result in coughing blood or mucus [26]. A type of extrinsic allergic alveolitis or occupational mycosis is the Malt worker's lung; it is due to inhalation of spores from *A. clavatus* and *A. fumigatus* from contaminated barley, causing lung inflammation [25].

(iii) Individuals with compromised immune systems can develop pulmonary infections. Chronic infection can occur to people with conditions such as asthma or tuberculosis [25]. Aspergillosis takes multiple forms, of which aspergilloma (fungus ball) is manifested by a mass of mold in the lung, resulting in coughing blood in advanced stage, while invasive aspergillosis spreads throughout the body. *A. nidulans* and *A. fumigatus* have been found to cause invasive pulmonary aspergillosis [29,30].

The taxonomic identification of the fungi peptides to the *Aspergillus* species is shown in **SI_Proteomics Identification in Skins (Fungi peptides identification)** using the Basic Local Alignment Search Tool (https://blast.ncbi.nlm.nih.gov/Blast.cgi). None of the key species mentioned above were found to match all the peptides, so there is not one unique species that could account for all fungi peptides. The spectra of ATP synthase subunit beta mitochondrial peptide K.VVDLLAPYAR.G, 14- 3-3 protein peptide K.DSTLIMQLLR.D, Formate dehydrogenase peptide R.GYGGDVWFPQPAPK.E are shown in **Figure S3.B16a-c.**

The many conditions that fall under aspergillosis are typically characterized by hemoptysis (coughing blood) and sputum (coughing mucus). The presence of *Aspergillus* species in **P4d** with acute phase proteins from the immune system is unlikely to be coincidental and seems to indicate a response to an inflammation due to the fungus, perhaps an allergic reaction to the spores. The presence of these proteins on the threads might be the result of coughing blood due to an infection in the lungs. Most human proteins identified in **P4d** were similarly found, together with proteins from the *Mycobacterium tuberculosis*, in the analysis of a blood spot from Chekhov's shirt [18]. Chekhov, who died from tuberculosis, likely shed these proteins from coughing blood too. Since the proteomics data are inconclusive as to which species of *Aspergillus* is present, or the possibility that multiple species are present, it is difficult to conclude on the exact pathology responsible for the immunological reaction.

Aspergillus has been reported to cause damage on parchment [31], the presence of fungal hyphae was indeed visible by SEM-BSE imaging of sample **P4d**. Conversely, sample **P4c**, from another fragment of the same fabric, did not contain fungi proteins, excluding mold infestation on the threads themselves. Both samples **P4c** and **P4d** are from patches used to repair a damaged cope. An inventory of 1885 mentions that "Cope No. P4 [the cope dates from the beginning of the 16th c.] contains patches of an older fabric, the largest of which has been detached and deposited in drawer XVIII. It is a green silk fabric in which a very dense pattern consisting only of plant motifs is woven in with gilded straps". Another inventory from 1656 lists "old and dilapidated copes, dalmatics and a curtain of green gold fabric that were already out of use". These liturgical textiles have not survived but may have provided the material for these patches. Based on these information, the human contamination likely pre-dates the inventory of 1885, and the storage of the patch in a drawer, away from the light, has likely contributed to the preservation of these proteins. **Figure S3.B17** shows the widespread presence of deposits on the surface of sample **P4d**, most likely of biological nature, which might indeed be associated with fungal hyphae.

Table S3.B7. Human proteins identified in sample P4d: searched against *UniProtKB/Swiss-Prot reviewed* database, with a FDR of 1%, a protein score -10lgP ≥ 50, a minimum of two peptides and **one** unique peptide. -10lgP is Peaks score; TOTAL PEPT. is total number of peptides; U. is number of unique peptides in PEAKS; % is protein percentage coverage and # PSM is number of peptide-to-spectrum matches

PROTEIN GROUP	*ACCESSION NUMBER*	*-10lgP*	*%*	*TOTAL PEPT.*	*U.*	*# PSM*	*AVG. MASS*	*DESCRIPTION*
30	P04264|K2C1_HUMAN	239.2	60	41	19	284	66039	Keratin type II cytoskeletal 1
38	P13645|K1C10_HUMAN	214.72	55	30	17	183	58827	Keratin type I cytoskeletal 10
41	P35527|K1C9_HUMAN	215.1	44	25	24	171	62064	Keratin type I cytoskeletal 9
44	P02768|ALBU_HUMAN	206.64	39	26	24	155	69367	Serum albumin
45	P02538|K2C6A_HUMAN	172.58	43	23	2	139	60045	Keratin type II cytoskeletal 6A
57	P13647|K2C5_HUMAN	135.56	23	13	2	85	62378	Keratin type II cytoskeletal 5
59	P02461|CO3A1_HUMAN	145.41	9	12	1	82	138564	Collagen alpha-1(III) chain
62	P01009|A1AT_HUMAN	158.47	42	19	5	77	46737	Alpha-1-antitrypsin
65	P08779|K1C16_HUMAN	177.48	41	18	5	77	51268	Keratin type I cytoskeletal 16
66	P02647|APOA1_HUMAN	167.05	43	14	11	67	30778	Apolipoprotein A-I
72	P01024|CO3_HUMAN	187.63	15	19	19	59	187147	Complement C3
80	P35908|K22E_HUMAN	129.09	14	9	2	56	65433	Keratin type II cytoskeletal 2 epidermal
81	P02533|K1C14_HUMAN	148.17	33	16	4	57	51562	Keratin type I cytoskeletal 14
86	P01023|A2MG_HUMAN	150.64	12	13	12	51	163290	Alpha-2-macroglobulin
89	P00738|HPT_HUMAN	133.91	24	11	11	46	45205	Haptoglobin
94	P02787|TRFE_HUMAN	137.25	14	9	8	41	77064	Serotransferrin
96	Q04695|K1C17_HUMAN	104.81	15	8	1	32	48106	Keratin type I cytoskeletal 17
98	P0DOX5|IGG1_HUMAN	127	16	7	6	30	49329	Immunoglobulin gamma-1 heavy chain
	P01857|IGHG1_HUMAN	127	22	7	6	30	36106	Immunoglobulin heavy constant gamma 1
101	P02790|HEMO_HUMAN	121.93	20	8	8	26	51676	Hemopexin
102	Q7Z3Y8|K1C27_HUMAN	81.95	8	4	1	24	49822	Keratin type I cytoskeletal 27
111	P0C0S8|H2A1_HUMAN	61.28	26	3	2	12	14091	Histone H2A type 1
	P04908|H2A1B_HUMAN	61.28	26	3	2	12	14135	Histone H2A type 1-B/E
	P20671|H2A1D_HUMAN	61.28	26	3	2	12	14107	Histone H2A type 1-D
112	Q93079|H2B1H_HUMAN	81.16	29	5	3	18	13892	Histone H2B type 1-H
	Q99880|H2B1L_HUMAN	81.16	29	5	3	18	13952	Histone H2B type 1-L
	Q99879|H2B1M_HUMAN	81.16	29	5	3	18	13989	Histone H2B type 1-M
	Q99877|H2B1N_HUMAN	81.16	29	5	3	18	13922	Histone H2B type 1-N

PROTEIN GROUP	ACCESSION NUMBER	-10lgP	%	TOTAL PEPT.	U.	# PSM	AVG. MASS	DESCRIPTION
	P23527|H2B1O_HUMAN	81.16	29	5	3	18	13906	Histone H2B type 1-O
	Q16778|H2B2E_HUMAN	81.16	29	5	3	18	13920	Histone H2B type 2-E
	Q5QNW6|H2B2F_HUMAN	81.16	29	5	3	18	13920	Histone H2B type 2-F
	Q8N257|H2B3B_HUMAN	81.16	29	5	3	18	13908	Histone H2B type 3-B
114	P01834|IGKC_HUMAN	79.21	30	2	2	14	11765	Immunoglobulin kappa constant
	P0DOX7|IGK_HUMAN	79.21	15	2	2	14	23379	Immunoglobulin kappa light chain
119	P01011|AACT_HUMAN	77.32	11	5	3	17	47651	Alpha-1-antichymotrypsin
125	P0C0L4|CO4A_HUMAN	78.13	3	4	3	11	192784	Complement C4-A
	P0C0L5|CO4B_HUMAN	78.13	3	4	3	11	192750	Complement C4-B
133	P0DOX2|IGA2_HUMAN	59.84	4	2	2	12	48934	Immunoglobulin alpha-2 heavy chain
	P01876|IGHA1_HUMAN	59.84	5	2	2	12	37655	Immunoglobulin heavy constant alpha 1
	P01877|IGHA2_HUMAN	59.84	5	2	2	12	36591	Immunoglobulin heavy constant alpha 2
136	P69905|HBA_HUMAN	80.44	25	3	3	11	15258	Hemoglobin subunit alpha
141	P01859|IGHG2_HUMAN	58.81	7	2	1	10	35901	Immunoglobulin heavy constant gamma 2
151	P05164|PERM_HUMAN	63.21	3	2	2	8	83869	Myeloperoxidase
152	P00450|CERU_HUMAN	52.48	3	2	2	8	122205	Ceruloplasmin
161	P05155|IC1_HUMAN	67.63	9	3	3	5	55154	Plasma protease C1 inhibitor
164	P68871|HBB_HUMAN	60.8	18	2	2	7	15998	Hemoglobin subunit beta
	P02042|HBD_HUMAN	60.8	18	2	2	7	16055	Hemoglobin subunit delta
173	P02671|FIBA_HUMAN	58.47	5	3	3	6	94973	Fibrinogen alpha chain
175	P02679|FIBG_HUMAN	60.77	7	2	2	7	51512	Fibrinogen gamma chain
181	P0CG04|IGLC1_HUMAN	56.04	14	1	1	7	11348	Immunoglobulin lambda constant 1
	P0DOY2|IGLC2_HUMAN	56.04	14	1	1	7	11294	Immunoglobulin lambda constant 2
	P0DOY3|IGLC3_HUMAN	56.04	14	1	1	7	11266	Immunoglobulin lambda constant 3
	P0CF74|IGLC6_HUMAN	56.04	14	1	1	7	11277	Immunoglobulin lambda constant 6
	A0M8Q6|IGLC7_HUMAN	56.04	14	1	1	7	11254	Immunoglobulin lambda constant 7
	P0DOX8|IGL1_HUMAN	56.04	7	1	1	7	22830	Immunoglobulin lambda-1 light chain
	B9A064|IGLL5_HUMAN	56.04	7	1	1	7	23063	Immunoglobulin lambda-like polypeptide 5

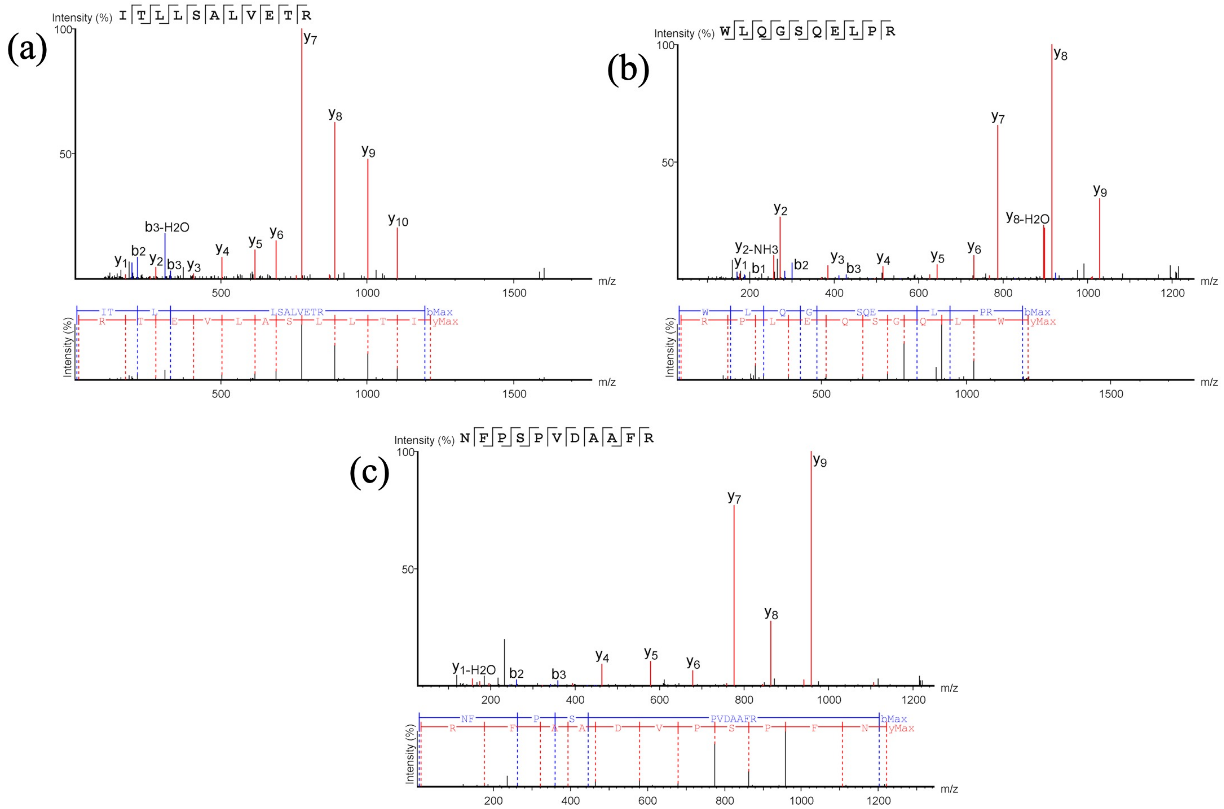

Figure S3.B15. Human peptide markers in P4d. a) Alpha-1-antichymotrypsin peptide K.I_{380}TLLSALVETR_{390}.T, score -l0lgP=40.2, *m/z*=608.3685, z=2, ppm=-0.7, # PSM=6**; b)** Immunoglobulin alpha-2 heavy chain peptide R.W_{366}LQGSQELPR_{375}.E, score -l0lgP=43.19, *m/z*=607.3215, z=2, ppm=2.8, # PSM=6**; c)** Hemopexin peptide K.N_{92}FPSPVDAAFR_{102}.Q, score -l0lgP=49.02, *m/z*=610.8085, z=2, ppm=3.3, # PSM=6

Table S3.B8. Fungi proteins identified in sample P4d: searched against *UniProtKB/Swiss-Prot reviewed* database, with a FDR of 1%, a protein score -10lgP ≥ 50, a minimum of two peptides and **one** unique peptide. -10lgP is Peaks score; TOTAL PEPT. is total number of peptides; U. is number of unique peptides in PEAKS; % is protein percentage coverage and # PSM is number of peptide-to-spectrum matches. **Aspergillus nidulans* is the heterotypic synonym of *E. nidulans*

PROTEIN GROUP	*ACCESSION NUMBER*	*-10lgP*	*%*	*TOTAL PEPT.*	*U.*	*# PSM*	*AVG. MASS*	*DESCRIPTION*
115	P23704|ATPB_NEUCR	87.6	9	4	1	18	55533	ATP synthase subunit beta mitochondrial OS=Neurospora crassa
134	Q8TFN0|NDK_EMENI	63.67	17	2	2	12	16908	Nucleoside diphosphate kinase OS=Emericella nidulans*
140	B8NLM9|ARTA_ASPFN<br>Q99002|1433_TRIHA	64.01	8	2	1	10	26869<br>29998	14-3-3 family protein artA OS=Aspergillus flavus<br>14-3-3 protein homolog OS=Trichoderma harzianum
150	Q8X1P0|CATA_BLUGH	61.42	3	2	2	8	79011	Catalase OS=Blumeria graminis f. sp. hordei
165	Q03134|FDH_EMENI	61.58	6	2	2	8	40131	Formate dehydrogenase OS=Emericella nidulans*

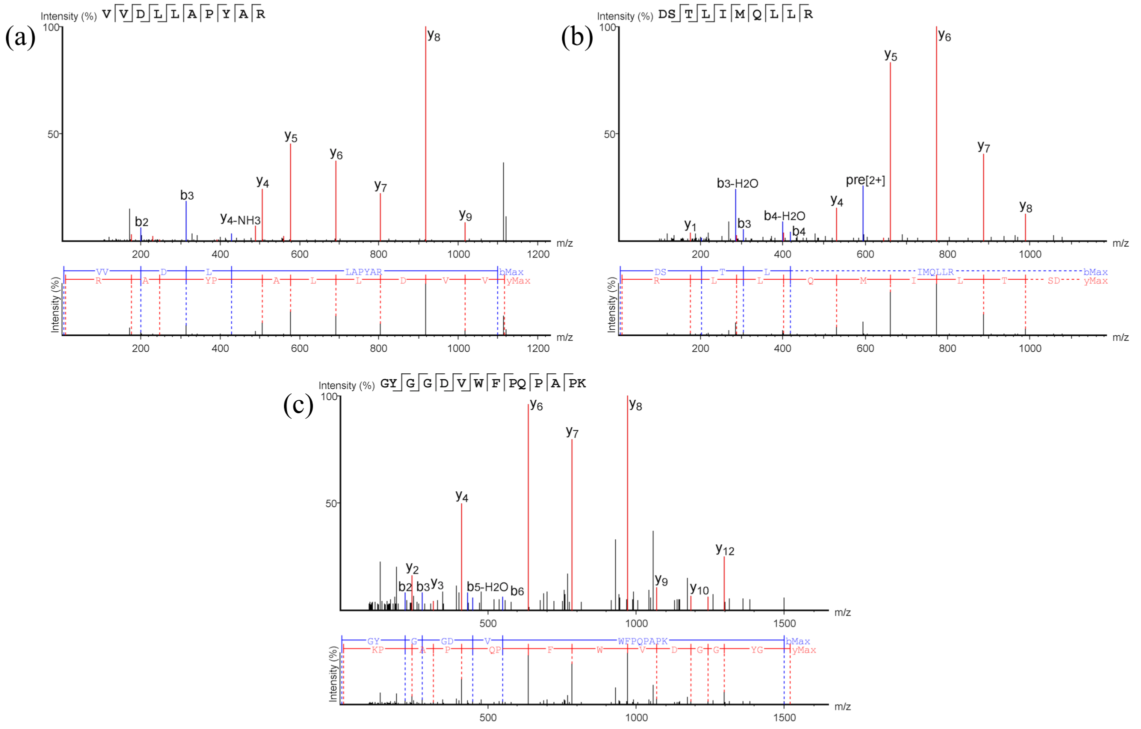

Figure S3.B16. Fungi peptide markers in P4d. a) ATP synthase subunit beta mitochondrial peptide K.V$_{178}$VDLLAPYAR$_{187}$.G, score -l0lgP=43.67, *m/z*=558.8238, z=2, ppm=-0.7, # PSM=6**; b)** 14-3-3 protein peptide K.D$_{215}$STLIMQLLR$_{224}$.D, score -l0lgP=46.19, *m/z*=595.3329, z=2, ppm=-0.4, # PSM=6**; c)** Formate dehydrogenase peptide R.G$_{279}$YGGDVWFPQPAPK$_{292}$.E, score -l0lgP=52.40, *m/z*=759.8722, z=2, ppm=-0.3, # PSM=6

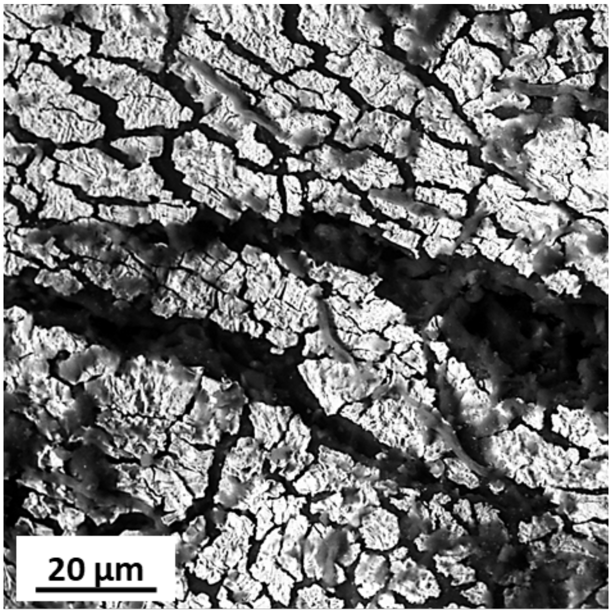

Figure S3.B17. Possible biological contamination on the surface of sample P4d. SEM-BSE image of the sample surface (scale bar 20 µm). Micrograph by Thomas Lam © Museum Conservation Institute, Smithsonian Institution

Identification of Equus sp. collagen markers

Equus sp. markers were identified in three samples: D12b (back), 1862:16 III and 1862:16 IV. Among the *Equus* species for which collagen proteins are available at the time of writing are *E. quagga* (zebra), *E. asinus* (domesticated donkey) and *E. caballus* and *E. przewalskii* (domesticated and wild horse respectively). In our region of interest, either donkey or horse is the expected source of equine collagen. The three samples with equine collagen are in textiles with an attribution to the Middle East and Central Asia/China. Besides domesticated horse and donkey, the wild Przewalski's horse is a potential source, especially for samples 1862:16, while a wild type of donkey *E. hemionus* or Asiatic wild ass is known in Asia. Markers that can differentiate domesticated donkey from horse have been described[32] and can be found in COL1A1 (one peptide) and in COL1A2 (four peptides). Only one marker was found in all of our samples, it corresponds to the sequence GA**S**GPAGVR/GA**T**GPAGVR in position 422-430 of COL1A2. GA**S**GPAGVR is known in COL1A2 of *E. caballus*, while GA**T**GPAGVR is found in *E. asinus* and *E. quagga*. While GA**S**GPAGVR is not found in *Bos* sp., *Ovis* sp. or *Capra* sp., GA**T**GPAGVR is found in all three. Both peptides were identified in all three samples, indicating the presence of at least domesticated horse collagen. The identification of GA**T**GPAGVR likely comes from sheep as the peptide was frequently found in the sheep leather samples, rather than donkey. **Figure S3.B18** shows peptide GASGPAGVR identified in sample 1862:16 III. While it can be confirmed that domesticated horse collagen is present in the samples, the presence or not of donkey (wild or domesticated) cannot be excluded based on the available sequences.

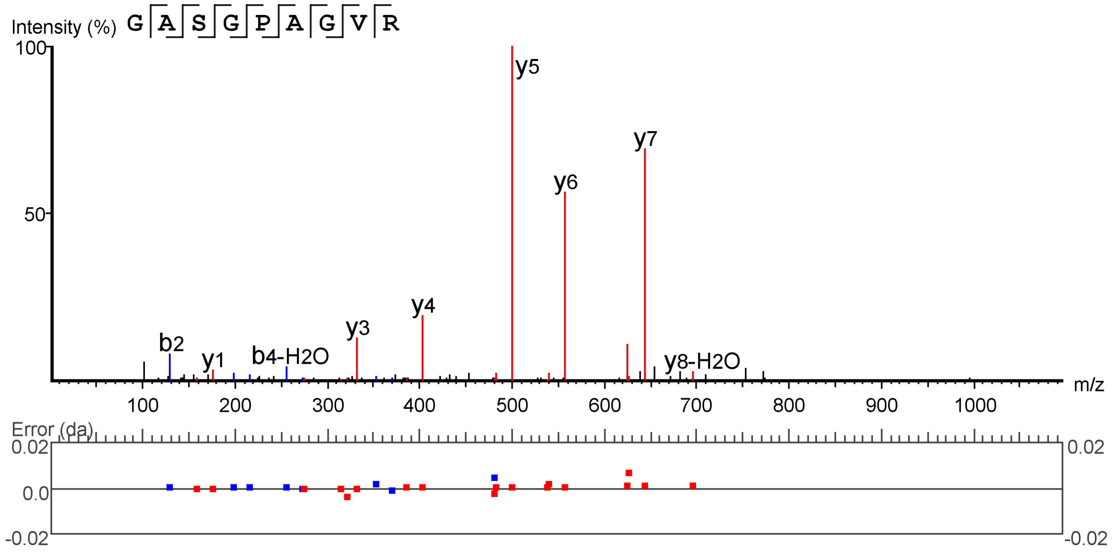

Figure S3.B18. Horse collagen marker in sample 1862:16 III. COL1A2 peptide R.G$_{422}$ASGPAGVR$_{430}$.G, score -l0lgP=31.21, *m/z*=386.2085, z=2, ppm=-1.3, # PSM=3

Contamination due to conservation purposes?

The collection of the Cooper-Hewitt, Smithsonian Design Museum has been actively used for educational purposes. The textiles along with other objects in the museum's collection, were studied, researched, and even copied in order to learn techniques and ultimately give students the necessary skills for employment in decorative arts professions (see Photograph, Students Studying Textiles, Cooper Union Museum for the Arts of Decoration | Objects | Collection of Cooper Hewitt, Smithsonian Design Museum). A common practice from the early 20th c. was to glue the textiles to cardboards to be studied. Macro-observation of object 1902-1-262 showed some white deposits (**Figure S3.B19a**) on the metal threads that could be consistent with glue. On-site analysis by FTIR-ATR of the white deposits indicates possible protein glue mixed with gypsum (**Figure S3.B19b**).

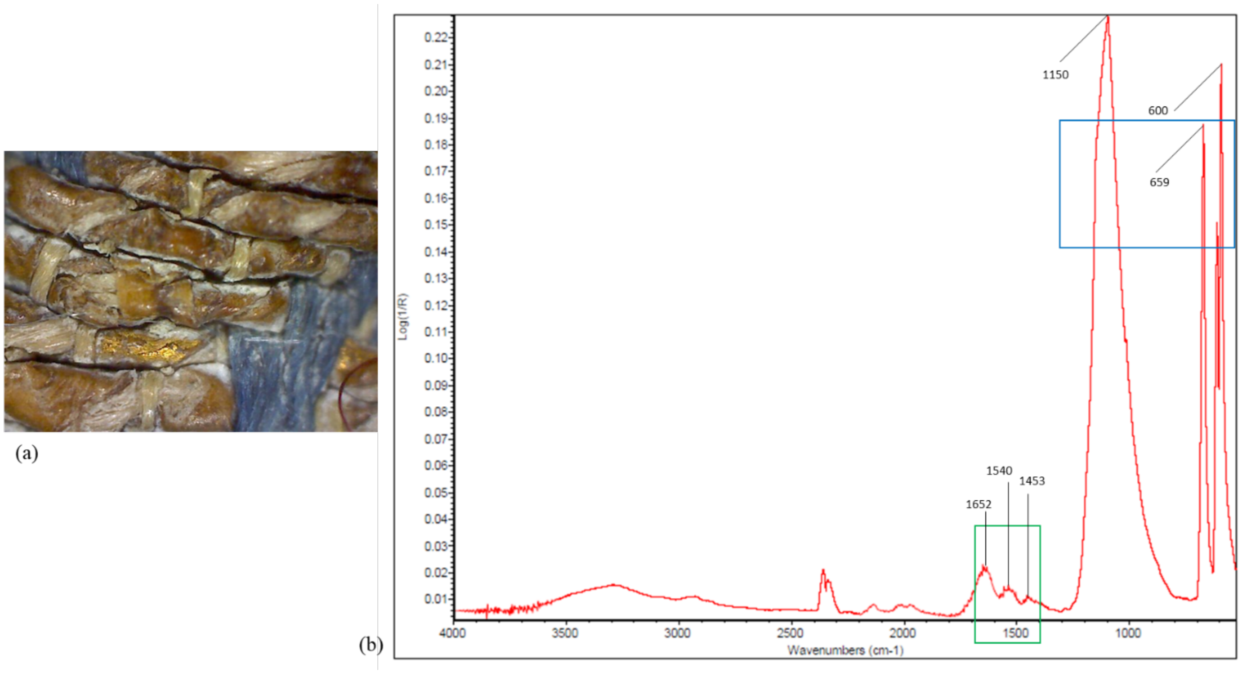

Figure S3.B19. a) White deposits on the object 1902-1-262. Detail of the obverse of the weave. Photo by Cristina Scibe © Museum Conservation Institute, Smithsonian Institution. **b) FTIR-ATR spectrum of the white deposits.** The absorption region of the calcium sulphate is marked by the blue square, while the absorption region of proteins is indicated by the green square. The FTIR-ATR spectrum was kindly provided by Kira Eng-Wilmot and Geena Roth and reveals the nature of the white deposits widespread on the obverse of the object. The peaks at 1453 cm^{-1}, 1540 cm^{-1} and 1652 cm^{-1} are typical of a proteinaceous material, while the peaks at 600 cm^{-1}, 659 cm^{-1} and 1150 cm^{-1} are typical of calcium sulphate (hydrated).

3.C Calculation of deamidation

Deamidation was calculated for the three main chains of collagen in percentage of asparagine N and glutamine Q with deamidation to the total number of N and Q residues in all peptide-spectrum matches identified in the three proteins.

The results of deamidation on the three main chains of collagen for the membrane threads are shown **Figure S3.C1**, indicating as expected much higher deamidation for asparagine than glutamine, consistent with studies of ancient collagen [33,34]. In yellow are the oldest samples and in dark brown the youngest samples. For asparagine, the percentage ranges from 39% in **1902-1-279_1** to 56% in **P12**, compared to 35% on average for the cow membrane references. There is no clear correlation between the age of the samples and their increase in deamidation. For glutamine, the percentage ranges from 0.3% in **P9** to 17% in **1902-1-227_1**, compared to 0.4% on average for the cow membrane references. Here again there is no clear correlation between the age of the samples and their increase in deamidation, although some of the oldest samples have the highest increase in deamidation and some of the youngest have the lowest increase. More than age, the context in which the textile was kept, the area sampled, the exposure (front or back of textile) and metal covering, as well as perhaps the thickness of the membrane, must have an influence of the deamidation increase, as illustrated by samples from the same textile but taken in different areas (e.g. **1902-1-279_1** and **1902-1-279_2**).

a)

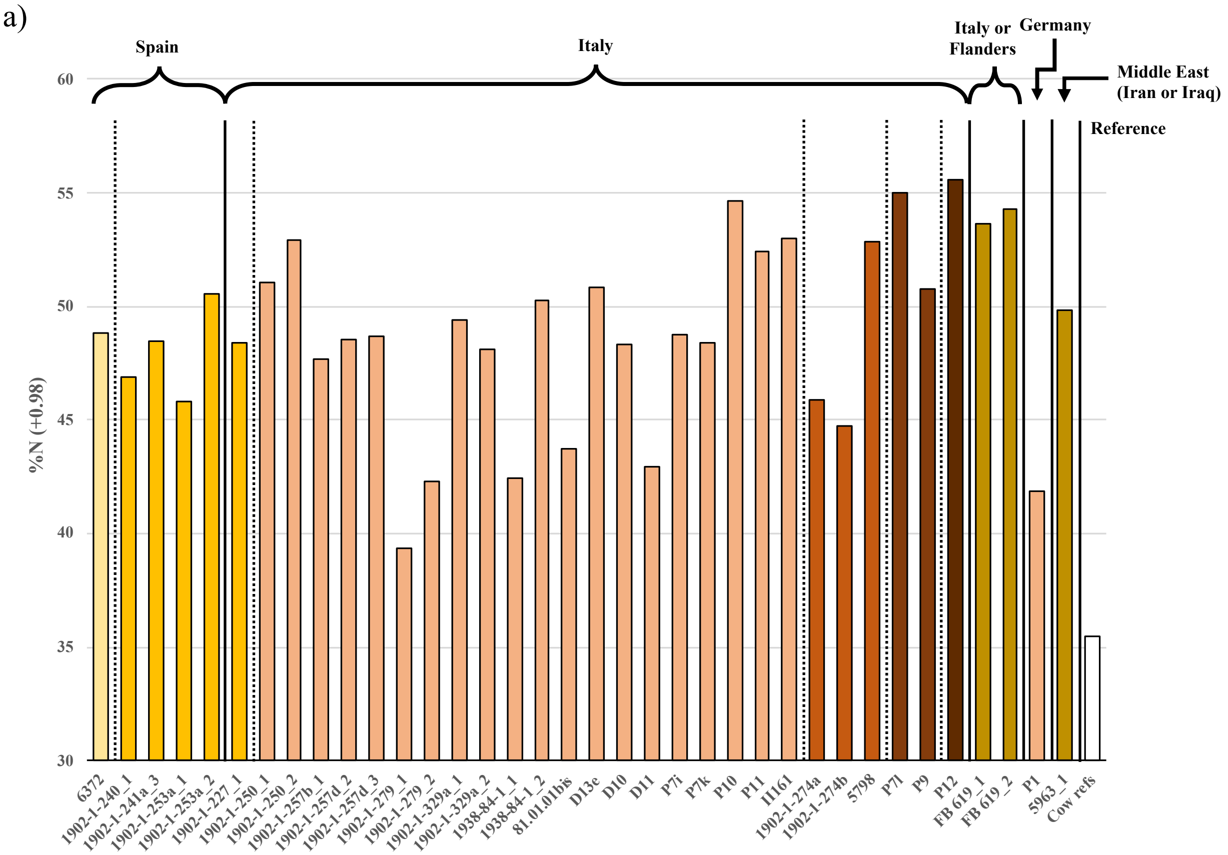

b)

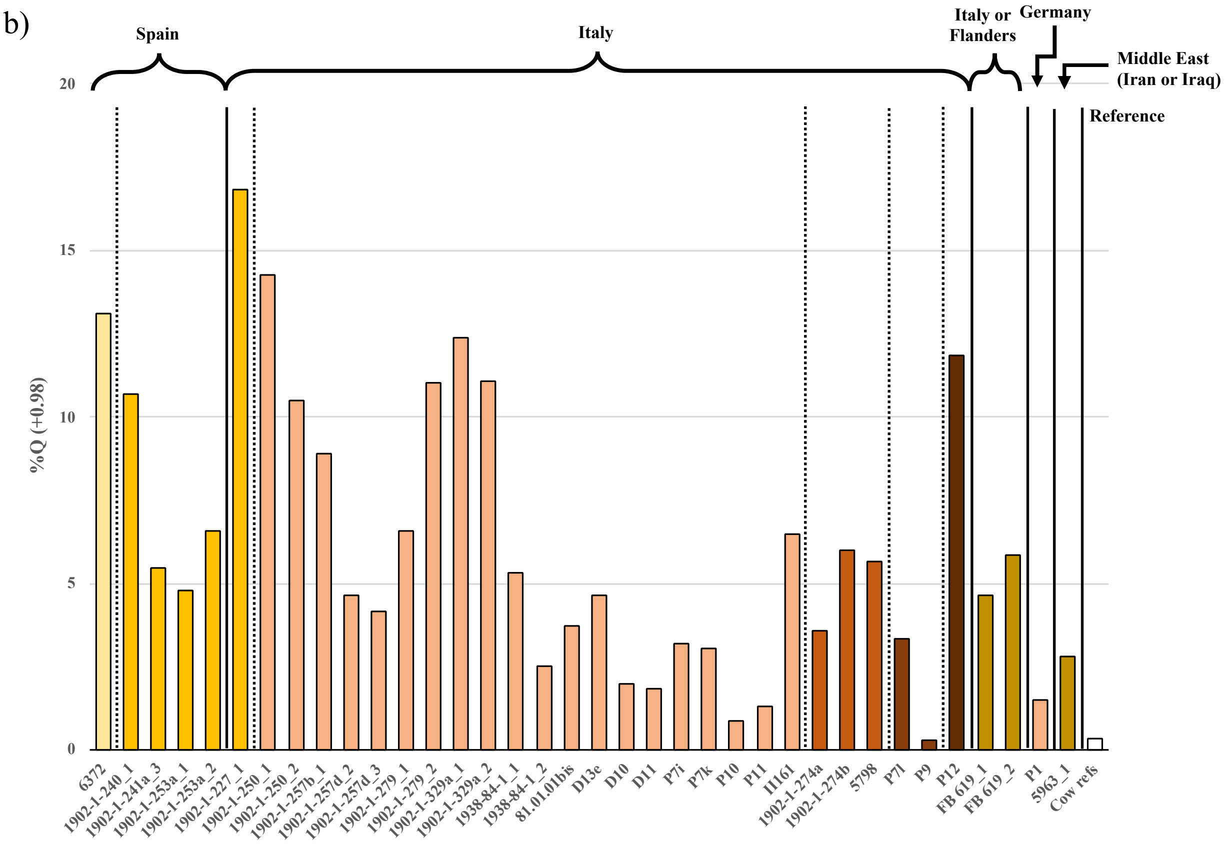

Figure S3.C1. a) Deamidation of asparagine N in membrane threads. b) Deamidation of glutamine Q in membrane threads. Percentage of N and Q with deamidation calculated in all samples for the three main chains of *Bos* collagen, based on their peptide-spectrum matches. Color key for textile samples as follow: **12-13th century; 13th century; 13-14th century; 14th century; 14-15th century; 15th century; 16th century.** With NCBI collagen vertebrates, FDR 1%, protein score -10lgP ≥ 50, and zero unique peptides.

As with the membrane threads, deamidation was calculated for the skin threads for the three main chains of collagen. The values, calculated only on the collagen of the identified skin species, are shown **Figure S3.C2**. In yellow are the oldest samples and in dark brown the youngest samples. For asparagine, the percentage ranges from 48% in **D12b** (sheep skin) to 96% in **5776_1** (goat skin). For reference, a vegetable-tanned leather (cattle) has a 67% deamidation level. For glutamine, the percentage ranges from 23% in **03.02.02_3** (sheep skin) to 81% in **5776_1** (goat skin), compared to 39% for the leather reference. There is no clear correlation between the age of the samples and their increase in deamidation. Instead, we notice that deamidation tends to be higher for the goat-skin samples rather than the sheep-skin samples: **Figure S3.C3** showing %N(+0.98) plotted against %Q(+0.98). For the samples that contain an

adhesive made of collagen glue from another mammalian species, the actual values might be skewed due to deamidation from homologous peptides. However the adhesive layers are usually thin in comparison to the skin layers, and the contribution from the adhesive can be considered negligible. **Figure S3.C3** also shows that the archaeological skins have deamidation levels in the range of vegetable-tanned skin or parchment rather than raw hide, vellum or alum-tawed and oil tanned leather. For reference, deamidation values for the adhesives are indicated **Figure S3.C3** and were evaluated by calculating deamidation percentage in ovalbumin for the egg white (for samples with percentage coverage of ovalbumin > 20%), horse collagens in the suspected horse glue and sturgeon collagens (only COL1A1 and COL1A2) for the fish glue. For egg white, percentage of deamidation of asparagine and glutamine were around 40%, with asparagine only slightly more deamidated than glutamine. Similar levels of deamidation for egg white were also observed in painting binders, compared to glue made from collagen where asparagine deamidation is much higher [35]. In horse glue, indeed, and although it was based on only two samples, the deamidation levels are quite high, especially for asparagine. Deamidation from possible collagen glue from other mammals was not calculated due to the similarity between the collagens of *Ovis aries*, *Capra hircus* and *Bos taurus*, and the impossibility of determining how much collagen comes from the skins or the adhesives. Deamidation was however calculated from the sturgeon glue and shows much lower levels than the mammalian skins and glue. In the study of commercial glues for restoration, fish glue was generally found to be less deamidated than bone and hide glues [36].

a)

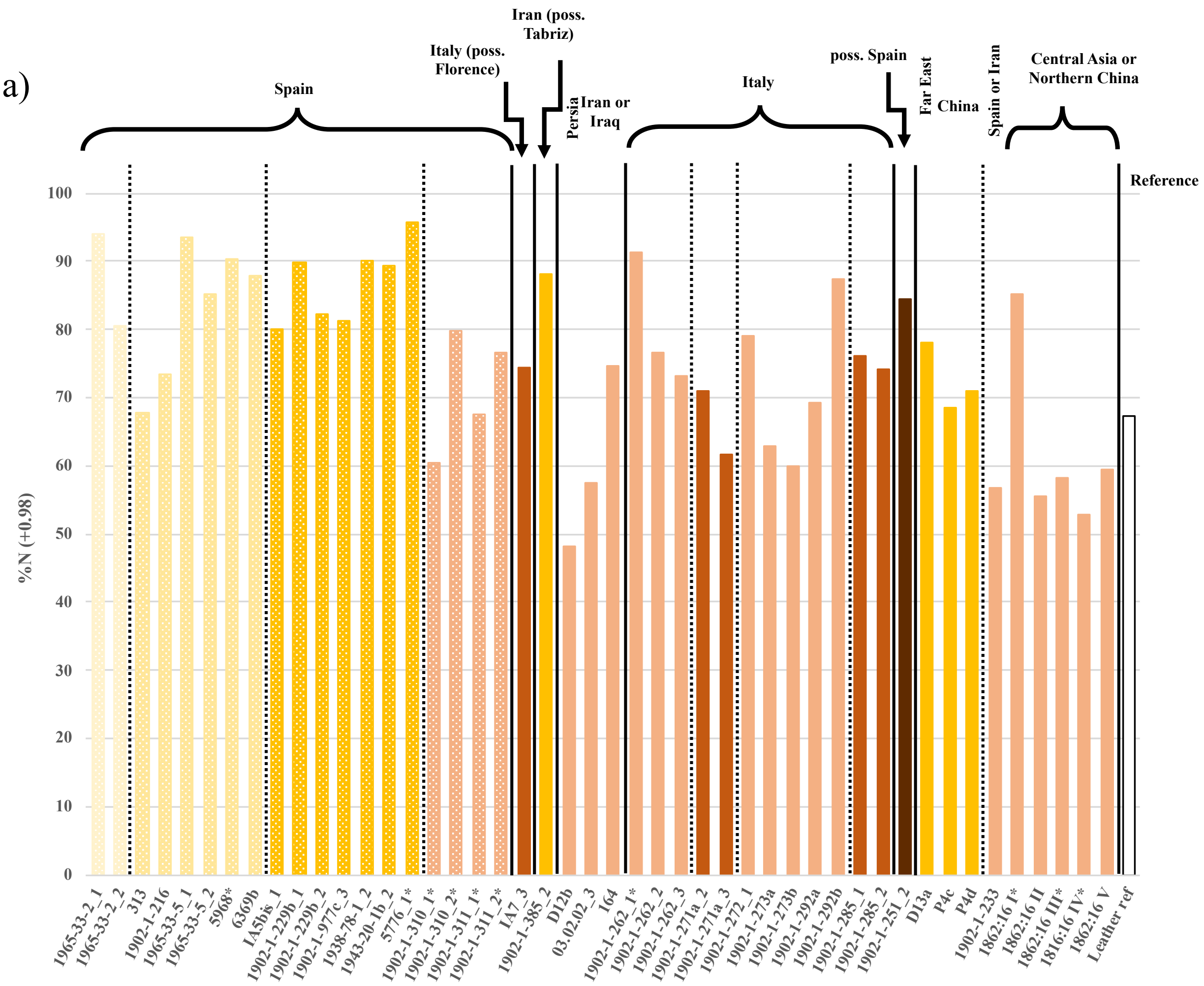
Iran (poss. Tabriz)
Italy (poss. Florence)
Spain
Persia
Iran or Iraq
Italy
poss. Spain
Far East
China
Spain or Iran
Central Asia or Northern China
Reference
%N (+0.98)
100
90
80
70
60
50
40
30
20
10
0
1965-33-2_1
1965-33-2_2
313
1902-1-216
1965-33-5_1
1965-33-5_2
5968*
6369b
IA5bis_1
1902-1-229b_1
1902-1-229b_2
1902-1-977c_3
1938-78-1_2
1943-20-1b_2
5776_1*
1902-1-310_1*
1902-1-310_2*
1902-1-311_1*
1902-1-311_2*
IA7_3
1902-1-385_2
D12b
03.02.02_3
164
1902-1-262_1*
1902-1-262_2
1902-1-262_3
1902-1-271a_2
1902-1-271a_3
1902-1-272_1
1902-1-273a
1902-1-273b
1902-1-292a
1902-1-292b
1902-1-285_1
1902-1-285_2
1902-1-251_2
D13a
P4c
P4d
1902-1-233
1862:16 I*
1862:16 II
1862:16 III*
1816:16 IV*
1862:16 V
Leather ref

b)

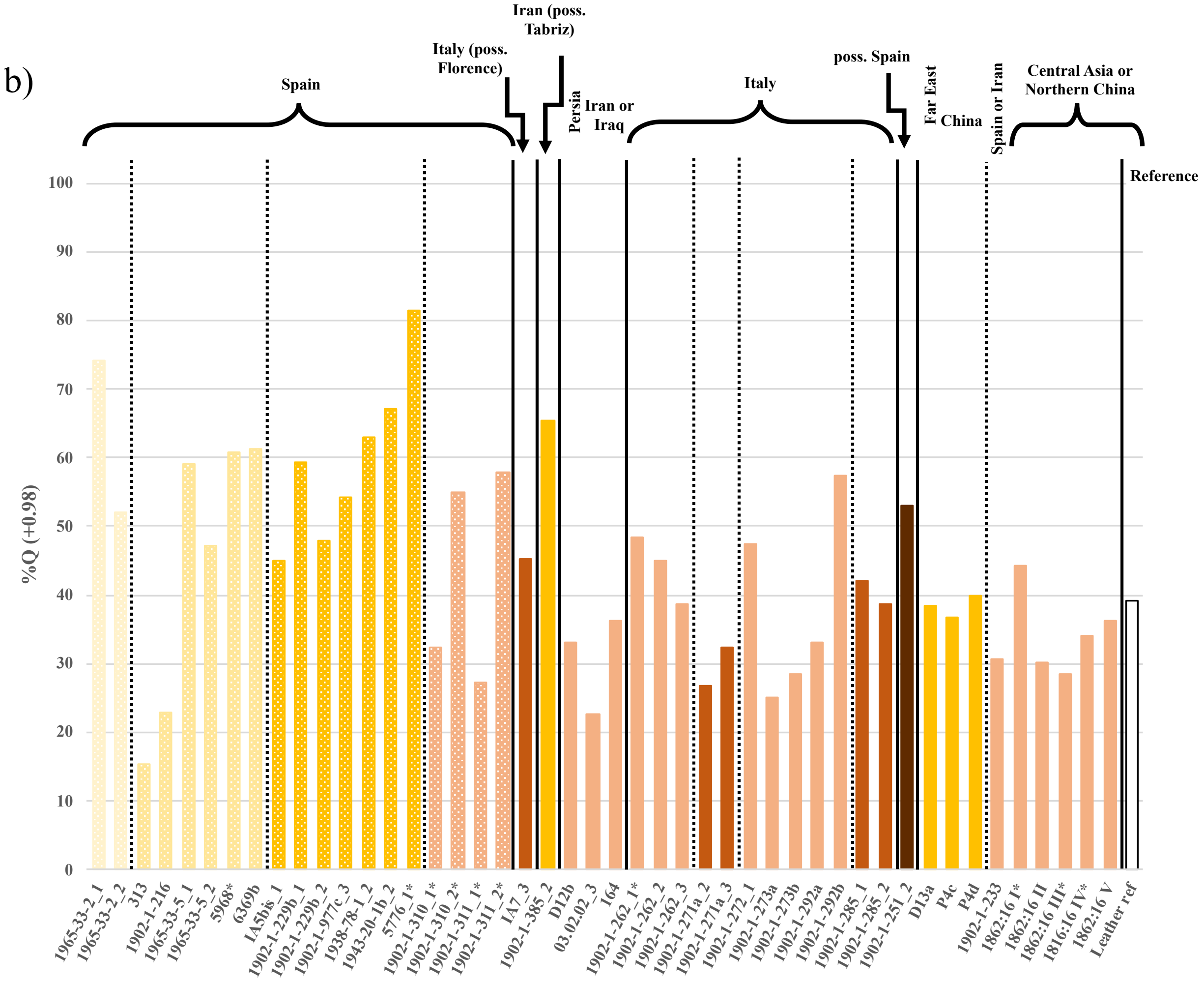

Figure S3.C2. a) Deamidation of asparagine N in skin threads. b) Deamidation of glutamine Q in skin threads. Percentage of N and Q with deamidation calculated in all samples for the three main chains of collagen of the identified skin species, based on their peptide-spectrum matches. Color key for textile samples as follow: **11-12th century; 12-13th century**; **13th century**; **14th century**; **14-15th century**; **15th century.** Full colored background represents the sheep skin substrates, white dots on colored background represents goat skin substrates. With NCBI collagen vertebrates, FDR 1%, protein score -10lgP ≥ 50, and zero unique peptides. * indicates samples where more than one mammalian collagen species was found.

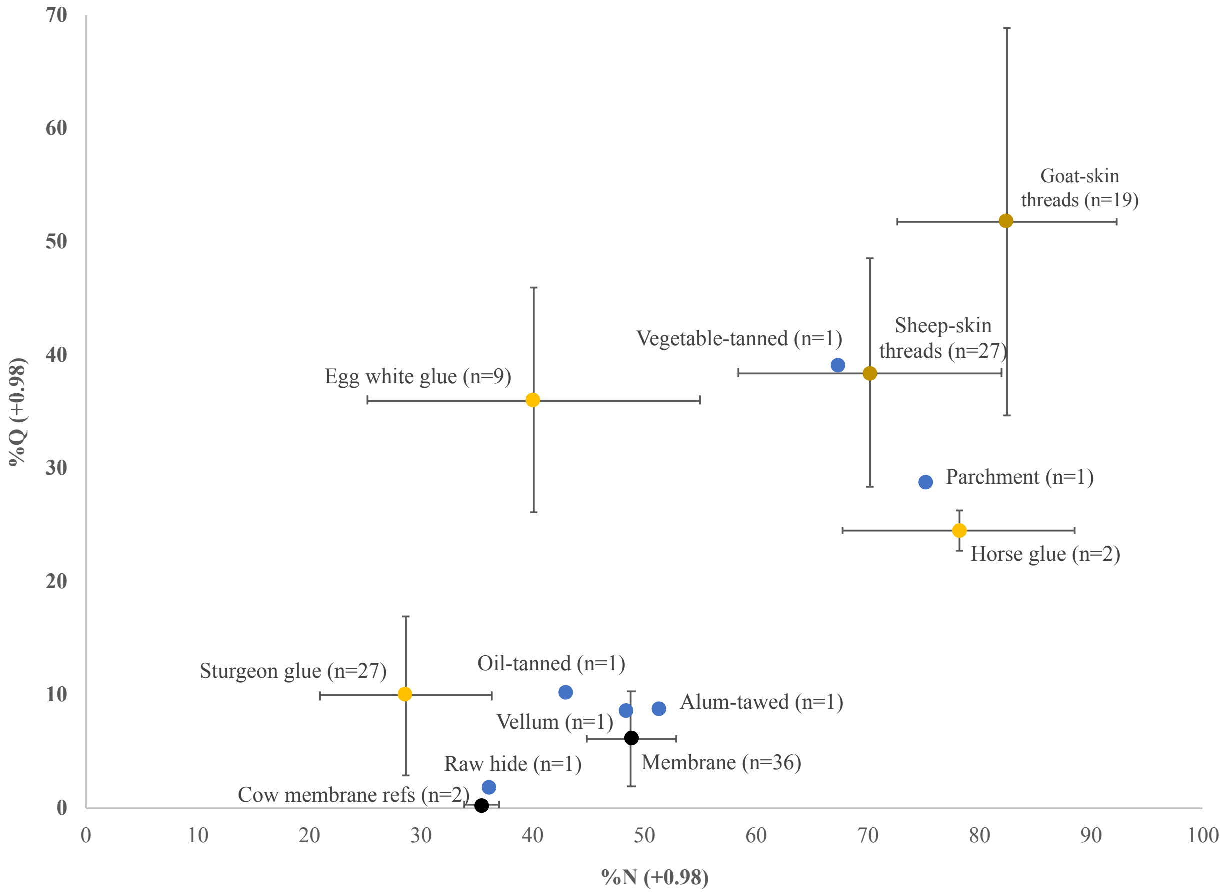

Figure S3.C3. Percentage of N and Q with deamidation by sample categories. With NCBI collagen vertebrates, FDR 1%, protein score -10lgP ≥ 50, and zero unique peptides. Average values with standard deviations are shown for the archaeological skin threads in brown, the archaeological adhesives in yellow, the reference skin samples in bleu (one sample tested for each type), and for comparison the average of the archaeological membrane threads in black. Deamidation for egg white glue was calculated for samples with percentage coverage of ovalbumin > 20%.

BIBLIOGRAPHY

1 Popowich, A. K., Cleland, T. P. & Solazzo, C. Characterization of membrane metal threads by proteomics and analysis of a 14th c. thread from an Italian textile. *J. Cult. Herit.* **33**, 10-17 (2018).

2 Krist, G., Kimmel, T., Pichler, B. & Delvai, E. *Medieval fabric fragments from the collection of the Universalmuseum Joanneum in Graz, Austria. Technological analysis and conservation*, University of Applied Arts, Vienna, (2017).

3 Bridge, T. W. The Natural History of Isinglass. *J. I. Brewing* **11**, 508-531 (1905).

4 Du, K. *et al.* The sterlet sturgeon genome sequence and the mechanisms of segmental rediploidization. *Nature Ecology & Evolution* **4**, 841-852 (2020).

5 Denecker, G., Ovaere, P., Vandenabeele, P. & Declercq, W. Caspase-14 reveals its secrets. *The Journal of cell biology* **180**, 451-458 (2008).

6 Uhlén, M. *et al.* Tissue-based map of the human proteome. *Science* **347**, 1260419 (2015).

7 Méhul, B., Bernard, D., Simonetti, L., Bernard, M. A. & Schmidt, R. Identification and cloning of a new calmodulin-like protein from human epidermis. *J Biol Chem* **275**, 12841-12847 (2000).

8 Eckert, R. L. *et al.* S100 proteins in the epidermis. *J Invest Dermatol* **123**, 23-33 (2004).

9 Wang, S. *et al.* S100A8/A9 in Inflammation. *Frontiers in Immunology* **9** (2018).

10 Thorey, I. S. *et al.* The Ca2+-binding Proteins S100A8 and S100A9 Are Encoded by Novel Injury-regulated Genes. *Journal of Biological Chemistry* **276**, 35818-35825 (2001).

11 Lee, K. C. & Eckert, R. L. S100A7 (Psoriasin)--mechanism of antibacterial action in wounds. *J Invest Dermatol* **127**, 945-957 (2007).

12 Broome, A.-M., Ryan, D. & Eckert, R. L. S100 Protein Subcellular Localization During Epidermal Differentiation and Psoriasis. *Journal of Histochemistry & Cytochemistry* **51**, 675-685 (2003).

13 Markiewicz, A., Sigorski, D., Markiewicz, M., Owczarczyk-Saczonek, A. & Placek, W. Caspase-14-From Biomolecular Basics to Clinical Approach. A Review of Available Data. *Int J Mol Sci* **22** (2021).

14 Masinter, M. F. *Sarah & Eleanor: The Hewitt Sisters. Founders of the Nation's Design Museum*. (Cooper Hewitt, Smithsonian Design Museum, NYC, 2016).

15 Pires, E., Carvalho, L. d. C., Shimada, I. & McCullagh, J. Human Blood and Bird Egg Proteins Identified in Red Paint Covering a 1000-Year-Old Gold Mask from Peru. *Journal of Proteome Research* **20**, 5212-5217 (2021).

16 Wiktorowicz, C. J., Arnold, B., Wiktorowicz, J. E., Murray, M. L. & Kurosky, A. Hemorrhagic fever virus, human blood, and tissues in Iron Age mortuary vessels. *Journal of Archaeological Science* **78**, 29-39 (2017).

17 Granzotto, C., Sutherland, K., Goo, Y. A. & Aksamija, A. Characterization of surface materials on African sculptures: new insights from a multi-analytical study including proteomics. *Analyst* **146**, 3305-3316 (2021).

18 D'Amato, A. *et al.* Anton Chekhov and Robert Koch Cheek to Cheek: A Proteomic Study. *Proteomics* **18**, e1700447; 10.1002/pmic.201700447 (2018).

19 Jain, S., Gautam, V. & Naseem, S. Acute-phase proteins: As diagnostic tool. *J Pharm Bioallied Sci* **3**, 118-127 (2011).

20 Gruys, E., Toussaint, M. J. M., Niewold, T. A. & Koopmans, S. J. Acute phase reaction and acute phase proteins. *J Zhejiang Univ Sci B* **6**, 1045-1056 (2005).
21 Perkins, D. D. Neurospora: the organism behind the molecular revolution. *Genetics* **130**, 687-701 (1992).
22 Perkins, D. D. & Davis, R. H. Evidence for safety of Neurospora species for academic and commercial uses. *Appl Environ Microbiol* **66**, 5107-5109 (2000).
23 Cowger, C. & Brown, J. K. M. Blumeria graminis (powdery mildew of grasses and cereals). *Invasive Species Compendium* DOI:10.1079/ISC.22075.20210198939 (2019).
24 Noir, S., Colby, T., Harzen, A., Schmidt, J. & Panstruga, R. A proteomic analysis of powdery mildew (Blumeria graminis f.sp. hordei) conidiospores. *Molecular Plant Pathology* **10**, 223-236 (2009).
25 Bennett, J. W. An Overview of the Genus Aspergillus in *The Aspergilli* 23-34 (CRC Press, 2016).
26 Kosmidis, C. *Aspergillosis*, <https://rarediseases.org/rare-diseases/aspergillosis/> (2018).
27 Dhakal A & E., S. Aflatoxin Toxicity. [Updated 2021 May 24], Pages (Treasure Island (FL): StatPearls Publishing, 2021).
28 Tillie-Leblond, I. & Tonnel, A. B. Allergic bronchopulmonary aspergillosis. *Allergy* **60**, 1004-1013 (2005).
29 Henriet, S. S. V., Verweij, P. E. & Warris, A. Aspergillus nidulans and Chronic Granulomatous Disease: A Unique Host–Pathogen Interaction. *The Journal of Infectious Diseases* **206**, 1128-1137 (2012).
30 Krel, M. *et al.* Host biomarkers of invasive pulmonary aspergillosis to monitor therapeutic response. *Antimicrob Agents Chemother* **58**, 3373-3378 (2014).
31 Polacheck, I. *et al.* Damage to an ancient parchment document by Aspergillus. *Mycopathologia* **106**, 89-93 (2004).
32 Paladugu, R. *et al.* Your horse is a donkey! Identifying domesticated equids from Western Iberia using collagen fingerprinting. *Journal of Archaeological Science* **149**, 105696 (2023).
33 Wilson, J., van Doorn, N. L. & Collins, M. J. Assessing the Extent of Bone Degradation Using Glutamine Deamidation in Collagen. *Analytical Chemistry* **84**, 9041-9048 (2012).
34 Ramsøe, A. *et al.* DeamiDATE 1.0: Site-specific deamidation as a tool to assess authenticity of members of ancient proteomes. *Journal of Archaeological Science* **115**, 105080 (2020).
35 Mackie, M. *et al.* Palaeoproteomic Profiling of Conservation Layers on a 14th Century Italian Wall Painting. *Angewandte Chemie International Edition* **57**, 7369-7374 (2018).
36 Ntasi, G. *et al.* Proteomic Characterization of Collagen-Based Animal Glues for Restoration. *J. Proteome Res.* **21**, 2173-2184 (2022).
