# Supplementary material for: Palaeoproteomics and microanalysis reveal techniques of production of animal-based metal threads in medieval textiles
Source: Sci Rep. 2024 Mar 4;14:5320. doi: 10.1038/s41598-024-54480-4 (PMC10912450; doi:10.1038/s41598-024-54480-4)
Supplement: Supplementary file 4 — Supplementary Information 4. [file 41598_2024_54480_MOESM4_ESM.pdf]

SI-4_SEM-EDS and SEM-microXRF

Palaeoproteomics and microanalysis reveal techniques of production of animal-based metal threads in medieval textiles

This PDF file includes:

Supplementary Text, Figures and Tables

Contents:

4.A SEM-EDS morphological characterization: strip substrate

4.B SEM-EDS and SEM-µXRF analysis: Membrane-based metal threads

4.C SEM-EDS and SEM-µXRF analysis: Skin-based metal threads

List of figures:

4.A

Figure S4.A1. Strip morphological characterization. (a-b) Skin substrate. (c-d) Membrane substrate.

4.B

Figure S4.B1. (a-d) Metal coating morphology on membrane-based strips.

Figure S4.B2. Scheme of gilt-silver coating layering on membrane strips.

Figure S4.B3. Mercury detection by SEM-µXRF on gilt-silver leaf coatings.

Figure S4.B4. (a-b) EDS surface elemental mapping of the membrane strip.

4.C

Figure S4.C1. (a-c) Metal coating morphology on skin-based strips.

Figure S4.C2. (a-d) Burnishing marks and “craquelure” of the metal leaves surface on skin-based strips.

Figure S4.C3. Coatings compositional groups in skin-based strips: EDS surface elemental mapping.

Figure S4.C4a. Correlation between the metal coating composition of the samples and the corresponding objects provenance.

Figure S4.C4b. Correlation between the metal coating composition of the samples and the corresponding objects date.

Figure S4.C5. EDS elemental mapping of the skin strip: cross-section VS surface analysis.

List of Tables:

4.B

Table S4.B1. Elemental characterization of metal coatings in membrane-based threads.

4.C

Table S4.C1. Elemental characterization of metal coatings in skin-based threads (wrapped and flat strips).

Table S4.C2. Coatings compositional groups and subgroups in skin-based threads.

Table S4.C3. Gold-coatings (group II). Average concentration values for gold (Au) and silver (Ag) calculated for each provenance group of the objects sampled.

4.A SEM-EDS morphological characterization: strip substrate

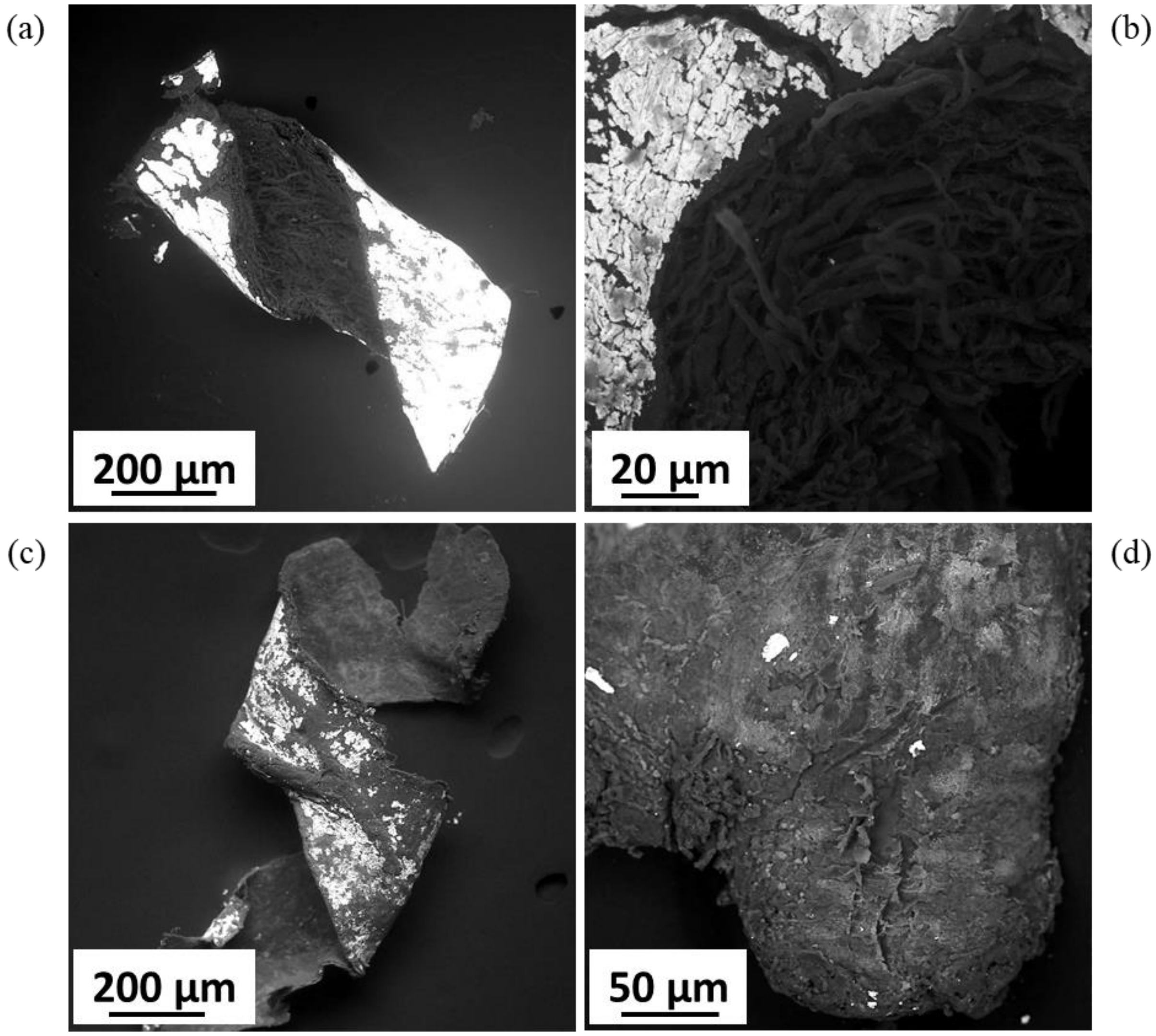

Figure S4.A1. Strips morphological characterization. Skin substrate: a) SEM-BSE image, sample **1943-20-1b_2** (scale bar 200µm); b) SEM-BSE image, fibrous structure typical of leather, sample **1902-1-251_1** (scale bar 20µm). **Membrane substrate:** c) SEM-BSE image, sample **1902-1-241a_2** (scale bar 200µm); d) SEM-BSE image, inner uncoated surface of the strip, sample **1902-1-257d_2** (scale bar 50µm). Images by Thomas Lam © Museum Conservation Institute, Smithsonian Institution

4.B SEM-EDS and SEM-µXRF analysis: Membrane-based metal threads

The different morphology of the bi-layered gilt silver leaves is possibly related to the size and thickness of the leaves used, which sometimes were applied one on top of the other to get a thicker gilding of a base metal [1]. Moreover, the leaves showed a geometry alteration induced by the presence of metal corrosion products and the natural shrinkage of the underneath membranous support.

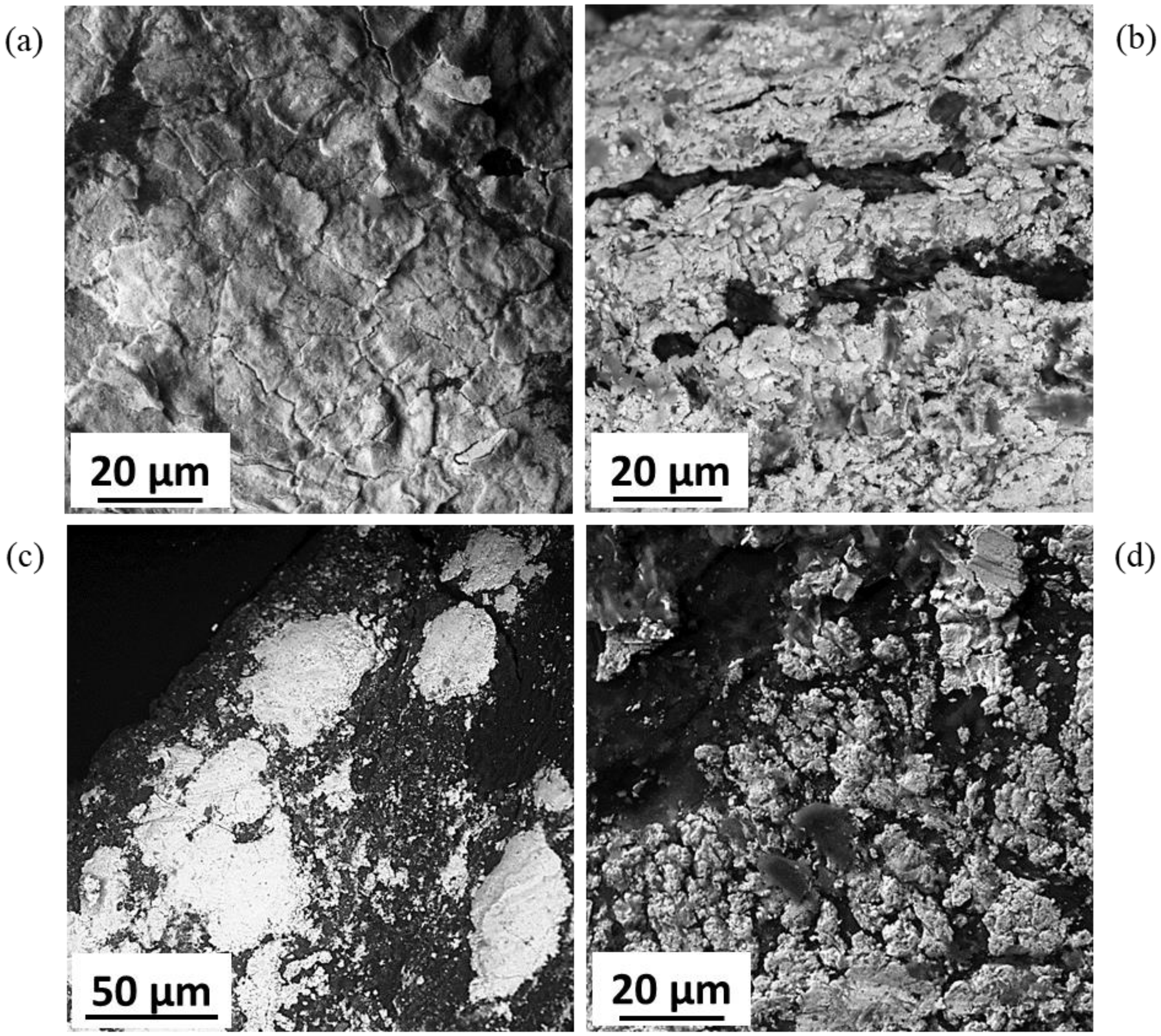

Figure S4.B1. Metal coating morphology on membrane-based strips: a) SEM-BSE image, leaf-made coating, sample **P12** (scale bar 20µm); b) SEM-BSE image, leaf-made coating, abundant presence of silver sulphides (sulphur concentrations of 11.75-12.84 wt%, see **Table S4.B1**), sample **1902-1-329a_1** (scale bar 20µm); c) SEM-BSE image, worn and delaminated coating, possibly leaf-made, sample **1902-1-227_2** (scale bar 50µm); d) SEM-BSE image, worn and delaminated coating, possibly leaf-made, sample **P9** (scale bar 20µm). Images by Thomas Lam © Museum Conservation Institute, Smithsonian Institution

Table S4.B1. Elemental characterization of metal coatings in membrane-based threads. Detail by sample of the SEM-EDS/µ-XRF qualitative analysis of the elements detected as associated to the metal composition. The SEM-EDS semi-quantitative analysis of the main elements, gold (Au) and silver (Ag), and the silver/gold weight ratios are reported. Sulphur (S) and chlorine (Cl) were quantified as well as associated to silver degradation products, the elemental concentration of chlorine was reported when above 1wt%. Elemental concentrations are expressed in atomic weight percentage (wt%). For the first 11 samples, according to EDS surface elemental maps, average concentrations were acquired separately in areas showing higher distribution of gold (gl) or silver (sl). A multi-voltage EDS analysis was attempted at 5kV and 15kV for sample 3RU8457_1, which showed the most well-preserved metal coating. Finally, for the other samples, overall average concentrations were acquired. For each sample, the description of the metal coating identified is reported. (*) µ-XRF data; gl = gold layer; sl = silver layer.

Sample	Main elements			Degradation products		Minor and trace elements	Metal coating
	Au	Ag	Ag/Au	S	Cl		
1902-1-240_2 gl	7,08	84,43	11,93	8,49	<1	(*) Cu, Fe, Ni	Gilt-silver leaf
1902-1-240_2 sl	5,25	84,91	16,17	9,85			
1902-1-241a_2 gl	14,28	73,15	5,12	12,57	<1	(*) Cu, Fe, Hg, Ni, Ti	Gilt-silver leaf
1902-1-241a_2 sl	3,74	84,79	22,60	11,47			
1902-1-253a_1 gl	42,71	50,63	1,19	6,66	<1	(*) Cu, Fe, Ti	Gilt-silver leaf
1902-1-253a_1 sl	14,28	73,92	5,18	11,8			
1902-1-227_2 sl	2,1	77,37	36,84	5,97	14,57	(*) Cu, Fe, Hg, Zn, Ni, Ti	Worn coating
1902-1-250_2 gl	13,4	82,38	6,15	1,13	3,09	(*) Cu, Fe, Ni	Gilt-silver leaf
1902-1-250_2 sl	3,91	88,66	22,67	3,53	3,9		
1938-84-1_1 sl		78,37	no Au	21,63			Almost completely worn coating
1902-1-257b_1 gl	14,49	72,43	5,00	13,08	<1	(*) Fe, Hg	Gilt-silver leaf
1902-1-257b_1 sl	3,2	83,77	26,18	13,03			
1902-1-257d_2 gl	22,14	69,89	3,16	7,97	<1	(*) Cu, Fe, Zn, Pb	Gilt-silver leaf
1902-1-257d_2 sl	4,68	85,25	18,21	8,83	1,25		
1902-1-329a_1 gl	17,62	70,63	4,01	11,75	<1	(*) Cu, Fe, Hg, Zn, Ni, Ti	Gilt-silver leaf
1902-1-329a_1 sl	5,13	82,03	15,99	12,84			
1902-1-329a_2 gl	32,63	54,97	1,68	7,77	4,64		Gilt-silver leaf
1902-1-329a_2 sl	4,68	81,96	17,51	12,78	<1		
P7i gl	21,44	68,25	3,18	5,24	5,07		Gilt-silver leaf
P7i sl	15,25	72,03	4,72	3,43	9,3		
3RU8457_1 gl (5kV)	44,72	52,94	1,18	2,34			Gilt-silver leaf
3RU8457_1 sl (15kV)	23,56	75,72	3,21	0,73			
3RU8457_2	17,98	79,68	4,43	2,34			Gilt-silver leaf
1902-1-274a	19,70	76,92	3,90	3,38	<1	Fe	Gilt-silver leaf
1902-1-274b	23,98	68,91	2,87	6,05	1,06		Gilt-silver leaf
1902-1-279_1	18,01	78,51	4,36	1,94	1,54		Gilt-silver leaf

Table S4.B1. (continued)

Sample	Main elements			Degradation products		Minor and trace elements	Metal coating
	Au	Ag	Ag/Au	S	Cl		
D13e	17,81	70,64	3,97	11,54			Gilt-silver leaf
D10	17,82	77,22	4,33	3,92	1,08		Gilt-silver leaf
D11	9,48	81,54	8,60	8,98			Gilt-silver leaf
P7k	11,89	74,56	6,27	7,92	5,63	Fe	Gilt-silver leaf
P7l	37,35	57,39	1,54	3,32	1,94	Cu, Fe	Gilt-silver leaf
P9	8,82	71,06	8,06	2,88	17,24	Fe	Gilt-silver leaf
P10		82,96	no Au	11,46	5,59		Worn coating
P11	16,19	74,26	4,58	8,00	1,56		Gilt-silver leaf
P12	11,70	77,23	6,60	11,07		Fe	Gilt-silver leaf
P1	22,09	68,11	3,08	15,05	5,90	Fe	Gilt-silver leaf

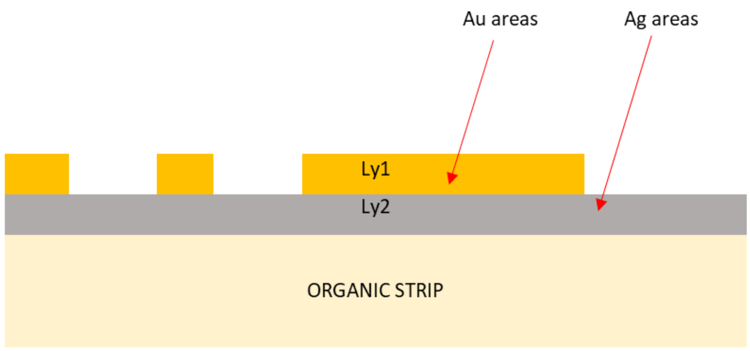

Figure S4.B2. Scheme of gilt-silver coating layering on membrane strips. Spectra where acquired either on gold areas (Au areas) and on silver areas (Ag areas), where the silver layer underneath the gold was exposed and the top gold layer worn.

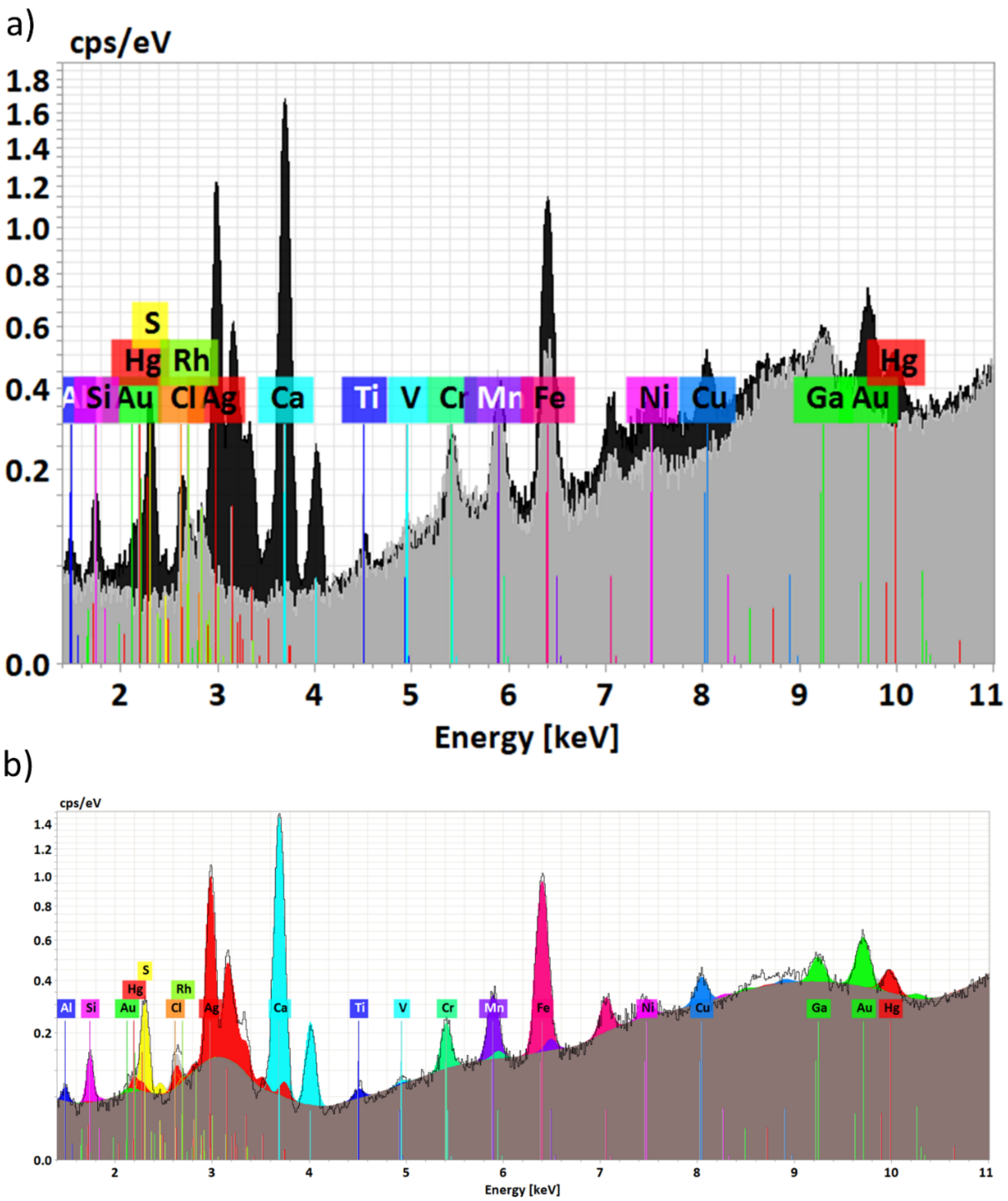

Figure S4.B3. Mercury detection by SEM-μXRF on gilt-silver leaf coatings. Sample **1902-1-241a_2**: a) Sum spectra from the sample in black and sum spectra from background aluminum stub in gray; b) Presence of Hg verified from deconvoluted peak at 9.989 keV for the Hg Lα1 line. V, Cr, Mn, and Ga are artifact peaks from the aluminum stub visible because of the greater depth penetration in μXRF.

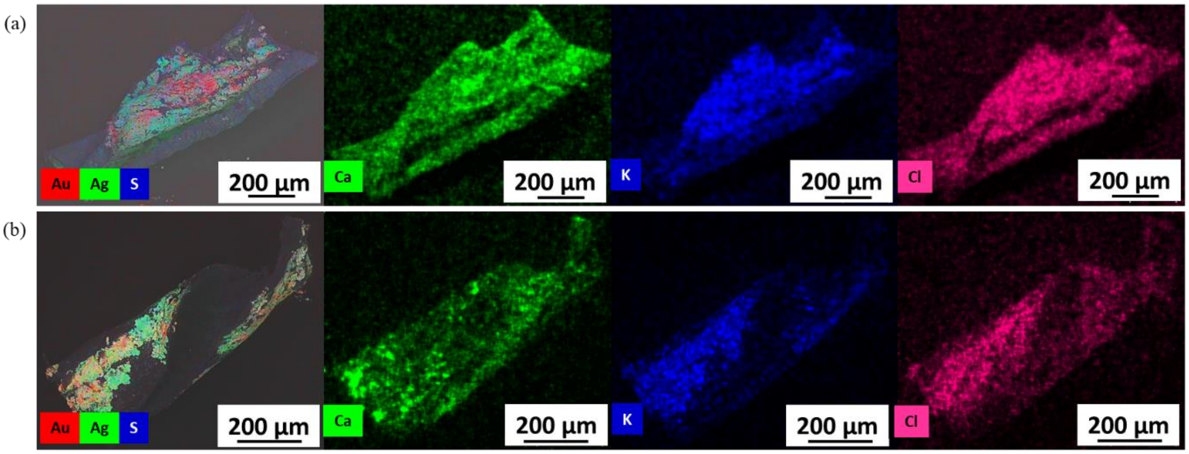

Figure S4.B4. EDS surface elemental mapping of the membrane strip. EDS maps acquired at 15kV (scale bar 200µm). From left to right: composite map showing the distribution of gold (in red), silver (in green), and sulphur (in blue) distributed on the metal surface; calcium (Ca) distribution map in green; potassium (K) distribution map in blue; chlorine (Cl) distribution map in pink. Calcium, potassium and chlorine appeared homogeneously distributed on the membrane strip beyond the metal surface: a) sample **1902-1-329a_1**; b) sample **1902-1-257b_1**.
Images by Thomas Lam © Museum Conservation Institute, Smithsonian Institution

As shown in **Fig. S4.B4**, by the EDS surface mapping of the strips, a few elements were partly associated to the substrate, most likely as residuals of the membranous strip treatments rather than environmental contamination, namely, calcium, potassium, and chlorine. It is known that gut membranes were washed and treated by soaking in weak lime and sodium hypochlorite or potassium water (potash) baths, as in goldbeater's skin making [2,3].

4.C SEM-EDS and SEM-µXRF analysis: Skin-based metal threads

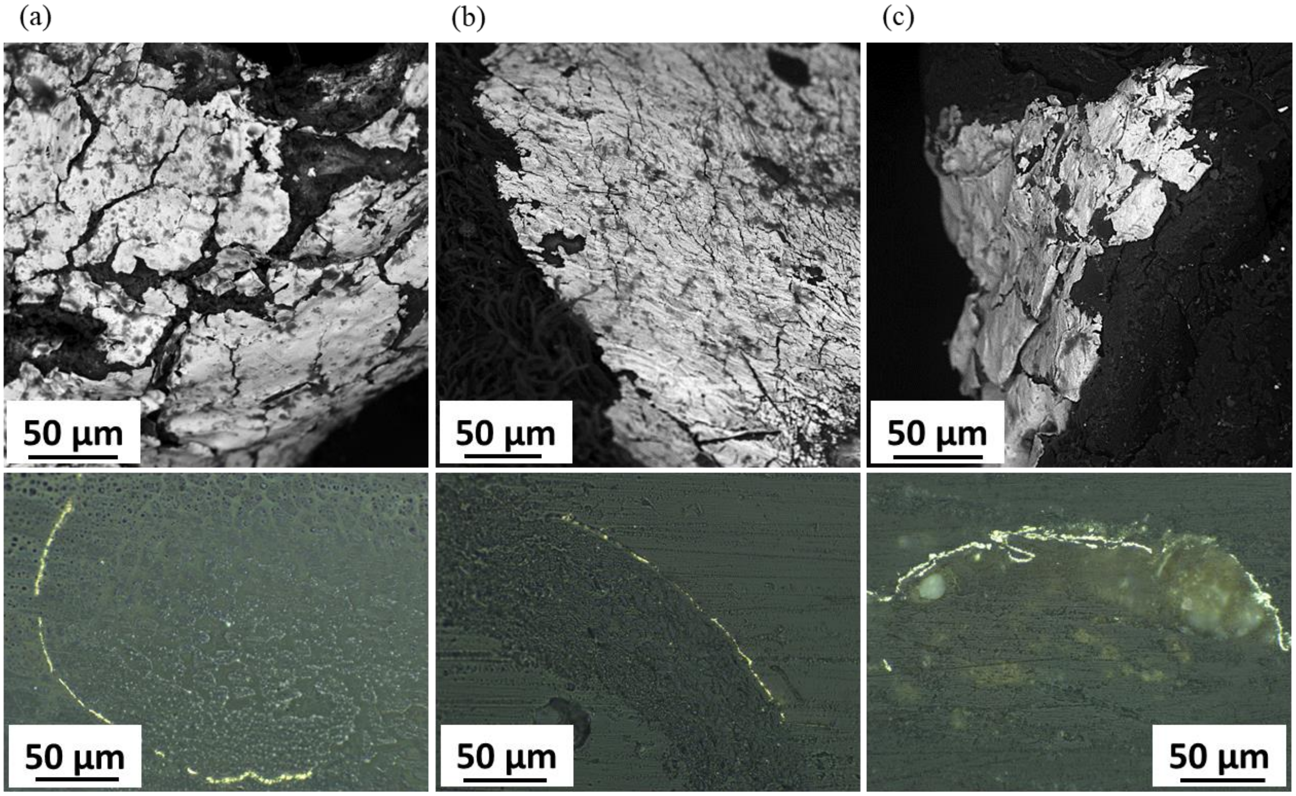

Figure S4.C1. Metal coating morphology on skin-based strips. From top to bottom: SEM-BSE images (scale bar 50µm), cross-sectional BF images (scale bar 50µm). a) Leaf-made coating, sample **1902-1-977c_3**; b) Leaf-made coating, sample **1902-1-292a**; c) Powder-made coating, sample **1902-1-310_2**. SEM-BSE images by Thomas Lam © Museum Conservation Institute, Smithsonian Institution; BF images by Cristina Scibè © Museum Conservation Institute, Smithsonian Institution.

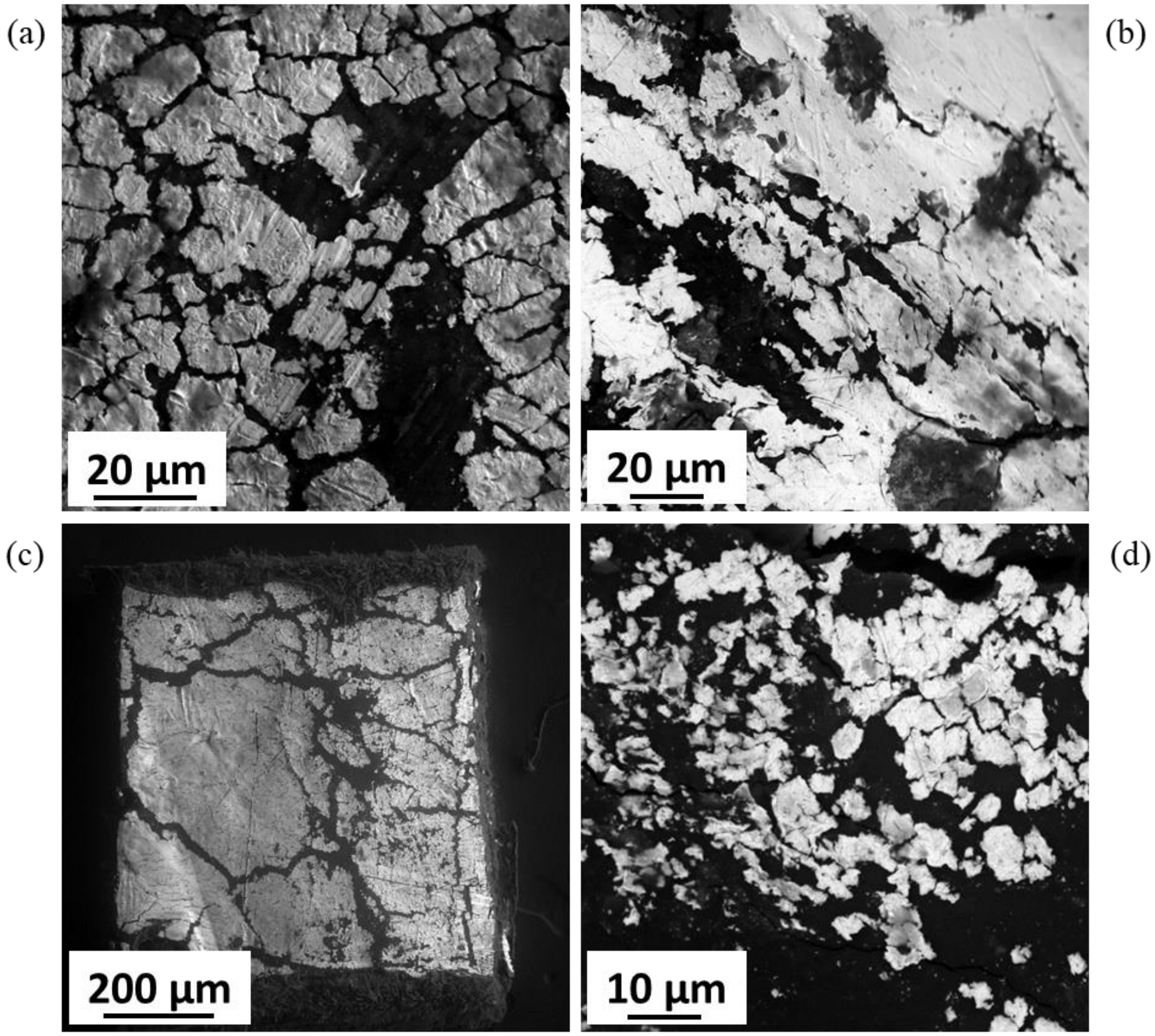

Figure S4.C2. Burnishing marks and "craquelure" of the metal leaves surface on skin-based strips: a) SEM-BSE image (scale bar 20µm), almost pure gold coating, sample **D13a;** b) SEM-BSE image (scale bar 20µm), gold-silver alloy coating, sample **1943-20-1b_2**; c) SEM-BSE image (scale bar 200µm), almost pure gold coating, sample **1862:16 I**; d) SEM-BSE image (scale bar 10µm), almost pure gold coating, sample **1902-1-251_1**. A remarkable net of cracks, resembling paintings "craquelure" [4] was observed in most of the coatings, regardless their elemental composition, possibly induced by the skin substrates and adhesive grounds beneath, which naturally cracked and broke the above thin metal surface, inducing sometimes a significant degradation and delamination of the leaves. Images by Thomas Lam © Museum Conservation Institute, Smithsonian Institution.

Table S4.C1. Elemental characterization of the metal coatings in skin-based threads (wrapped and flat strips). Detail by sample of the SEM-EDS/μ-XRF qualitative analysis of the elements detected as associated to the metal composition. The SEM-EDS semi-quantitative analysis of the main elements, gold (Au) and silver (Ag), and just in a case of copper (Cu) as minor element, is reported. The average concentrations are expressed in atomic weight percentage (wt%). Sulphur (S) and chlorine (Cl) were quantified solely in silver (group I), gold-silver-copper alloy (group II.3), and gilt-silver (group III) coatings, as associated to silver degradation products. The metal medium (leaf or powder) is indicated as identified by SEM-BSE analysis, with the corresponding composition, which for gold coatings (group II) is expressed in carats (k). Finally, the classification of the coatings is given (refers to **Table S4.C2**). (*) μ-XRF data; gl = gold layer; sl = silver layer; f = front of the strip; b = back of the strip; S= silver; GS= gilt-silver

Sample	Main elements		Degradation products		Minor and trace elements	Metal medium		Classification
	Au	Ag	S	Cl		Leaf	Powder	Group/ Subgroup
1902-1-229b_2	0,96	85,35	13,69		(*) Cu, Fe, Zn, Ti	S		I
1965-33-5_1	96,51	3,49			(*) Cu, Fe, Zn, Ni, Ti	23,16k		II.1.c
1965-33-2_2	87,07	12,93			Fe	20,89k		II.2.b
1902-1-977c_3	90,92	9,08				21,82k		II.2.b
1938-78-1_2	82,26	17,74			Fe	19,74k		II.2.c
1943-20-1b_2	85,33	14,67				20,48k		II.2.c
1902-1-310_2	25,88	68,28	4,54	1,30	(*) Cu, Fe, Ni, Ti		GS	III
1902-1-311_1 gl	42,09	49,87	5,27	2,77	(*) Cu, Fe, Pb, Ni, Ti	GS		III
1902-1-311_1 sl	22,42	66,80	8,11	2,67				
1902-1-385_2	99,37	0,63			(*) Cu, Fe	23,85k		II.1.a
1902-1-273a	99,15	0,85				23,80k		II.1.a
D12b	98,04	1,96				23,53k		II.1.b
1902-1-262_2	98,86	1,14			(*) Cu, Fe, Ni, Ti, Pb	23,73k		II.1.b
11902-1-272_2	98,4	1,6				23,62k		II.1.b
1902-1-251_1	98,04	1,96			(*) Cu, Fe, Ni, Ti	23,53k		II.1.b
1902-1-271a_1	98,76	1,24			Fe	23,70k		II.1.b
1902-1-285_1	98,63	1,37			Fe	23,67k		II.1.b
1902-1-292a	98,42	1,58			(*) Cu, Fe, Ni, Ti	23,62k		II.1.b
1902-1-292b	98,54	1,46			(*) Cu, Fe, Hg, Ni	23,65k		II.1.b
D13a	99,73	0,27			Fe	23,94k		II.1.a
P4c	99,47	0,53			Fe	23,87k		II.1.a

Table S4.C1. (continued)

Sample	Main elements		Degradation products		Minor and trace elements	Metal medium		Classification
	Au	Ag	S	Cl		Leaf	Powder	Group/ Subgroup
P4d	97,94	2,06			Fe	23,51k		II.1.b
1862: 16 I	99,17	0,83			Fe	23,80k		II.1.a
1862: 16 II	98,86	1,14			Fe	23,73k		II.1.b
1862: 16 III (f)	96,86	3,14			Fe	23,25k		II.1.c
1862: 16 III (b)	0,97	84,01	13,29	1,09		GS		III
1862: 16 IV (f)	94,43	5,57			Fe, Pb	22,66k		II.2.a
1862: 16 IV (b)		86,26	10,75	2,48		GS		III
1862: 16 V		91,63	7,94	0,44		S		I
1902-1-233	72,61	16,50	4,88	2,43	Cu (3,58), Fe	17,43k		II.3

Table S4.C2. Coatings compositional groups and subgroups in skin-based threads. Metal coatings were classified according to the SEM-EDS semi-quantitative analysis of the main metal elements, gold and silver. Three main groups (I to III) were identified: silver coatings, gold coatings and gilt-silver coatings. Then, according to the gold content, 7 sub-groups were further defined within group II (1a-c, 2a-c and 3) based on the gold purity expressed in carats (k). In detail: almost pure gold coatings (II.1), having a gold content ranging from 99,73wt%, corresponding to 23,94 carat (ca. 24k), to 96,51wt%, corresponding to 23,16 carat (ca. 23k); gold-silver alloy coatings (II.2), having a gold content ranging from 94,43wt%, corresponding to 22,66 carat (ca. 22k), to 82,26wt%, corresponding to 19,74 carat (ca. 20k); gold-silver-copper alloy (II.3), with a gold content of 72,61wt%, silver of 16,50wt% and copper of 3,58wt%. Within the silver coatings group, the gold concentration below 2wt%, is in agreement with the literature on European medieval and post-medieval silver [5].The presence of gold in silver metals is an indicator of the silver source and one of the most important trace elements for silver authenticity. Within group III, gilt-silver coatings, a distinction was made according to the medium used: leaf or powder. #samples= number of samples belonging to the group/subgroup, wt%=weight percentage, k= carat.

Groups	I	II							III	
	**Silver coatings**	**Gold coatings**							**Gilt-silver coatings**	
# samples	2	22							4	
Subgroups	Au < 2wt%	**1. Almost pure gold**			**2. Gold-silver alloy**			**3. Gold-silver-copper alloy**	**Leaf**	**Powder**
		**a**	**b**	**c**	**a**	**b**	**c**	ca. 18k (17,43k)		
		ca. 24k (>=23,80k)	23-24k (23,51-23,73k)	ca. 23k (23,16-23,25k)	ca. 22k (22,66k)	ca. 21k (20,89-21,82k)	ca. 20k (19,74-20,48k)			
# samples	2	5	10	2	1	2	2	1	3	1

Table S4.C3. Gold coatings (group II). Average concentration values for gold (Au) and silver (Ag) calculated for each provenance group of the objects sampled. The average values are expressed in atomic weight percentage (wt%), and are reported with the associated standard deviation, minimum and maximum value calculated. Far Eastern flat-strips as well as Hispano-Islamic wrapped-strips showed a broader variety of coating compositions, as indicates the standard deviation value associated to gold concentration average measurements of respectively 9,17wt% and 5,50wt%. Conversely, all the wrapped-strips belonging to Middle Eastern and/or Italian objects showed a quite homogeneous composition, indeed the associated standard deviation to the gold concentration measurements was of 0,43wt%.

**Objects provenance**	**Au**				**Ag**			
	**Av.**	**SD**	**Min**	**Max**	**Av.**	**SD**	**Min**	**Max**
Islamic Spain	88,42	5,50	82,26	96,51	11,58	5,50	3,49	17,74
Middle East (Iran or Iraq) and/or Italy	98,62	0,43	98,04	99,37	1,38	0,43	0,63	1,96
Far East (East and Central Asia/China)	94,88	9,17	72,61	99,73	3,76	5,44	0,27	16,50

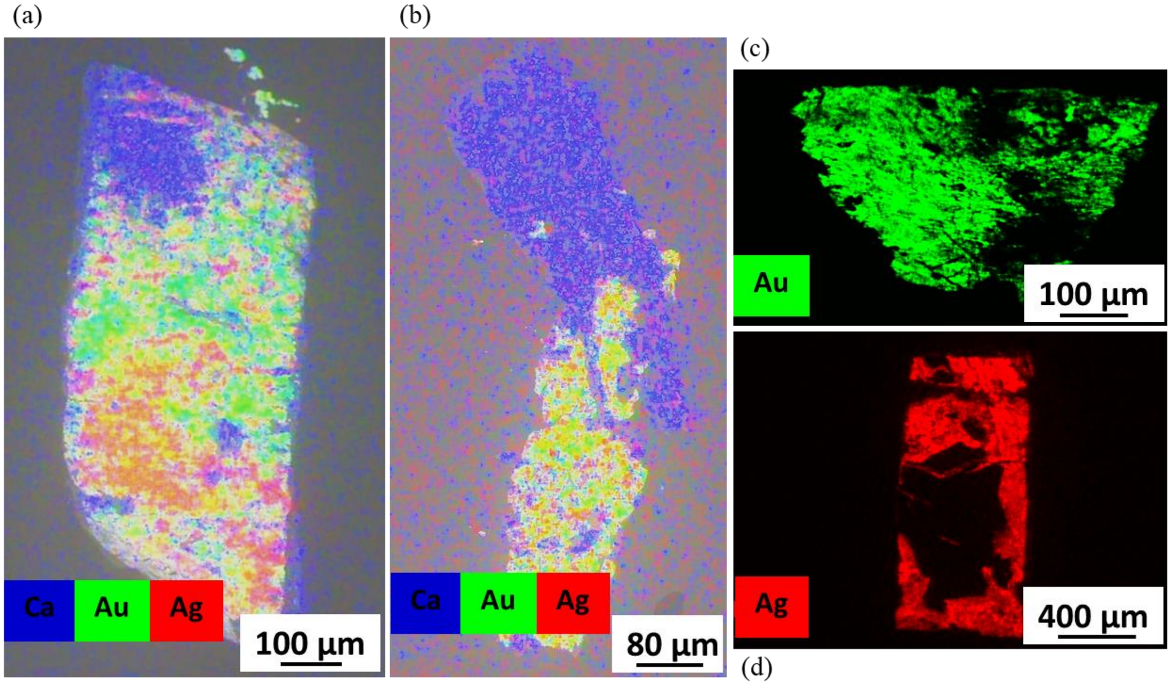

Figure S4.C3. Coatings compositional groups in skin-based strips: EDS surface elemental mapping: a) Gilt-silver coating (group III), sample **1902-1-311_1**. Composite map acquired at 15kV (scale bar 100µm), showing the distribution of gold (in green) on top of silver (in red) in a bi-layered coating, and calcium (in blue) in correspondence of the skin substrate; b) Gold coating (group II), gold-silver alloy (II.2), sample **1965-33-2_2**. Composite map acquired at 15kV (scale bar 80µm), showing the distribution of gold (in green) and silver (in red) in correspondence of the same layer, and calcium (in blue) in correspondence of the skin substrate; c) Gold coating (group II), almost pure gold coating (II.1), sample **1902-1-292a**. Gold distribution map (Au) acquired at 15kV (scale bar 100µm); d) Silver coating (group I), sample **1862:16 V**. Silver distribution map (Ag) acquired at 15kV (scale bar 400µm).

a)

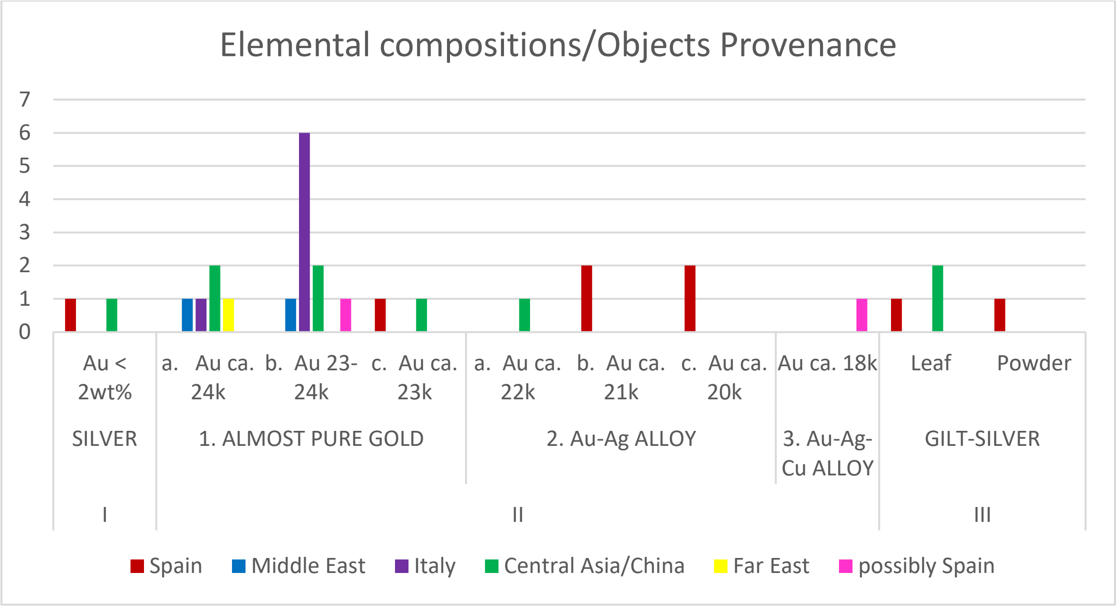

b)

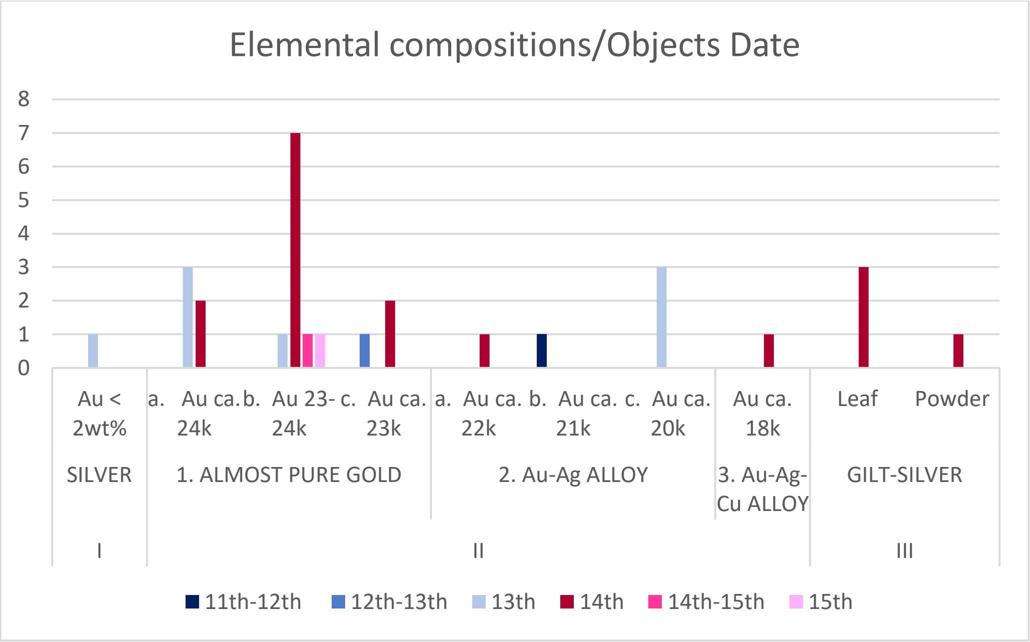

Figure S4.C4. Correlation between the metal coating composition of the samples and the corresponding objects provenance and date. a) Detail by coatings composition group and subgroups of the number of samples identified per each geographical area. b) Detail by coatings composition group and subgroup of the number of samples identified per each time period.

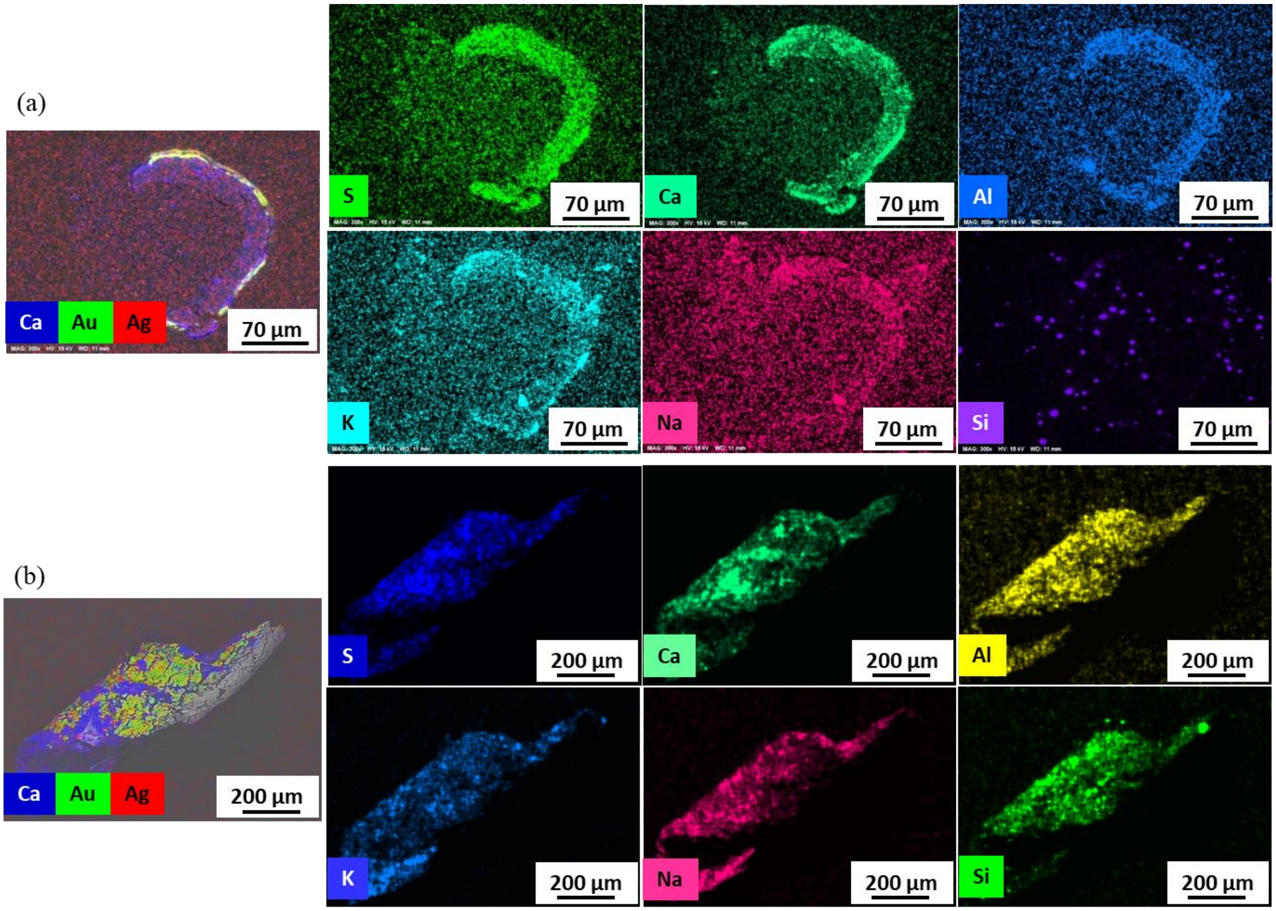

Figure S4.C5. EDS elemental mapping of the skin strip: cross-section VS surface analysis. EDS maps acquired at 15kV on sample **1902-1-977c_3:** a) EDS cross-section mapping (scale bar 70µm). From left to right: composite map showing the distribution of gold (in green) and silver (in red) along the metal layer, and calcium (in blue) on the skin substrate; elemental distribution maps of sulphur (S), calcium (Ca), aluminum (Al), potassium (K), sodium (Na) and silicon (Si); b) EDS surface mapping (scale bar 200µm). From left to right: composite map showing the distribution of gold (in green) and silver (in red) on the metal surface, and calcium (in blue) on the underneath skin substrate; elemental distribution maps of sulphur (S), calcium (Ca), aluminum (Al), potassium (K), sodium (Na) and silicon (Si).

As shown in **Fig. S4.C5**, by the EDS surface mapping (and cross-section mapping attempted in a few samples), calcium, potassium, sulphur, and in some cases, sodium, chlorine, and aluminum were found to be distributed in correspondence to the strip substrate. These elements may be potentially residuals of the raw hide treatments (washing, salting, depilation, pre-tanning and tanning processes), beyond the skin composition itself (sulphur presence in collagen-based materials) or environmental contamination.

Indeed, as largely reported in literature, the first phase of a raw skin treatment is the washing followed by wet salting in order to preserve it until putrefaction. Sodium sulphides (Na_2S) and lime ($Ca(OH)_2$) or chalk ($CaCO_3$) were also used as part of the depilatory process of hides. Moreover, the use of potassium alum salts ($KAl(SO_4)_2 \cdot 12H_20$), was largely reported either for skin tawing and in pre-tanning processes. Throughout tanning history, alum was often used in mixture with other materials, such as salt, egg yolk, olive oil and flour to prepare the hide to the tanning process, as well as to enhance the final colouring reactions [6,7]. In a former study, conducted on gilt leather flat-strips on early 20th century traditional Chinese children hats, the presence of calcium and potassium detected in the undersides of the strips was related to the processing of the leathers [8]. Moreover, the use of potassium alum salts was also attested in a recent investigation conducted on Japanese paper-based metallic threads on a collection of Japanese samurai armors, to treat the paper in addition to clay or calcium carbonate and animal glue, with the aim to give additional body ad strength to the paper support [9].

BIBLIOGRAPHY

1 Wu, Q. *et al.* Does substrate colour affect the visual appearance of gilded medieval sculptures? Part II: SEM–EDX observations on gold leaf samples taken from medieval wooden sculptures. *Herit. Sci.* **8**, 119; 10.1186/s40494-020-00456-2 (2020).

2 Enciclopedia del negoziante ossia Gran Dizionario del Commercio, dell’Industria, del Banco e delle Manifatture 478-480 (Guis, Antonelli, 1840).

3 de Groot, Z. H. & Danforth, C. The Manufacture of Goldbeater’s Skin, Transparent Parchment, and Split Parchment. *Art in Translation* **13**, 408-418 (2021).

4 Bucklow, S. *Formal connoisseurship and the characterisation of craquelure*, University of Cambridge (1996).

5 Craddock, P. Gold and Silver in *Scientific Investigation of Copies, Fakes and Forgeries (1st ed.)* (ed P. Craddock) 369-393 (Routledge, 2009).

6 Covington, A. D. The chemistry of tanning materials in *Conservation of leather and related materials* (eds Marion Kite & Roy Thomson) 22-35 (Butterworth-Heinemann, 2006).

7 Thomson, R. The manufacture of leather in *Conservation of leather and related materials* (eds Marion Kite & Roy Thomson) 66-81 (Butterworth-Heinemann, 2006).

8 Cheung, A., Solazzo, C. & Tsui, W.-s. Unveil the Gold – Revealing Metal Threads and Decorative Materials of Early Twentieth Century Traditional Chinese Children's Hats. *Stud. Conserv.* **66**, 357-374 (2021).

9 Geminiani, L. *et al.* Unveiling the Complexity of Japanese Metallic Threads. *Heritage* **4**, 4017-4039 (2021).
